# Dehydrocoupling Polymerization: Poly(silylether) Synthesis by Using an Iron β‐Diketiminate Catalyst

**DOI:** 10.1002/chem.202201642

**Published:** 2022-09-08

**Authors:** Mirela A. Farcaş‐Johnson, Sara H. Kyne, Ruth L. Webster

**Affiliations:** ^1^ Department of Chemistry University of Bath Claverton Down Bath UK; ^2^ School of Chemistry Monash University Clayton Victoria 3800 Australia

**Keywords:** dehydrocoupling, homogeneous catalysis, iron, polymerizations, poly(silylether)s

## Abstract

We describe the iron‐catalyzed polymerizations of diol and silane monomers to obtain fourteen different poly(silylether) products with number average molecular weights (*M*_n_) up to 36.3 kDa. The polymerization reactions developed in this study are operationally simple and applicable to 1° and 2° silane monomer substrates and a range of benzylic and aliphatic diol substrates as well as one polyol example. The polymers were characterized by IR spectroscopy, DSC and TGA and, where solubility allowed, ^1^H, ^13^C{^1^H}, ^29^Si{^1^H} NMR spectroscopies, GPC and MALDI‐TOF were also employed. The materials obtained displayed low *T*_g_ values (−70.6 to 19.1 °C) and were stable upon heating up to *T*_–5%,Ar_ 421.6 °C. A trend in *T*_–5%,Ar_ was observed whereby use of a 2° silane leads to higher *T*_–5%,Ar_ compared to those obtained using a 1° silane. Reaction monitoring was undertaken by in situ gas evolution studies coupled with GPC analysis to follow the progression of chain‐length growth which confirmed a condensation polymerization‐type mechanism.

## Graphical abstract

An efficient iron catalyzed method for the formation of poly(silylether)s has been developed, where the only by‐product of the reaction is hydrogen gas. Fourteen different polymers have been prepared, which show a range of different thermal properties. Insight into the reaction, which proceeds via a condensation polymerization mechanism is provided.

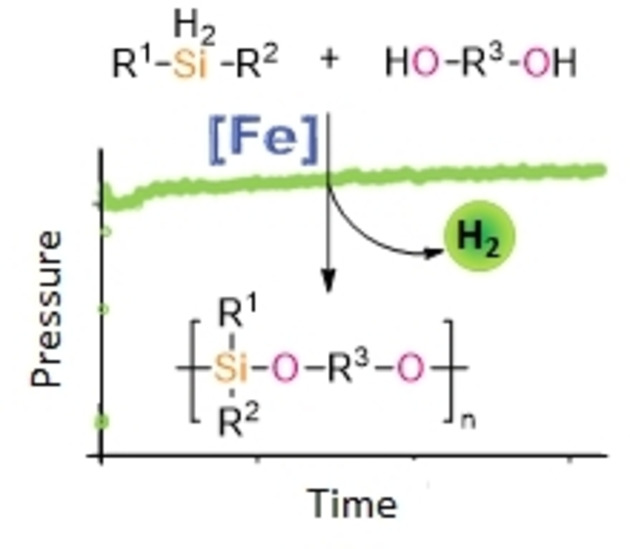

## Introduction

The global production and usage of silicon‐based polymers has increased vastly due to their interesting physical and electronic properties, and as an alternative to carbon‐based polymers.^[1]^ The surge in demand for more sustainable and readily recyclable silicone polymers has seen interest build in the synthesis and catalytic break down of these materials.^[2]^ Due to their high temperature and chemical stability, Si−O‐based polymers are desirable in the chemical, materials and medical industries.^[3]^ Poly(silylether)s are just one of the numerous types of Si−O polymers. With a particularly mobile Si−O−C backbone linkage, this subclass of silicon polymers is highly flexible and durable, and by the addition of bulkier substituents on the backbone of the chain, developments and improvements to their properties have been possible.^[1a]^ Poly(silylether)s have a diverse array of applications, for example, they have been used to improve solubility in carbon dioxide^[4]^ and as electrolyte additives in batteries.^[5]^ Furthermore, poly(silylether)s have been proposed for space applications,^[6]^ applications in the microelectronics industry as high‐performance polymers^[7]^ and as drug delivery materials,^[8]^ whilst enantiopure poly(silylether)s show promising applications as chiral separation materials.^[9]^

**Scheme 1:**
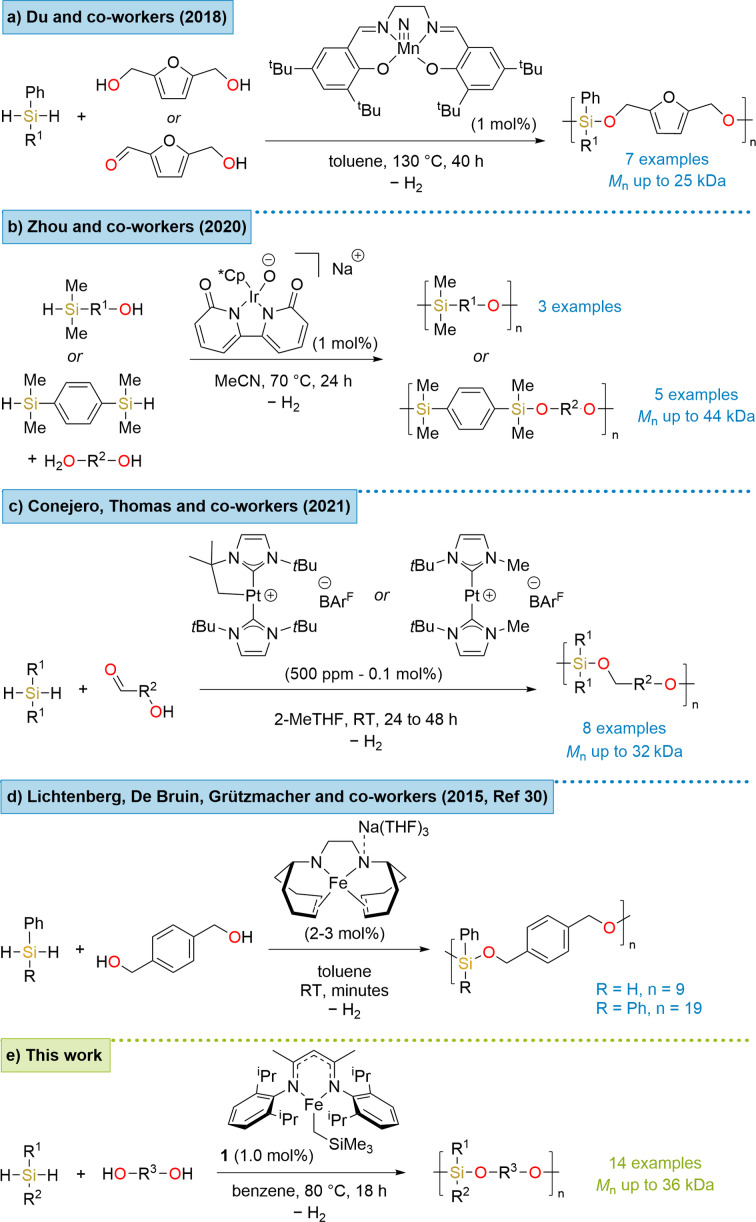
a‐d) Previous reports on transition metal‐catalyzed dehydrocoupling to prepare poly(silylether)s; e) our iron catalyzed method presented here.

Recent developments in transition‐metal catalyzed dehydrocoupling for the synthesis of these polymers include Du and co‐workers use of an air‐stable Mn(V) salen complex to react various bioderived furan‐containing monomers with hydrosilanes (Scheme 1a).^[10]^ The catalyst was able to mediate both the dehydrogenative cross‐coupling of alcohols with hydrosilanes and the hydrosilylation of carbonyls. A few years later, Zhou and co‐workers demonstrated the use of an air‐stable anionic iridium catalyst that was also able to catalyze the synthesis of some partially biobased polymers (using monomers of the form AA and BB or AB, Scheme 1b).^[11]^ Later the same year, they also applied Co(acac)_2_ with the addition of a 1,5‐bis(diphenylphosphino)pentane (dpppe) ligand to mediate the same transformations.^[12]^ This was undertaken in a straightforward one‐pot, two‐step procedure. Hill, Manners and co‐workers have used group 2 pre‐catalysts to prepare poly(silylether)s containing a ferrocenyl pendant group.^[13]^ Most recently, Conejero, Thomas and co‐workers applied two different NHC Pt(II) complexes to copolymerize hydrosilanes with various difunctional hydroxyaldehyde monomers. The reaction conditions were mild with very low catalyst loading (Scheme 1c).^[14]^ However, there is currently a paucity of iron catalysts applied to poly(silylether) synthesis in the literature. Elegant studies from Lichtenberg, de Bruin and Grützmacher into the chemistry of low valent Fe(I) complexes have employed these complexes in the dehydrocoupling polymerization of phenylsilane or diphenylsilane with 1,4‐benzenedimethanol (Scheme 1d).^[15]^ The development of iron catalysis for the synthesis of poly(silylether)s would reduce the present global dependency on precious metal catalysis as well as provide a less toxic and expensive route to these new materials. In previous work, we published a method for the heterodehydrocoupling of silanes with alcohols to form various silylether monomers and only one example of a tetrameric unit using **1** as the iron pre‐catalyst.^[16]^ Herein we show the versatility of the iron pre‐catalyst (**1**) and report our development of an efficient methodology to prepare high *M*_n_ poly(silylether)s (Scheme 1e).

## Results and Discussion

Our previous report was used to furnish short chain poly(silylether)s, but the reaction in its published form was clearly inefficient and only generated units that were dimeric to tetrameric in length. We considered that with appropriate development of reaction conditions and work‐up, it would be possible to apply the dehydrocoupling procedure to expand the scope of the reaction up to polymers, with improved *M*_n_ and *Ð*, and thus be able to study the properties of our small library of poly(silylether)s.

We initiated our optimization by using 1,4‐benzenedimethanol and phenylsilane as the monomer substrates in the presence of **1** (5 mol%) (Table 1, Entry 1). After 2 h, the reaction affords an insoluble product that cannot be characterized by GPC. Use of a secondary silane, methylphenylsilane, generates linear products which are far easier to characterize and thus, with a reduced catalytic loading of 1 mol% and 1 : 1 ratio of secondary silane:diol at 80 °C, modest *M*_n_ polymer (**2 a**) is obtained (Entry 2). Unfortunately, no reaction is observed at lower catalyst loading at RT (Entry 3). After further optimization (Entries 4 to 7), we arrived at a standard reaction procedure of diol:silane in a 1 : 2 ratio, 1 mol% **1**, 1 mL C_6_D_6_, 80 °C, 18 h (Entry 6). Although a higher catalyst loading leads to higher *M*_n_, we feel that the need to use more catalyst is not offset by the fairly modest increase in *M*_n_ (Entry 7). It is worth noting that the reactions do not proceed in J‐Young NMR tubes presumably due to build of H_2_, which limits the forward polymerization reaction. Efficient work‐up is carried out by handling under an inert atmosphere. Insoluble products are washed with pentane to remove short chain length species and any iron catalyst‐based residues.

**Table 1:** Polymerization optimization procedure using 1,4‐benzenedimethanol, methylphenylsilane and **1**.

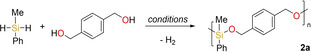					
*Entry*	*1,4‐benzenedimethanol* [equiv.]	*MePhSiH_2_* [equiv.]	***1*** [mol %]	*T* [°C]	***2 a** M*_n_ [Da]
1^[a]^	3	1.00	10	80	*Insoluble*
2	1	1.00	1	80	1 440
3	1	1.25	0.5	RT	NR^[b]^
4	1	1.75	10	70	5 421
5	1	1.75	10	80	12 321
6	1	2.00	1	80	21 546
7	1	2.00	5	80	24 657

[a] Phenylsilane employed. [b] No reaction detected by ^1^H NMR spectroscopy.

**Scheme 2:**
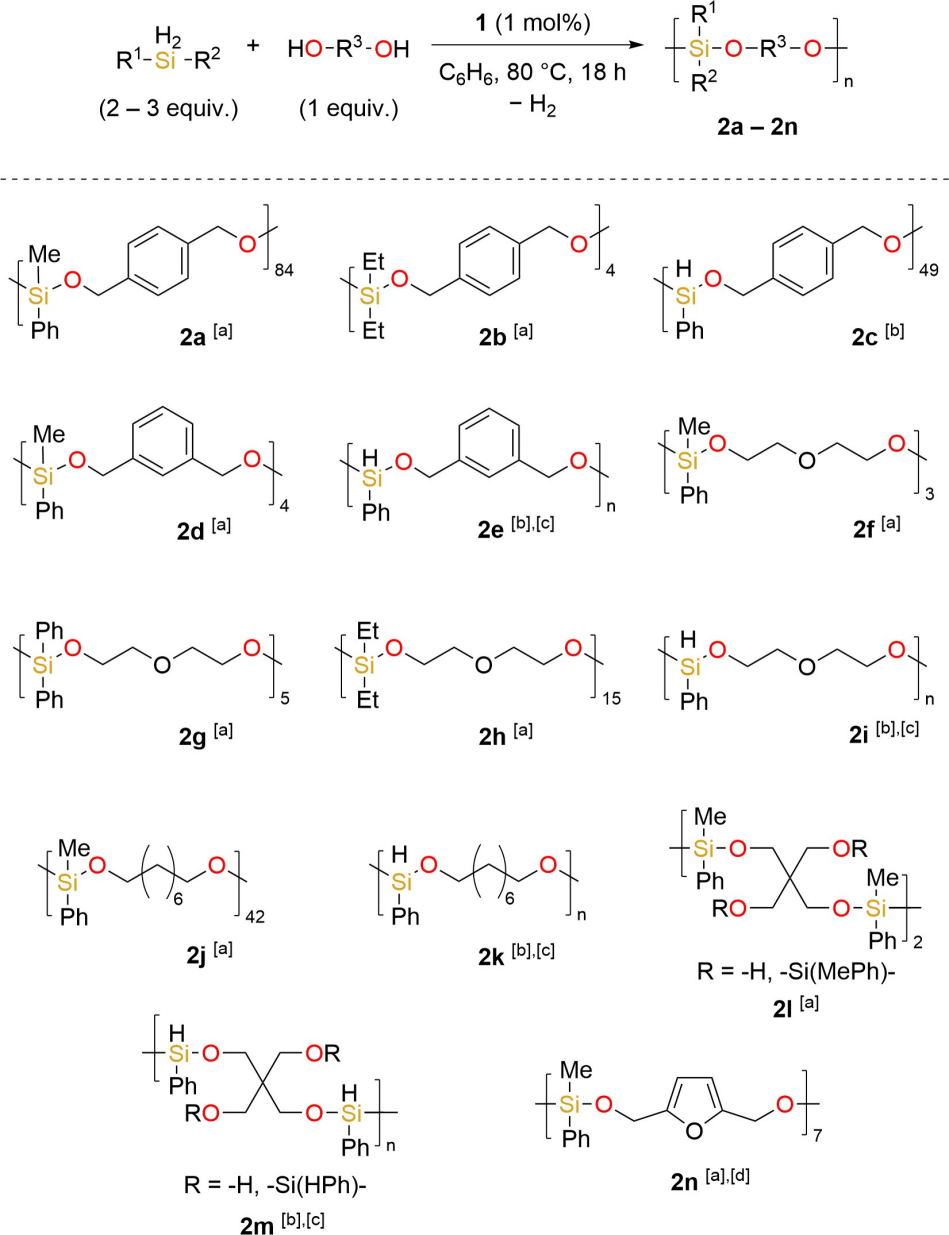
Scope and properties of poly(silylether) products. Conditions: diol/polyol (0.4 mmol, 1 equiv.), silane (0.8 to 1.2 mmol, 2 to 3 equiv.),**1** (1 mol%), C_6_D_6_ (1 mL), 80 °C, 18 h. [a] silane (0.8 mmol, 2 equiv.) [b] silane (1.2 mmol, 3 equiv.) [c] insoluble product, only solid‐state analysis available. [d] 4 mol% **1**.

We set out to expand our library of poly(silylether)s and have furnished a range of polymer products **2 a** to **2 n** (Scheme 2). In each case, the formation of a Si−O bond is confirmed by IR spectroscopy, with an indicative stretch around 1057 cm^−1^.^[17]^ For products with sufficient solubility, GPC was used to determine *M*_n_ and *Ð*, while ^1^H, ^13^C{^1^H} and ^29^Si{^1^H} NMR characterization of the isolated products along with MALDI‐TOF data is provided where possible. For all polymer samples a clear loss in Si–H multiplicity is observed, for example by ^1^H NMR spectroscopy MePhSiH_2_ appears as a distinctive quartet at 4.49 ppm in the monomer, but this is completely lost at the end of the reaction. Taking the ^1^H NMR spectrum of **2 a** as a specific example, this indicates a symmetrical repeating polymer chain with the methylene CH_2_ protons observed as a singlet at 4.76 ppm and the SiCH_3_ protons appearing as a new singlet at 0.30 ppm. MALDI‐TOF data for **2 a** confirms the polymer consists of silane and diol repeating units with methylphenylsilane and alcohol end groups i.e. the CH_2_TMS group from the pre‐catalyst is not incorporated into the polymer chain. Furthermore, TGA and DSC were obtained to provide data on the thermal properties of the samples. For products where solubility is a limiting factor only IR, TGA and DSC data are obtained.

The unactivated silane, diethylsilane, does not polymerize with 1,4‐benzenedimethanol as readily as methylphenylsilane (Table 2, compare Entries 1 and 2), but when phenylsilane and 1,4‐benzenedimethanol are reacted using our optimized reaction conditions **2 c** is produced as a viscous oil and with a slightly lower *M*_n_ compared to **2 a** (11.9 kDa for **2 c** *versus* 21.5 kDa for **2 a**, Table 2, Entries 3 and 1). 1,3‐Benzenedimethanol is also tolerated in the reactions with our chosen 1° and 2° silanes to give products **2 d** and **2 e**. GPC data reveals a moderate average molecular weight for **2 d** indicative of a shorter, oligomeric compound (Table 2, Entry 4). For **2 e** (Table 2, Entry 5), no GPC data is available due to the insoluble nature of the rubbery solid. Applying our optimized conditions to the reaction of diethylene glycol and methylphenylsilane, **2 f** is prepared forming very moderate *M*_n_ material (Table 2, Entry 6). In contrast to our previously published example using phenylsilane (where the reported *M*_n_ is 944 Da),^[15a]^ using our optimized conditions, an insoluble product is afforded in the case of **2 i** (Table 2, Entry 9). To investigate whether a change in 2° silane can affect the polymer *M*_n_, diphenylsilane was employed as a substrate. Unfortunately, a similarly low *M*_n_ species is formed; oligomer **2 g** (Table 2, Entry 7), but this does confirm bulkier silane substrates are equally tolerated as methylphenylsilane. Interestingly, and in contrast to the results obtained with 1,4‐benzenedimethanol as the coupling partner, diethylsilane reacts particularly well with diethylene glycol to give **2 h** as a *M*_n_ polymer (3.2 kDa for **2 h** versus 0.9 kDa for **2 b**, Table 2, Entries 8 and 2). Indeed, this silane gives the highest *M*_n_ polymer with diethylene glycol (compare Table 2, Entries 6, 7 and 8). 1,8‐Octanediol is an example of a linear alkyl diol able to undergo reaction with methylphenylsilane generating **2 j** with a respectable *M_n_* of 10.6 kDa (Table 2, Entry 10), while reaction with phenylsilane gives an insoluble product **2 k** (Table 2, Entry 11).

**Table 2:** Polymer properties, determined by GPC, DSC and TGA.

*Entry*	*Product*	*M*_n_ [kDa]	*M*_w_ [kDa]	*Đ*	*T*_g_ [°C]^[a]^	*T*_*–5%*,Ar_ [°C]^[b]^	*T*_inf,Ar_ [°C]^[b]^
1	**2 a**	21.5	70.6	3.3	−15.4	368.8	442.3
2	**2 b**	0.9	1.2	1.3	*None*	365.8	439.4
3	**2 c**	11.9	21.9	1.8	−41.8	350.3	405.1
4	**2 d**	1.3	1.8	1.5	−52.8	384.1	427.3
5^[c]^	**2 e**	*Insol*.	*Insol*.	*Insol*.	1.37	376.1	360.8
							507.6
6	**2 f**	0.9	1.3	1.5	−70.6	332.7	436.8
7	**2 g**	1.6	2.3	1.5	−42.8	310.2	373.6
8	**2 h**	3.2	6.8	2.1	−5.5	245.5	300.1
9^[c]^	**2 i**	*Insol*.	*Insol*.	*Insol*.	−46.5	303.7	413.5
10	**2 j**	10.6	24.7	2.3	*None*	421.6	478.7
11^[c]^	**2 k**	*Insol*.	*Insol*.	*Insol*.	*None*	399.1	460.0
12	**2 l**	0.6	1.1	1.9	19.1	218.3	294.8
							451.5
13^[c]^	**2 m**	*Insol*.	*Insol*.	*Insol*.	*None*	227.8	263.7
							453.4
14	**2 n**	1.8	2.9	1.7	*None*	234.8	242.6
							357.3

[a] Determined using DSC (−50 to +270 °C or −100 to +100 °C for oily samples, see Supporting Information) [b] Determined using TGA (+25 to +600 °C). [c] *Insol*.=insoluble sample.

To probe whether our polymerization procedure can be extended beyond diol substrates, we applied our reaction conditions to pentaerythritol with methylphenylsilane and phenylsilane to give products **2 l** and **2 m** (Entries 12 and 13). A small species with an average of two repeating units was formed as product **2 l**. It is suspected that further polymerizations are hindered by steric bulk around the silicon atom caused by the large polyol unit. The product of pentaerythritol and phenylsilane gives a highly insoluble product in the form of **2 m** which could not be analyzed by GPC. Of the polymers generated, those formed from 1° phenylsilane are more likely to produce a highly insoluble material due to the multiple sites for polymerization, allowing for polymer branching and network formation, which hinder the ability for solvent to enter the network and dissolve the polymers. **2 c** is an exception to this.

Finally, the bio‐based diol, 2,5‐bis(hydroxymethyl)furan, reacts reasonably well to give a modest *M*_n_ product on reaction with methylphenylsilane (Table 2, Entry 14).

In terms of thermal properties (see Table 2, *T*_g_, *T*_‐5%,Ar_ and *T*_inf,Ar_), **2 a** has the largest *M*_n_ as well as a moderate value for *T*_g_ (−15.4 °C), whilst (with the exception of small oligomeric product **2 l**) **2 f** has the second lowest *M*_n_ and lowest recorded *T*_g_ (−70.6 °C). Regarding thermal transition events obtained from DSC, **2 c**, **2 d**, **2 g** and **2 i** all have similar *T*_g_ values within the same negative region (−52.8 to −41.8 °C). This is consistent with their oily nature. Products **2 b**, **2 j**, **2 k**,**2 m** and **2 n** show no evidence of glass transition events within the range of temperatures studied (−50 to +270 °C) However, in the case of **2 l** and **2 m**, two other thermal events are observed: there is potential cold crystallization^[18]^ at 158.3 °C for **2 l** (Table 2, Entry 12) and in the case of **2 m** (Table 2, Entry 13) there is a *T*_c_ at 121.4 °C and a potential *T*_m_ event at 175.6 °C. There is no evidence of correlation between the insoluble nature of the polymers and their thermal transition temperatures.

The thermal stability of the products isolated are, in general, pleasingly high with the maximum rate of degradation occurring at inflection points (*T*_inf,Ar_) of 242.6 to 478.7 °C (Table 2). We note that the phenylsilane polymers are less stable than their methylphenylsilane counterparts in all cases, with maximum degradation occurring at lower temperature (e.g., compare Table 2, Entries 1 and 4 to Entries 3 and 5). The TGA traces of polymers **2 a**, **2 h** and **2 j** show one distinct degradation event (see Supporting Information) each with a total mass decrease of 87 %, 96 % and 77 % respectively after the experiment. In contrast, the other polymers show more than one additional minor degradation event across the temperature range. Any mass decrease events at temperatures of 200 °C and below are attributed to solvent loss or loss of smaller and more volatile oligomers than the bulk sample. We hypothesize that for the network polymer examples **2 e**, **2 l** and **2 m**, which have two relatively high degradation events (see Table 2, Entries 5, 12 and 13), this could be attributed to either a) two different species within the polymer mixture having different degradation thresholds as shown by the two mass loss events or b) two degradation steps within the same mechanism where firstly one group of bonds is broken and the volatile products are lost leaving behind the remainder of the polymer chain, which finally degrades at a higher temperature. It is reasonable to assume that the same degradation mechanism occurs for **2 l** and **2 m** as they have very comparable mass loss event temperatures.

MALDI‐TOF data was obtained for a number of the products in addition to **2 a** (see above). The end groups are also a silane and an alcohol group, suggesting that all polymerizations follow a similar mechanism of reaction, and the spectral fingerprint is synonymous with a condensation, i.e. chain growth, mechanism. Interestingly, the repeating unit for polymer **2 c** is higher than expected by a value of 78 Da. This corresponds to a benzene molecule, which may be trapped within the polymer structure. It is possible that the benzene trapped within the polymer network assists with the solubility of **2 c** and could explain the unexpected solubility of **2 c** compared to **2 e**, **2 i**, **2 k** and **2 m**.

When 1,8‐octanediol and phenylsilane are reacted to give **2 k**, the polymer has an interesting property in that it swells upon the addition of solvents including benzene, THF and toluene. A study was undertaken in triplicate to investigate the swelling ability. This shows a ×2.2 increase in mass of the polymer when left to swell in toluene over a period of two days. The swelling is due to the ability of **2 k** to form polymer networks which branch from a central silicon atom. Interestingly, this swelling ability is not observed for other examples which would also form branched products (e.g. **2 c**, **2 e** and **2 i**). We can attribute this to the flexible nature of the aliphatic linker present in **2 k** which has a greater ability to retain solvent in its network structure in comparison to the more rigid linkers present in **2 c**, **2 e** and **2 i**.

**Figure 1:**
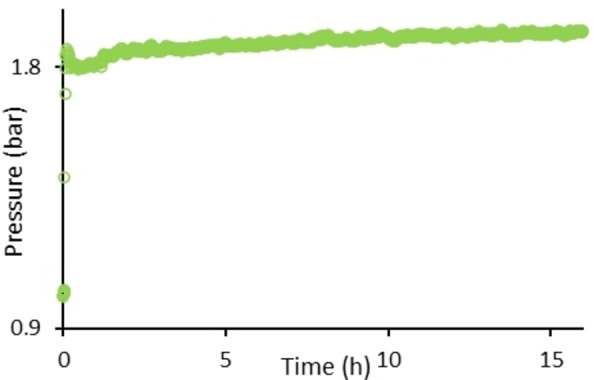
H_2_ gas evolution as a function of time during the synthesis of **2 k**.

We next set out to investigate the progression of gas evolution during a polymerization reaction. The gas evolution during the synthesis of **2 k** was monitored in situ under our standard conditions (Figure 1). Qualitatively, the reaction proceeds with a sudden spike in gas evolution within the first 10 min. This is followed by a plateau and gradual further increase until the 16 h endpoint. Such a reaction profile is likely to be indicative of a condensation polymerization mechanism, which undergoes initial formation of small molecule dimers and trimers during the early stages of polymerization, after which these slowly react together to gradually form higher molecular weight products. This is also in‐line with the relatively broad polydispersity index observed (see Table 2, Entries 1, 8 and 10).

**Figure 2:**
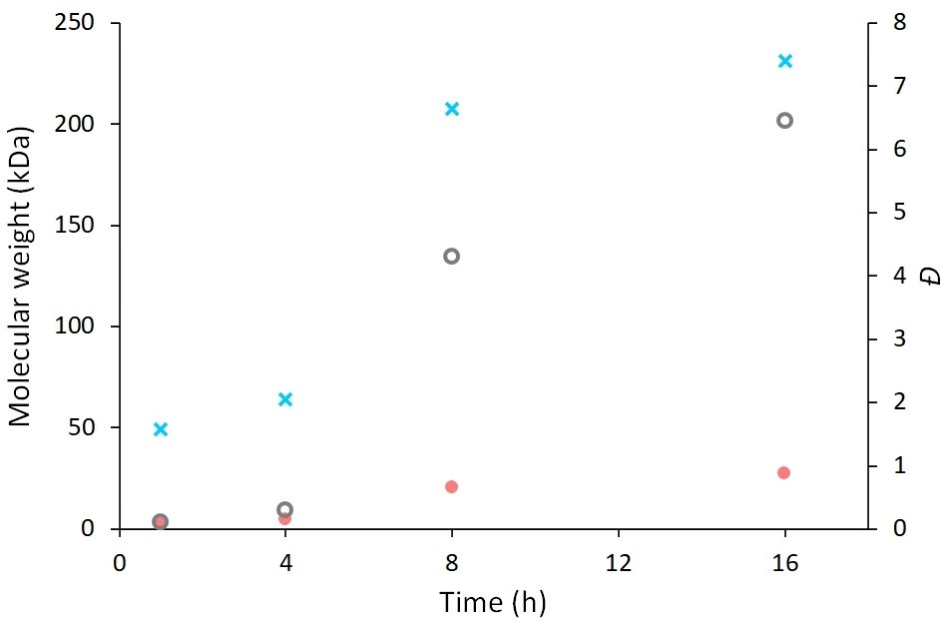
Increase in *M*_n_ (•), *M*_w_ (○) and *Ð* (×) as a function of time during the synthesis of **2 a**.

To probe this theory further, a set of experiments were carried out in parallel where the chain length growth of **2 a** was monitored by GPC analysis over a period of 16 h (Figure 2). The data shows small molecular weight species forming initially, followed by an increase in molecular weight over 12 h to reach a steady plateau at a maximum value of *M*_n_ 27.2 kDa and *M*_w_ 201.6 kDa. Note that this data was obtained on the crude reaction mixture with no further work‐up. Pleasingly, this further indicates that a condensation polymerization mechanism is occurring. A similar trend is seen when polydispersity index is monitored over reaction time. The relatively large values of *Đ* reached by the end of the reaction suggested some small oligomers remain in the solution as the reaction proceeds (these are normally removed in our work‐up procedure).

Finally, as proof of concept, we subjected samples with very low *M*_n_ to vacuum at 80 °C in the presence of 1 mol% **1** (**2 b**, **2 d**, **2 f**, **2 g**, **2 h**) and 4 mol% **1** (**2 n**). If these samples are pre‐polymers, this second set of more forcing condensation conditions will allow the short chains to react and thus generate higher *M*_n_ polymer.^[19]^ This is indeed the case (Table 3). Using crude pre‐polymer mixtures (to demonstrate broad applicability, without the need for isolation and removal of very low *M*_n_ species), we subjected **2 b** directly to vacuum condensation conditions. Pleasingly, this results in a greater than five times increase in *M*_n_. As expected with condensation polymerization, this also leads to broadening of the *Ð* value to 2.5 (compare Table 3, Entries 1 and 2). We observe similar trends in polymer data when employing isolated material. For example, **2 d** shows an increase in *M*_n_ from 1.3 to 8.4 kDa, there is a concomitant broadening of *Ð* to 4.1 and *T*_g_ increases from −52.8 to −16.4 °C (compare Table 3, Entries 3 and 4). **2 f** transforms from a highly soluble species to an insoluble solid, there is a huge change in *T*_g_, from −70.6 °C to +196.2 °C and a marked drop in *T*_−5%,Ar_ and *T*_inf,Ar_ (compare Table 3, Entries 5 and 6). **2 g** displays *M*_n_ that is six times greater than that of the pre‐polymer sample, only a modest increase in *Ð* (from 1.5 to 2.2) and substantial changes in *T*_g_ and *T*_−5%,Ar_ (compare Table 3, Entries 7 and 8). Subjecting the crude samples of **2 h** and **2 n** to the vacuum polymerization conditions increases the molecular weight such that GPC could not be obtained (Table 3, Entries 10 and 12). **2 n** pre‐polymer shows no *T*_g_, but after vacuum polymerization a *T*_g_ of −3.8 °C is obtained, but more generally, vacuum polymerization leads to only modest changes in *T*_−5%,Ar_ and *T*_inf,Ar_ for **2 h** and **2 n**. It is also important to note that no reaction takes place in the absence of **1**, even with this more forcing set of reaction conditions.

**Table 3:** Change in pre‐polymer properties after exposure to vacuum at 80 °C in the presence of **1**.

*Entry*	*Product*	*M*_n_ [kDa]	*M*_w_ [kDa]	*Đ*	*T*_g_ [°C]^[a]^	*T*_–5%,Ar_ [°C]^[b]^	*T*_inf,Ar_ [°C]^[b]^
1	**2 b**	6.2	12.0	1.9	*None*	365.8	439.4
**2** ^[c],[d]^	**2 b +vac**	36.3	90.4	2.5	*None*	356.8	436.5
3	**2 d**	1.3	1.9	1.5	−52.8	384.1	427.3
4^[c]^	**2 d+vac**	8.4	34.5	4.1	−16.4	344.1	409.1
5	**2 f**	1.1	1.3	1.2	−70.6	332.7	436.8
6^[c],[e]^	**2 f+vac**	*Insol*.	*Insol*.	*Insol*.	196.2	188.7	255.2
7	**2 g**	2.4	3.9	1.6	−42.8	310.2	373.6
8^[c]^	**2 g +vac**	15.0	32.9	2.2	−13.6	251.7	357.3
9	**2 h**	1.5	2.0	1.4	−5.5	245.5	300.1
10^[c],[d],[e]^	**2 h+vac**	*Insol*.	*Insol*.	*Insol*.	*None*	238.2	236.6
11	**2 n**	1.3	1.9	1.5	None	234.8	242.6
							357.3
12^[c],[d],[e]^	**2 n+vac**	*Insol*.	*Insol*.	*Insol*.	−3.8	233.7	242.6
							349.7

[a] Determined using DSC (−50 to +270 °C or −100 to +100 °C for oily samples, see Supporting Information) [b] Determined using TGA (+25 to +600 °C). [c] Vacuum conditions: **1** (1 mol%) as a solution in toluene and pre‐polymer (1 equiv.) added to a sealed 60 cm^3^ J‐Young Schlenk vessel. The corresponding solution was stirred at 80 °C for 1 h. Volatiles removed under vacuum and the residue stirred at 80 °C under a dynamic vacuum for a further 18 h. [d] Crude reaction mixture subjected to vacuum conditions. [e] *Insol*.=insoluble sample.

## Conclusion

In summary, we have presented an operationally simple iron‐catalyzed polymerization reaction of silane monomers with a range of diols to form different poly(silylether) products. The optimized reaction conditions were applicable to 1° and 2° silane monomer substrates reacting with benzylic and aliphatic diol and polyol substrates. We have characterized fourteen polymers using a range of techniques including IR spectroscopy, DSC and TGA. Furthermore, we have confirmed that the reaction proceeds via a condensation polymerization‐type mechanism through in situ reaction monitoring techniques and have applied this knowledge to convert very low *M*_n_ pre‐polymer samples to higher *M*_n_ species. Work is ongoing to develop our polymerization chemistry with **1** further, as well as investigate the kinetics basis of these catalytic polymerization reactions to form poly(silylether)s.

## Experimental Section

General method for dehydrocoupling polymerization: to a 60 cm^3^ J‐Young Schlenk vessel, pre‐catalyst **1** (0.004 mmol, 1 mol%) was added in 1 mL of benzene, under an inert atmosphere. Diol or polyol (0.4 mmol, 1 equiv.) and silane (0.8–1.2 mmol, 2–3 equiv.) were then added to the reaction vessel and the corresponding solution was stirred at 80 °C for 18 h. The volatiles were removed on a Schlenk‐line and the residue was washed with dry pentane. The pentane insoluble fractions were then dried and analyzed. ^1^H, ^13^C{^1^H} and ^29^Si{^1^H} NMR spectroscopies, GPC, MALDI‐TOF, DSC, TG‐MS and IR were used to analyse the THF soluble products. For products that were highly insoluble DSC, TG‐MS and IR were obtained only. See Supporting Information for other methods (catalyst synthesis, vacuum polymerisation conditions, mechanistic study methods) and full polymer analysis data.

## Conflict of interest

The authors declare no conflict of interest.

1

## Supporting information

As a service to our authors and readers, this journal provides supporting information supplied by the authors. Such materials are peer reviewed and may be re‐organized for online delivery, but are not copy‐edited or typeset. Technical support issues arising from supporting information (other than missing files) should be addressed to the authors.

Supporting Information

## Data Availability

The data that support the findings of this study are available in the supplementary material of this article.
