# Supplementary material for: Dehydrocoupling Polymerization: Poly(silylether) Synthesis by Using an Iron β‐Diketiminate Catalyst
Source: Chemistry. 2022 Sep 8;28(62):e202201642. doi: 10.1002/chem.202201642 (PMC9826106; doi:10.1002/chem.202201642)
Supplement: Supplementary file 1 — Supporting Information [file CHEM-28-0-s001.pdf]

Chemistry–A European Journal

Supporting Information

Dehydrocoupling Polymerization: Poly(silylether) Synthesis by Using an Iron β-Diketiminate Catalyst

Mirela A. Farcaş-Johnson, Sara H. Kyne,* and Ruth L. Webster*

Contents

General considerations 2

Method for the preparation of pre-catalyst **1** 2

Method optimization for the catalytic dehydrocoupling reactions 3

General method for catalytic dehydrocoupling polymerizations 3

Poly(silylether) syntheses 4

Polymerizations under vacuum conditions 9

Blank vacuum polymerization reaction 10

Swelling study of **2k** 10

Polymer preparation 10

Swelling study 10

Reaction monitoring of *in situ* gas evolution 11

Chain-grown reaction monitoring by GPC 12

NMR Spectrum of **1** 14

NMR spectra of products 14

Gel Permeation Chromatography (GPC) data 29

Optimization procedure 29

Polymerizations under vacuum conditions 85

GPC monitored chain growth experiment 121

Matrix-assisted Laser Desorption/Ionization Time-of-Flight Mass Spectrometry (MALDI-TOF) data 137

Fourier-transform infrared (FTIR) Spectroscopy Data of **2a-2n** 142

Differential Scanning Calorimetry (DSC) data of **2a-2n** 149

Differential Scanning Calorimetry (DSC) data of vacuum polymerizations 153

Thermogravimetric Analysis (TGA) Data of **2a-2n** 156

Thermogravimetric Analysis (TGA) data of vacuum polymerization reactions 163

References 166

General considerations

Unless otherwise stated, all solvents and reagents were used as purchased and all reactions were performed under an inert atmosphere using standard Schlenk and glovebox techniques. Heated and anhydrous reactions were undertaken in Teflon-sealed 60 mL J-Young Schlenk vessels.

Laboratory grade benzene, benzene-d_6, pentane and THF were purchased from Fisher Scientific or Sigma Aldrich and dried over sodium/benzophenone and distilled prior use. Reagents were purchased from Merk, Acros or Fluorochem and kept in the glovebox. TMP·BH_3 (2,2,6,6-tetramethylpiperidine borane) was synthesised according to literature procedure.[1] Room temperature (RT) refers to 298 K. Temperatures of 0 °C and −78 °C were obtained using ice/water and CO_2(s)/acetone baths respectively.

^{1}H, ^{13}C{^{1}H} and ^{29}Si{^{1}H} NMR spectra were recorded on Bruker Avance or Agilent 500-400 MHz NMR spectrometers. In C_6D_6, ^{1}H and ^{13}C{^{1}H} NMR chemical shifts are reported relative to C_6H_6 at 7.16 ppm and 128.06 ppm, respectively. Coupling constants (J) are reported in Hertz (Hz). Multiplicities are indicated by: br s (broad singlet), s (singlet), d (doublet), t (triplet), q (quartet) and m (multiplet).

Fourier Transform Infrared Spectroscopy (IR) was recorded on Perkin Elmer Spectrum 100 FT-IR Spectrometer. Size Exclusion Chromatography (GPC) analysis were performed using a SEC instrument Agilent 1260 GPC/SEC MDS. The mobile phase was GPC-grade tetrahydrofuran (THF 1mL/min) and the calibration was done using a polystyrene standard. Differential Scanning Calorimetry (DSC) was performed using a TA instrument DSC Q20, controlled by the program Q series. Thermogravimetric analysis (TGA) was performed on a Setsys Evolution TGA 16/18 by Setaram instrument using a 10K/min temperature program up to 600 °C and under an atmosphere of argon. Matrix-assisted laser desorption/ionization time-of-flight mass spectroscopy (MALDI-TOF) was performed on using a Bruker AutoFlex Matrix-Assisted Laser Desorption Ionisation (MALDI) mass spectrometer. The matrix used for analysis was dithranol and the counterion was a sodium trifluoroacetate in a 10:2:1 sample:matrix:cation ratio all dissolved in THF.

Method for the preparation of pre-catalyst 1

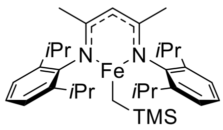

*n*Butyl lithium (solution in hexane, 1.67 mL, 4.18 mmol) was added to a Schlenk flask containing a stirred solution of β-diketimine (1.75 g, 4.18 mmol) in THF (20 mL) at –78 °C. This was then allowed to warm to room temperature and the resulting pale-yellow solution was stirred for 30 mins. Next, solid $FeCl_2(THF)_{1.5}$ (982 mg, 4.28 mmol) was added to the solution. The resulting yellow solution was stirred for 45 min. Solid $LiCH_2SiMe_3$ (394 mg, 4.18 mmol) was then added, and a very dark orange solution was obtained. After 30 min the solution turned dark red. After another 15 mins stirring the solvent was removed *in vacuo*. Any residual THF was then removed by stirring the residue with pentane (3 × 25 mL) and subsequent evaporation of all volatiles. Next, the residue was extracted with pentane *via* filtration through a pad of celite. The yellow/brown extract was concentrated to approximately 10 mL and cooled to –20 °C to afford **1** as olive yellow crystals. Analytical data were in accordance with those reported in literature (See Figure S6 for ^{1}H NMR spectrum).[2]

Method optimization for the catalytic dehydrocoupling reactions

Table S1: Table of optimization procedure method.

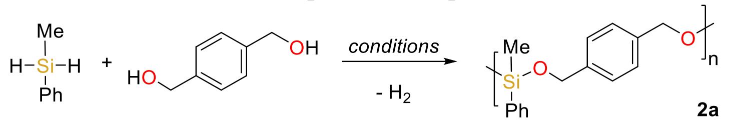

Entry	1,4-benzenedimethanol [equiv.]	$MePhSiH_2$ [equiv.]	**1** [mol%]	T [°C]	**2a** M_n [Da]	**GPC data Figure**
1[a]	3	1.00	10	80	*Insoluble*	NA
2	1	1.00	1	80	1 440	**S31**
3	1	1.25	0.5	RT	NR[b]	NA
4	1	1.75	10	70	5 421	**S32**
5	1	1.75	10	80	12 321	**S33**
6	1	2.00	1	80	21 546	**S35**
7	1	2.00	5	80	24 657	**S34**

[a] Phenylsilane employed. [b] No reaction detected by ^1H NMR spectroscopy.

Under an atmosphere of argon, to a sealed 60 cm^3 J-Young Schlenk vessel, pre-catalyst **1** (0.04 - 0.004 mmol, 10 - 1 mol%) was added in 1 mL of benzene. Diol (0.4 mmol, 1 equiv.) and silane (Entry 1 used phenylsilane, Entries 2 – 6 used methylphenylsilane) (0.8-1.2 mmol, 2-3 equiv.) were then added to the reaction vessel and the corresponding solution was stirred at RT - 80 °C for 18 h. The volatiles were removed on the Schlenk-line and the residue was washed with dry pentane. Where possible the product was dissolved in THF and analysed by GPC.

General method for catalytic dehydrocoupling polymerizations

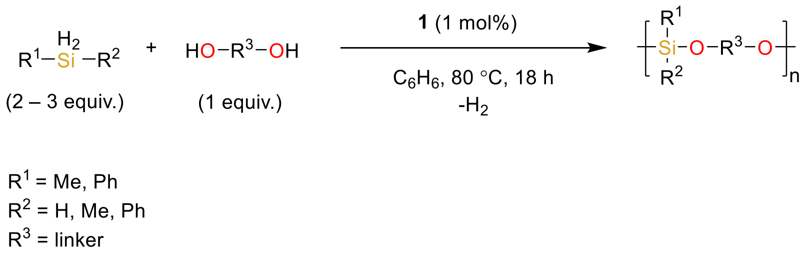

In the glovebox, to a 60 cm^3 J-Young Schlenk vessel, pre-catalyst **1** (0.004 mmol, 1 mol%) was added in 1 mL of benzene. Diol or polyol (0.4 mmol, 1 equiv.) and silane (0.8-1.2 mmol, 2-3 equiv.) were then added to the reaction vessel and the corresponding solution was stirred at 80 °C for 18 h. The volatiles were removed on the Schlenk-line and the residue was washed with dry pentane. The pentane insoluble fractions were then dried and analyzed.

^1H, $^{13}C\{^1H\}$ and $^{29}Si\{^1H\}$ NMR spectroscopies, GPC, MALDI-TOF, DSC, TG-MS and IR were used to analyse the THF soluble products. For products that were highly insoluble DSC, TG-MS and IR were obtained only.

Poly(silylether) syntheses

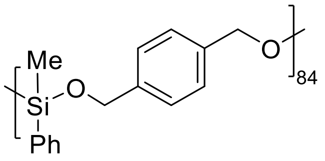

2a

Product **2a** was synthesized according to the general method by reacting 1,4-benzenedimethanol (55.3 mg, 0.4 mmol, 1 equiv.) with methylphenylsilane (109.9 µL, 0.8 mmol, 2 equiv.) in the presence of **1** (2.3 mg, 0.004 mmol, 1 mol%) and the reaction was heated to 80 °C for 18 h. The product was recovered as a brown sticky oil (76.6 mg). **^{1}H NMR** (500 MHz, C_6D_6): δ 7.72 (br s, 2H, Ar–*H*), 7.25 (br s, 3H, Ar–*H*), 7.10 (br s, 4H, Ar–*H*), 4.70 (br s, 4H, OCH_2Ph), 0.32 (br s, 3H, $SiCH_3$) ppm; **$^{13}C\{^1H\}$ NMR** (500 MHz, C_6D_6): δ 139.99, 134.63, 130.54, 128.59, 128.33, 126.96, 64.93, -3.75 ppm; **$^{29}Si\{^1H\}$ NMR** (500 MHz, C_6D_6): δ -16.27 ppm; **GPC**: M_n 21 546 Da, M_w 70 584 Da, *Đ* 3.3; **MALDI-TOF** (see Figure S63); **DSC**: T_g -15.5 °C; **TGA**: $T_{-5\%,Ar}$ 368.8 °C, $T_{inf,Ar}$ 442.3 °C; **FTIR** ν_{max}: 2868, 1429, 1374, 1258, 1214, 1120, 1057 (Si–O)[4], 1018, 998, 945, 860, 804, 778, 739, 716, 698, 671, 643, 636, 621, 601, 591, 582, 571, 563 cm^{-1}.

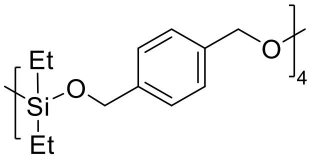

2b

Product **2b** was synthesized according to the general method by reacting 1,4-benzenedimethanol (55.3 mg, 0.4 mmol, 1 equiv.) with diethylsilane (103.6 µL, 0.8 mmol, 2 equiv.) in the presence of **1** (2.3 mg, 0.004 mmol, 1 mol%) and the reaction was heated to 80 °C for 18 h. The product was recovered as a brown oil (92.4 mg). **^{1}H NMR** (500 MHz, C_6D_6): δ 7.34 (m, 3H, Ar–*H*), 4.72 (m, 4H, OCH_2Ph), 1.01 (m, 6H, $SiCH_2CH_3$), 0.66 (m, 4H, $SiCH_2CH_3$) ppm; **$^{13}C\{^1H\}$ NMR** (500 MHz, C_6D_6): δ 140.40, 140.33, 140.16, 128.35, 126.84, 126.77, 66.66, 64.64, 6.92, 6.86, 5.64, 4.57 ppm; **$^{29}Si\{^1H\}$ NMR** (500 MHz, C_6D_6): δ -3.68 ppm; **GPC**: M_n 897 Da, M_w 1 190 Da, *Đ* 1.3; **MALDI-TOF** (see Figure S64); **DSC**: T_g none; **TGA**: $T_{-5\%,Ar}$ 365.8 °C, $T_{inf,Ar}$ 439.4 °C; **FTIR** ν_{max}: 2956, 2912, 2876, 2773, 2100, 1621, 1551, 1515, 1459, 1413, 1373, 1241, 1214, 1173, 1070 (Si–O)[4], 1007, 973, 822, 739, 639, 615, 569 cm^{-1}.

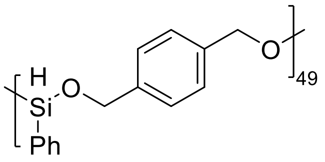

2c

Product **2c** was synthesized according to the general method by reacting 1,4-benzenedimethanol (55.3 mg, 0.4 mmol, 1 equiv.) with phenylsilane (148.1 µL, 1.2 mmol, 3 equiv.) in the presence of **1** (2.3 mg, 0.004 mmol, 1

mol%) and the reaction was heated to 80 °C for 18 h. The product was recovered as a brown oil (162.8 mg). **^{1}H NMR (500 MHz, C_6D_6):** δ 7.89 – 7.84 (m, 5H, Ar–*H*), 7.29 (s, 4H, Ar–*H*), 4.81 (s, 4H, OC*H*$_2$Ph) ppm; **^{13}C{^{1}H} NMR (500 MHz, C_6D_6):** δ 139.85, 136.06, 135.57, 135.50, 135.15, 133.10, 130.76, 130.68, 130.14, 128.59, 128.47, 128.42, 128.40, 128.35, 127.19, 127.06, 126.99, 126.26, 65.40, 65.19 ppm; **^{29}Si{^{1}H} NMR** (500 MHz, C_6D_6): δ -30.59 ppm; **GPC**: M_n 11 888 Da, M_w 21 852 Da, $Đ$ 1.8; **MALDI-TOF** (see Figure S65); **DSC**: T_g -41.8 °C; **TGA**: $T_{-5\%,Ar}$ 350.3 °C, $T_{inf,Ar}$ 405.1 °C; **FTIR** ν_{max}: 2911, 2865, 2140, 1904, 1883, 1591, 1429, 1373, 1215, 1125, 1116, 1068, 1052 (Si–O)[4], 998, 863, 835, 782, 764, 737, 718, 695, 679, 673, 620, 571, 562 cm^{-1}.

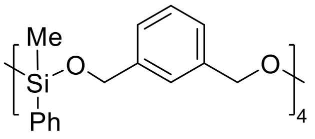

2d

Product **2d** was synthesized according to the general method by reacting 1,3-benzenedimethanol (55.3 mg, 0.4 mmol, 1 equiv.) with methylphenylsilane (109.9 µL, 0.8 mmol, 2 equiv.) in the presence of **1** (2.3 mg, 0.004 mmol, 1 mol%) and the reaction was heated to 80 °C for 18 h. The product was recovered as a grey solid (23.3 mg). **^{1}H NMR** (500 MHz, C_6D_6): δ 7.78, 7.58, 7.36, 7.19, 5.28, 4.75, 4.64, 0.39, 0.36 ppm (signals are broad and unassignable – see Figure S16); **^{13}C{^{1}H} NMR** (500 MHz, C_6D_6): δ 141.06, 140.88, 134.63, 134.32, 130.44, 125.78, 125.21, 66.51, 65.03, -2.51, -3.78 ppm; **^{29}Si{^{1}H} NMR** (500 MHz, C_6D_6): δ -1.80, -16.25 (terminal *Si*) ppm; **GPC**: M_n 1 256 Da, M_w 1 825 Da, $Đ$ 1.5; **DSC**: T_g -52.8 °C; **TGA**: $T_{-5\%,Ar}$ 384.1 °C, $T_{inf,Ar}$ 427.3 °C; **FTIR** ν_{max}: 3839, 3069, 2871, 2224, 2120, 1996, 1612, 1591, 1488, 1459, 1429, 1379, 1255, 1156, 1119, 1063 (Si–O)[4], 998, 860, 776, 739, 719, 698 cm^{-1}.

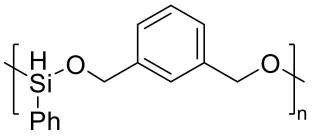

2e

Product **2e** was synthesized according to the general method by reacting 1,3-benzenedimethanol (55.3 mg, 0.4 mmol, 1 equiv.) with phenylsilane (148.1 µL, 1.2 mmol, 3 equiv.) in the presence of **1** (2.3 mg, 0.004 mmol, 1 mol%) and the reaction was heated to 80 °C for 18 h. The product was recovered as a brown gel (9.0 mg). **MALDI-TOF** (see Figure S66); **DSC**: T_g 1.35 °C; **TGA**: $T_{-5\%,Ar}$ 376.1 °C, $T_{inf,Ar}$ 360.8 °C, 507.6 °C; **FTIR** ν_{max}: 3051, 2927, 2871, 2164, 2035, 2011, 1963, 1612, 1592, 1552, 1489, 1460, 1448, 1430, 1380, 1261, 1157, 1127, 1097, 1055 (Si–O)[4], 998, 907, 830, 797, 771, 737, 696 cm^{-1}.

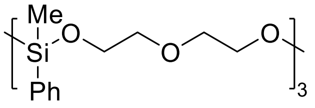

2f

Product **2f** was synthesized according to the general method by reacting diethylene glycol (38.0 µL, 0.4 mmol, 1 equiv.) with methylphenylsilane (109.9 µL, 0.8 mmol, 2 equiv.) in the presence of **1** (2.3 mg, 0.004 mmol, 1

mol%) and the reaction was heated to 80 °C for 18 h. The product was recovered as a brown oil (133.1 mg). **^{1}H NMR** (500 MHz, C_6D_6): δ 7.83 – 7.78 (m, 2H), 7.66 – 7.60 (m, 3H), 7.28 – 7.16 (m, 9H), 5.28 (s, 1H), 3.93 – 3.80 (m, 5H), 3.77 – 3.65 (m, 4H), 3.52 – 3.30 (m, 10H), 2.11 (s, 1H), 0.44 – 0.38 (m, 9H) ppm; **^{13}C{^{1}H} NMR** (500 MHz, C_6D_6): δ 136.56, 135.39, 135.24, 134.66, 134.30, 133.79, 130.28, 129.33, 128.59, 128.57, 128.35, 125.70, 73.05, 72.73, 72.70, 72.66, 72.63, 72.55, 64.24, 64.20, 63.80, 62.77, 62.73, 62.70, -0.54, -2.31, -3.81 ppm; **^{29}Si{^{1}H} NMR** (500 MHz, C_6D_6): δ -2.33, -17.32.ppm; **GPC**: M_n 885 Da, M_w 1 328 Da, $Đ$ 1.5; **MALDI-TOF** (see Figure S67); **DSC**: T_g -70.6 °C; **TGA**: $T_{-5\%,Ar}$ 332.7 °C, $T_{inf,Ar}$ 436.8 °C; **FTIR** ν_{max}: 2923, 2872, 2120, 1429, 1355, 1291, 1253, 1143, 1118, 1087 (Si–O)[4], 998, 959, 860, 797, 762, 737, 700, 657 cm^{-1}.

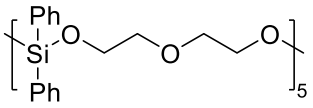

2g

Product **2g** was synthesized according to the general method by reacting diethylene glycol (38.0 µL, 0.4 mmol, 1 equiv.) with diphenylsilane (148.5 µL, 0.8 mmol, 2 equiv.) in the presence of **1** (2.3 mg, 0.004 mmol, 1 mol%) and the reaction was heated to 80 °C for 18 h. The product was recovered as a brown oil (86.5 mg). **^{1}H NMR (500 MHz, C_6D_6):** δ 7.91 – 7.82 (m, 3H, Ar–*H*), 7.70 (br s, 2H, Ar–*H*), 7.19 (s, 5H, Ar–*H*), 4.03 – 3.71 (m, 4H, O–C*H*$_2$), 3.57 – 3.38 (m, 4H, O–C*H*$_2$); **^{13}C{^{1}H} NMR** (500 MHz, C_6D_6): δ 136.06, 135.57, 135.50, 135.15, 134.79, 133.54, 130.54, 130.52, 130.14, 128.59, 128.33, 72.59, 72.52, 64.62, 63.03, 62.99 ppm; **^{29}Si{^{1}H} NMR** (500 MHz, C_6D_6): δ -31.68 ppm; **GPC**: M_n 1 588 Da, M_w 2 315 Da, $Đ$ 1.5; **MALDI-TOF** (see Figure S68); **DSC**: T_g -42.8 °C; **TGA**: $T_{-5\%,Ar}$ 310.2 °C, $T_{inf,Ar}$ °C; **FTIR** ν_{max}: 3070, 3049, 2929, 2873, 2132, 1981, 1896, 1591, 1486, 1456, 1429, 1379, 1356, 1328, 1291, 1252, 1115, 1082 (Si–O)[4], 997, 956, 840, 819, 735, 698 cm^{-1}.

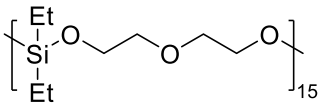

2h

Product **2h** was synthesized according to the general method by reacting diethylene glycol (38.0 µL, 0.4 mmol, 1 equiv.) with diethylsilane (103.6 µL, 0.8 mmol, 2 equiv.) in the presence of **1** (2.3 mg, 0.004 mmol, 1 mol%) and the reaction was heated to 80 °C for 18 h. The product was recovered as a brown oil (58.1 mg). **^{1}H NMR (500 MHz, C_6D_6):** δ 3.90 (t, J = 5.6 Hz, 4H, OC*H*$_2$CH$_2$O), 3.56 (t, J = 5.3 Hz, 4H, OCH$_2$C*H*$_2$O), 1.11 (q, J = 8.2 Hz, 6H, SiCH$_2$C*H*$_3$), 0.74 (t, J = 7.9 Hz, 4H, SiC*H*$_2$CH$_3$) ppm; **^{13}C{^{1}H} NMR** (500 MHz, C_6D_6): δ 73.05, 62.51, 6.86, 4.45 ppm; **^{29}Si{^{1}H} NMR** (500 MHz, C_6D_6): δ -4.64 ppm; **GPC**: M_n ~~14 432~~3 183 Da, M_w 6 788 ~~34 347~~ Da, $Đ$ 2.~~4~~1; **MALDI-TOF** (see Figure S69); **DSC**: T_g -5.45 °C; **TGA**: $T_{-5\%,Ar}$ 245.5 °C, $T_{inf,Ar}$ 300.08 °C; **FTIR** ν_{max}: 2955, 2916, 2876, 2116, 1990, 1459, 1414, 1380, 1355, 1292, 1242, 1142, 1089 (Si–O)[4], 1009, 947, 820, 737 cm^{-1}.

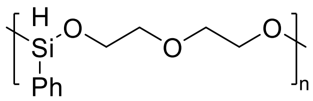

2i

Product **2i** was synthesized according to the general method by reacting diethylene glycol (38.0 µL, 0.4 mmol, 1 equiv.) with phenylsilane (148.1 µL, 0.8 mmol, 3 equiv.) in the presence of **1** (2.3 mg, 0.004 mmol, 1 mol%) and the reaction was heated to 80 °C for 18 h. The product was recovered as a highly insoluble grey solid (106.8 mg). **MALDI-TOF** (see Figure S70); **DSC**: T_g -47.8 °C; **TGA**: $T_{-5\%,Ar}$ 303.7 °C, $T_{inf,Ar}$ 413.5 °C; **FTIR** ν_{max}: 3072, 3049, 2935, 2876, 2163, 1593, 1458, 1430, 1392, 1329, 1287, 1260, 1125, 1072 (Si–O)[4], 998, 959, 907, 827, 787, 736, 698, 620, 595, 572 cm^{-1}.

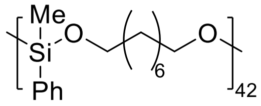

2j

Product **2j** was synthesized according to the general method by reacting 1,8-octanediol (58.5 mg, 0.4 mmol, 1 equiv.) with methylphenylsilane (109.9 µL, 0.8 mmol, 2 equiv.) in the presence of **1** (2.3 mg, 0.004 mmol, 1 mol%) and the reaction was heated to 80 °C for 18 h. The product was recovered as a brown oil (43.0 mg). **^{1}H NMR (500 MHz, C_6D_6):** δ 7.86 – 7.80 (m, 2H, Ar–*H*), 7.32 – 7.20 (m, 3H, Ar–*H*), 3.79 (t, 4H, C*H*$_2$), 1.62 (p, *J* = 2.0 Hz, 5H, C*H*), 1.44 – 1.31 (m, 6H, C*H*$_2$), 1.31 – 1.18 (m, 6H, C*H*$_2$), 0.43 (s, 3H, SiC*H*$_3$) ppm; **^{13}C{^{1}H} NMR (500 MHz, C_6D_6):** δ 135.74, 134.57, 130.29, 128.35, 63.13, 33.13, 29.89, 26.30, 20.80, -3.87 ppm; **^{29}Si{^{1}H} NMR (500 MHz, C_6D_6):** δ -18.65 ppm; **GPC**: M_n 10 579 Da, M_w 24 727 Da, $Đ$ 2.3; **MALDI-TOF** (see Figure S71); **DSC**: T_g none; **TGA**: $T_{-5\%,Ar}$ 421.6 °C, $T_{inf,Ar}$ 478.7 °C; **FTIR** ν_{max}: 3071, 3050, 2929, 2855, 2140, 1968, 1593, 1464, 1430, 1388, 1266, 1127, 1082 (Si–O)[4], 997, 920, 834, 736, 698, 619, 574 cm^{-1}.

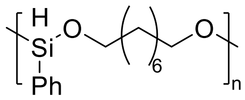

2k

Product **2k** was synthesized according to the general method by reacting 1,8-octanediol (58.5 mg, 0.4 mmol, 1 equiv.) with phenylsilane (148.1 µL, 1.2 mmol, 3 equiv.) in the presence of **1** (2.3 mg, 0.004 mmol, 1 mol%) and the reaction was heated to 80 °C for 18 h. The product was recovered as a brown oil (112.7 mg). **DSC**: T_g none; **TGA**: $T_{-5\%,Ar}$ 399.1 °C, $T_{inf,Ar}$ 460.0 °C; **FTIR** ν_{max}: 3070, 3050, 3006, 2929, 2855, 2743, 2127, 2051, 2037, 1974, 1622, 1593, 1551, 1463, 1429, 1388, 1268, 1127, 1083 (Si–O)[4], 997, 922, 841, 818, 767, 735, 715, 697 cm^{-1}.

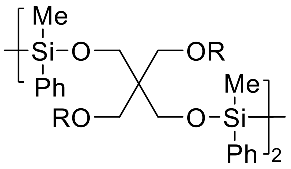

R = -H, -Si(MePh)-

2l

Product **2l** was synthesized according to the general method by reacting pentaerythritol (54.5 mg, 0.4 mmol, 1 equiv.) with methylphenylsilane (109.9 µL, 0.8 mmol, 2 equiv.) in the presence of **1** (2.3 mg, 0.004 mmol, 1 mol%) and the reaction was heated to 80 °C for 18 h. The product was recovered as a grey solid (22.9 mg). **^{1}H NMR (500 MHz, C_6D_6):** δ 7.69 (s, 4H Ar–*H*), 7.21 (s, 6H, Ar–*H*), 4.28 (d, *J* = 9.2 Hz, 2H, CH_2), 3.97 (d, *J* = 9.3 Hz, 2H, CH_2), 3.56 (d, *J* = 11.5 Hz, 2H, CH_2), 3.47 (d, *J* = 11.7 Hz, 2H, CH_2), 0.33 (s, 6H, SiCH_3) ppm; **^{13}C{^{1}H} NMR (500 MHz, C_6D_6):** δ 134.18, 130.70, (Ar–*C* obscured by solvent signal), 66.95, 66.76, 42.4, -2.81 ppm; **^{29}Si{^{1}H} NMR (500 MHz, C_6D_6):** δ -11.83 ppm; **GPC**: M_n 606 Da, M_w 1 148 Da, *Đ* 1.9; **DSC**: T_g 19.0 °C, T_c 158.3 °C; **TGA**: $T_{-5\%,Ar}$ 218.3 °C, $T_{inf,Ar}$ 294.8, 451.5 °C; **FTIR** ν_{max}: 3071, 3047, 2959, 2926, 2876, 2121, 2025, 1956, 1591, 1554, 1474, 1429, 1386, 1258, 1258, 1197, 1156, 1109, 1067, 1027 (Si–O)[4], 998, 819, 779, 740, 710, 698, 669, 659 cm^{-1}.

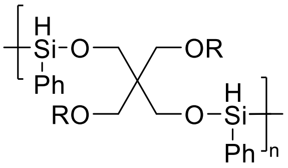

R = -H, -Si(HPh)-

2m

Product **2m** was synthesized according to the general method by reacting pentaerythritol (54.5 mg, 0.4 mmol, 1 equiv.) with phenylsilane (148.1 µL, 1.2 mmol, 3 equiv.) in the presence of **1** (2.3 mg, 0.004 mmol, 1 mol%) and the reaction was heated to 80 °C for 18 h. The product was recovered as a highly insoluble grey solid (49.2 mg). **DSC**: T_g none, T_m 121.4 °C, T_c 175.6 °C; **TGA**: $T_{-5\%,Ar}$ 227.8 °C, $T_{inf,Ar}$ 263.7, 453.4 °C; **FTIR** ν_{max}: 3315, 2954, 2884, 2163, 2035, 2009, 1979, 1593, 1452, 1430, 1406, 1382, 1276, 1161, 1128, 1039, 1012 (Si–O)[4], 993, 872, 847, 808, 767, 737, 697, 660 cm^{-1}.

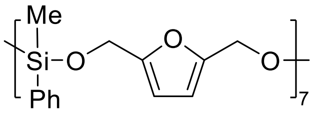

2n

Product **2n** was synthesized according to the general method by reacting 1,2-bis(hydroxymethyl)furan (BHMF) (51.3 mg, 0.4 mmol, 1 equiv.) with methylphenylsilane (109.9 µL, 0.8 mmol, 2 equiv.) in the presence of **1** (9.2 mg, 0.016 mmol, 4 mol%) and the reaction was heated to 80 °C for 18 h. The product was recovered as a brown oil (96.8 mg). **^{1}H NMR (500 MHz, C_6D_6):** δ 7.73 (br s, 2H, Ar–*H*), 7.20 (br 2, 3H, Ar–*H*), 6.04 (d, *J* = 4.6 Hz.

4H, OCH_2), 0.34 (s, 3H, SiCH_3) ppm; **$^{13}C\{^1H\}$ NMR (500 MHz, C_6D_6):** δ 154.01, 134.66, 130.44, 128.35, 108.92, 67.83, 57.86, 25.83, -3.75 ppm; **GPC**: M_n 1 775 Da, M_w 2 948 Da, *Đ* 1.7; **MALDI-TOF** (see Figure S72); **DSC**: T_g none; **TGA**: $T_{-5\%,Ar}$ 234.8 °C, $T_{inf,Ar}$ 242.6, 357.3 °C; **FTIR** ν_{max}: 2869, 2125, 1591, 1429, 1374, 1258, 1223, 1197, 1120, 1049 (Si–O)[4], 1018, 998, 925, 856, 781, 736, 699, 680, 623, 581, 573, 566, 558 cm^{-1}.

Polymerizations under vacuum conditions

2b

To a sealed 60 cm^3 J-Young Schlenk vessel, pre-catalyst **1** (2.3 mg, 0.004 mmol, 1 mol%) was added in 1 mL of benzene. 1,4-benzenedimethanol (55.3 mg, 0.4 mmol, 1 equiv.) and diethylsilane (103.6 µL, 0.8 mmol, 2 equiv.) were then added to the reaction vessel and the corresponding solution was stirred at 80 °C for 18 h. GPC analysis was carried out on an aliquot of the brown solution. The remaining solution was left to continue stirring at 80 °C for a further 18 h under a dynamic vacuum where all volatiles were removed in the process. A sticky brown solid (61.2 mg) was collected and analyzed by GPC, DSC and TGA. **GPC**: M_n 36 282 Da, M_w 90 362 Da, *Đ* 2.5; **DSC:** T_g none; **TGA:** $T_{-5\%,Ar}$ 356.8 °C, $T_{inf,Ar}$ 436.5 °C.

2d

To a sealed 60 cm^3 J-Young Schlenk vessel, pre-catalyst **1** (1.2 mg, 0.002 mmol, 1 mol%) was added in 0.6 mL of toluene. Polymer **2d** (51.2 mg, 0.2 mmol, 1 equiv.) was then added to the reaction vessel and the corresponding solution was stirred at 80 °C for 1 h. The volatiles were removed on the Schlenk-line and the residue was left to stir at 80 °C under a dynamic vacuum for a further 18 h. The pentane insoluble sticky brown solid (15.4 mg) was then dried and analyzed by GPC, DSC and TGA. **GPC:** M_n 8354 Da, M_w 34533 Da, *Đ* 4.1; **DSC:** T_g -16.4 °C; **TGA:** $T_{-5\%,Ar}$ 344.1 °C, $T_{inf,Ar}$ 409.1 °C.

2f

To a sealed 60 cm^3 J-Young Schlenk vessel, pre-catalyst **1** (2.3 mg, 0.004 mmol, 1 mol%) was added in 1 mL of benzene. Diethylene glycol (38.0 µL, 0.4 mmol, 1 equiv.) and methylphenylsilane (109.9 µL, 0.8 mmol, 2 equiv.) were then added to the reaction vessel and the corresponding solution was stirred at 80 °C for 18 h. The volatiles were removed on the Schlenk-line and GPC analysis was carried out on the brown oily residue. Next, a further amount of pre-catalyst **1** (0.004 mmol, 1 mol%) and toluene (0.6 mL) were added. The solution was left to stir at 80 °C for 1 h. The volatiles were then removed, and the residue was left to continue stirring at 80 °C for a further 18 h under a dynamic vacuum. An insoluble brown solid (13.9 mg) was collected and analyzed by DSC and TGA. **DSC:** T_g 196.2 °C; **TGA:** $T_{-5\%,Ar}$ 188.7 °C, $T_{inf,Ar}$ 225.2 °C.

2g

To a sealed 60 cm^3 J-Young Schlenk vessel, pre-catalyst **1** (2.3 mg, 0.004 mmol, 1 mol%) was added in 1 mL of benzene. Diethylene glycol (38.0 µL, 0.4 mmol, 1 equiv.) and diphenylsilane (148.5 µL, 0.8 mmol, 2 equiv.) were then added to the reaction vessel and the corresponding solution was stirred at 80 °C for 18 h. The volatiles were removed on the Schlenk-line and GPC analysis was carried out on the brown oily residue. Next, the residue was left to stir at 80 °C for 18 h under a dynamic vacuum. A brown oil (22.5 mg) was collected and analyzed by GPC, DSC and TGA. **GPC:** M_n 14 986 Da, M_w 32 925 Da, *Đ* 2.2; **DSC:** T_g **-**13.64 °C; **TGA:** $T_{-5\%,Ar}$ 251.7 °C, $T_{inf,Ar}$ 357.3 °C.

2h

To a sealed 60 cm^3 J-Young Schlenk vessel, pre-catalyst **1** (2.3 mg, 0.004 mmol, 1 mol%) was added in 1 mL of benzene. Diethylene glycol (38.0 µL, 0.4 mmol, 1 equiv.) and diethylsilane (103.6 µL, 0.8 mmol, 2 equiv.) were then added to the reaction vessel and the corresponding solution was stirred at 80 °C for 18 h. GPC analysis was carried out on an aliquot of the brown solution. The remaining solution was left to continue stirring at 80 °C for a further 18 h under a dynamic vacuum where all volatiles were removed in the process. An insoluble sticky brown solid (23.3 mg) was collected and analyzed by DSC and TGA. **DSC:** T_g none °C; **TGA:** $T_{-5\%,Ar}$ 238.2 °C, $T_{inf,Ar}$ 263.6 °C.

2n

To a sealed 60 cm^3 J-Young Schlenk vessel, pre-catalyst **1** (9.2 mg, 0.016 mmol, 4 mol%) was added in 1 mL of benzene. Diethylene glycol (38.0 µL, 0.4 mmol, 1 equiv.) and diethylsilane (103.6 µL, 0.8 mmol, 2 equiv.) were then added to the reaction vessel and the corresponding solution was stirred at 80 °C for 18 h. GPC analysis was carried out on an aliquot of the brown solution. The remaining solution was left to continue stirring at 80 °C for a further 18 h under a dynamic vacuum where all volatiles were removed in the process. An insoluble sticky brown solid (61.5 mg) was collected and analyzed by DSC and TGA. **DSC:** T_g -3.78 °C; **TGA:** $T_{-5\%,Ar}$ 233.7 °C, $T_{inf,Ar}$ 242.6, 349.7 °C.

Blank vacuum polymerization reaction

To a sealed 60 cm^3 J-Young Schlenk vessel, polymer **2d** (43.5 mg, 0.17 mmol) was added, followed by 0.6 mL toluene and the corresponding solution was stirred at 80 °C for 1 h. The volatiles were removed on the Schlenk-line and the residue was left to stir at 80 °C under a dynamic vacuum for a further 18 h. After this time the sticky brown solid analyzed by GPC. **GPC:** M_n 774 Da, M_w 989 Da, $Đ$ 1.3.

Swelling study of 2k

Polymer preparation

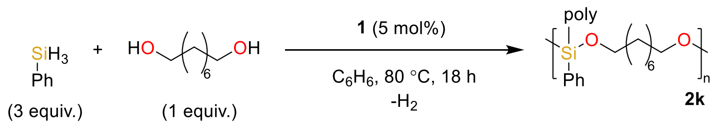

To a sealed 60 cm^3 J-Young Schlenk vessel, pre-catalyst **1** (11.2 mg, 0.002 mmol, 5 mol%) was added in 1 mL of benzene. 1,8-octanediol (58.5 mg, 0.4 mmol, 1 equiv.) and phenylsilane (148.1 µL, 1.2 mmol, 3 equiv.) were then added to the reaction vessel and the corresponding mixture was stirred at 80 °C for 18 h. The volatiles were removed on the Schlenk-line and the residue was washed with pentane. The pentane insoluble fraction was then collected as an insoluble orange gel (262.5 mg).

Swelling study

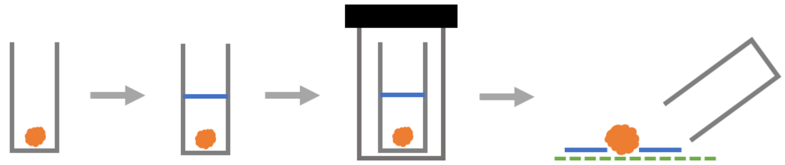

Figure S1: Schematic of the procedure described to study the swelling of polymer **2k**.

A dry sample of polymer **2k** (approx. 10 – 15 mg, (m_0)) was added to a vial which was filled with toluene until the sample was fully submerged. The vial was placed in a secondary container which was fully sealed. The sample was left submerged in the solvent for 2 days 8 h. After this time, the polymer sample was removed from the solvent and the surface of the soaked polymer sample was dried on a paper towel. The mass of the soaked sample (m_1) was then recoded and compared to the dry mass. This procedure was performed in triplicate.

Table S2: Polymer masses of **2k** polymer samples recorded during swelling study.

Entry	Dry mass (m_0) [mg]	Wet mass (m_1) [mg]	m_1/m_0
1	13.9	36.9	2.65
2	10.8	22.4	2.07
3	15.3	29.8	1.95

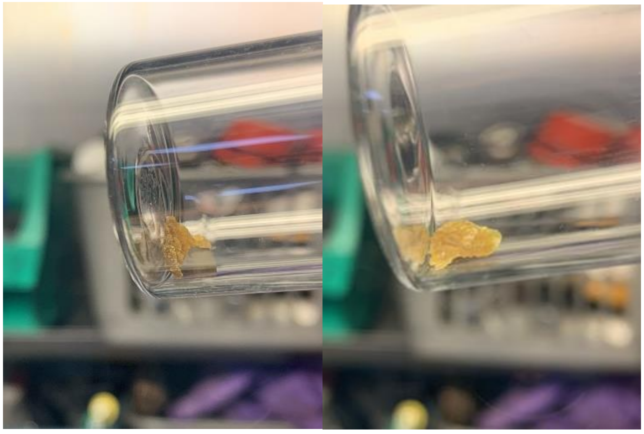

Figure S2: Dry sample of **2k** (left). Swollen sample of **2k** after 2 days 8 h in toluene (right).

Reaction monitoring of *in situ* gas evolution

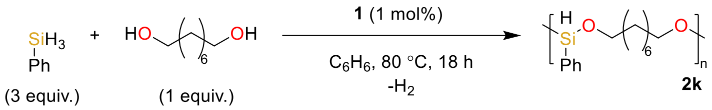

A Man on The Moon series 103 kit to study the gas evolution *in situ* by monitoring pressure increase. The study was carried out on the dehydrocoupling polymerization reaction between 1,8-octanediol and phenylsilane to synthesise **2k** which released H_2 gas.

In a 12 cm^3 airtight 2-neck round-bottom flask designed to fit the Man on The Moon apparatus, **1** (2.3 mg, 0.004 mmol, 1 mol%) and 1,8-octanediol (58.5 mg, 0.4 mmol, 1 equiv.) were added inside the glovebox. A solution of phenylsilane (148.1 µL, 1.2 mmol, 3 equiv.) in benzene (1 mL) was prepared in a separate vessel. The round-bottom flask containing the solids was connected to the Man on The Moon pressure increase sensor whilst maintaining an inert atmosphere of N_2 and this was heated to 80 °C to allow for pressure and temperature equilibration. The gas pressure inside the vessel at 80 °C was noted at this point. The solution of phenylsilane in

benzene was then added through a suba-seal whilst maintaining a constant flow of N_2 to the system. Monitoring of gas evolution began as soon as this addition occurred. The solution was left to stir at 80 °C for 18 h. The pressure inside the closed vessel was registered every minute for 23 h.

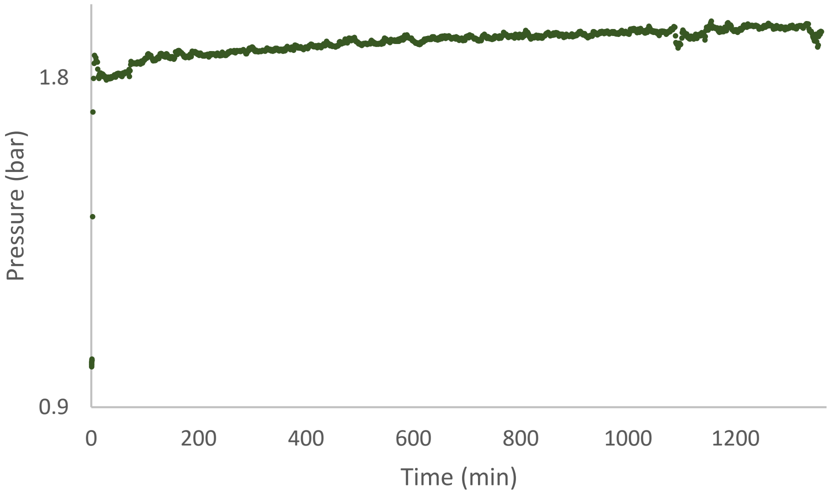

Figure S3: Plot of gas evolution versus time for the dehydrocoupling reaction of 1,8-octanediol and phenylsilane catalyzed by **1**.

Chain-grown reaction monitoring by GPC

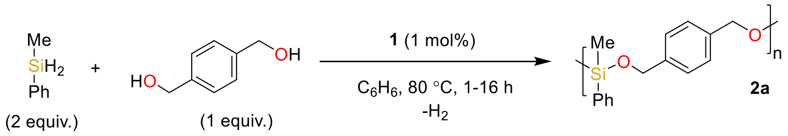

Study to monitor chain length growth over time was done on dehydrocoupling polymerisation reaction between 1,4-benzenedimethanol and methylphenylsilane.

In a 60 cm^3 J-Young schlenk, **1** (2.3 mg, 0.004 mmol, 1 mol%), 1,4-benzenedimethanol (55.3 mg, 0.4 mmol, 1 equiv.) and methylphenylsilane (109.9 µL, 0.80 mmol, 2 equiv.) were dissolved in benzene (1 mL). This was repeated three more times. The four vessels containing reaction mixtures were left to stir at 80 °C for 1 - 16 h with a stirring speed of 250 rpm. The reactions were stopped by removing the volatiles on a Schlenk line under an atmosphere of N_2 and recording the GPS data of the crude mixture dissolved in GPC grade THF. This was repeated at T = 1, 4, 8 and 16 h. The values of M_n, M_w and $Đ$ for each sample was recorded and plotted *vs.* their respective timepoints.

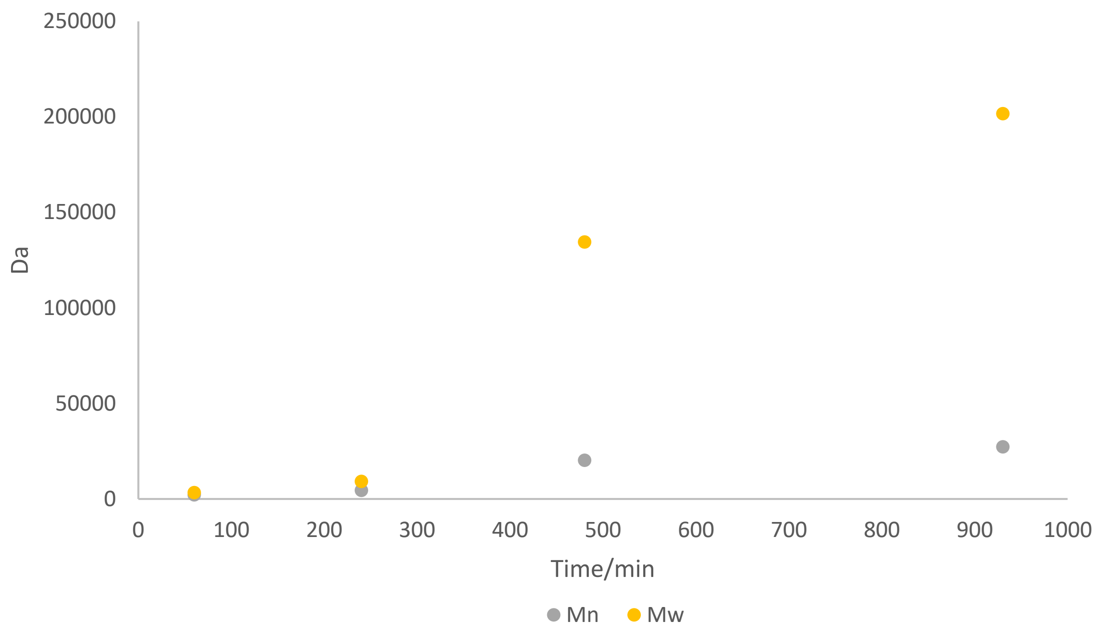

Figure S4: Plot of average molecular weight by number (M_n) and average molecular weight by weight (M_w) versus time for the dehydrocoupling reaction of 1,4-benzenedimethanol and methylphenylsilane catalyzed by **1**. The samples were taken of the crude reaction mixture.

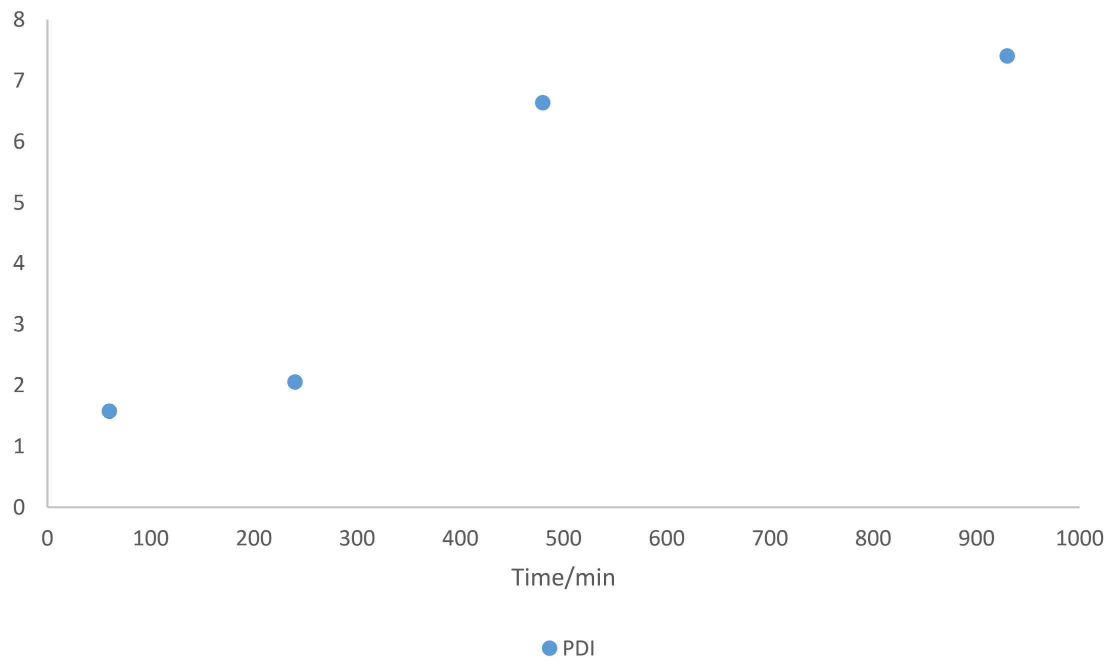

Figure S5: Plot of polymer dispersity index (*Đ*) versus time for the dehydrocoupling reaction of 1,4-benzenedimethanol and methylphenylsilane catalyzed by **1**. The samples were taken of the crude reaction mixture.

NMR Spectrum of 1

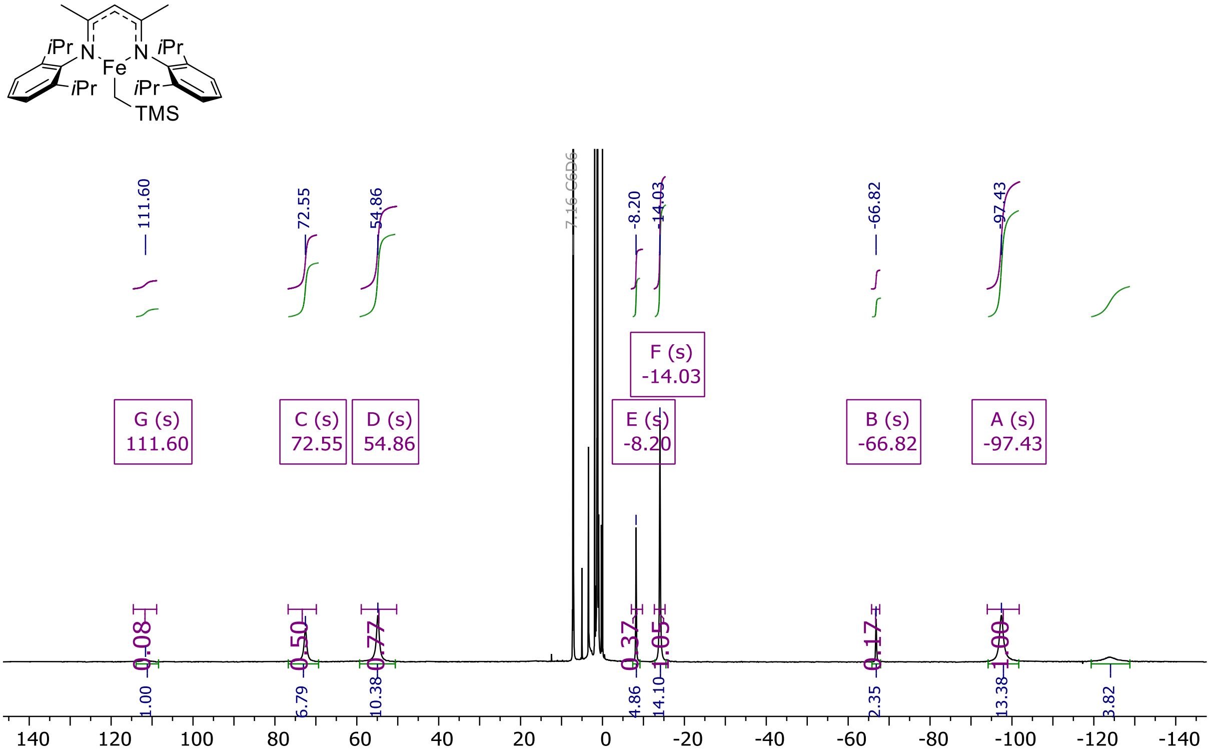

Figure S6: ^{1}H NMR spectrum of **1**.

NMR spectra of products

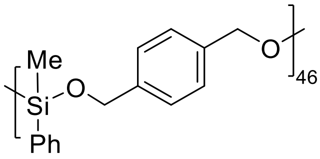

2a

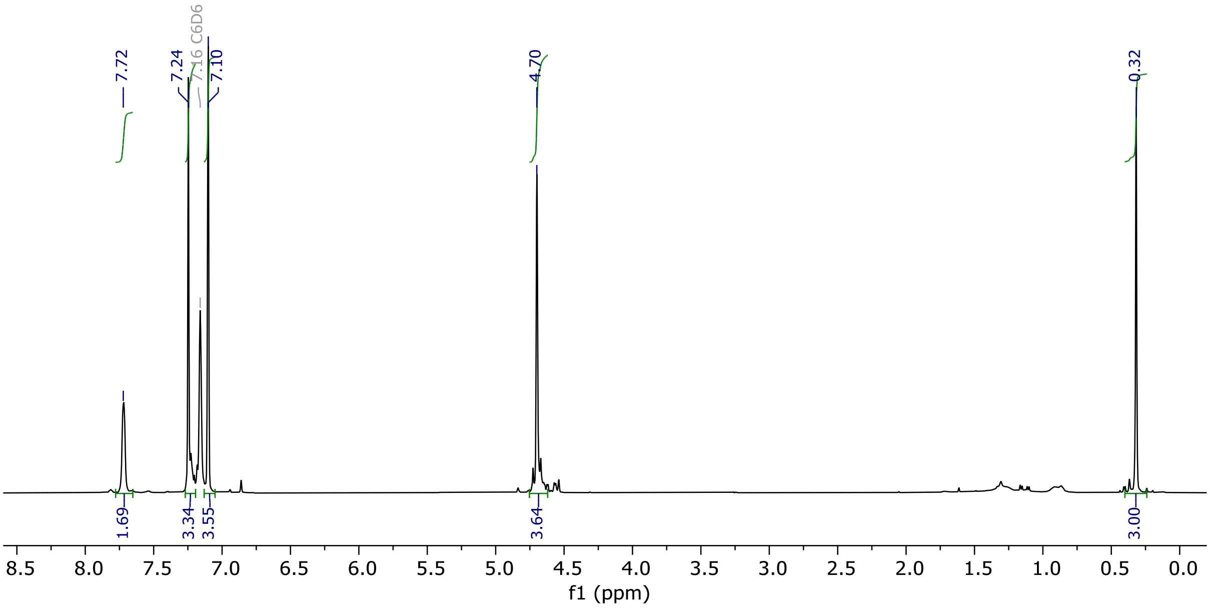

Figure S7: ^{1}H NMR spectrum of **2a**.

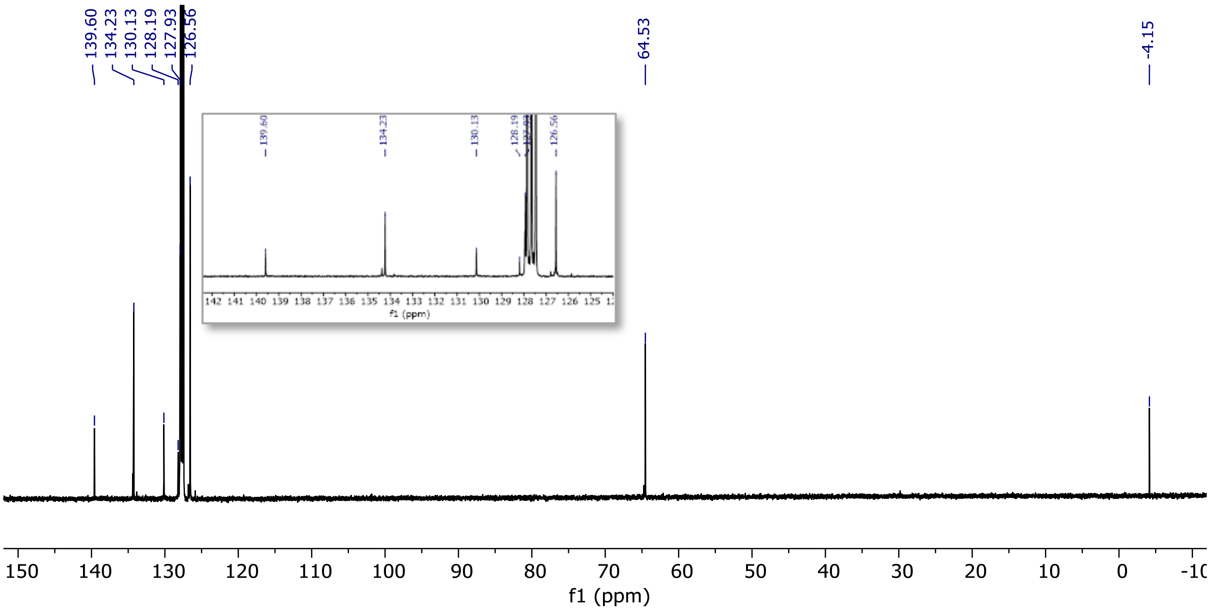

Figure S8: $^{13}C\{^{1}H\}$ NMR spectrum of **2a**.

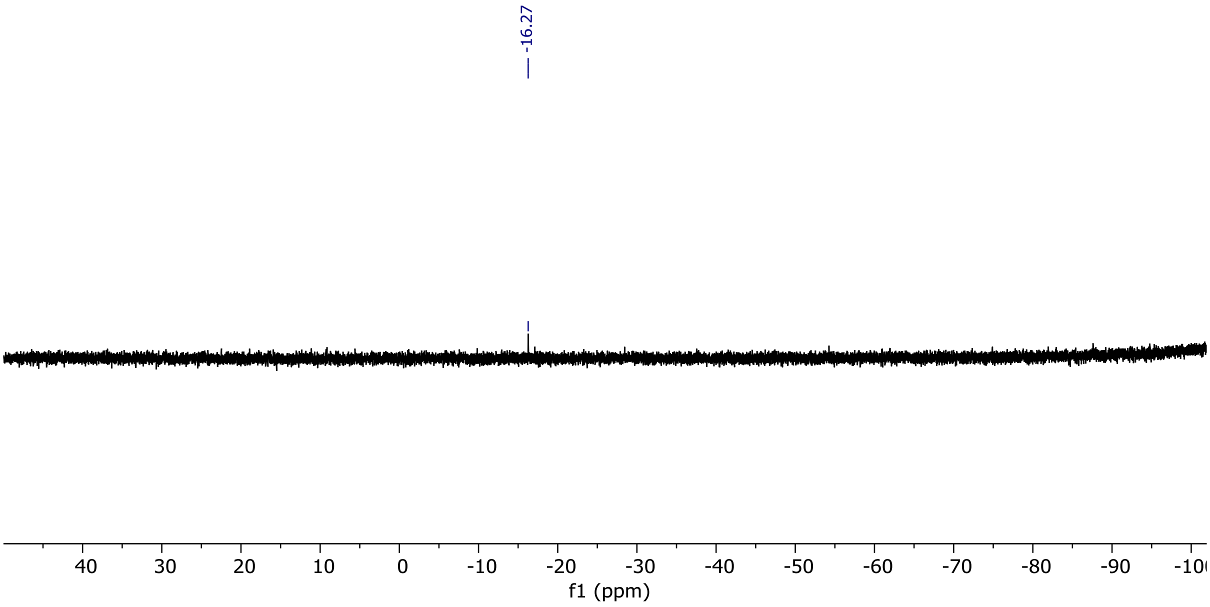

Figure S9: $^{29}Si\{^{1}H\}$ NMR spectrum of **2a**.

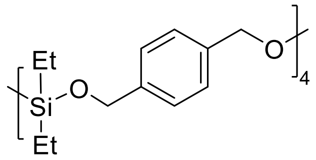

2b

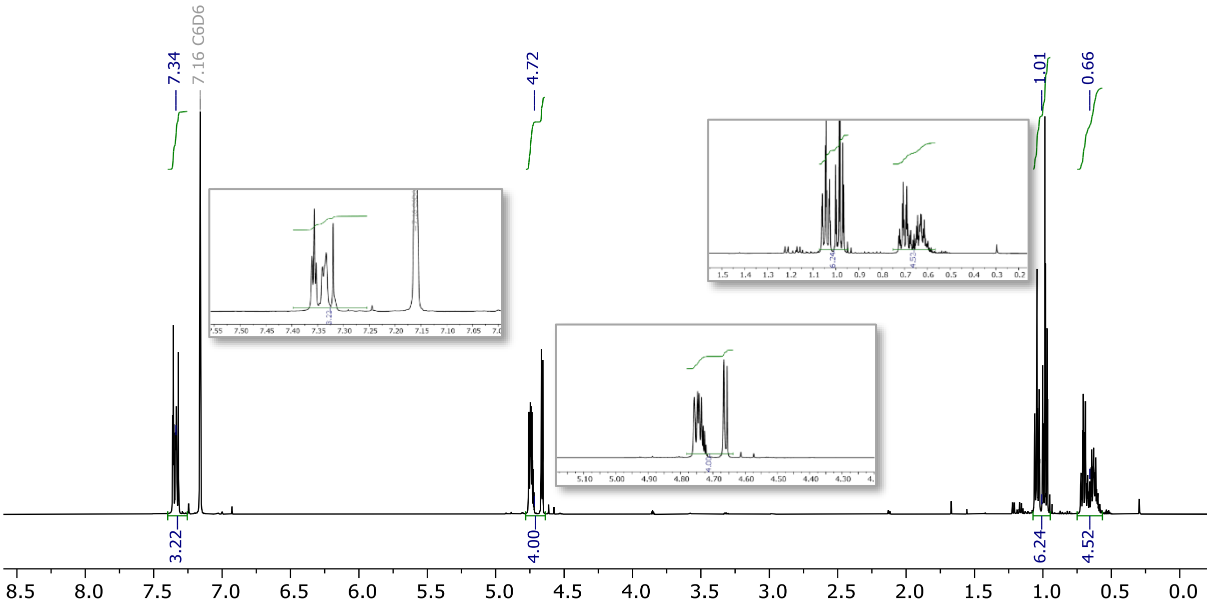

Figure S10: ^{1}H NMR spectrum of **2b**.

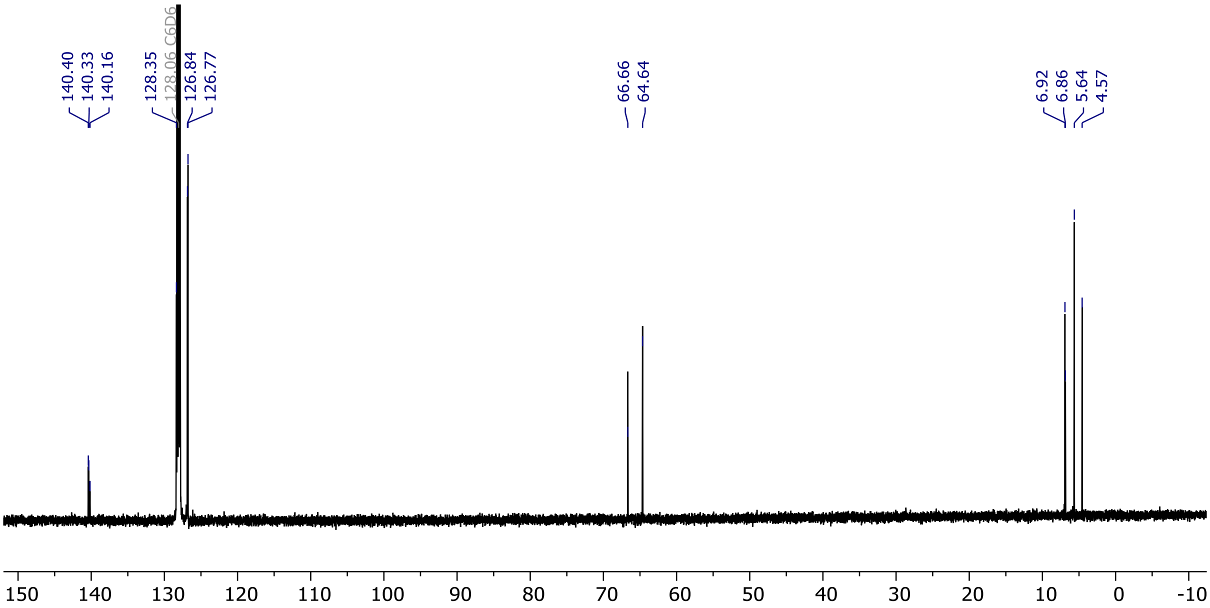

Figure S11: ^{13}C{^{1}H} NMR spectrum of **2b**.

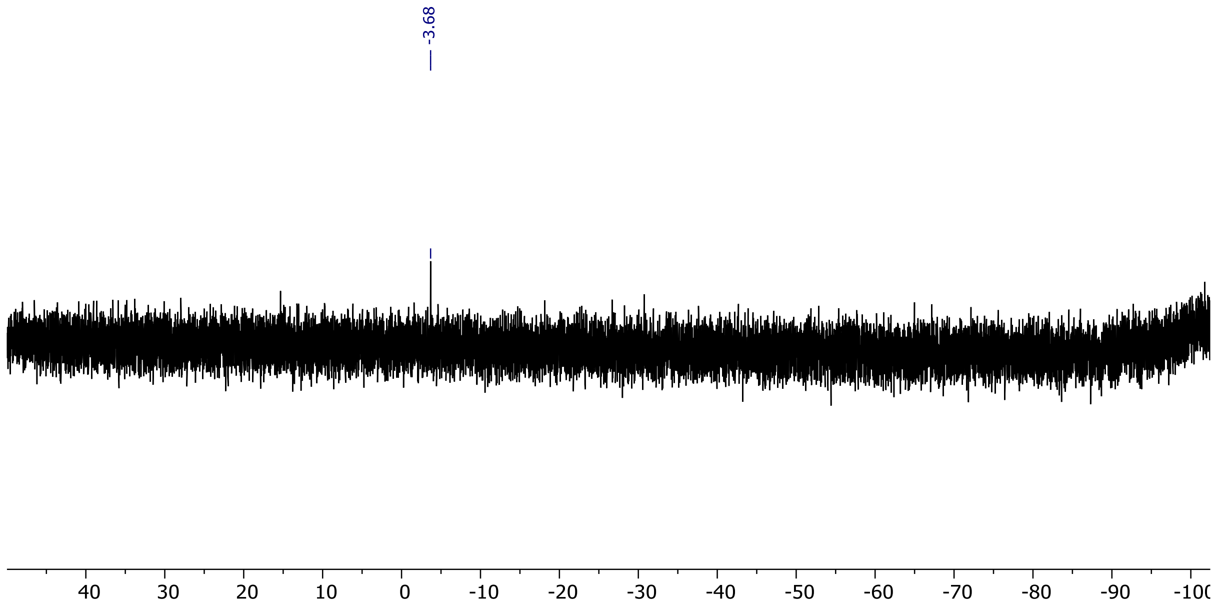

Figure S12: $^{29}Si\{^1H\}$ NMR spectrum of **2b**.

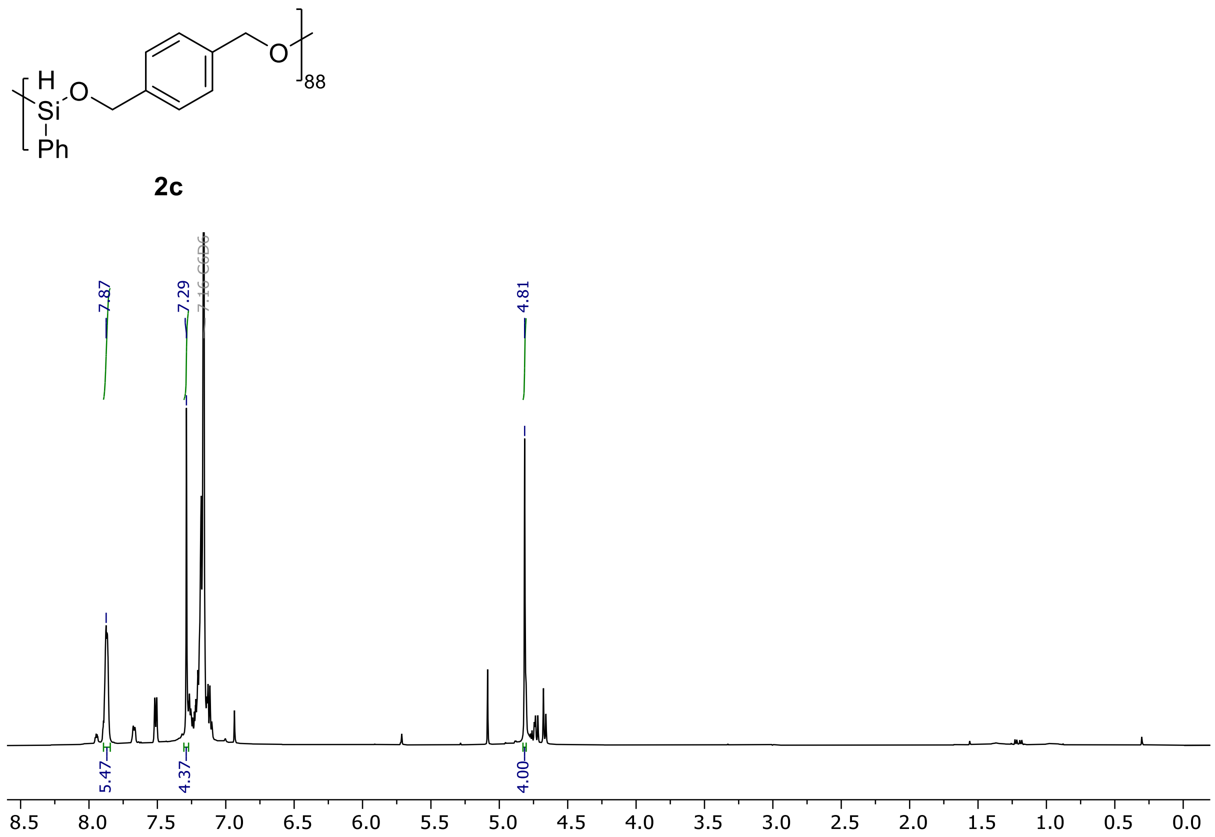

Figure S13: ^1H NMR spectrum of **2c**.

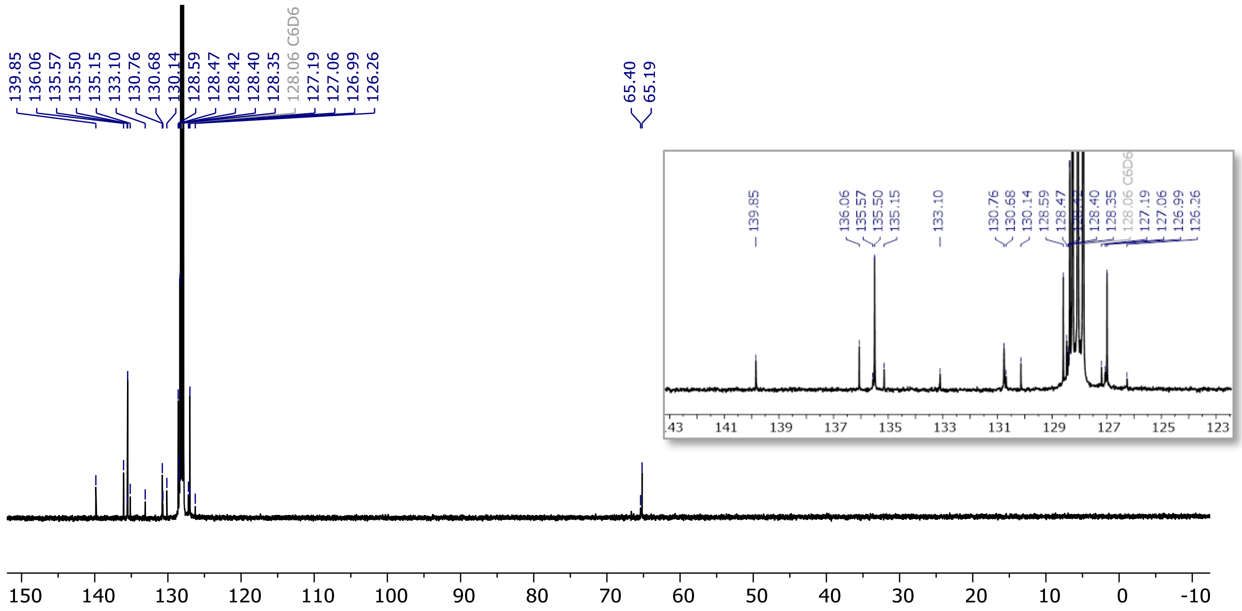

Figure S14: $^{13}C\{^{1}H\}$ NMR spectrum of **2c**.

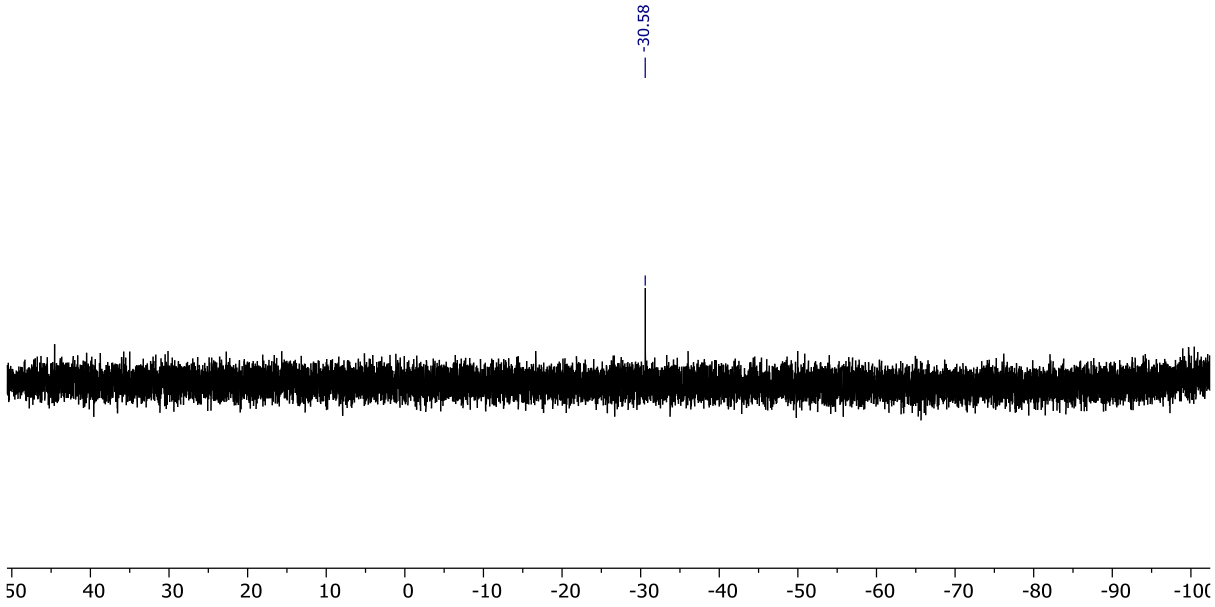

Figure S15: $^{29}Si\{^{1}H\}$ NMR spectrum of **2c**.

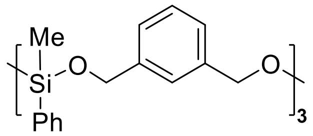

2d

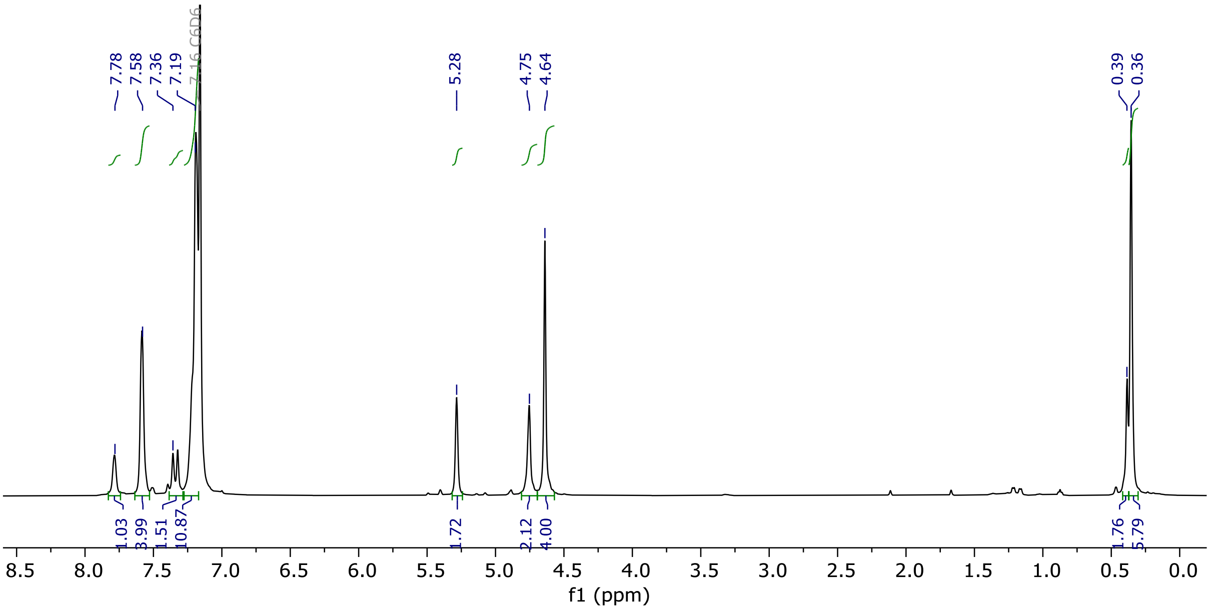

Figure S16: ^{1}H NMR spectrum of **2d**.

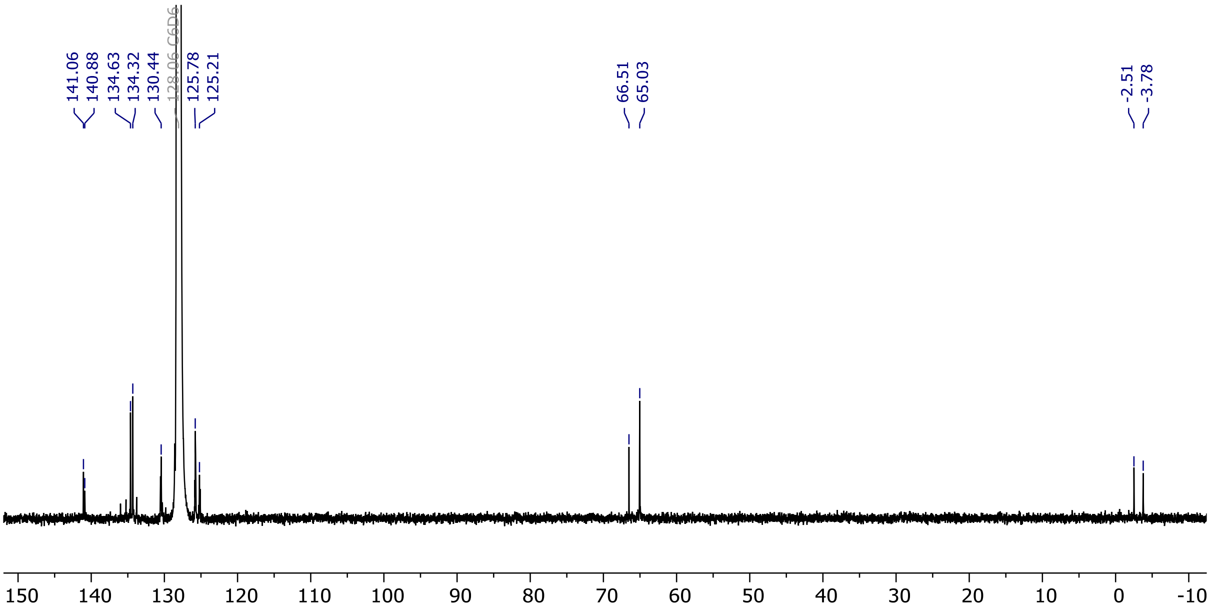

Figure S17: ^{13}C{^{1}H} NMR spectrum of **2d**.

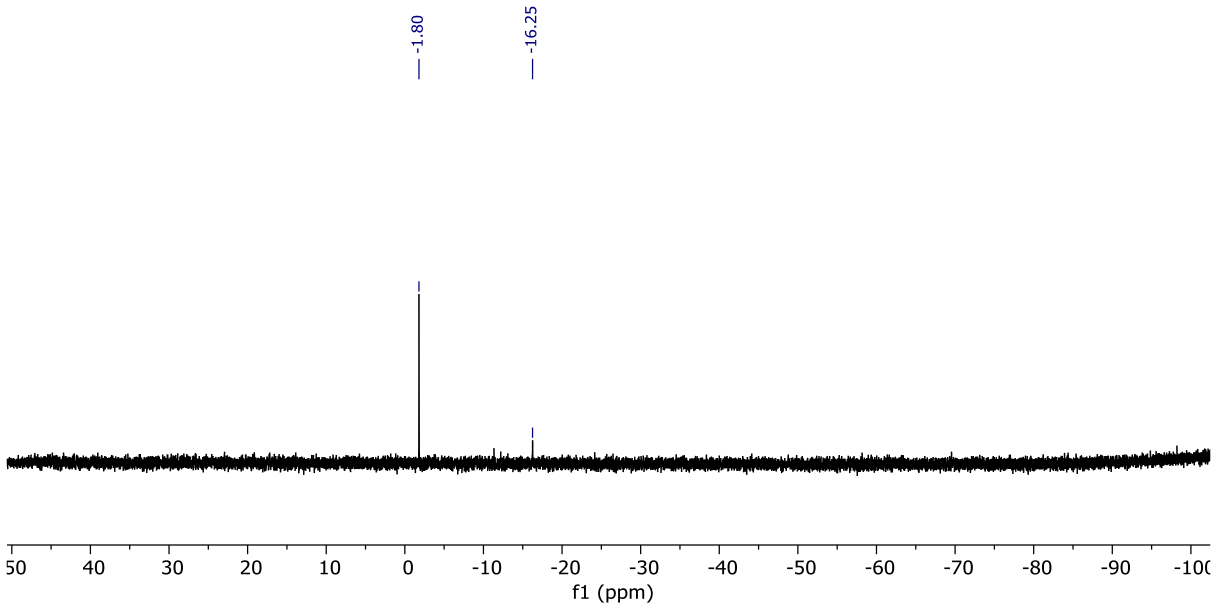

Figure S18: $^{29}Si\{^{1}H\}$ NMR spectrum of **2d**.

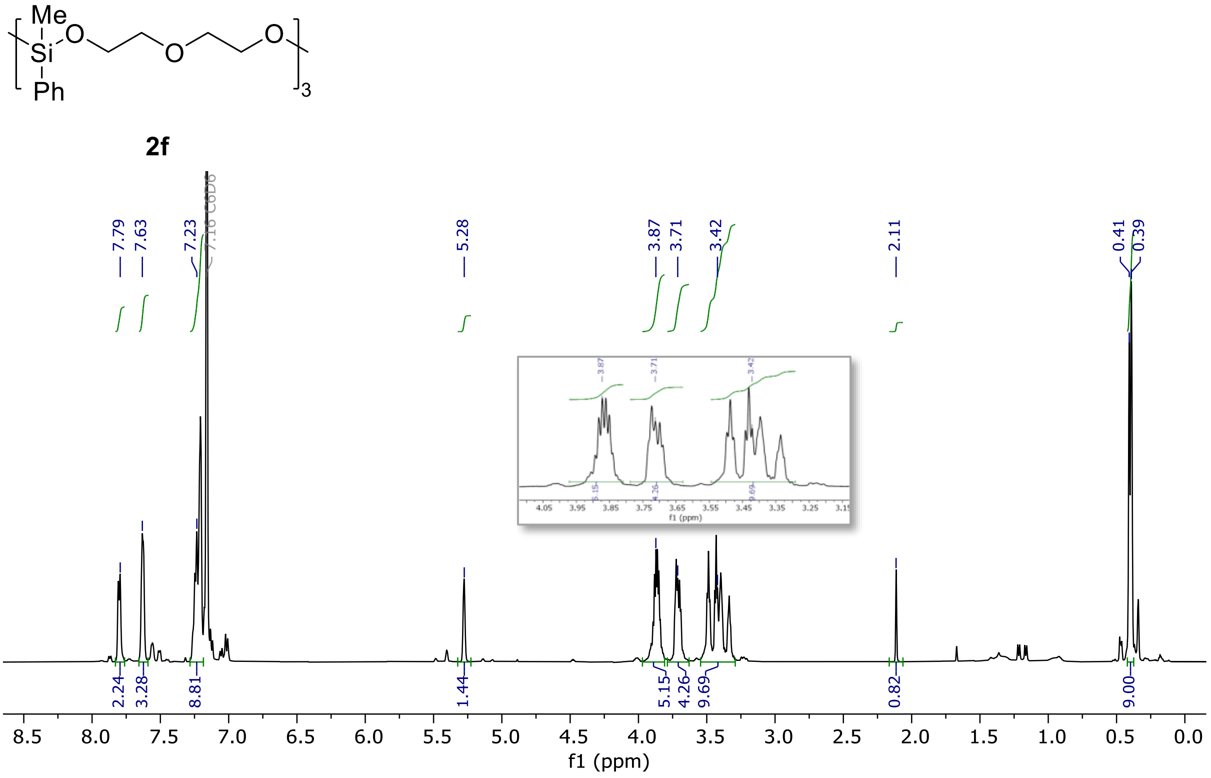

Figure S19: ^{1}H NMR spectrum of **2f**.

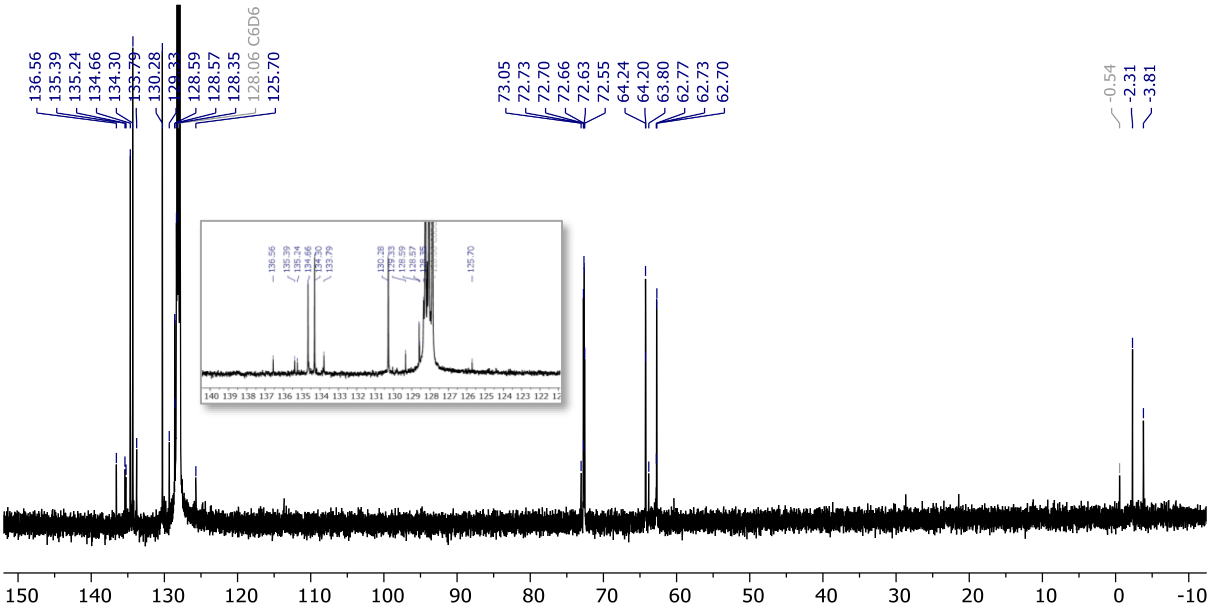

Figure S20: $^{13}C\{^{1}H\}$ NMR spectrum of **2f**.

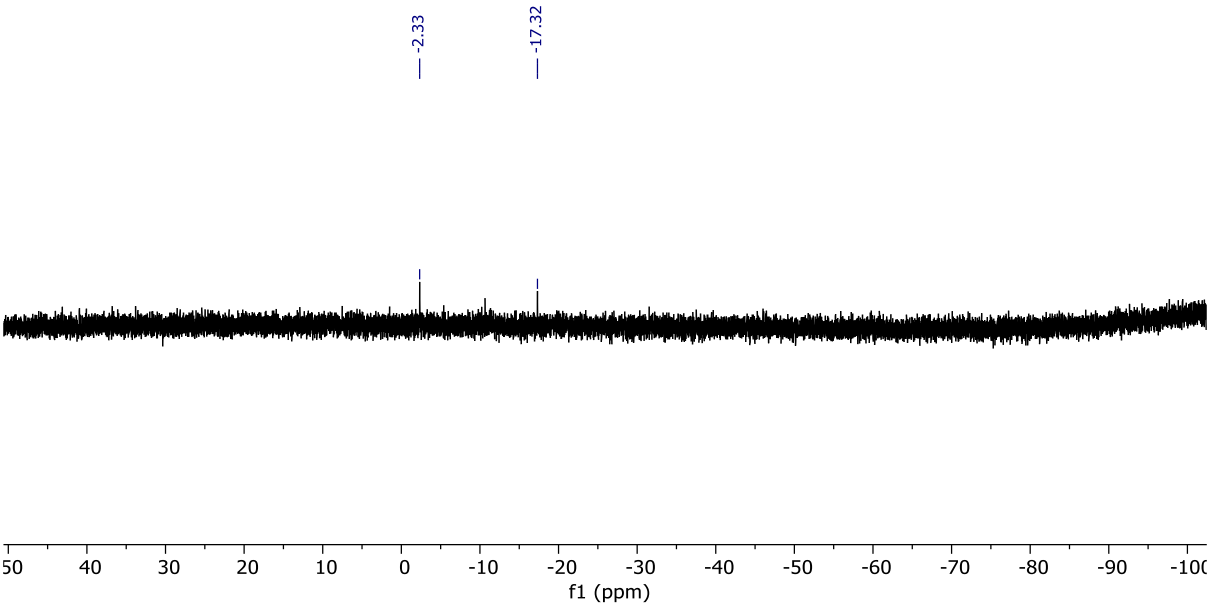

Figure S21: $^{29}Si\{^{1}H\}$ NMR spectrum of **2f**.

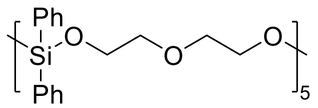

2g

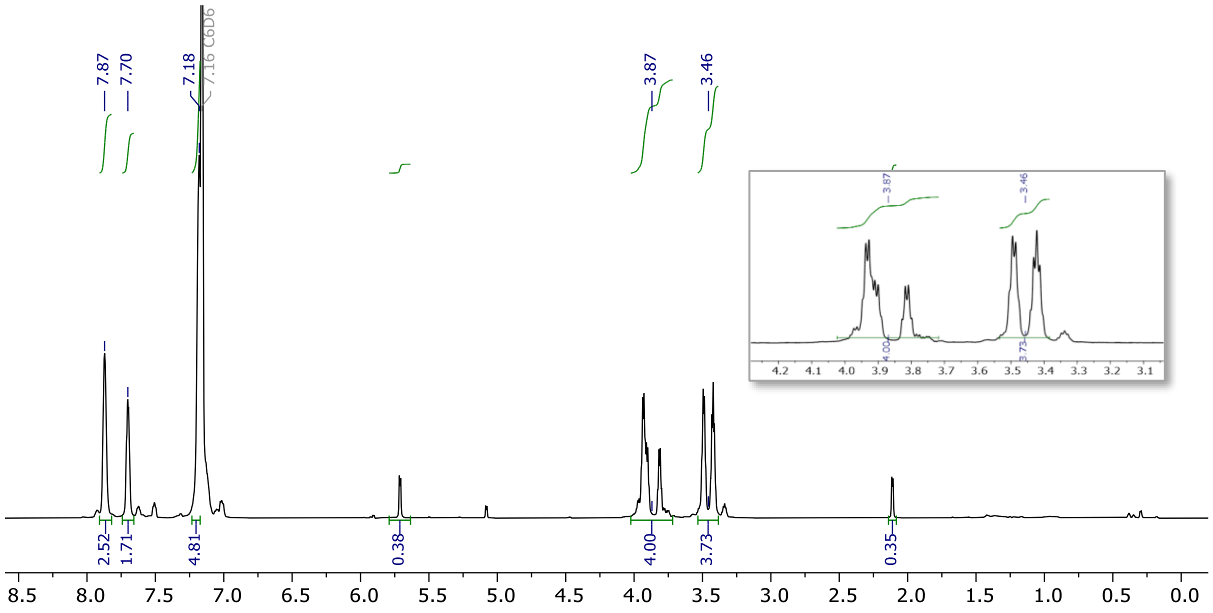

Figure S22: ^{1}H NMR spectrum of **2g**.

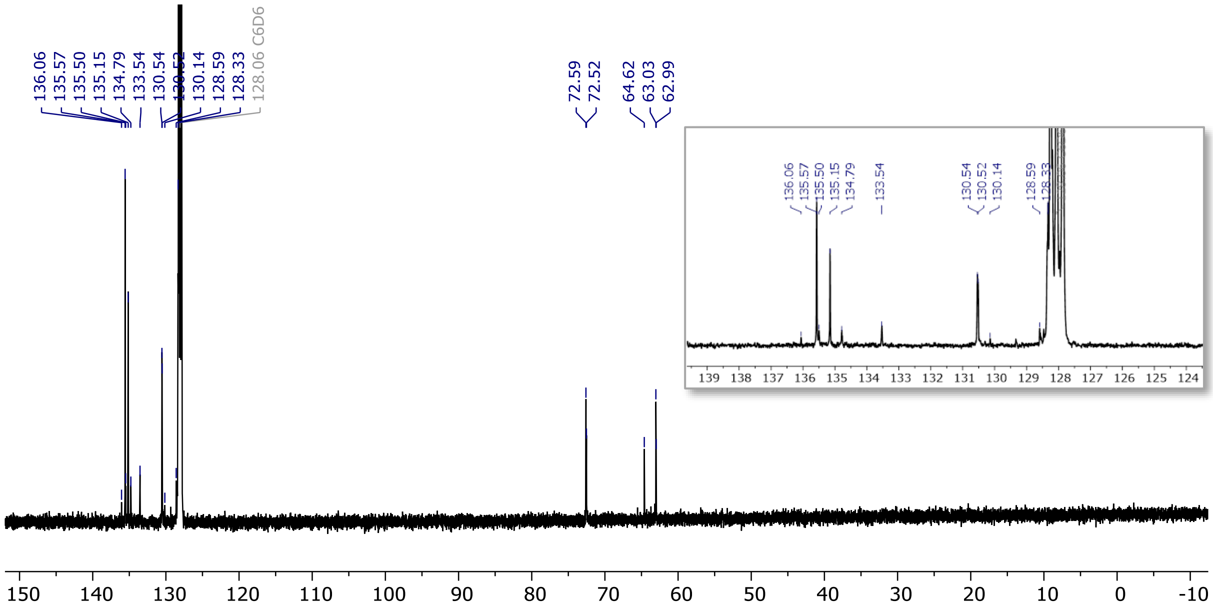

Figure S23: ^{13}C{^{1}H} NMR spectrum of **2g**.

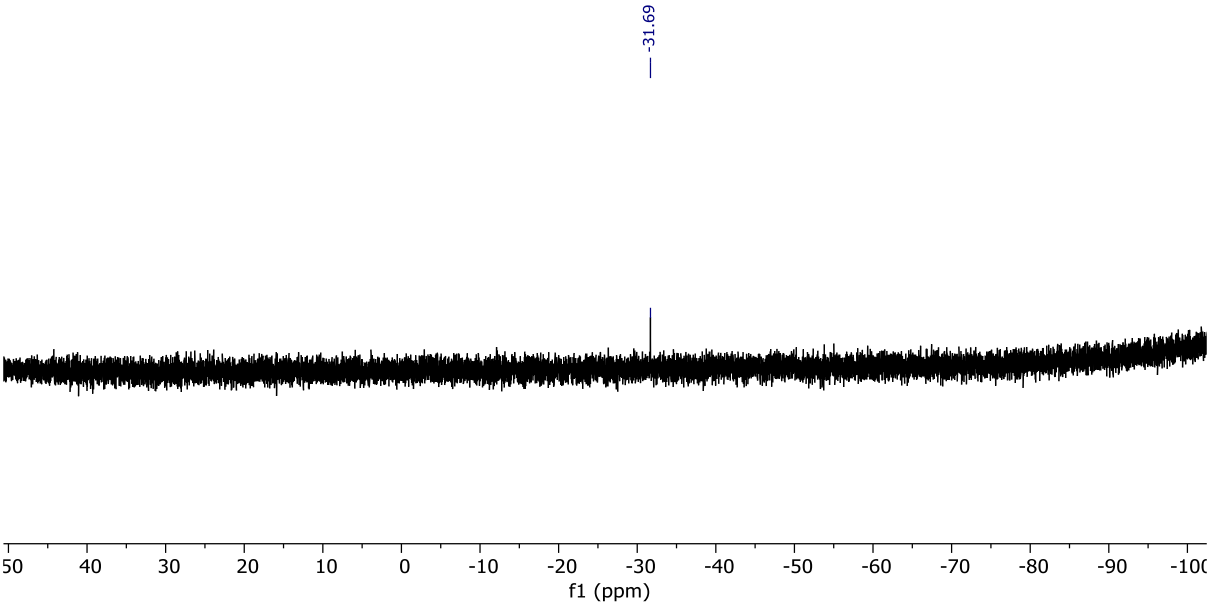

Figure S24: $^{29}Si\{^{1}H\}$ NMR spectrum of **2g**.

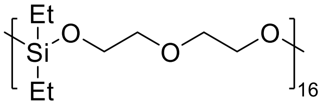

2h

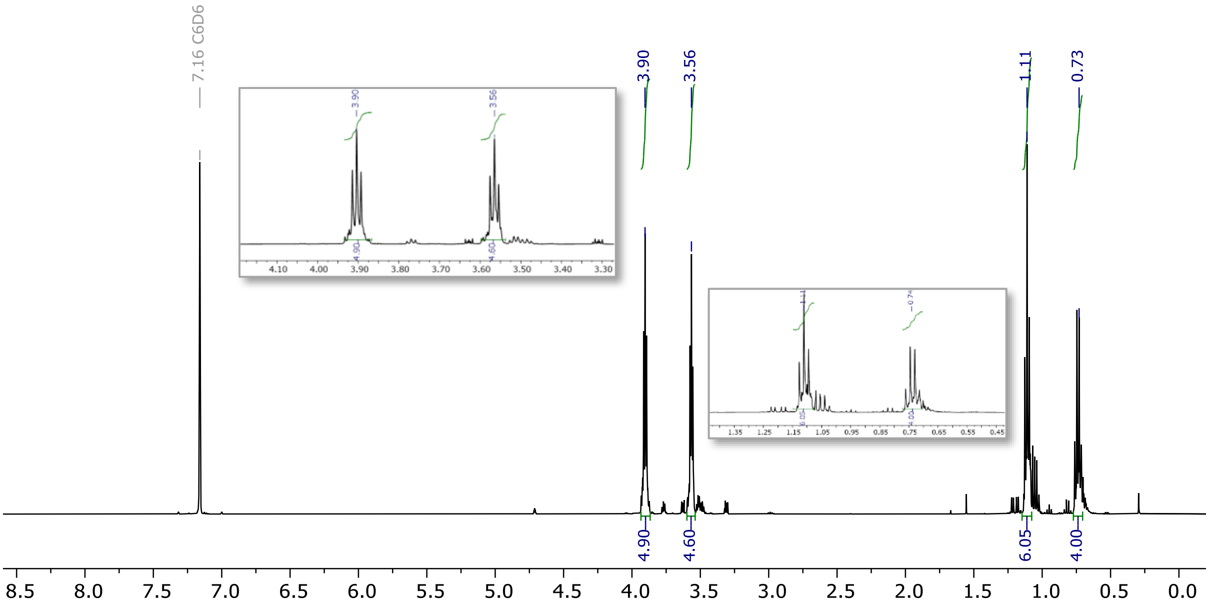

Figure S25: ^{1}H NMR spectrum of **2h**.

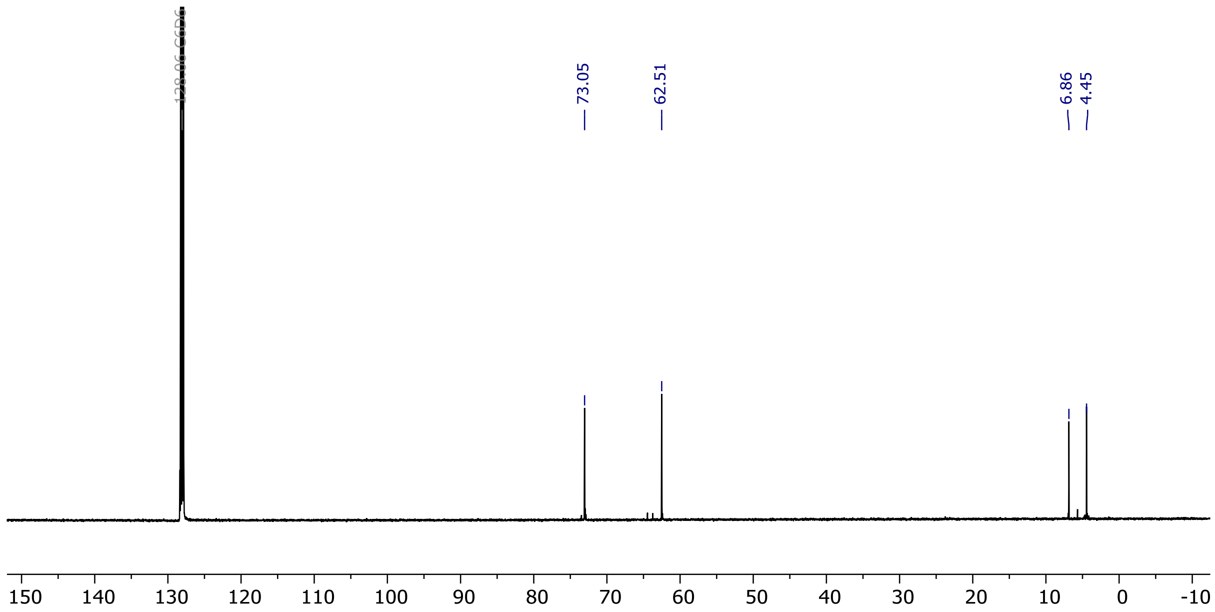

Figure S26: $^{13}C\{^{1}H\}$ NMR spectrum of **2h**.

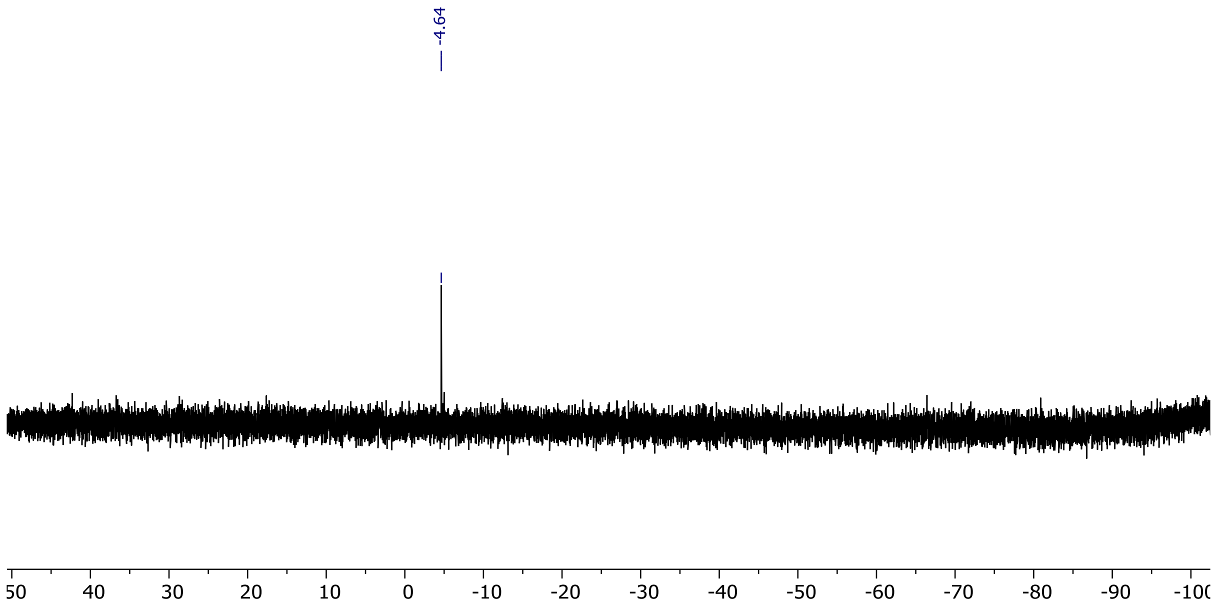

Figure S27: $^{29}Si\{^{1}H\}$ NMR spectrum of **2h**.

2j

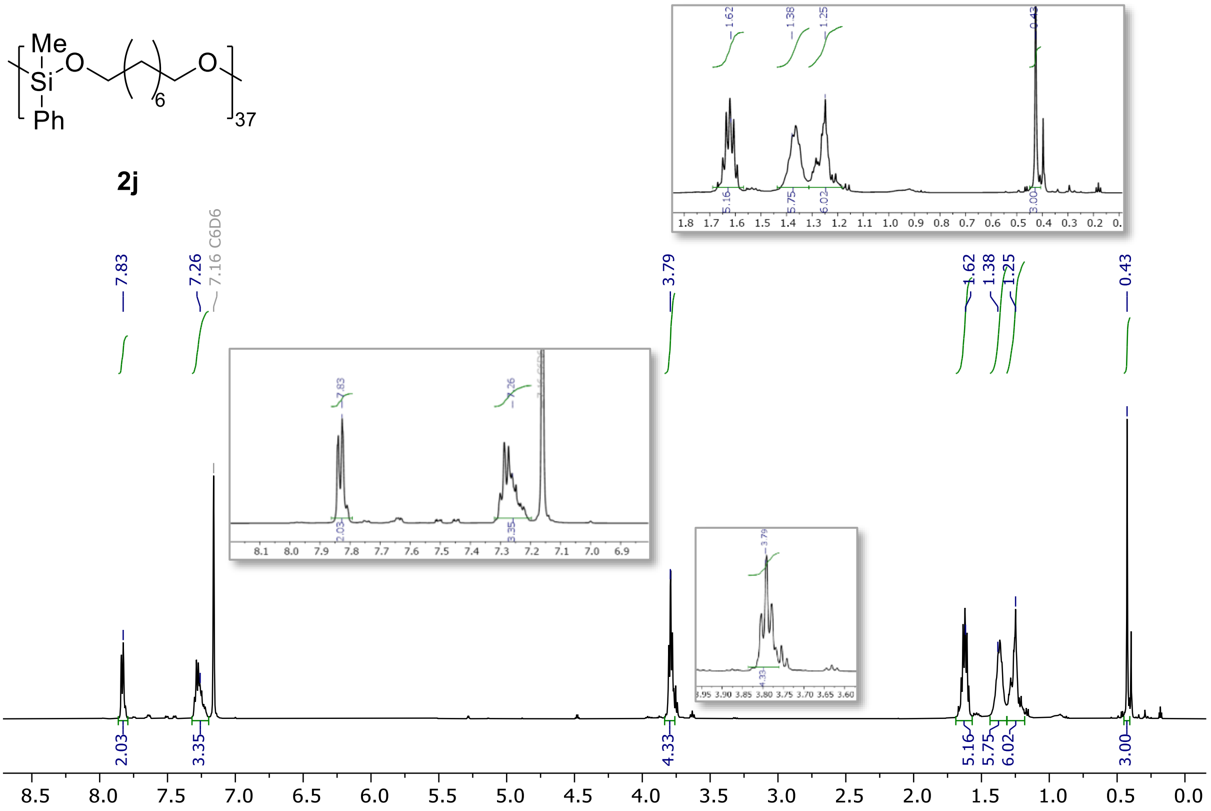

Figure S28: ^{1}H NMR spectrum of **2j**.

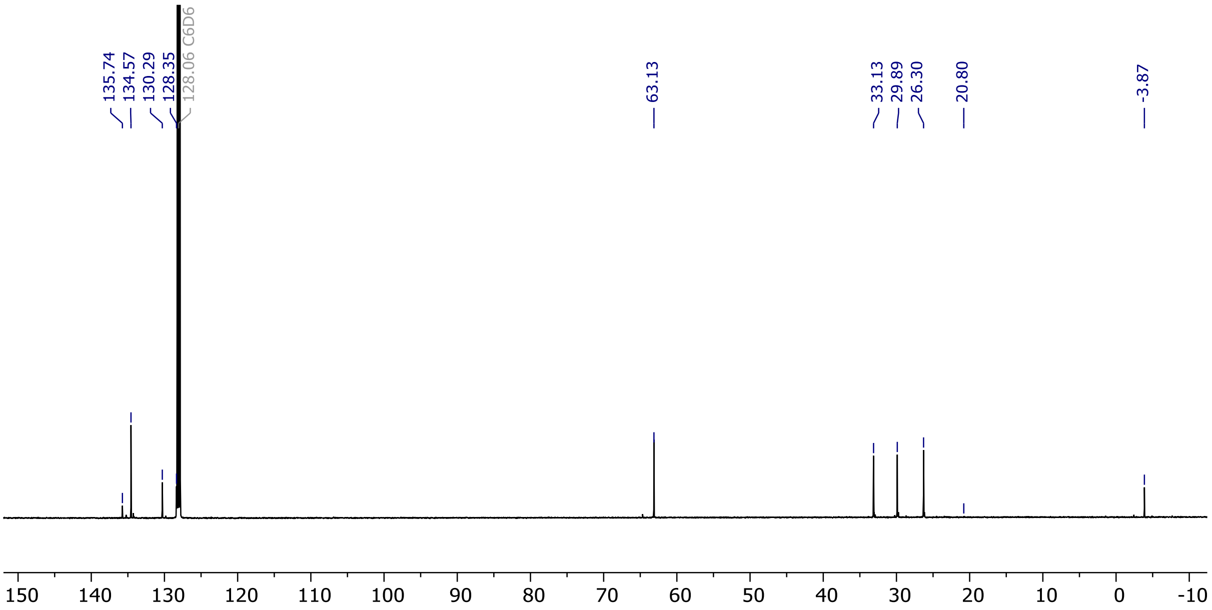

Figure S29: ^{13}C{^{1}H} NMR spectrum of **2j**.

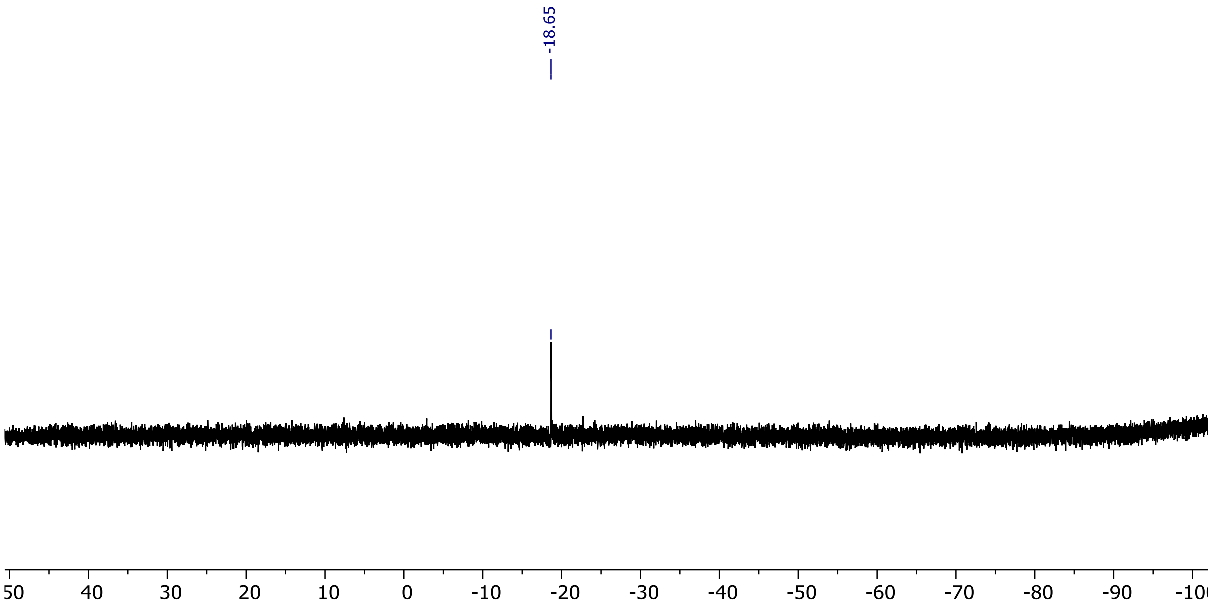

Figure S30: $^{29}Si\{^1H\}$ NMR spectrum of **2j**.

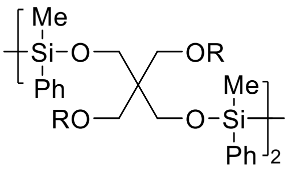

R = -H, -Si(MePh)-

2l

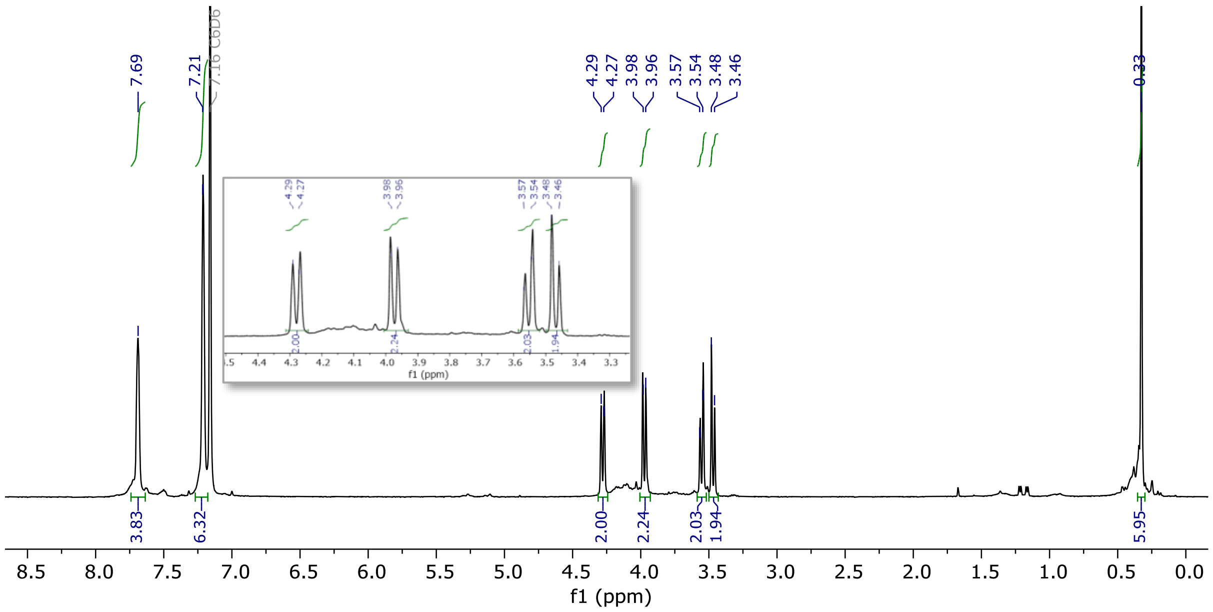

Figure S31: ^1H NMR spectrum of **2l**.

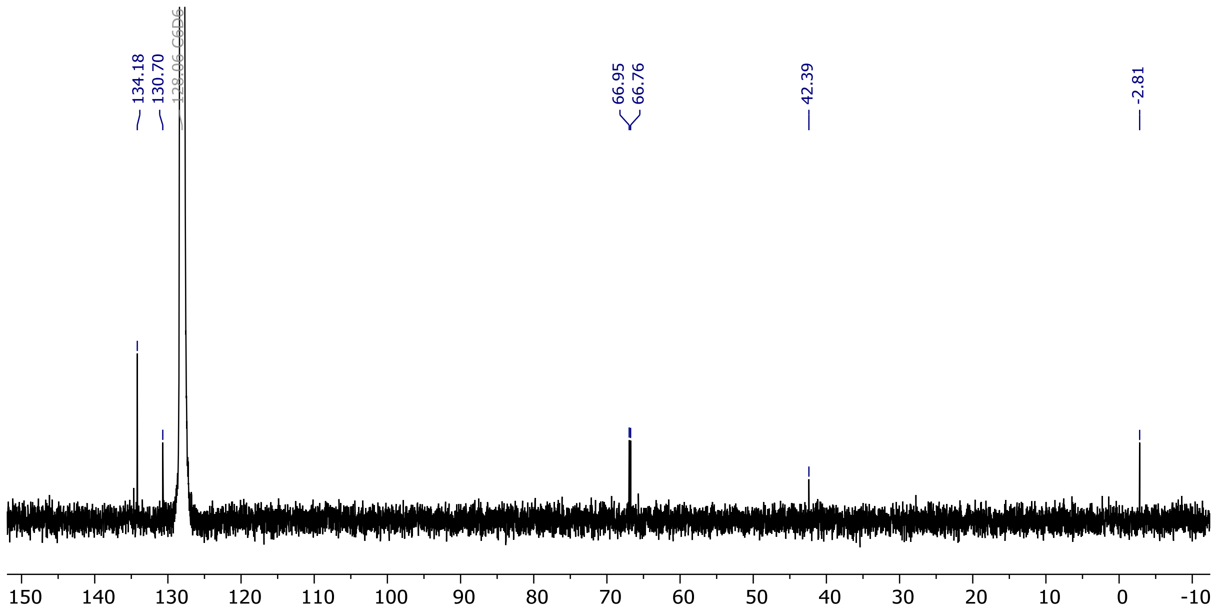

Figure S32: $^{13}C\{^{1}H\}$ NMR spectrum of **2l**.

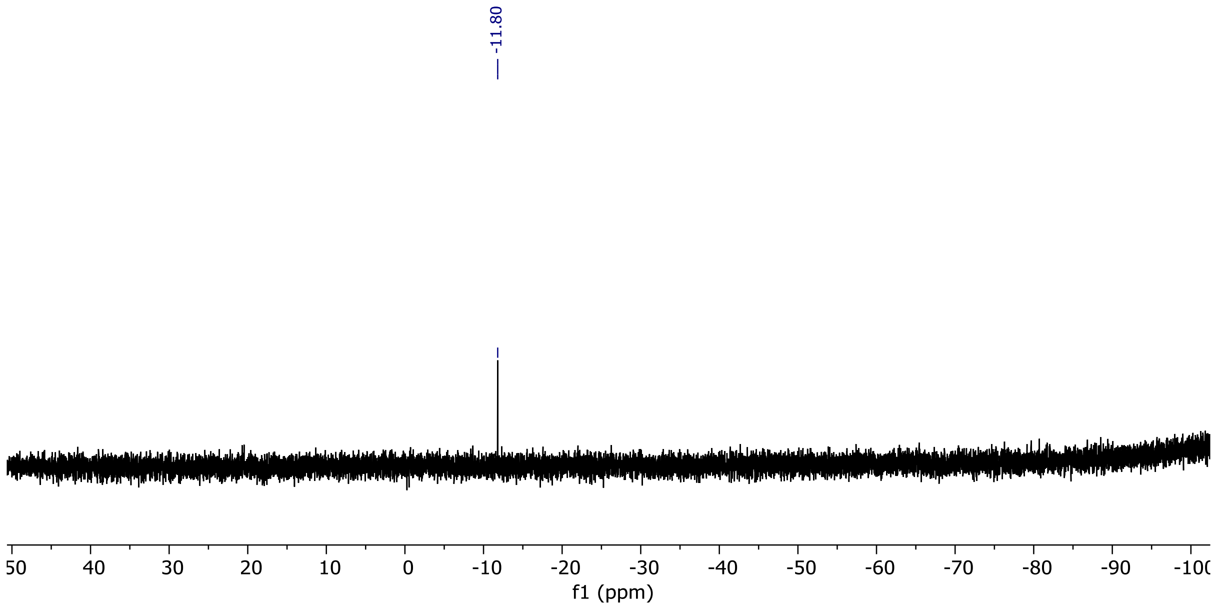

Figure S33: $^{29}Si\{^{1}H\}$ NMR spectrum of **2l**.

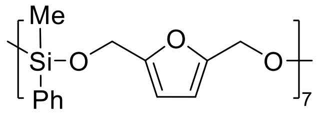

2n

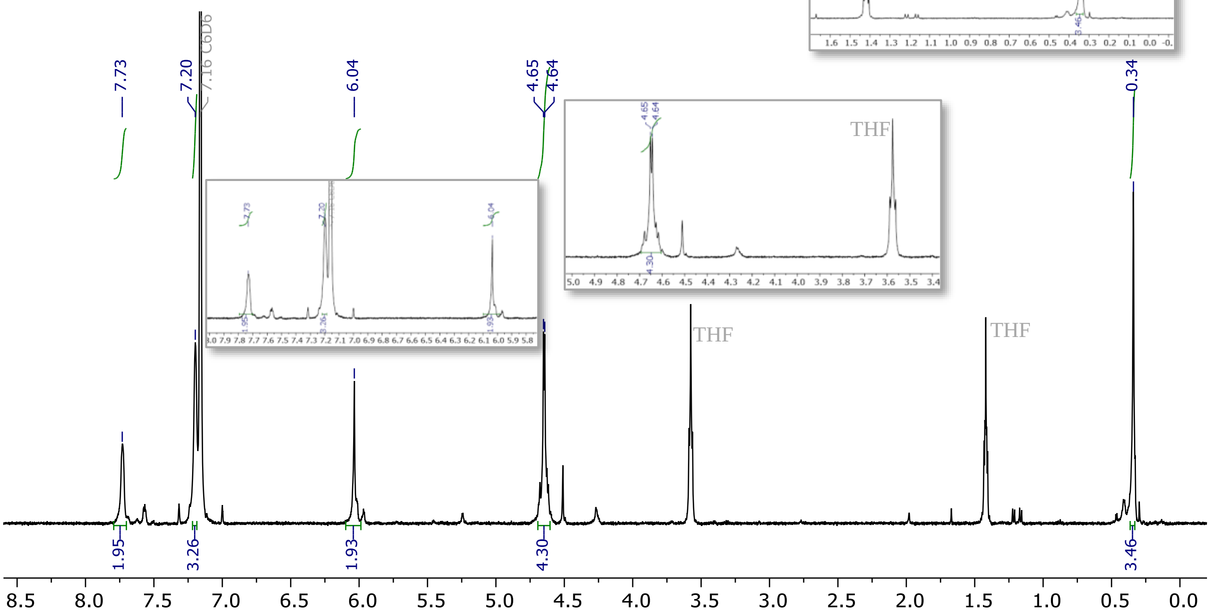

Figure S34: ^{1}H NMR spectrum of **2n**.

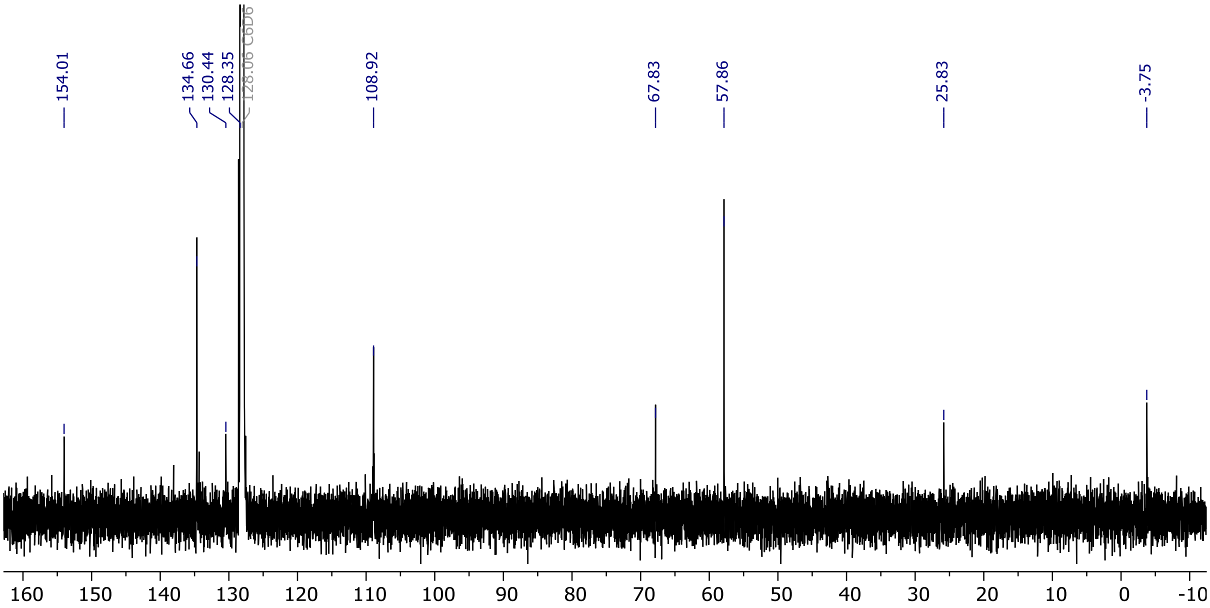

Figure S35: ^{13}C{^{1}H} NMR spectrum of **2n**.

Gel Permeation Chromatography (GPC) data

Optimization procedure

Agilent GPC/SEC Software
Sample GPC Analysis Report

Agilent Technologies

MAJ1-150 crude

**Workspace Details**			
Workspace name	Polylactide		
Location	C:\ProgramData\Agilent Technologies\GPC\Workspaces\Polylactide\		
Comments			
Created by	Administrator at 13:44:31 on 15 June 2015		
**Sample Properties**			
Sample name	MAJ1-150 crude		
File name	ICF_08_02_2022-0005.sample		
Collected by	GPC at 14:38:51 on 08 February 2022		
Instrument name	Instrument 1		
**Column Calibration Details**			
Name	PSty Jan 2022		
Created by	GPC at 07:21:08 on 05 January 2022		
Last modified by	GPC at 07:23:45 on 05 January 2022		
Comments	GPC Column Calibration created 05 January 2022 by GPC<br>GPC Column Calibration amended 05 January 2022 by GPC<br>GPC Column Calibration amended 05 January 2022 by GPC<br>GPC Column Calibration amended 05 January 2022 by GPC		
Calibration Type	Narrow Standard	Curve Fit Used	3
Calibration Curve	y = - 0.003181x^3 + 0.1419x^2 - 2.499x + 20.12		
High Limit MW RT (mins)	11.25000	Low Limit MW RT (mins)	17.58333
High Limit MW (g/mol)	283800	Low Limit MW (g/mol)	580
Flow Rate Marker Name		Flow Marker RT (mins)	0.00000
K (Input) ((10e-5) dL/g)	14.100		
Alpha (Input)	0.700		
Residual Sum Of Squares	0.00186199	Corrected Sum Of Squares	6.97134
Coeff. Of Determination	0.999733	Standard Y Error Estimate	0.0176162
Linear Correlation Coeff	-0.999415		

Column Calibration Data Points

Point	Peak Max RT (mins)	MW	Log MW	Point in Use?	Percent Error
1	11.25000	283800	5.45	Yes	1.92
2	11.85000	135700	5.13	Yes	-3.66
3	12.55000	67600	4.83	Yes	0.37
4	13.43333	29460	4.47	Yes	3.26
5	13.66667	22290	4.35	Yes	-2.84
6	14.65000	9820	3.99	Yes	4.37
7	15.35000	4910	3.69	Yes	-2.15
8	15.85000	3050	3.48	Yes	-4.49
9	16.88333	1250	3.10	Yes	4.07
10	17.58333	580	2.76	Yes	-1.35

Analyst: .... **Date:** ....

Checked By: .... **Date:** ....

Agilent GPC/SEC Software A.02.01 [9] Page 1 of 4

Generated by GPC at 15:25 on 08 February 2022

Agilent GPC/SEC Software
Sample GPC Analysis Report

Agilent Technologies

Processing Parameters

Method	Last modified by Administrator at 13:44:30 on 15 June 2015
Using Flow Rate Correction	No
Mark-Houwink K ((10e-5) dL/g)	14.100
Mark-Houwink Alpha	0.700
Concentration Detector Used in Analysis	RI
Injection volume (µL)	100.00
Flow rate (mL/min)	1.00

MW Ranges Method

Calculate MW Ranges — No

Percentage Fractions Method

Calculate Percentage Fractions — No

Results

Analysed by — GPC at 15:24:51 on 08 February 2022

Comments

Molecular Weight Averages

Peak	Mp (g/mol)	Mn (g/mol)	Mw (g/mol)	Mz (g/mol)	Mz+1 (g/mol)	Mv (g/mol)	PD
Peak 1	2201	1440	2296	3610	5967	3364	1.594

Peak Information

	Start (mins)	End (mins)
Baseline region 1	11.05000	11.68333
Baseline region 2	26.75000	27.50000
Peak 1	13.70000	18.23333

Peak Trace Information

Peak	Trace	Peak Max RT (mins)	Peak Area (mV.s)	Peak Height (mV)
Peak 1	RI	16.26667	1526.133	14.325
Peak 1	VS DP	16.15000	509.017	5.222
Peak 1	VS IP	15.61667	18.428	-0.338
Peak 1	LS 90°	15.88333	111.553	1.012
Peak 1	LS 15°	17.53333	41.928	-0.315

Analyst: .... **Date:** ....

Checked By: .... **Date:** ....

Chromatogram Plot

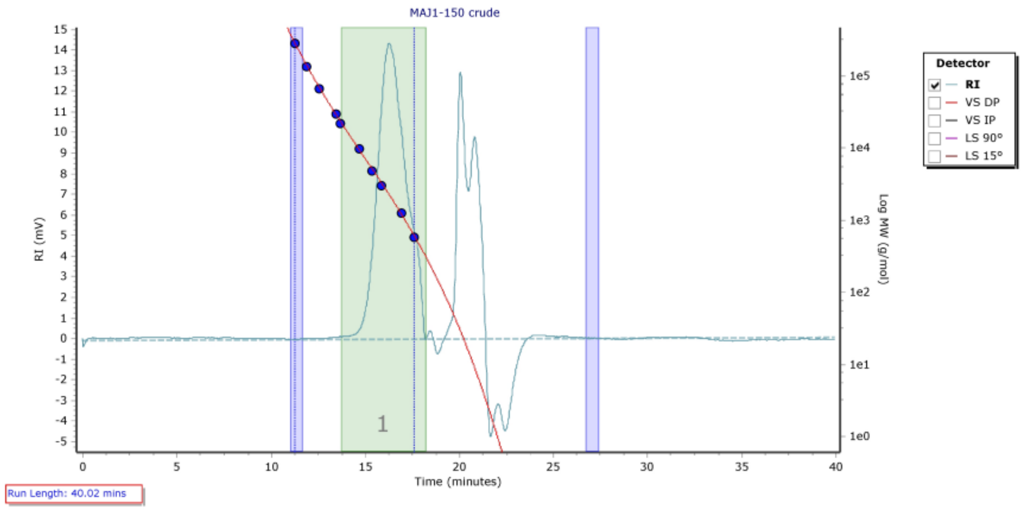

Analyst: .... **Date:** ....

Checked By: .... **Date:** ....

Agilent GPC/SEC Software
Sample GPC Analysis Report

Agilent Technologies

Distribution Plot

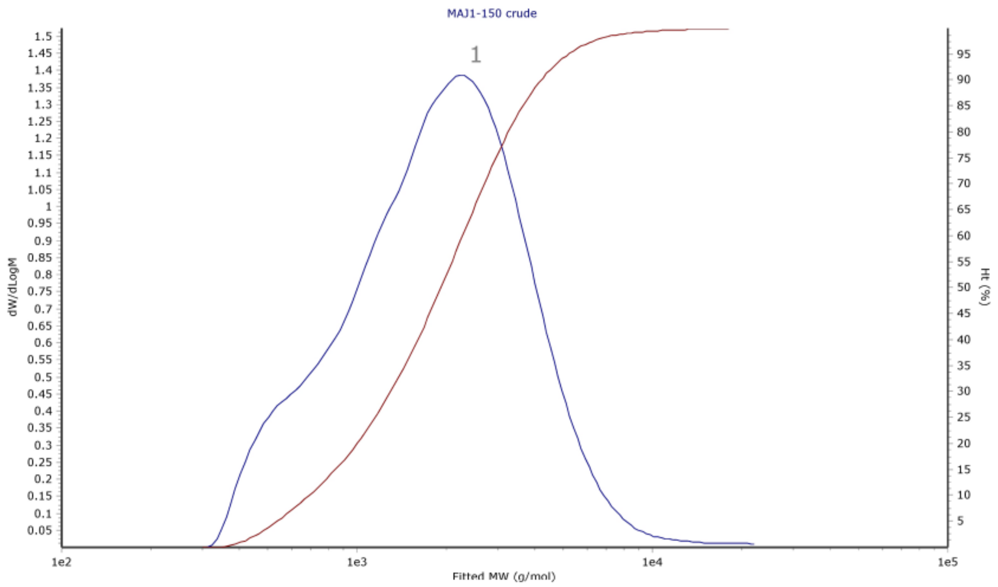

Analyst: .... **Date:** ....

Checked By: .... **Date:** ....

Agilent GPC/SEC Software A.02.01 [9] Page 4 of 4 Generated by GPC at 15:25 on 08 February 2022

Figure S36: GPC data for **Table S1**, Entry 2.

MAJ1-047_1

Workspace Details

Workspace name	Polylactide
Location	C:\ProgramData\Agilent Technologies\GPC\Workspaces\Polylactide\
Comments	
Created by	Administrator at 13:44:31 on 15 June 2015

Sample Properties

Sample name	MAJ1-047_1
File name	ICF_12_07_2021-0002.sample
Collected by	GPC at 13:10:58 on 12 July 2021
Instrument name	Instrument 1

Column Calibration Details

Name	PSty 7thJune2021
Created by	GPC at 10:31:14 on 08 June 2021
Last modified by	GPC at 10:33:07 on 08 June 2021
Comments	GPC Column Calibration created 08 June 2021 by GPC<br>GPC Column Calibration amended 08 June 2021 by GPC<br>GPC Column Calibration amended 08 June 2021 by GPC<br>GPC Column Calibration amended 08 June 2021 by GPC

Calibration Type	Narrow Standard	Curve Fit Used	3
Calibration Curve	y = - 0.002194x^3 + 0.09598x^2 - 1.75x + 16.01		
High Limit MW RT (mins)	10.96667	Low Limit MW RT (mins)	18.08333
High Limit MW (g/mol)	299400	Low Limit MW (g/mol)	580
Flow Rate Marker Name		Flow Marker RT (mins)	0.00000
K (Input) ((10e-5) dL/g)	14.100		
Alpha (Input)	0.700		
Residual Sum Of Squares	0.00194182	Corrected Sum Of Squares	7.33781
Coeff. Of Determination	0.999735	Standard Y Error Estimate	0.0179899
Linear Correlation Coeff	-0.99962		

Column Calibration Data Points

Point	Peak Max RT (mins)	MW	Log MW	Point in Use?	Percent Error
1	10.96667	299400	5.48	Yes	4.04
2	11.56667	151700	5.18	Yes	-5.48
3	12.50000	66350	4.82	Yes	-3.34
4	13.21667	38100	4.58	Yes	2.43
5	14.03333	19880	4.30	Yes	4.54
6	14.83333	9920	4.00	Yes	-0.09
7	15.68333	4920	3.69	Yes	-0.81
8	16.51667	2360	3.37	Yes	-3.97
9	17.28333	1260	3.10	Yes	1.65
10	18.08333	580	2.76	Yes	0.52

Analyst: .... **Date:** ....

Checked By: .... **Date:** ....

Agilent GPC/SEC Software
Sample GPC Analysis Report

Agilent Technologies

Processing Parameters

Method	Last modified by Administrator at 13:44:30 on 15 June 2015
Using Flow Rate Correction	No
Mark-Houwink K ((10e-5) dL/g)	14.100
Mark-Houwink Alpha	0.700
Concentration Detector Used in Analysis	RI
Injection volume (µL)	100.00
Flow rate (mL/min)	1.00

MW Ranges Method

Calculate MW Ranges	No

Percentage Fractions Method

Calculate Percentage Fractions	No

Results

Analysed by	GPC at 18:20:25 on 12 July 2021
Comments	

Molecular Weight Averages

Peak	Mp (g/mol)	Mn (g/mol)	Mw (g/mol)	Mz (g/mol)	Mz+1 (g/mol)	Mv (g/mol)	PD
Peak 1	10064	5421	10155	16953	25004	15889	1.873

Peak Information

	Start (mins)	End (mins)
Baseline region 1	8.68333	9.43333
Baseline region 2	26.25000	27.56667
Peak 1	12.08333	17.21667

Peak Trace Information

Peak	Trace	Peak Max RT (mins)	Peak Area (mV.s)	Peak Height (mV)
Peak 1	RI	14.83333	2159.206	13.810
Peak 1	VS DP	14.60000	1861.681	13.585
Peak 1	VS IP	14.38333	140.503	1.046
Peak 1	LS 90°	14.28333	687.246	5.436
Peak 1	LS 15°	14.20000	176.398	1.481

Analyst: .... **Date:** ....

Checked By: .... **Date:** ....

Agilent GPC/SEC Software
Sample GPC Analysis Report

Agilent Technologies

Chromatogram Plot

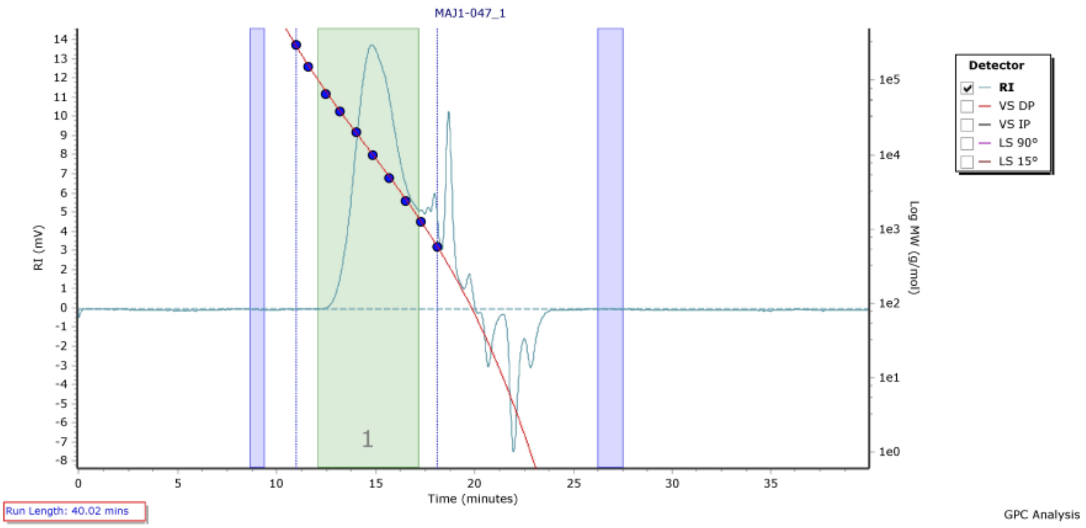

Analyst: .... **Date:** ....

Checked By: .... **Date:** ....

Agilent GPC/SEC Software
Sample GPC Analysis Report

Agilent Technologies

Distribution Plot

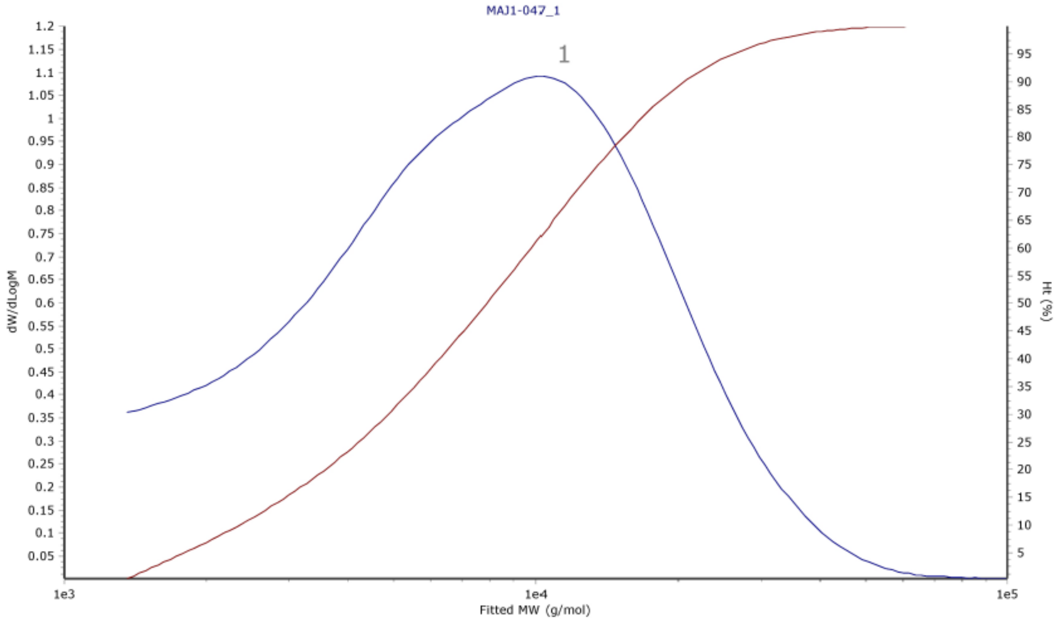

Analyst: .... **Date:** ....

Checked By: .... **Date:** ....

Agilent GPC/SEC Software A.02.01 [9] Page 4 of 4

Generated by GPC at 14:48 on 13 July 2021

Figure S37: GPC data for **Table S1**, Entry 4.

MAJ1-050_2

Workspace Details

Workspace name	Polylactide
Location	C:\ProgramData\Agilent Technologies\GPC\Workspaces\Polylactide\
Comments	
Created by	Administrator at 13:44:31 on 15 June 2015

Sample Properties

Sample name	MAJ1-050_2
File name	ICF_21_07_2021-0035.sample
Collected by	GPC at 11:53:28 on 22 July 2021
Instrument name	Instrument 1

Column Calibration Details

Name	PSty July19th 2021
Created by	GPC at 14:33:05 on 19 July 2021
Last modified by	GPC at 14:35:07 on 19 July 2021
Comments	GPC Column Calibration created 19 July 2021 by GPC<br>GPC Column Calibration amended 19 July 2021 by GPC<br>GPC Column Calibration amended 19 July 2021 by GPC<br>GPC Column Calibration amended 19 July 2021 by GPC

Calibration Type	Narrow Standard	Curve Fit Used	3
Calibration Curve	y = - 0.00208x^3 + 0.09409x^2 - 1.779x + 16.51		
High Limit MW RT (mins)	11.20000	Low Limit MW RT (mins)	18.18333
High Limit MW (g/mol)	299400	Low Limit MW (g/mol)	580
Flow Rate Marker Name		Flow Marker RT (mins)	0.00000
K (Input) ((10e-5) dL/g)	14.100		
Alpha (Input)	0.700		
Residual Sum Of Squares	0.00159464	Corrected Sum Of Squares	7.33781
Coeff. Of Determination	0.999783	Standard Y Error Estimate	0.0163025
Linear Correlation Coeff	-0.999609		

Column Calibration Data Points

Point	Peak Max RT (mins)	MW	Log MW	Point in Use?	Percent Error
1	11.20000	299400	5.48	Yes	3.15
2	11.80000	151700	5.18	Yes	-3.99
3	12.68333	66350	4.82	Yes	-3.04
4	13.35000	38100	4.58	Yes	0.97
5	14.16667	19880	4.30	Yes	5.64
6	14.91667	9920	4.00	Yes	-1.11
7	15.76667	4920	3.69	Yes	-0.54
8	16.60000	2360	3.37	Yes	-3.39
9	17.36667	1260	3.10	Yes	1.56
10	18.18333	580	2.76	Yes	0.33

Analyst: .... **Date:** ....

Checked By: .... **Date:** ....

.

Agilent GPC/SEC Software
Sample GPC Analysis Report

Agilent Technologies

Processing Parameters

Method	Last modified by Administrator at 13:44:30 on 15 June 2015
Using Flow Rate Correction	No
Mark-Houwink K ((10e-5) dL/g)	14.100
Mark-Houwink Alpha	0.700
Concentration Detector Used in Analysis	RI
Injection volume (µL)	100.00
Flow rate (mL/min)	1.00

MW Ranges Method

Calculate MW Ranges	No

Percentage Fractions Method

Calculate Percentage Fractions	No

Results

Analysed by	GPC at 12:34:56 on 22 July 2021
Comments	

Molecular Weight Averages

Peak	Mp (g/mol)	Mn (g/mol)	Mw (g/mol)	Mz (g/mol)	Mz+1 (g/mol)	Mv (g/mol)	PD
Peak 1	23503	12321	73618	383064	992808	315748	5.975

Peak Information

	Start (mins)	End (mins)
Baseline region 1	7.63333	8.15000
Baseline region 2	24.25000	25.16667
Peak 1	9.21667	17.90000

Peak Trace Information

Peak	Trace	Peak Max RT (mins)	Peak Area (mV.s)	Peak Height (mV)
Peak 1	RI	13.86667	2436.869	10.974
Peak 1	VS DP	12.76667	5707.837	24.405
Peak 1	VS IP	12.36667	192.094	1.324
Peak 1	LS 90°	11.28333	5703.652	29.985
Peak 1	LS 15°	11.30000	1787.520	9.453

Analyst: .... **Date:** ....

Checked By: .... **Date:** ....

Chromatogram Plot

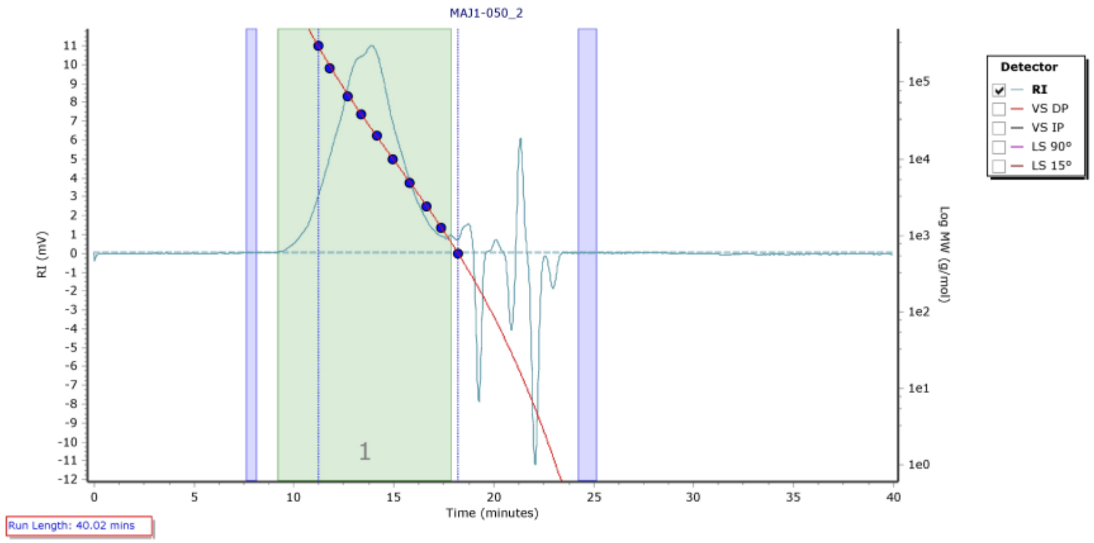

Analyst: .... **Date:** ....

Checked By: .... **Date:** ....

Agilent GPC/SEC Software
Sample GPC Analysis Report

Agilent Technologies

Distribution Plot

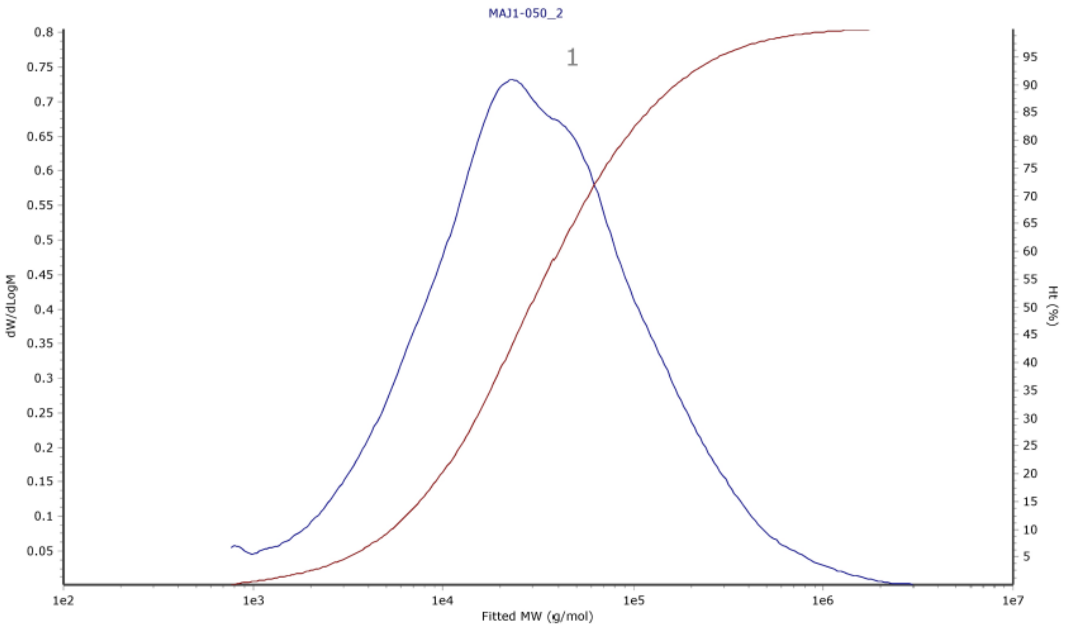

Analyst: .... **Date:** ....

Checked By: .... **Date:** ....

Agilent GPC/SEC Software A.02.01 [9] Page 4 of 4

Generated by GPC at 12:35 on 22 July 2021

Figure S38: GPC data for **Table S1**, Entry 5.

Agilent Technologies

MAJ1-186 PI

Workspace Details

Workspace name	Polylactide
Location	C:\ProgramData\Agilent Technologies\GPC\Workspaces\Polylactide\
Comments	
Created by	Administrator at 13:44:31 on 15 June 2015

Sample Properties

Sample name	MAJ1-186 PI
File name	ICF_22_06_2022-0020-1.sample
Collected by	GPC at 14:26:12 on 26 June 2022
Instrument name	Instrument 1

Column Calibration Details

Name	PSty26thMay2022
Created by	GPC at 14:09:29 on 26 May 2022
Last modified by	GPC at 14:10:44 on 26 May 2022
Comments	GPC Column Calibration created 26 May 2022 by GPC<br>GPC Column Calibration amended 26 May 2022 by GPC<br>GPC Column Calibration amended 26 May 2022 by GPC<br>GPC Column Calibration amended 26 May 2022 by GPC<br>GPC Column Calibration amended 26 May 2022 by GPC

Calibration Type	Narrow Standard	Curve Fit Used	3
Calibration Curve	y = - 0.001896x^3 + 0.08659x^2 - 1.684x + 16.11		
High Limit MW RT (mins)	11.21667	Low Limit MW RT (mins)	18.08333
High Limit MW (g/mol)	283800	Low Limit MW (g/mol)	580
Flow Rate Marker Name		Flow Marker RT (mins)	0.00000
K (Input) ((10e-5) dL/g)	14.100		
Alpha (Input)	0.700		
Residual Sum Of Squares	0.00330604	Corrected Sum Of Squares	8.92203
Coeff. Of Determination	0.999629	Standard Y Error Estimate	0.0191661
Linear Correlation Coeff	-0.999452		

Column Calibration Data Points

Point	Peak Max RT (mins)	MW	Log MW	Point in Use?	Percent Error
1	11.21667	283800	5.45	Yes	2.59
2	11.21667	283800	5.45	Yes	2.59
3	11.83333	135700	5.13	Yes	-8.47
4	12.63333	67600	4.83	Yes	-0.98
5	13.58333	29480	4.47	Yes	1.73
6	13.58333	29480	4.47	Yes	1.73
7	13.88333	22290	4.35	Yes	-0.05
8	14.93333	9820	3.99	Yes	7.23
9	15.63333	4910	3.69	Yes	-2.81
10	15.63333	4910	3.69	Yes	-2.81
11	16.18333	3050	3.48	Yes	-3.74
12	17.26667	1250	3.10	Yes	1.77
13	18.08333	580	2.76	Yes	0.33

Analyst: .... **Date:** ....

Checked By: .... **Date:** ....

Agilent GPC/SEC Software
Sample GPC Analysis Report

Agilent Technologies

Processing Parameters

Method	Last modified by Administrator at 13:44:30 on 15 June 2015
Using Flow Rate Correction	No
Mark-Houwink K ((10e-5) dL/g)	14.100
Mark-Houwink Alpha	0.700
Concentration Detector Used in Analysis	RI
Injection volume (µL)	100.00
Flow rate (mL/min)	1.00

MW Ranges Method

Calculate MW Ranges No

Percentage Fractions Method

Calculate Percentage Fractions No

Results

Analysed by GPC at 15:28:18 on 29 June 2022

Comments

Molecular Weight Averages

Peak	Mp (g/mol)	Mn (g/mol)	Mw (g/mol)	Mz (g/mol)	Mz+1 (g/mol)	Mv (g/mol)	PD
Peak 1	49484	24657	80123	207965	359070	187095	3.25

Peak Information

	Start (mins)	End (mins)
Baseline region 1	4.60000	5.25000
Baseline region 2	29.93333	30.40000
Peak 1	10.25000	16.15000

Peak Trace Information

Peak	Trace	Peak Max RT (mins)	Peak Area (mV.s)	Peak Height (mV)
Peak 1	RI	12.98333	722.341	3.831
Peak 1	VS DP	12.13333	2202.868	11.667
Peak 1	VS IP	11.75000	103.792	0.664
Peak 1	LS 90°	11.53333	1738.586	12.024
Peak 1	LS 15°	11.53333	506.938	3.677

Analyst: .... **Date:** ....

Checked By: .... **Date:** ....

Chromatogram Plot

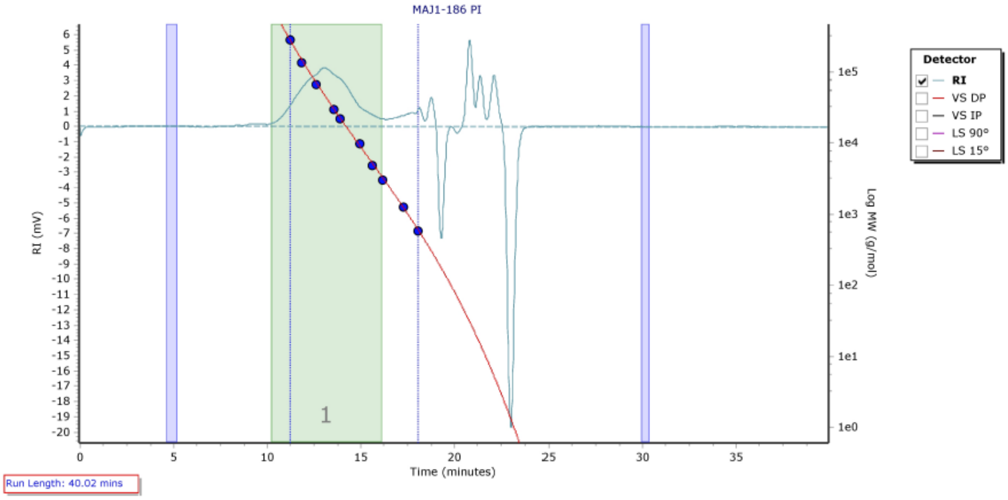

Analyst: .... **Date:** ....

Checked By: .... **Date:** ....

Agilent GPC/SEC Software
Sample GPC Analysis Report

Agilent Technologies

Distribution Plot

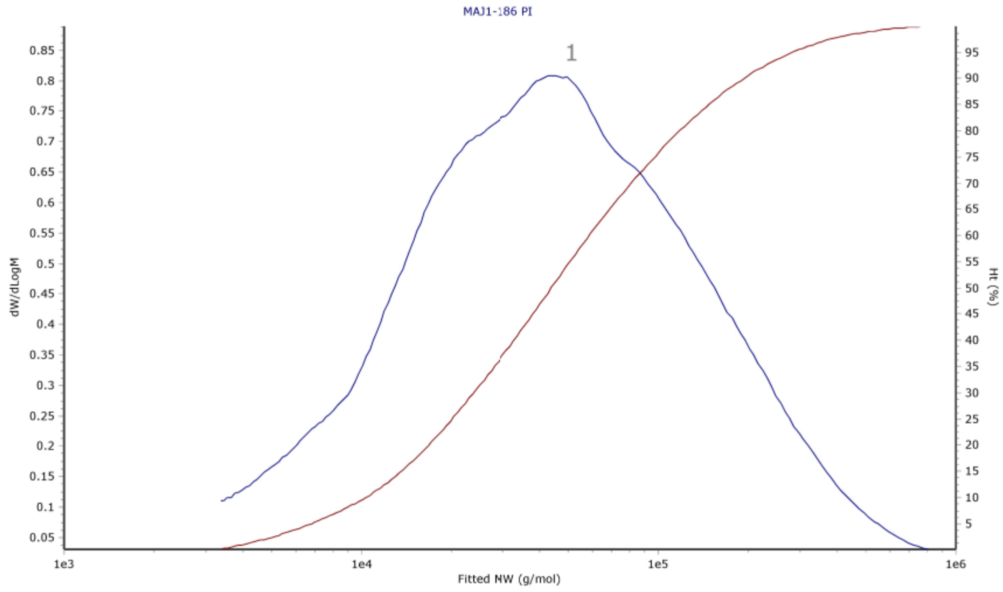

Analyst: .... **Date:** ....

Checked By: .... **Date:** ....

Agilent GPC/SEC Software A.02.01 [9] Page 4 of 4

Generated by GPC at 15:28 on 29 June 2022

Figure S39: GPC data for **Table S1**, Entry 7.

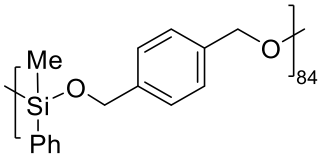

2a

Agilent GPC/SEC Software
Sample GPC Analysis Report

Agilent Technologies

MAJ1-116

**Workspace Details**	
Workspace name	Polylactide
Location	C:\ProgramData\Agilent Technologies\GPC\Workspaces\Polylactide\
Comments	
Created by	Administrator at 13:44:31 on 15 June 2015

**Sample Properties**	
Sample name	MAJ1-116
File name	ICF_25_10_2021-0004.sample
Collected by	GPC at 17:03:43 on 25 October 2021
Instrument name	Instrument 1

**Column Calibration Details**	
Name	PSty July19th 2021
Created by	GPC at 14:33:05 on 19 July 2021
Last modified by	GPC at 14:35:07 on 19 July 2021
Comments	GPC Column Calibration created 19 July 2021 by GPC<br>GPC Column Calibration amended 19 July 2021 by GPC<br>GPC Column Calibration amended 19 July 2021 by GPC<br>GPC Column Calibration amended 19 July 2021 by GPC

Calibration Type	Narrow Standard	Curve Fit Used	3
Calibration Curve	y = - 0.00208x^3 + 0.09409x^2 - 1.779x + 16.51		
High Limit MW RT (mins)	11.20000	Low Limit MW RT (mins)	18.18333
High Limit MW (g/mol)	299400	Low Limit MW (g/mol)	580
Flow Rate Marker Name		Flow Marker RT (mins)	0.00000
K (Input) ((10e-5) dL/g)	14.100		
Alpha (Input)	0.700		
Residual Sum Of Squares	0.00159464	Corrected Sum Of Squares	7.33781
Coeff. Of Determination	0.999783	Standard Y Error Estimate	0.0163025
Linear Correlation Coeff	-0.999609		

Column Calibration Data Points

Point	Peak Max RT (mins)	MW	Log MW	Point in Use?	Percent Error
1	11.20000	299400	5.48	Yes	3.15
2	11.80000	151700	5.18	Yes	-3.99
3	12.68333	66350	4.82	Yes	-3.04
4	13.35000	38100	4.58	Yes	0.97
5	14.16667	19880	4.30	Yes	5.64
6	14.91667	9920	4.00	Yes	-1.11
7	15.76667	4920	3.69	Yes	-0.54
8	16.60000	2360	3.37	Yes	-3.39
9	17.36667	1260	3.10	Yes	1.56
10	18.18333	580	2.76	Yes	0.33

Analyst: .... **Date:** ....

Checked By: .... **Date:** ....

Agilent GPC/SEC Software
Sample GPC Analysis Report

Agilent Technologies

Processing Parameters

Method	Last modified by Administrator at 13:44:30 on 15 June 2015
Using Flow Rate Correction	No
Mark-Houwink K ((10e-5) dL/g)	14.100
Mark-Houwink Alpha	0.700
Concentration Detector Used in Analysis	RI
Injection volume (μL)	100.00
Flow rate (mL/min)	1.00

MW Ranges Method

Calculate MW Ranges	No

Percentage Fractions Method

Calculate Percentage Fractions	No

Results

Analysed by	GPC at 14:25:37 on 28 October 2021
Comments	

Molecular Weight Averages

Peak	Mp (g/mol)	Mn (g/mol)	Mw (g/mol)	Mz (g/mol)	Mz+1 (g/mol)	Mv (g/mol)	PD
Peak 1	38846	21542	70584	228146	755935	192439	3.277

Peak Information

	Start (mins)	End (mins)
Baseline region 1	8.13333	8.73333
Baseline region 2	33.93333	34.68333
Peak 1	8.90000	16.16667

Peak Trace Information

Peak	Trace	Peak Max RT (mins)	Peak Area (mV.s)	Peak Height (mV)
Peak 1	RI	13.31667	1916.686	9.884
Peak 1	VS DP	12.46667	4126.026	21.709
Peak 1	VS IP	11.45000	135.431	1.020
Peak 1	LS 90°	12.03333	2587.851	13.628
Peak 1	LS 15°	12.18333	809.931	4.277

Analyst: .... **Date:** ....

Checked By: .... **Date:** ....

Agilent GPC/SEC Software
Sample GPC Analysis Report

Agilent Technologies

Chromatogram Plot

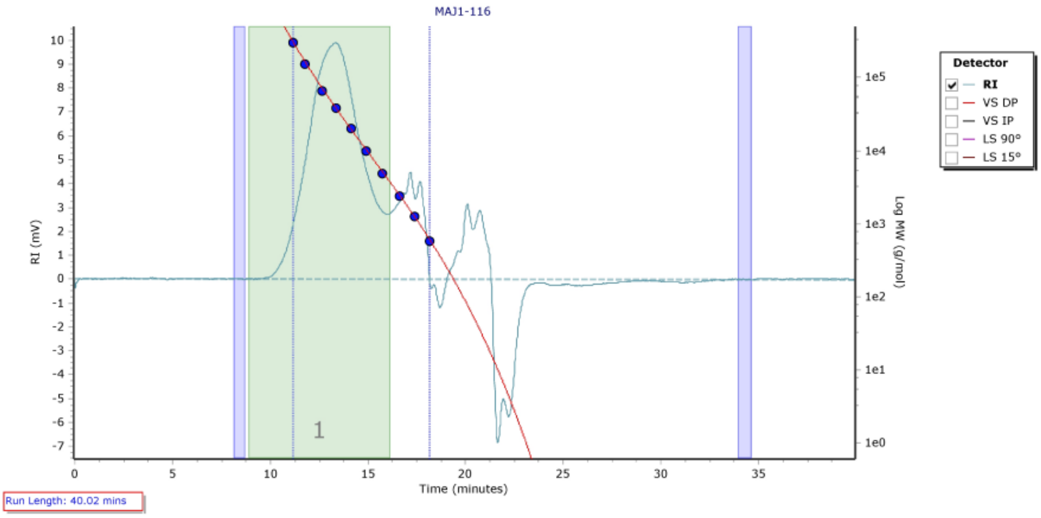

Analyst: .... **Date:** ....

Checked By: .... **Date:** ....

Agilent GPC/SEC Software
Sample GPC Analysis Report

Distribution Plot

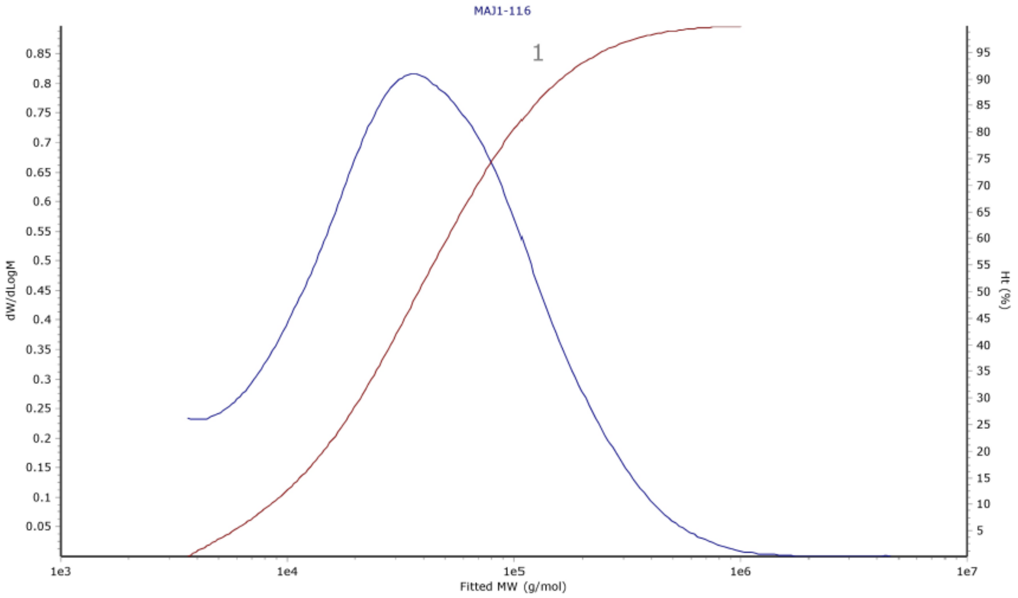

Analyst: .... **Date:** ....

Checked By: .... **Date:** ....

Agilent GPC/SEC Software A.02.01 [9] Page 4 of 4

Generated by GPC at 14:25 on 28 October 2021

Figure S40: GPC data of **2a**.

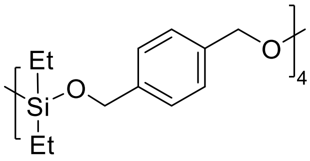

2b

Agilent GPC/SEC Software
Sample GPC Analysis Report

Agilent Technologies

MAJ1-182 PS only

Workspace Details

Workspace name	Polylactide
Location	C:\ProgramData\Agilent Technologies\GPC\Workspaces\Polylactide\
Comments	
Created by	Administrator at 13:44:31 on 15 June 2015

Sample Properties

Sample name	MAJ1-182 PS only
File name	ICF_22_06_2022-0005-1.sample
Collected by	GPC at 14:37:33 on 22 June 2022
Instrument name	Instrument 1

Column Calibration Details

Name	PSty26thMay2022
Created by	GPC at 14:09:29 on 26 May 2022
Last modified by	GPC at 14:10:44 on 26 May 2022
Comments	GPC Column Calibration created 26 May 2022 by GPC<br>GPC Column Calibration amended 26 May 2022 by GPC<br>GPC Column Calibration amended 26 May 2022 by GPC<br>GPC Column Calibration amended 26 May 2022 by GPC<br>GPC Column Calibration amended 26 May 2022 by GPC

Calibration Type	Narrow Standard	Curve Fit Used	3
Calibration Curve	y = - 0.001896x^3 + 0.08659x^2 - 1.684x + 16.11		
High Limit MW RT (mins)	11.21667	Low Limit MW RT (mins)	18.08333
High Limit MW (g/mol)	283800	Low Limit MW (g/mol)	580
Flow Rate Marker Name		Flow Marker RT (mins)	0.00000
K (Input) ((10e-5) dL/g)	14.100		
Alpha (Input)	0.700		
Residual Sum Of Squares	0.00330604	Corrected Sum Of Squares	8.92203
Coeff. Of Determination	0.999629	Standard Y Error Estimate	0.0191661
Linear Correlation Coeff	-0.999452		

Column Calibration Data Points

Point	Peak Max RT (mins)	MW	Log MW	Point in Use?	Percent Error
1	11.21667	283800	5.45	Yes	2.59
2	11.21667	283800	5.45	Yes	2.59
3	11.83333	135700	5.13	Yes	-8.47
4	12.63333	67600	4.83	Yes	-0.98
5	13.58333	29480	4.47	Yes	1.73
6	13.58333	29480	4.47	Yes	1.73
7	13.88333	22290	4.35	Yes	-0.05
8	14.93333	9820	3.99	Yes	7.23
9	15.63333	4910	3.69	Yes	-2.81
10	15.63333	4910	3.69	Yes	-2.81
11	16.18333	3050	3.48	Yes	-3.74
12	17.26667	1250	3.10	Yes	1.77
13	18.08333	580	2.76	Yes	0.33

Analyst: .... **Date:** ....

Checked By: .... **Date:** ....

Agilent GPC/SEC Software A.02.01 [9] Page 1 of 4

Generated by GPC at 11:06 on 05 July 2022

Agilent GPC/SEC Software
Sample GPC Analysis Report

Agilent Technologies

Processing Parameters

Method	Last modified by Administrator at 13:44:30 on 15 June 2015
Using Flow Rate Correction	No
Mark-Houwink K ((10e-5) dL/g)	14.100
Mark-Houwink Alpha	0.700
Concentration Detector Used in Analysis	RI
Injection volume (µL)	100.00
Flow rate (mL/min)	1.00

MW Ranges Method

Calculate MW Ranges	No

Percentage Fractions Method

Calculate Percentage Fractions	No

Results

Analysed by	GPC at 11:06:23 on 05 July 2022
Comments	

Molecular Weight Averages

Peak	Mp (g/mol)	Mn (g/mol)	Mw (g/mol)	Mz (g/mol)	Mz+1 (g/mol)	Mv (g/mol)	PD
Peak 1	543	897	1190	1613	2118	1543	1.327

Peak Information

	Start (mins)	End (mins)
Baseline region 1	7.30000	8.05000
Baseline region 2	26.68333	27.41667
Peak 1	15.45000	18.50000

Peak Trace Information

Peak	Trace	Peak Max RT (mins)	Peak Area (mV.s)	Peak Height (mV)
Peak 1	RI	18.16667	716.987	8.931
Peak 1	VS DP	17.66667	236.872	2.535
Peak 1	VS IP	17.31667	32.262	0.354
Peak 1	LS 90°	17.68333	11.789	0.191
Peak 1	LS 15°	18.10000	13.382	-0.154

Analyst: .... **Date:** ....

Checked By: .... **Date:** ....

Chromatogram Plot

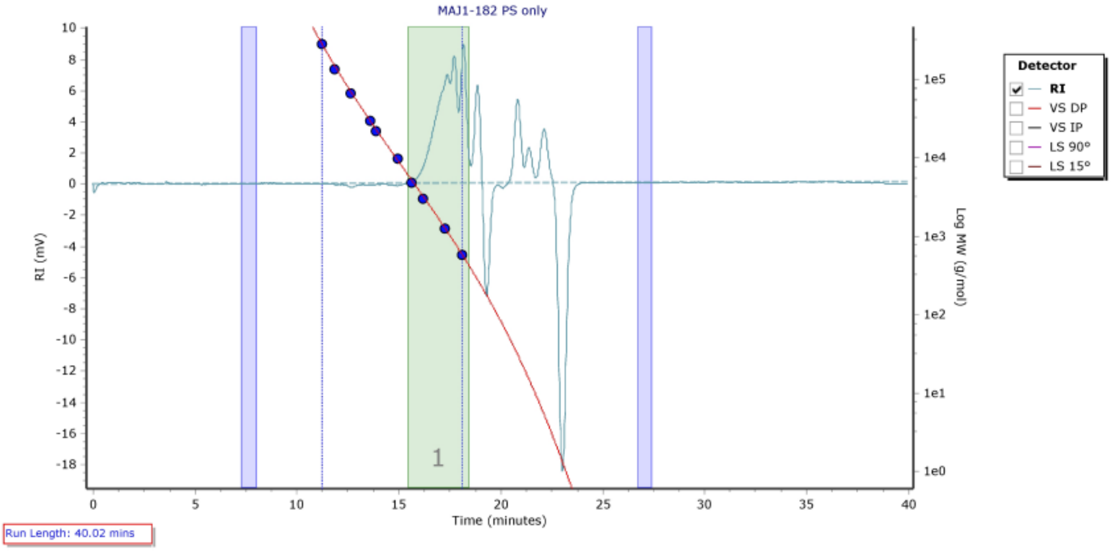

Analyst: .... **Date:** ....

Checked By: .... **Date:** ....

Agilent GPC/SEC Software
Sample GPC Analysis Report

Distribution Plot

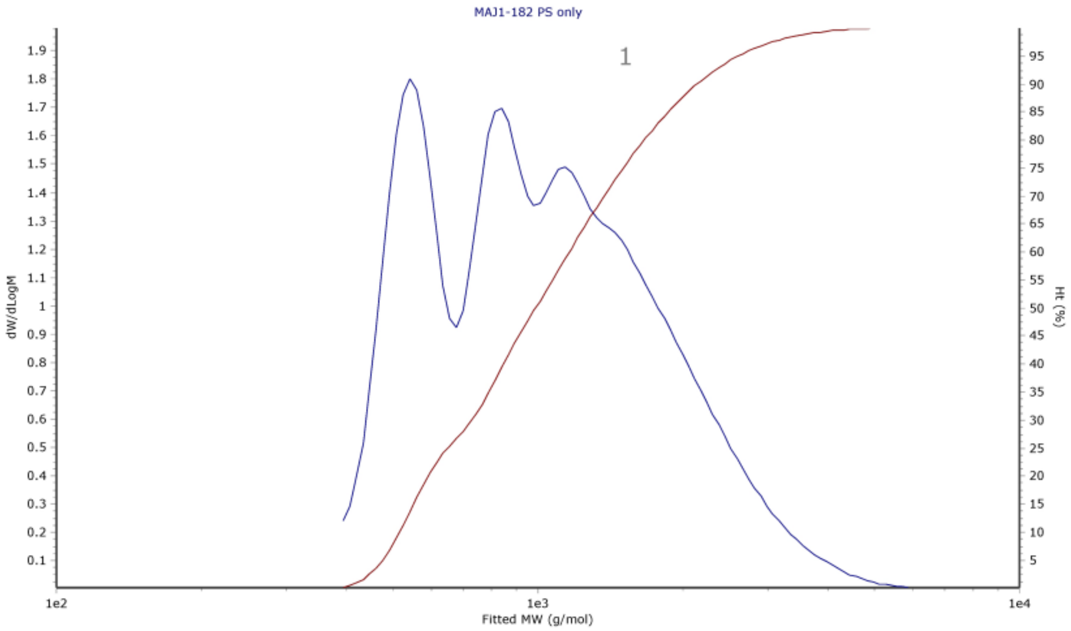

Analyst: .... **Date:** ....

Checked By: .... **Date:** ....

Agilent GPC/SEC Software A.02.01 [9] Page 4 of 4

Generated by GPC at 11:06 on 05 July 2022

Figure S41: GPC data of **2b**.

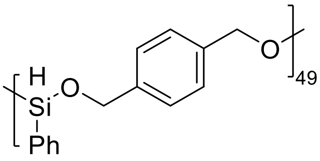

2c

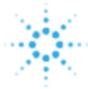

Agilent GPC/SEC Software
Sample GPC Analysis Report

MAJ1-118

Workspace Details

Workspace name	Polylactide
Location	C:\ProgramData\Agilent Technologies\GPC\Workspaces\Polylactide\
Comments	
Created by	Administrator at 13:44:31 on 15 June 2015

Sample Properties

Sample name	MAJ1-118
File name	ICF_29_10_2021-0026.sample
Collected by	GPC at 16:36:01 on 01 November 2021
Instrument name	Instrument 1

Column Calibration Details

Name	PSty July19th 2021
Created by	GPC at 14:33:05 on 19 July 2021
Last modified by	GPC at 14:35:07 on 19 July 2021
Comments	GPC Column Calibration created 19 July 2021 by GPC<br>GPC Column Calibration amended 19 July 2021 by GPC<br>GPC Column Calibration amended 19 July 2021 by GPC<br>GPC Column Calibration amended 19 July 2021 by GPC

Calibration Type	Narrow Standard	Curve Fit Used	3
Calibration Curve	y = - 0.00208x^3 + 0.09409x^2 - 1.779x + 16.51		

High Limit MW RT (mins)	11.20000	Low Limit MW RT (mins)	18.18333
High Limit MW (g/mol)	299400	Low Limit MW (g/mol)	580
Flow Rate Marker Name		Flow Marker RT (mins)	0.00000
K (Input) ((10e-5) dL/g)	14.100		
Alpha (Input)	0.700		
Residual Sum Of Squares	0.00159464	Corrected Sum Of Squares	7.33781
Coeff. Of Determination	0.999783	Standard Y Error Estimate	0.0163025
Linear Correlation Coeff	-0.999609		

Column Calibration Data Points

Point	Peak Max RT (mins)	MW	Log MW	Point in Use?	Percent Error
1	11.20000	299400	5.48	Yes	3.15
2	11.80000	151700	5.18	Yes	-3.99
3	12.68333	66350	4.82	Yes	-3.04
4	13.35000	38100	4.58	Yes	0.97
5	14.16667	19880	4.30	Yes	5.64
6	14.91667	9920	4.00	Yes	-1.11
7	15.76667	4920	3.69	Yes	-0.54
8	16.60000	2360	3.37	Yes	-3.39
9	17.36667	1260	3.10	Yes	1.56
10	18.18333	580	2.76	Yes	0.33

Analyst: .... **Date:** ....

Checked By: .... **Date:** ....

Agilent Technologies

Processing Parameters

Method	Last modified by Administrator at 13:44:30 on 15 June 2015
Using Flow Rate Correction	No
Mark-Houwink K ((10e-5) dL/g)	14.100
Mark-Houwink Alpha	0.700
Concentration Detector Used in Analysis	RI
Injection volume (µL)	100.00
Flow rate (mL/min)	1.00

MW Ranges Method

Calculate MW Ranges No

Percentage Fractions Method

Calculate Percentage Fractions No

Results

Analysed by GPC at 17:23:53 on 01 November 2021

Comments

Molecular Weight Averages

Peak	Mp (g/mol)	Mn (g/mol)	Mw (g/mol)	Mz (g/mol)	Mz+1 (g/mol)	Mv (g/mol)	PD
Peak 1	19565	11888	21852	38623	62796	35738	1.838

Peak Information

	Start (mins)	End (mins)
Baseline region 1	7.21667	7.81667
Baseline region 2	23.46667	23.81667
Peak 1	11.16667	16.23333

Peak Trace Information

Peak	Trace	Peak Max RT (mins)	Peak Area (mV.s)	Peak Height (mV)
Peak 1	RI	14.11667	1531.677	10.159
Peak 1	VS DP	13.78333	1247.253	8.913
Peak 1	VS IP	14.26667	36.640	0.603
Peak 1	LS 90°	13.63333	887.956	6.581
Peak 1	LS 15°	13.73333	271.238	2.050

Analyst: .... **Date:** ....

Checked By: .... **Date:** ....

Chromatogram Plot

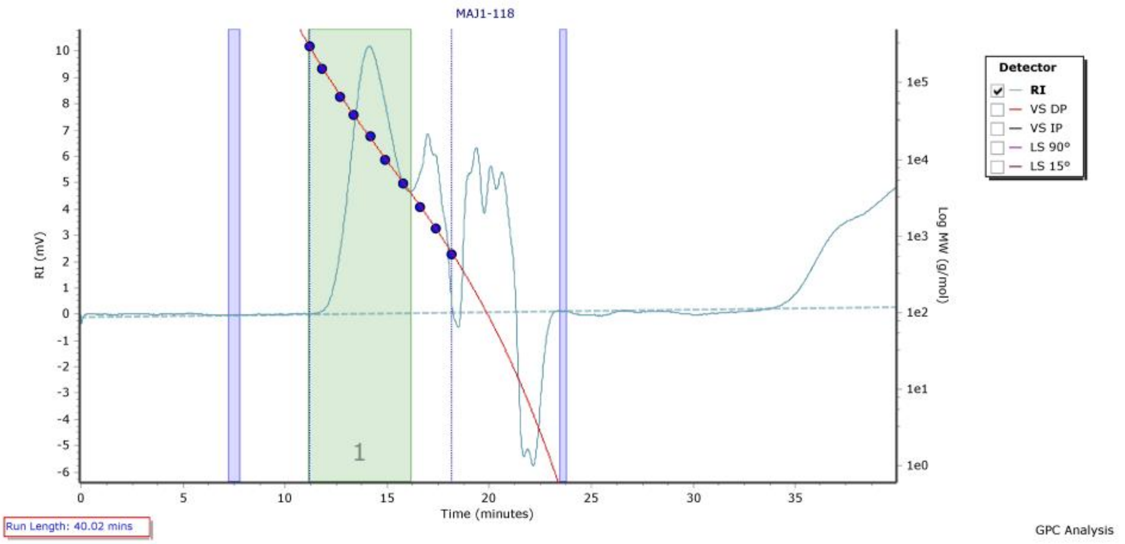

Analyst: .... **Date:** ....

Checked By: .... **Date:** ....

Agilent GPC/SEC Software
Sample GPC Analysis Report

Distribution Plot

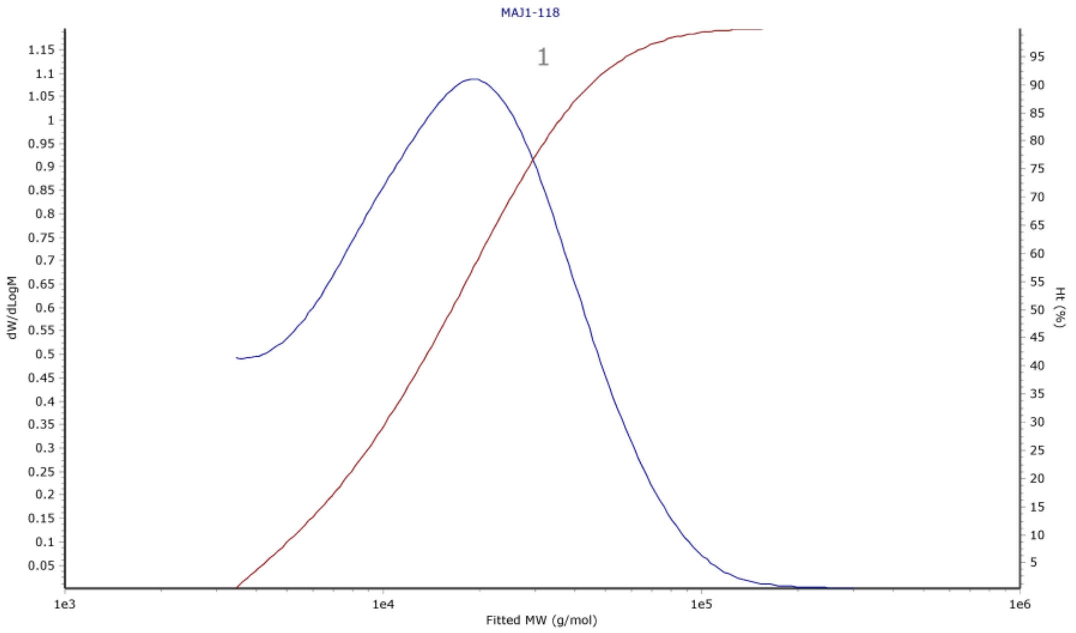

Analyst: .... **Date:** ....

Checked By: .... **Date:** ....

Agilent GPC/SEC Software A.02.01 [9] Page 4 of 4

Generated by GPC at 17:47 on 04 November 2021

Figure S42: GPC data of **2c**.

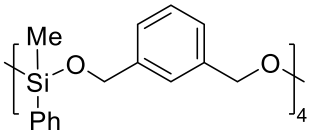

2d

Agilent GPC/SEC Software
Sample GPC Analysis Report

MAJ1-136_2

Workspace Details

Workspace name	Polylactide
Location	C:\ProgramData\Agilent Technologies\GPC\Workspaces\Polylactide\
Comments	
Created by	Administrator at 13:44:31 on 15 June 2015

Sample Properties

Sample name	MAJ1-136_2
File name	ICF_04_03_2022-0008.sample
Collected by	GPC at 12:03:07 on 05 March 2022
Instrument name	Instrument 1

Column Calibration Details

Name	PSty Jan 2022
Created by	GPC at 07:21:08 on 05 January 2022
Last modified by	GPC at 07:23:45 on 05 January 2022
Comments	GPC Column Calibration created 05 January 2022 by GPC<br>GPC Column Calibration amended 05 January 2022 by GPC<br>GPC Column Calibration amended 05 January 2022 by GPC<br>GPC Column Calibration amended 05 January 2022 by GPC

Calibration Type	Narrow Standard	Curve Fit Used	3
Calibration Curve	y = - 0.003181x^3 + 0.1419x^2 - 2.499x + 20.12		
High Limit MW RT (mins)	11.25000	Low Limit MW RT (mins)	17.58333
High Limit MW (g/mol)	283800	Low Limit MW (g/mol)	580
Flow Rate Marker Name		Flow Marker RT (mins)	0.00000
K (Input) ((10e-5) dL/g)	14.100		
Alpha (Input)	0.700		
Residual Sum Of Squares	0.00186199	Corrected Sum Of Squares	6.97134
Coeff. Of Determination	0.999733	Standard Y Error Estimate	0.0176162
Linear Correlation Coeff	-0.999415		

Column Calibration Data Points

Point	Peak Max RT (mins)	MW	Log MW	Point in Use?	Percent Error
1	11.25000	283800	5.45	Yes	1.92
2	11.85000	135700	5.13	Yes	-3.66
3	12.55000	67600	4.83	Yes	0.37
4	13.43333	29460	4.47	Yes	3.26
5	13.66667	22290	4.35	Yes	-2.84
6	14.65000	9820	3.99	Yes	4.37
7	15.35000	4910	3.69	Yes	-2.15
8	15.85000	3050	3.48	Yes	-4.49
9	16.88333	1250	3.10	Yes	4.07
10	17.58333	580	2.76	Yes	-1.35

Analyst: .... **Date:** ....

Checked By: .... **Date:** ....

Agilent GPC/SEC Software
Sample GPC Analysis Report

Agilent Technologies

Processing Parameters

Method	Last modified by Administrator at 13:44:30 on 15 June 2015
Using Flow Rate Correction	No
Mark-Houwink K ((10e-5) dL/g)	14.100
Mark-Houwink Alpha	0.700
Concentration Detector Used in Analysis	RI
Injection volume (µL)	100.00
Flow rate (mL/min)	1.00

MW Ranges Method

Calculate MW Ranges	No

Percentage Fractions Method

Calculate Percentage Fractions	No

Results

Analysed by	GPC at 12:44:09 on 05 March 2022
Comments	

Molecular Weight Averages

Peak	Mp (g/mol)	Mn (g/mol)	Mw (g/mol)	Mz (g/mol)	Mz+1 (g/mol)	Mv (g/mol)	PD
Peak 1	874	1256	1825	5211	21990	4074	1.453

Peak Information

	Start (mins)	End (mins)
Baseline region 1	7.58333	8.23333
Baseline region 2	30.50000	33.03333
Peak 1	12.73333	17.50000

Peak Trace Information

Peak	Trace	Peak Max RT (mins)	Peak Area (mV.s)	Peak Height (mV)
Peak 1	RI	17.20000	1933.806	26.586
Peak 1	VS DP	17.18333	730.710	6.686
Peak 1	VS IP	16.53333	85.577	0.901
Peak 1	LS 90°	16.78333	116.795	1.114
Peak 1	LS 15°	16.35000	38.558	0.318

Analyst: .... **Date:** ....

Checked By: .... **Date:** ....

Agilent GPC/SEC Software
Sample GPC Analysis Report

Agilent Technologies

Chromatogram Plot

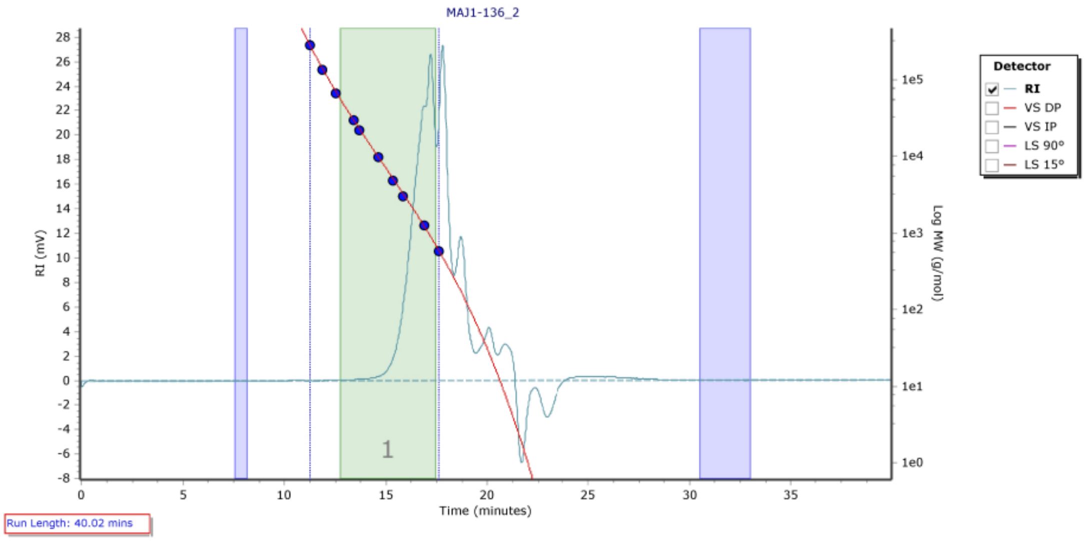

Analyst: .... **Date:** ....

Checked By: .... **Date:** ....

Agilent GPC/SEC Software
Sample GPC Analysis Report

Agilent Technologies

Distribution Plot

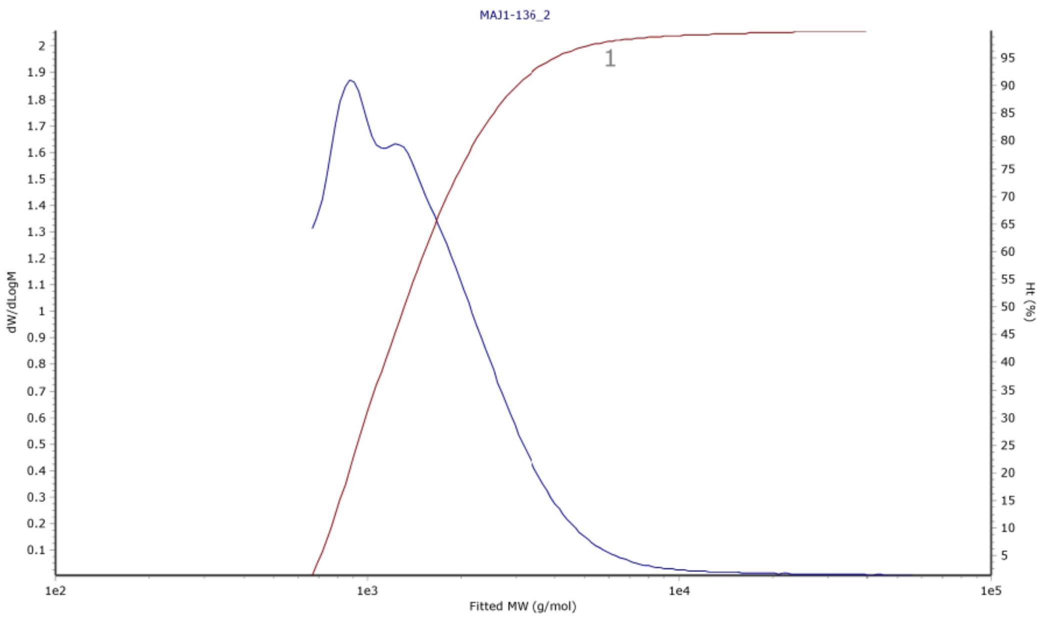

Analyst: .... **Date:** ....

Checked By: .... **Date:** ....

Agilent GPC/SEC Software A.02.01 [9] Page 4 of 4

Generated by GPC at 12:44 on 05 March 2022

Figure S43: GPC data of **2d**.

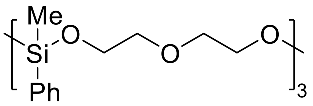

2f

Agilent GPC/SEC Software
Sample GPC Analysis Report

Agilent Technologies

MAJ1-119_3

Workspace Details

Workspace name	Polylactide
Location	C:\ProgramData\Agilent Technologies\GPC\Workspaces\Polylactide\
Comments	
Created by	Administrator at 13:44:31 on 15 June 2015

Sample Properties

Sample name	MAJ1-119_3
File name	ICF_04_11_2021-0020.sample
Collected by	GPC at 05:38:21 on 05 November 2021
Instrument name	Instrument 1

Column Calibration Details

Name	PSty 7thJune2021
Created by	GPC at 10:31:14 on 08 June 2021
Last modified by	GPC at 10:33:07 on 08 June 2021
Comments	GPC Column Calibration created 08 June 2021 by GPC<br>GPC Column Calibration amended 08 June 2021 by GPC<br>GPC Column Calibration amended 08 June 2021 by GPC<br>GPC Column Calibration amended 08 June 2021 by GPC

Calibration Type	Narrow Standard	Curve Fit Used	3
Calibration Curve	y = - 0.002194x^3 + 0.09598x^2 - 1.75x + 16.01		
High Limit MW RT (mins)	10.96667	Low Limit MW RT (mins)	18.08333
High Limit MW (g/mol)	299400	Low Limit MW (g/mol)	580
Flow Rate Marker Name		Flow Marker RT (mins)	0.00000
K (Input) ((10e-5) dL/g)	14.100		
Alpha (Input)	0.700		
Residual Sum Of Squares	0.00194182	Corrected Sum Of Squares	7.33781
Coeff. Of Determination	0.999735	Standard Y Error Estimate	0.0179899
Linear Correlation Coeff	-0.99962		

Column Calibration Data Points

Point	Peak Max RT (mins)	MW	Log MW	Point in Use?	Percent Error
1	10.96667	299400	5.48	Yes	4.04
2	11.56667	151700	5.18	Yes	-5.48
3	12.50000	66350	4.82	Yes	-3.34
4	13.21667	38100	4.58	Yes	2.43
5	14.03333	19880	4.30	Yes	4.54
6	14.83333	9920	4.00	Yes	-0.09
7	15.68333	4920	3.69	Yes	-0.81
8	16.51667	2360	3.37	Yes	-3.97
9	17.28333	1260	3.10	Yes	1.65
10	18.08333	580	2.76	Yes	0.52

Analyst: .... **Date:** ....

Checked By: .... **Date:** ....

Agilent GPC/SEC Software
Sample GPC Analysis Report

Agilent Technologies

Processing Parameters

Method	Last modified by Administrator at 13:44:30 on 15 June 2015
Using Flow Rate Correction	No
Mark-Houwink K ((10e-5) dL/g)	14.100
Mark-Houwink Alpha	0.700
Concentration Detector Used in Analysis	RI
Injection volume (µL)	100.00
Flow rate (mL/min)	1.00

MW Ranges Method

Calculate MW Ranges   No

Percentage Fractions Method

Calculate Percentage Fractions   No

Results

Analysed by   GPC at 16:36:21 on 05 November 2021
Comments

Molecular Weight Averages

Peak	Mp (g/mol)	Mn (g/mol)	Mw (g/mol)	Mz (g/mol)	Mz+1 (g/mol)	Mv (g/mol)	PD
Peak 1	1278	885	1328	2034	3122	1907	1.501

Peak Information

	Start (mins)	End (mins)
Baseline region 1	9.83333	10.55000
Baseline region 2	31.71667	32.73333
Peak 1	14.61667	18.83333

Peak Trace Information

Peak	Trace	Peak Max RT (mins)	Peak Area (mV.s)	Peak Height (mV)
Peak 1	RI	17.26667	1603.479	15.582
Peak 1	VS DP	17.26667	638.377	5.063
Peak 1	VS IP	16.63333	7.180	-0.451
Peak 1	LS 90°	17.26667	37.639	0.333
Peak 1	LS 15°	17.98333	4.461	-0.066

Analyst: .... **Date:** ....

Checked By: .... **Date:** ....

Agilent GPC/SEC Software
Sample GPC Analysis Report

Agilent Technologies

Chromatogram Plot

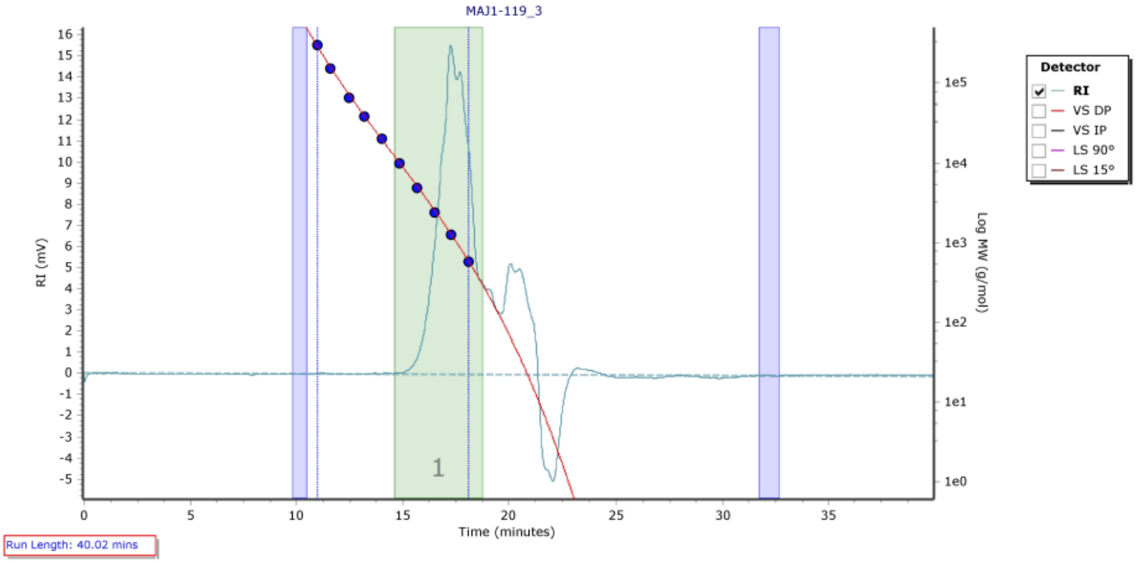

Analyst: .... **Date:** ....

Checked By: .... **Date:** ....

Agilent GPC/SEC Software
Sample GPC Analysis Report

Agilent Technologies

Distribution Plot

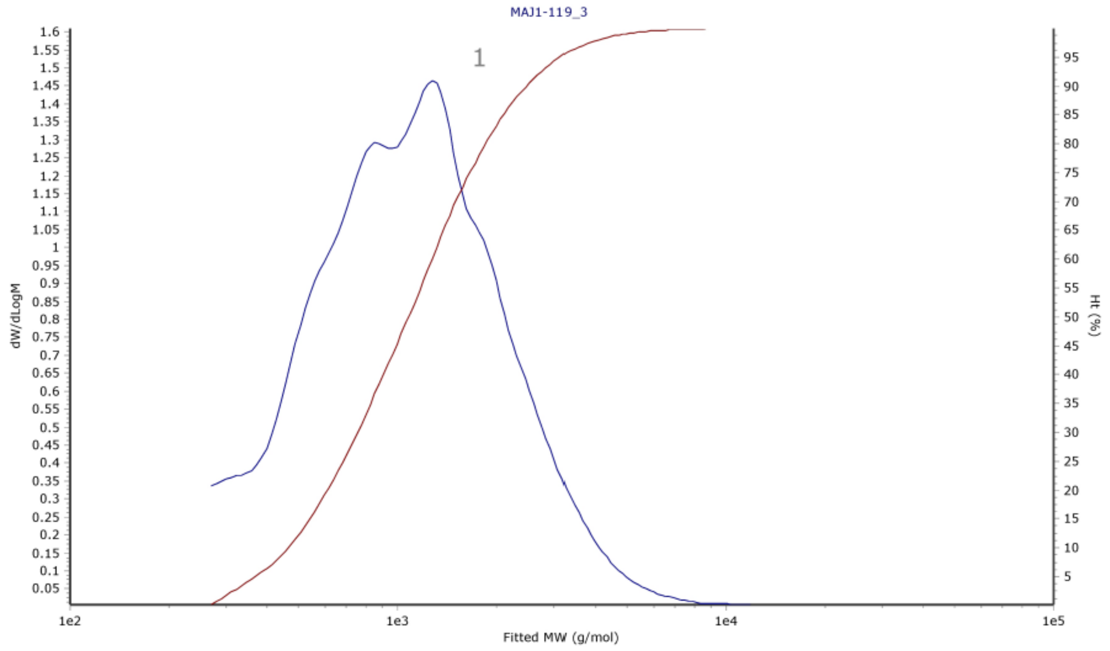

Analyst: .... **Date:** ....

Checked By: .... **Date:** ....

Agilent GPC/SEC Software A.02.01 [9] Page **4** of 4

Generated by GPC at 16:36 on 05 November 2021

Figure S44: GPC data of **2f**.

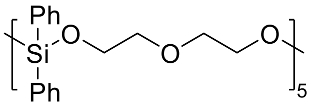

2g

Agilent GPC/SEC Software
Sample GPC Analysis Report

Agilent Technologies

MAJ1-119_2

Workspace Details

Workspace name	Polylactide
Location	C:\ProgramData\Agilent Technologies\GPC\Workspaces\Polylactide\
Comments	
Created by	Administrator at 13:44:31 on 15 June 2015

Sample Properties

Sample name	MAJ1-119_2
File name	ICF_04_11_2021-0019.sample
Collected by	GPC at 04:56:26 on 05 November 2021
Instrument name	Instrument 1

Column Calibration Details

Name	PSty 7thJune2021
Created by	GPC at 10:31:14 on 08 June 2021
Last modified by	GPC at 10:33:07 on 08 June 2021
Comments	GPC Column Calibration created 08 June 2021 by GPC<br>GPC Column Calibration amended 08 June 2021 by GPC<br>GPC Column Calibration amended 08 June 2021 by GPC<br>GPC Column Calibration amended 08 June 2021 by GPC

Calibration Type	Narrow Standard	Curve Fit Used	3
Calibration Curve	y = - 0.002194x^3 + 0.09598x^2 - 1.75x + 16.01		
High Limit MW RT (mins)	10.96667	Low Limit MW RT (mins)	18.08333
High Limit MW (g/mol)	299400	Low Limit MW (g/mol)	580
Flow Rate Marker Name		Flow Marker RT (mins)	0.00000
K (Input) ((10e-5) dL/g)	14.100		
Alpha (Input)	0.700		
Residual Sum Of Squares	0.00194182	Corrected Sum Of Squares	7.33781
Coeff. Of Determination	0.999735	Standard Y Error Estimate	0.0179899
Linear Correlation Coeff	-0.99962		

Column Calibration Data Points

Point	Peak Max RT (mins)	MW	Log MW	Point in Use?	Percent Error
1	10.96667	299400	5.48	Yes	4.04
2	11.56667	151700	5.18	Yes	-5.48
3	12.50000	66350	4.82	Yes	-3.34
4	13.21667	38100	4.58	Yes	2.43
5	14.03333	19880	4.30	Yes	4.54
6	14.83333	9920	4.00	Yes	-0.09
7	15.68333	4920	3.69	Yes	-0.81
8	16.51667	2360	3.37	Yes	-3.97
9	17.28333	1260	3.10	Yes	1.65
10	18.08333	580	2.76	Yes	0.52

Analyst: .... **Date:** ....

Checked By: .... **Date:** ....

Agilent GPC/SEC Software
Sample GPC Analysis Report

Agilent Technologies

Processing Parameters

Method	Last modified by Administrator at 13:44:30 on 15 June 2015
Using Flow Rate Correction	No
Mark-Houwink K ((10e-5) dL/g)	14.100
Mark-Houwink Alpha	0.700
Concentration Detector Used in Analysis	RI
Injection volume (µL)	100.00
Flow rate (mL/min)	1.00

MW Ranges Method

Calculate MW Ranges No

Percentage Fractions Method

Calculate Percentage Fractions No

Results

Analysed by GPC at 16:34:55 on 05 November 2021

Comments

Molecular Weight Averages

Peak	Mp (g/mol)	Mn (g/mol)	Mw (g/mol)	Mz (g/mol)	Mz+1 (g/mol)	Mv (g/mol)	PD
Peak 1	2061	1588	2315	3532	5873	3293	1.458

Peak Information

	Start (mins)	End (mins)
Baseline region 1	7.76667	8.43333
Baseline region 2	32.75000	33.88333
Peak 1	13.75000	18.36667

Peak Trace Information

Peak	Trace	Peak Max RT (mins)	Peak Area (mV.s)	Peak Height (mV)
Peak 1	RI	16.73333	1625.139	15.561
Peak 1	VS DP	16.70000	605.847	4.958
Peak 1	VS IP	15.90000	16.627	0.465
Peak 1	LS 90°	16.43333	106.891	0.869
Peak 1	LS 15°	16.11667	20.840	0.174

Analyst: .... **Date:** ....

Checked By: .... **Date:** ....

Agilent GPC/SEC Software
Sample GPC Analysis Report

Agilent Technologies

Chromatogram Plot

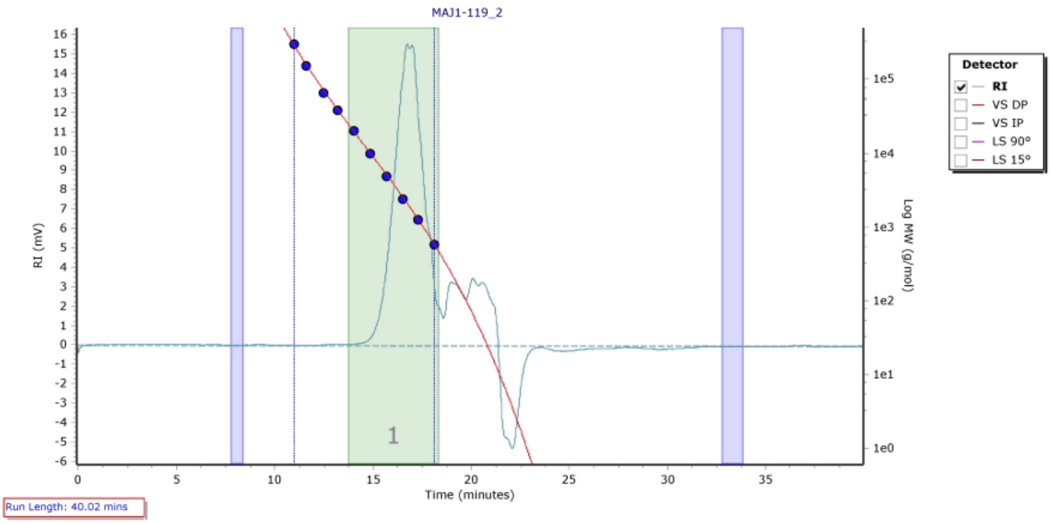

Analyst: .... **Date:** ....

Checked By: .... **Date:** ....

Agilent GPC/SEC Software
Sample GPC Analysis Report

Agilent Technologies

Distribution Plot

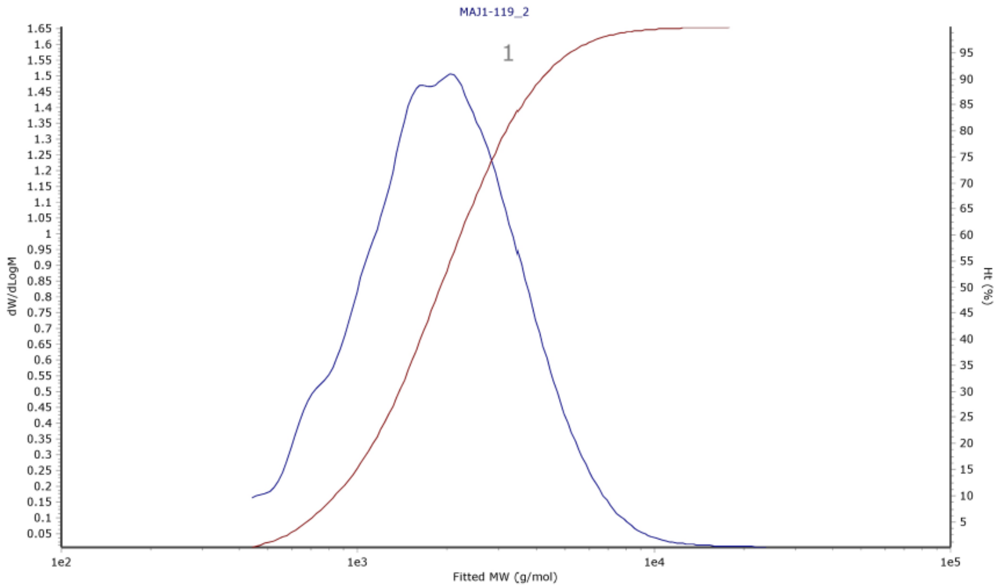

Analyst: .... **Date:** ....

Checked By: .... **Date:** ....

Agilent GPC/SEC Software A.02.01 [9] Page 4 of 4 Generated by GPC at 16:35 on 05 November 2021

Figure S45: GPC data of **2g**.

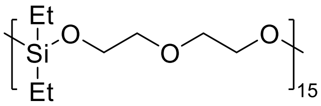

2h

Agilent GPC/SEC Software
Sample GPC Analysis Report

Agilent Technologies

MAJ1-183 PS only

Workspace Details

Workspace name	Polylactide
Location	C:\ProgramData\Agilent Technologies\GPC\Workspaces\Polylactide\
Comments	
Created by	Administrator at 13:44:31 on 15 June 2015

Sample Properties

Sample name	MAJ1-183 PS only
File name	ICF_22_06_2022-0006.sample
Collected by	GPC at 15:19:30 on 22 June 2022
Instrument name	Instrument 1

Column Calibration Details

Name	PSty26thMay2022
Created by	GPC at 14:09:29 on 26 May 2022
Last modified by	GPC at 14:10:44 on 26 May 2022
Comments	GPC Column Calibration created 26 May 2022 by GPC<br>GPC Column Calibration amended 26 May 2022 by GPC<br>GPC Column Calibration amended 26 May 2022 by GPC<br>GPC Column Calibration amended 26 May 2022 by GPC<br>GPC Column Calibration amended 26 May 2022 by GPC

Calibration Type	Narrow Standard	Curve Fit Used	3
Calibration Curve	y = - 0.001896x^3 + 0.08659x^2 - 1.684x + 16.11		
High Limit MW RT (mins)	11.21667	Low Limit MW RT (mins)	18.08333
High Limit MW (g/mol)	283800	Low Limit MW (g/mol)	580
Flow Rate Marker Name		Flow Marker RT (mins)	0.00000
K (Input) ((10e-5) dL/g)	14.100		
Alpha (Input)	0.700		
Residual Sum Of Squares	0.00330604	Corrected Sum Of Squares	8.92203
Coeff. Of Determination	0.999629	Standard Y Error Estimate	0.0191661
Linear Correlation Coeff	-0.999452		

Column Calibration Data Points

Point	Peak Max RT (mins)	MW	Log MW	Point in Use?	Percent Error
1	11.21667	283800	5.45	Yes	2.59
2	11.21667	283800	5.45	Yes	2.59
3	11.83333	135700	5.13	Yes	-8.47
4	12.63333	67600	4.83	Yes	-0.98
5	13.58333	29480	4.47	Yes	1.73
6	13.58333	29480	4.47	Yes	1.73
7	13.88333	22290	4.35	Yes	-0.05
8	14.93333	9820	3.99	Yes	7.23
9	15.63333	4910	3.69	Yes	-2.81
10	15.63333	4910	3.69	Yes	-2.81
11	16.18333	3050	3.48	Yes	-3.74
12	17.26667	1250	3.10	Yes	1.77
13	18.08333	580	2.76	Yes	0.33

Analyst: .... **Date:** ....

Checked By: .... **Date:** ....

Agilent GPC/SEC Software
Sample GPC Analysis Report

Agilent Technologies

Processing Parameters

Method	Last modified by Administrator at 13:44:30 on 15 June 2015
Using Flow Rate Correction	No
Mark-Houwink K ((10e-5) dL/g)	14.100
Mark-Houwink Alpha	0.700
Concentration Detector Used in Analysis	RI
Injection volume (µL)	100.00
Flow rate (mL/min)	1.00

MW Ranges Method

Calculate MW Ranges	No

Percentage Fractions Method

Calculate Percentage Fractions	No

Results

Analysed by	GPC at 16:49:18 on 23 June 2022
Comments	

Molecular Weight Averages

Peak	Mp (g/mol)	Mn (g/mol)	Mw (g/mol)	Mz (g/mol)	Mz+1 (g/mol)	Mv (g/mol)	PD
Peak 1	6322	3183	6788	11059	15053	10470	2.133

Peak Information

	Start (mins)	End (mins)
Baseline region 1	8.75000	9.28333
Baseline region 2	29.13333	29.73333
Peak 1	13.40000	18.05000

Peak Trace Information

Peak	Trace	Peak Max RT (mins)	Peak Area (mV.s)	Peak Height (mV)
Peak 1	RI	15.36667	229.736	1.575
Peak 1	VS DP	14.95000	647.003	4.213
Peak 1	VS IP	17.73333	10.244	-0.323
Peak 1	LS 90°	14.68333	10.134	0.168
Peak 1	LS 15°	14.18333	1.731	0.164

Analyst: .... **Date:** ....

Checked By: .... **Date:** ....

Chromatogram Plot

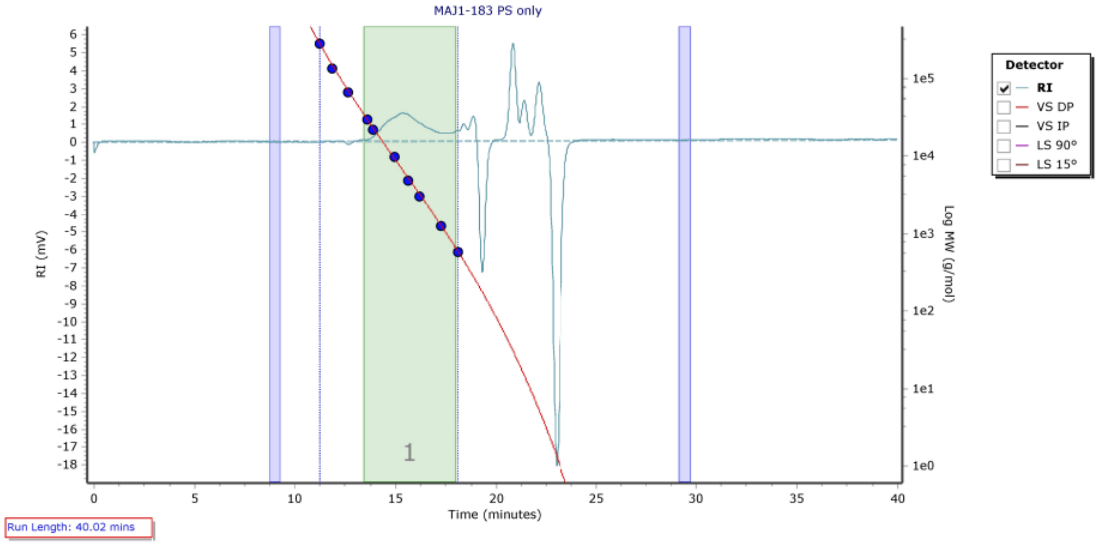

Analyst: .... **Date:** ....

Checked By: .... **Date:** ....

Agilent GPC/SEC Software
Sample GPC Analysis Report

Agilent Technologies

Distribution Plot

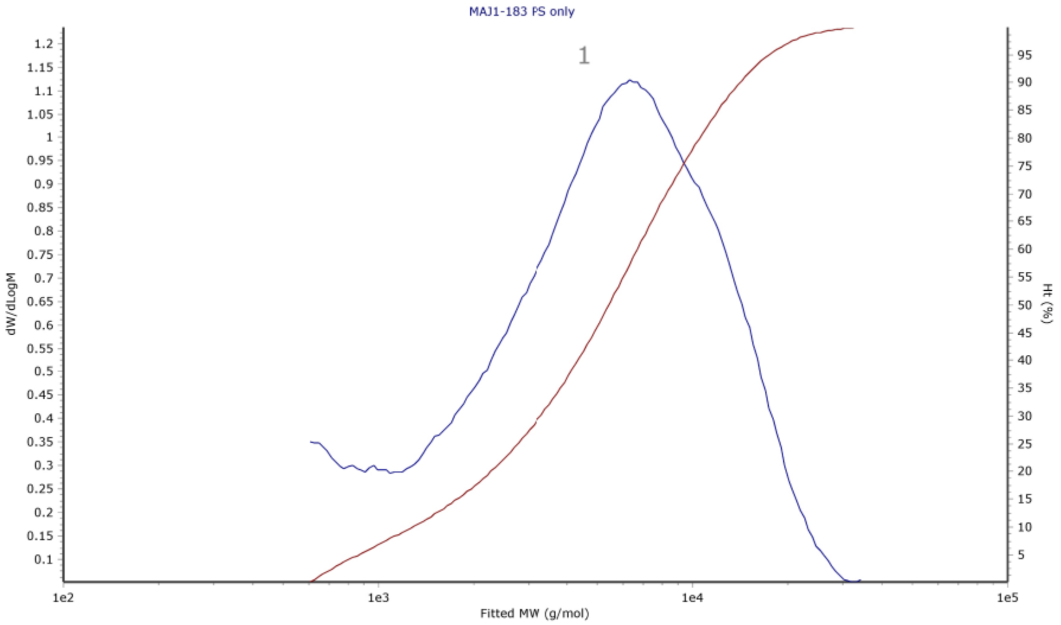

Analyst: .... **Date:** ....

Checked By: .... **Date:** ....

Agilent GPC/SEC Software A.02.01 [9] Page 4 of 4

Generated by GPC at 16:49 on 23 June 2022

Figure S46: GPC data of **2h**.

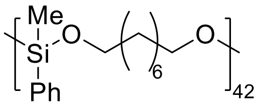

2j

Agilent GPC/SEC Software
Sample GPC Analysis Report

Agilent Technologies

MAJ1-146_2

Workspace Details

Workspace name	Polylactide
Location	C:\ProgramData\Agilent Technologies\GPC\Workspaces\Polylactide\
Comments	
Created by	Administrator at 13:44:31 on 15 June 2015

Sample Properties

Sample name	MAJ1-146_2
File name	ICF_07_03_2022-0040.sample
Collected by	GPC at 21:00:55 on 08 March 2022
Instrument name	Instrument 1

Column Calibration Details

Name	2021-08-05-001
Created by	GPC at 15:56:03 on 05 August 2021
Last modified by	GPC at 15:57:44 on 05 August 2021
Comments	PMMA 5thAugust 2021<br>GPC Column Calibration amended 05 August 2021 by GPC<br>GPC Column Calibration amended 05 August 2021 by GPC

Calibration Type	Narrow Standard	Curve Fit Used	3
Calibration Curve	y = - 0.005181x^3 + 0.2282x^2 - 3.682x + 25.39		
High Limit MW RT (mins)	11.41667	Low Limit MW RT (mins)	17.76667
High Limit MW (g/mol)	260900	Low Limit MW (g/mol)	885
Flow Rate Marker Name		Flow Marker RT (mins)	0.00000
K (Input) ((10e-5) dL/g)	14.100		
Alpha (Input)	0.700		
Residual Sum Of Squares	0.00718227	Corrected Sum Of Squares	5.70449
Coeff. Of Determination	0.998741	Standard Y Error Estimate	0.0423741
Linear Correlation Coeff	-0.998685		

Column Calibration Data Points

Point	Peak Max RT (mins)	MW	Log MW	Point in Use?	Percent Error
1	11.41667	260900	5.42	Yes	4.15
2	11.85000	146500	5.17	Yes	-6.25
3	12.60000	72800	4.86	Yes	-2.64
4	13.78333	30780	4.49	Yes	10.61
5	15.10000	9150	3.96	Yes	-9.45
6	16.01667	4760	3.68	Yes	-1.96
7	17.20000	1840	3.26	Yes	8.76
8	17.76667	885	2.95	Yes	-5.12

Analyst: .... **Date:** ....

Checked By: .... **Date:** ....

Agilent GPC/SEC Software
Sample GPC Analysis Report

Agilent Technologies

Processing Parameters

Method	Last modified by Administrator at 13:44:30 on 15 June 2015
Using Flow Rate Correction	No
Mark-Houwink K ((10e-5) dL/g)	14.100
Mark-Houwink Alpha	0.700
Concentration Detector Used in Analysis	RI
Injection volume (µL)	100.00
Flow rate (mL/min)	1.00

MW Ranges Method

Calculate MW Ranges	No

Percentage Fractions Method

Calculate Percentage Fractions	No

Results

Analysed by	GPC at 11:03:38 on 09 March 2022
Comments	

Molecular Weight Averages

Peak	Mp (g/mol)	Mn (g/mol)	Mw (g/mol)	Mz (g/mol)	Mz+1 (g/mol)	Mv (g/mol)	PD
Peak 1	19147	10579	24727	70137	205435	58609	2.337

Peak Information

	Start (mins)	End (mins)
Baseline region 1	3.93333	4.65000
Baseline region 2	32.55000	33.16667
Peak 1	10.80000	16.95000

Peak Trace Information

Peak	Trace	Peak Max RT (mins)	Peak Area (mV.s)	Peak Height (mV)
Peak 1	RI	14.26667	941.093	5.335
Peak 1	VS DP	13.63333	1718.714	10.438
Peak 1	VS IP	16.86667	140.586	-0.947
Peak 1	LS 90°	14.08333	241.029	4.627
Peak 1	LS 15°	16.56667	56.818	2.227

Analyst: .... **Date:** ....

Checked By: .... **Date:** ....

Agilent GPC/SEC Software
Sample GPC Analysis Report

Agilent Technologies

Chromatogram Plot

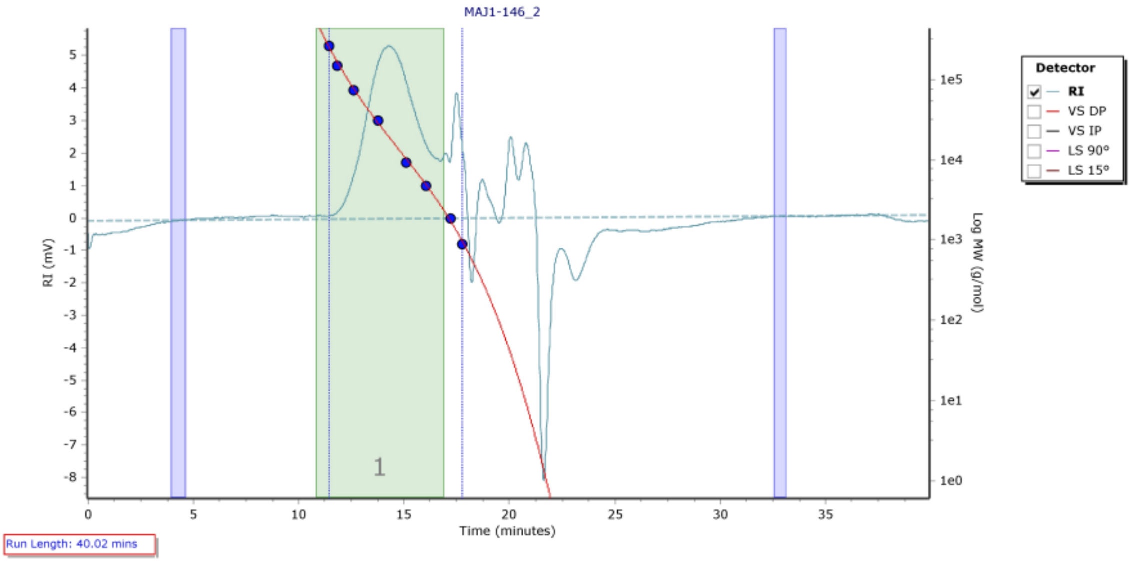

Analyst: .... **Date:** ....

Checked By: .... **Date:** ....

Agilent GPC/SEC Software
Sample GPC Analysis Report

Agilent Technologies

Distribution Plot

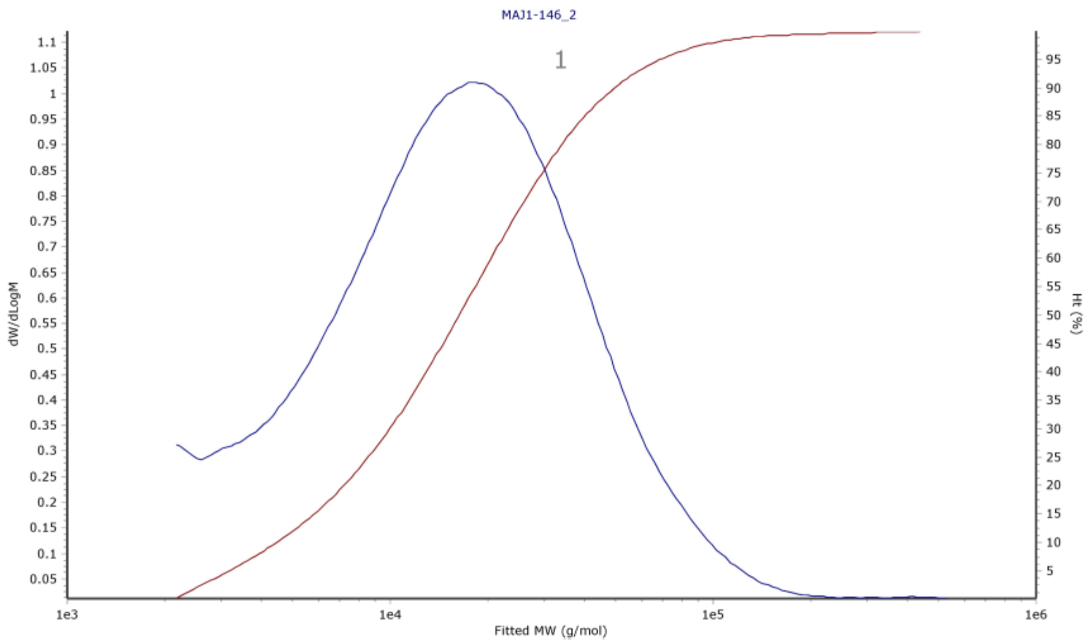

Analyst: .... **Date:** ....

Checked By: .... **Date:** ....

Agilent GPC/SEC Software A.02.01 [9] Page 4 of 4

Generated by GPC at 11:03 on 09 March 2022

Figure S47: GPC data of **2j**.

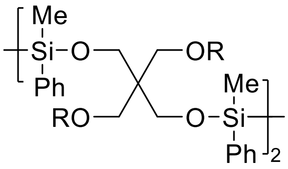

R = -H, -Si(MePh)-

2l

Agilent GPC/SEC Software
Sample GPC Analysis Report

Agilent Technologies

MAJ1-133_2

Workspace Details

Workspace name	Polylactide
Location	C:\ProgramData\Agilent Technologies\GPC\Workspaces\Polylactide\
Comments	
Created by	Administrator at 13:44:31 on 15 June 2015

Sample Properties

Sample name	MAJ1-133_2
File name	ICF_16_12_2021-0029-1.sample
Collected by	GPC at 15:01:44 on 17 December 2021
Instrument name	Instrument 1

Column Calibration Details

Name	PSty May2022
Created by	GPC at 15:34:51 on 28 April 2022
Last modified by	GPC at 16:20:40 on 28 April 2022
Comments	GPC Column Calibration created 28 April 2022 by GPC<br>GPC Column Calibration amended 28 April 2022 by GPC<br>GPC Column Calibration amended 28 April 2022 by GPC<br>GPC Column Calibration amended 28 April 2022 by GPC

Calibration Type	Narrow Standard	Curve Fit Used	3
Calibration Curve	y = - 0.002611x^3 + 0.118x^2 - 2.153x + 18.62		
High Limit MW RT (mins)	11.58333	Low Limit MW RT (mins)	18.21667
High Limit MW (g/mol)	283800	Low Limit MW (g/mol)	580
Flow Rate Marker Name		Flow Marker RT (mins)	0.00000
K (Input) ((10e-5) dL/g)	14.100		
Alpha (Input)	0.700		
Residual Sum Of Squares	0.00281894	Corrected Sum Of Squares	6.95434
Coeff. Of Determination	0.999595	Standard Y Error Estimate	0.0216754
Linear Correlation Coeff	-0.999538		

Column Calibration Data Points

Point	Peak Max RT (mins)	MW	Log MW	Point in Use?	Percent Error
1	11.58333	283800	5.45	Yes	0.76
2	12.28333	135700	5.13	Yes	0.05
3	12.98333	67600	4.83	Yes	-1.47
4	13.90000	28480	4.45	Yes	-4.07
5	14.25000	22290	4.35	Yes	2.44
6	15.26667	9820	3.99	Yes	8.59
7	15.88333	4910	3.69	Yes	-6.74
8	16.48333	3050	3.48	Yes	-0.71
9	17.43333	1260	3.10	Yes	-0.41
10	18.21667	580	2.76	Yes	0.83

Analyst: .... **Date:** ....

Checked By: .... **Date:** ....

Agilent GPC/SEC Software
Sample GPC Analysis Report

Agilent Technologies

Processing Parameters

Method	Last modified by Administrator at 13:44:30 on 15 June 2015
Using Flow Rate Correction	No
Mark-Houwink K ((10e-5) dL/g)	14.100
Mark-Houwink Alpha	0.700
Concentration Detector Used in Analysis	RI
Injection volume (µL)	100.00
Flow rate (mL/min)	1.00

MW Ranges Method

Calculate MW Ranges	No

Percentage Fractions Method

Calculate Percentage Fractions	No

Results

Analysed by	GPC at 09:28:28 on 16 May 2022
Comments	

Molecular Weight Averages

Peak	Mp (g/mol)	Mn (g/mol)	Mw (g/mol)	Mz (g/mol)	Mz+1 (g/mol)	Mv (g/mol)	PD
Peak 1	770	606	1148	2894	6692	2491	1.894

Peak Information

	Start (mins)	End (mins)
Baseline region 1	8.51667	9.11667
Baseline region 2	30.11667	30.96667
Peak 1	14.55000	19.58333

Peak Trace Information

Peak	Trace	Peak Max RT (mins)	Peak Area (mV.s)	Peak Height (mV)
Peak 1	RI	17.95000	1213.462	15.693
Peak 1	VS DP	17.98333	365.192	3.614
Peak 1	VS IP	16.45000	9.006	0.407
Peak 1	LS 90°	17.90000	21.660	0.227
Peak 1	LS 15°	15.21667	27.538	-0.139

Analyst: .... **Date:** ....

Checked By: .... **Date:** ....

Agilent GPC/SEC Software
Sample GPC Analysis Report

Agilent Technologies

Chromatogram Plot

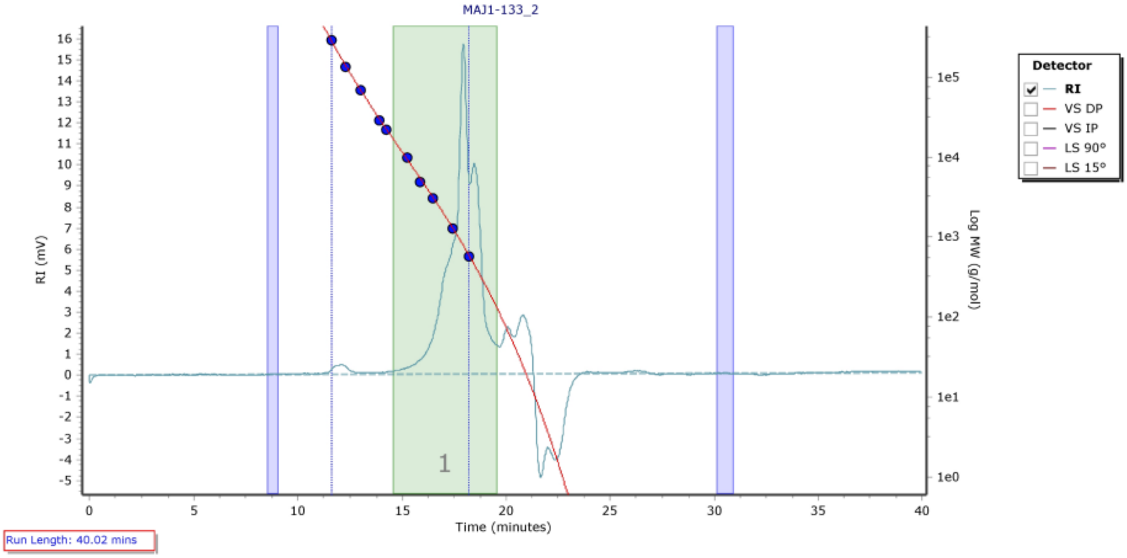

Analyst: .... **Date:** ....

Checked By: .... **Date:** ....

Agilent GPC/SEC Software
Sample GPC Analysis Report

Agilent Technologies

Distribution Plot

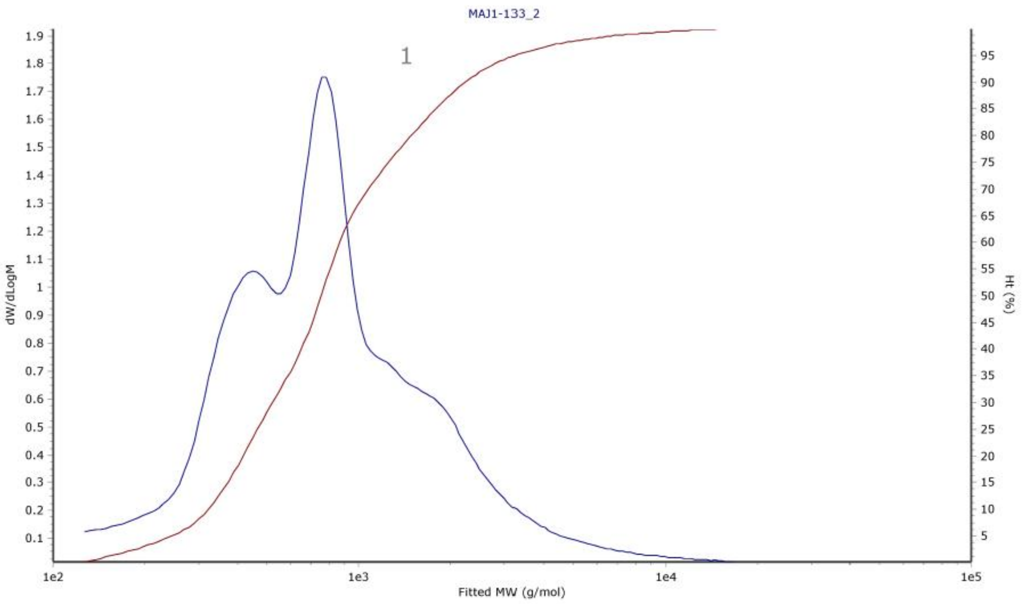

Analyst: .... **Date:** ....

Checked By: .... **Date:** ....

Agilent GPC/SEC Software A.02.01 [9] Page 4 of 4

Generated by GPC at 09:28 on 16 May 2022

Figure S48: GPC data of **2l**.

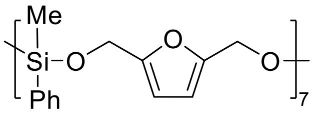

2n

Agilent GPC/SEC Software
Sample GPC Analysis Report

Agilent Technologies

MAJ1-184 PI

Workspace Details

Workspace name	Polylactide
Location	C:\ProgramData\Agilent Technologies\GPC\Workspaces\Polylactide\
Comments	
Created by	Administrator at 13:44:31 on 15 June 2015

Sample Properties

Sample name	MAJ1-184 PI
File name	ICF_22_06_2022-0007.sample
Collected by	GPC at 16:01:24 on 22 June 2022
Instrument name	Instrument 1

Column Calibration Details

Name	PSty26thMay2022
Created by	GPC at 14:09:29 on 26 May 2022
Last modified by	GPC at 14:10:44 on 26 May 2022
Comments	GPC Column Calibration created 26 May 2022 by GPC<br>GPC Column Calibration amended 26 May 2022 by GPC<br>GPC Column Calibration amended 26 May 2022 by GPC<br>GPC Column Calibration amended 26 May 2022 by GPC<br>GPC Column Calibration amended 26 May 2022 by GPC

Calibration Type	Narrow Standard	Curve Fit Used	3
Calibration Curve	y = - 0.001896x^3 + 0.08659x^2 - 1.684x + 16.11		
High Limit MW RT (mins)	11.21667	Low Limit MW RT (mins)	18.08333
High Limit MW (g/mol)	283800	Low Limit MW (g/mol)	580
Flow Rate Marker Name		Flow Marker RT (mins)	0.00000
K (Input) ((10e-5) dL/g)	14.100		
Alpha (Input)	0.700		
Residual Sum Of Squares	0.00330604	Corrected Sum Of Squares	8.92203
Coeff. Of Determination	0.999629	Standard Y Error Estimate	0.0191661
Linear Correlation Coeff	-0.999452		

Column Calibration Data Points

Point	Peak Max RT (mins)	MW	Log MW	Point in Use?	Percent Error
1	11.21667	283800	5.45	Yes	2.59
2	11.21667	283800	5.45	Yes	2.59
3	11.83333	135700	5.13	Yes	-8.47
4	12.63333	67600	4.83	Yes	-0.98
5	13.58333	29480	4.47	Yes	1.73
6	13.58333	29480	4.47	Yes	1.73
7	13.88333	22290	4.35	Yes	-0.05
8	14.93333	9820	3.99	Yes	7.23
9	15.63333	4910	3.69	Yes	-2.81
10	15.63333	4910	3.69	Yes	-2.81
11	16.18333	3050	3.48	Yes	-3.74
12	17.26667	1250	3.10	Yes	1.77
13	18.08333	580	2.76	Yes	0.33

Analyst: .... **Date:** ....

Checked By: .... **Date:** ....

Agilent GPC/SEC Software
Sample GPC Analysis Report

Agilent Technologies

Processing Parameters

Method	Last modified by Administrator at 13:44:30 on 15 June 2015
Using Flow Rate Correction	No
Mark-Houwink K ((10e-5) dL/g)	14.100
Mark-Houwink Alpha	0.700
Concentration Detector Used in Analysis	RI
Injection volume (µL)	100.00
Flow rate (mL/min)	1.00

MW Ranges Method

Calculate MW Ranges No

Percentage Fractions Method

Calculate Percentage Fractions No

Results

Analysed by GPC at 16:50:36 on 23 June 2022

Comments

Molecular Weight Averages

Peak	Mp (g/mol)	Mn (g/mol)	Mw (g/mol)	Mz (g/mol)	Mz+1 (g/mol)	Mv (g/mol)	PD
Peak 1	2179	1775	2948	4985	7961	4617	1.661

Peak Information

	Start (mins)	End (mins)
Baseline region 1	2.90000	3.61667
Baseline region 2	32.86667	33.78333
Peak 1	13.71667	18.43333

Peak Trace Information

Peak	Trace	Peak Max RT (mins)	Peak Area (mV.s)	Peak Height (mV)
Peak 1	RI	16.61667	372.756	3.088
Peak 1	VS DP	16.43333	185.073	1.345
Peak 1	VS IP	16.80000	12.073	0.187
Peak 1	LS 90°	15.88333	40.840	0.374
Peak 1	LS 15°	15.88333	40.945	0.243

Analyst: .... **Date:** ....

Checked By: .... **Date:** ....

Agilent GPC/SEC Software
Sample GPC Analysis Report

Agilent Technologies

Chromatogram Plot

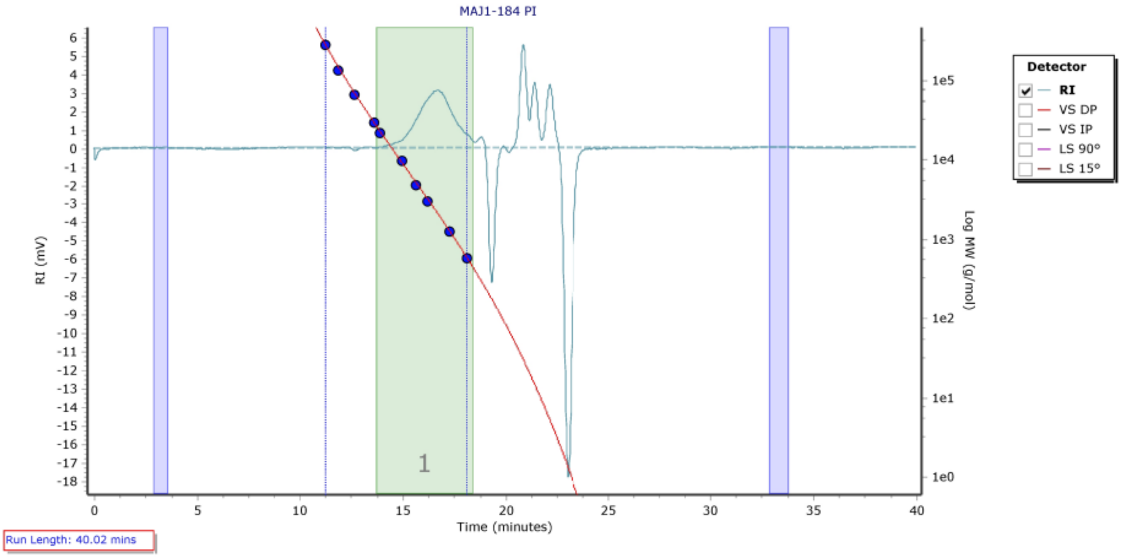

Analyst: .... **Date:** ....

Checked By: .... **Date:** ....

Agilent GPC/SEC Software
Sample GPC Analysis Report

Agilent Technologies

Distribution Plot

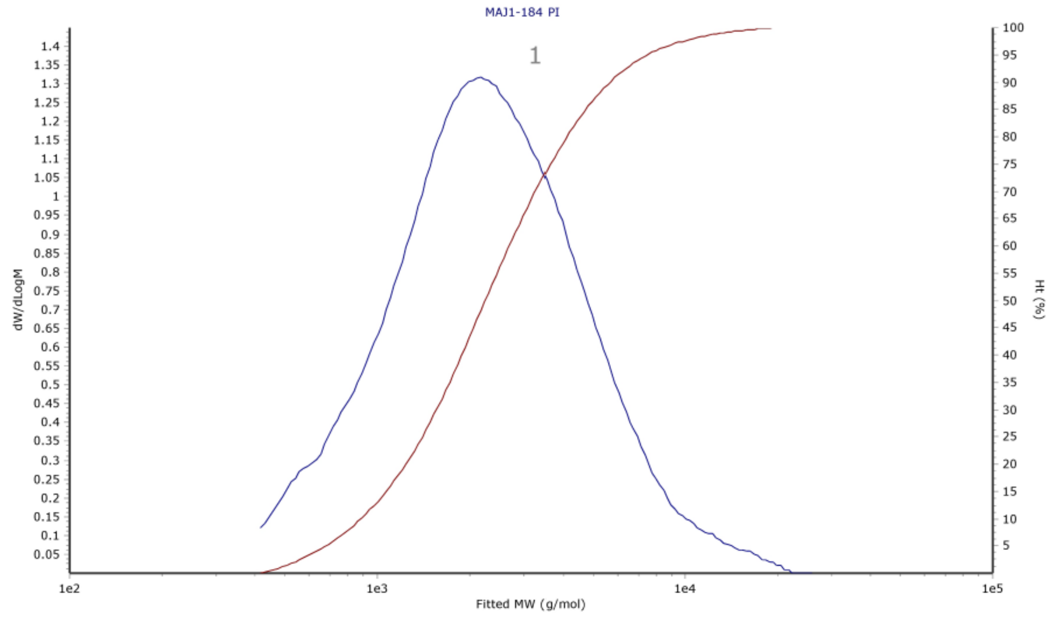

Analyst: .... **Date:** ....

Checked By: .... **Date:** ....

Agilent GPC/SEC Software A.02.01 [9] Page 4 of 4

Generated by GPC at 16:50 on 23 June 2022

Figure S49: GPC data of **2n**.

Polymerizations under vacuum conditions

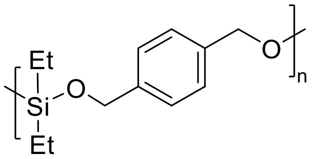

2b

Agilent GPC/SEC Software
Sample GPC Analysis Report

Agilent Technologies

MAJ1-187 pre vac

Workspace Details

Workspace name	Polylactide
Location	C:\ProgramData\Agilent Technologies\GPC\Workspaces\Polylactide\
Comments	
Created by	Administrator at 13:44:31 on 15 June 2015

Sample Properties

Sample name	MAJ1-187 pre vac
File name	ICF_29_06_2022-0004.sample
Collected by	GPC at 14:42:56 on 30 June 2022
Instrument name	Instrument 1

Column Calibration Details

Name	PSty26thMay2022
Created by	GPC at 14:09:29 on 26 May 2022
Last modified by	GPC at 14:10:44 on 26 May 2022
Comments	GPC Column Calibration created 26 May 2022 by GPC<br>GPC Column Calibration amended 26 May 2022 by GPC<br>GPC Column Calibration amended 26 May 2022 by GPC<br>GPC Column Calibration amended 26 May 2022 by GPC<br>GPC Column Calibration amended 26 May 2022 by GPC

Calibration Type	Narrow Standard	Curve Fit Used	3
Calibration Curve	y = - 0.001896x^3 + 0.08659x^2 - 1.684x + 16.11		
High Limit MW RT (mins)	11.21667	Low Limit MW RT (mins)	18.08333
High Limit MW (g/mol)	283800	Low Limit MW (g/mol)	580
Flow Rate Marker Name		Flow Marker RT (mins)	0.00000
K (Input) ((10e-5) dL/g)	14.100		
Alpha (Input)	0.700		
Residual Sum Of Squares	0.00330604	Corrected Sum Of Squares	8.92203
Coeff. Of Determination	0.999629	Standard Y Error Estimate	0.0191661
Linear Correlation Coeff	-0.999452		

Column Calibration Data Points

Point	Peak Max RT (mins)	MW	Log MW	Point in Use?	Percent Error
1	11.21667	283800	5.45	Yes	2.59
2	11.21667	283800	5.45	Yes	2.59
3	11.83333	135700	5.13	Yes	-8.47
4	12.63333	67600	4.83	Yes	-0.98
5	13.58333	29480	4.47	Yes	1.73
6	13.58333	29480	4.47	Yes	1.73
7	13.88333	22290	4.35	Yes	-0.05
8	14.93333	9820	3.99	Yes	7.23
9	15.63333	4910	3.69	Yes	-2.81
10	15.63333	4910	3.69	Yes	-2.81
11	16.18333	3050	3.48	Yes	-3.74
12	17.26667	1250	3.10	Yes	1.77
13	18.08333	580	2.76	Yes	0.33

Analyst: .... **Date:** ....

Checked By: .... **Date:** ....

Agilent GPC/SEC Software A.02.01 [9] Page 1 of 4

Generated by GPC at 16:37 on 02 July 2022

Agilent GPC/SEC Software
Sample GPC Analysis Report

Agilent Technologies

Processing Parameters

Method	Last modified by Administrator at 13:44:30 on 15 June 2015
Using Flow Rate Correction	No
Mark-Houwink K ((10e-5) dL/g)	14.100
Mark-Houwink Alpha	0.700
Concentration Detector Used in Analysis	RI
Injection volume (µL)	100.00
Flow rate (mL/min)	1.00

MW Ranges Method

Calculate MW Ranges No

Percentage Fractions Method

Calculate Percentage Fractions No

Results

Analysed by GPC at 16:37:32 on 02 July 2022

Comments

Molecular Weight Averages

Peak	Mp (g/mol)	Mn (g/mol)	Mw (g/mol)	Mz (g/mol)	Mz+1 (g/mol)	Mv (g/mol)	PD
Peak 1	12961	6245	11993	18761	25056	17822	1.92

Peak Information

	Start (mins)	End (mins)
Baseline region 1	7.60000	8.01667
Baseline region 2	30.08333	30.80000
Peak 1	12.58333	17.23333

Peak Trace Information

Peak	Trace	Peak Max RT (mins)	Peak Area (mV.s)	Peak Height (mV)
Peak 1	RI	14.51667	454.012	3.208
Peak 1	VS DP	14.28333	827.540	6.080
Peak 1	VS IP	14.56667	39.091	0.384
Peak 1	LS 90°	14.16667	85.131	0.913
Peak 1	LS 15°	12.68333	38.504	-0.363

Analyst: .... **Date:** ....

Checked By: .... **Date:** ....

Chromatogram Plot

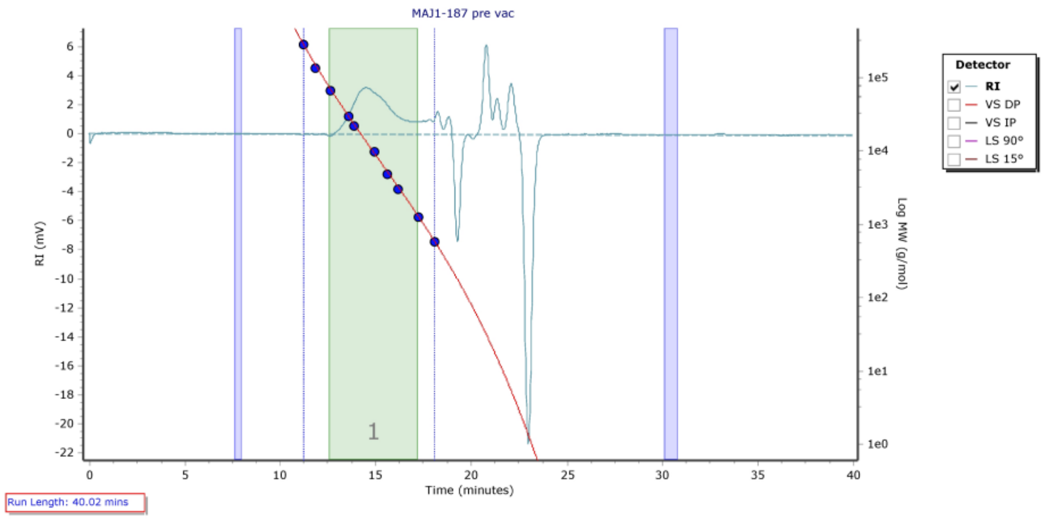

Analyst: .... **Date:** ....

Checked By: .... **Date:** ....

Agilent GPC/SEC Software
Sample GPC Analysis Report

Agilent Technologies

Distribution Plot

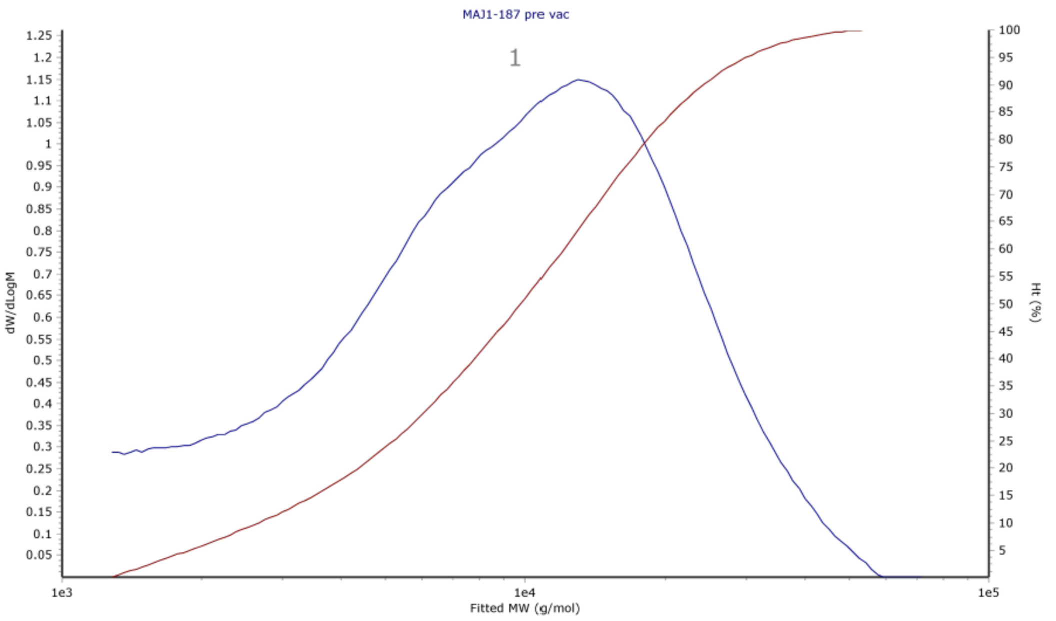

Analyst: .... **Date:** ....

Checked By: .... **Date:** ....

Agilent GPC/SEC Software A.02.01 [9] Page 4 of 4

Generated by GPC at 16:37 on 02 July 2022

Figure S50: GPC data of the crude product formed from the polymerization of **2b** under standard conditions (before further reaction under a dynamic vacuum).

MAJ1-187

Workspace Details

Workspace name	Polylactide
Location	C:\ProgramData\Agilent Technologies\GPC\Workspaces\Polylactide\
Comments	
Created by	Administrator at 13:44:31 on 15 June 2015

Sample Properties

Sample name	MAJ1-187
File name	ICF_29_06_2022-0014.sample
Collected by	GPC at 13:39:21 on 02 July 2022
Instrument name	Instrument 1

Column Calibration Details

Name	PSty26thMay2022
Created by	GPC at 14:09:29 on 26 May 2022
Last modified by	GPC at 14:10:44 on 26 May 2022
Comments	GPC Column Calibration created 26 May 2022 by GPC<br>GPC Column Calibration amended 26 May 2022 by GPC<br>GPC Column Calibration amended 26 May 2022 by GPC<br>GPC Column Calibration amended 26 May 2022 by GPC<br>GPC Column Calibration amended 26 May 2022 by GPC

Calibration Type	Narrow Standard	Curve Fit Used	3
Calibration Curve	y = - 0.001896x^3 + 0.08659x^2 - 1.684x + 16.11		
High Limit MW RT (mins)	11.21667	Low Limit MW RT (mins)	18.08333
High Limit MW (g/mol)	283800	Low Limit MW (g/mol)	580
Flow Rate Marker Name		Flow Marker RT (mins)	0.00000
K (Input) ((10e-5) dL/g)	14.100		
Alpha (Input)	0.700		
Residual Sum Of Squares	0.00330604	Corrected Sum Of Squares	8.92203
Coeff. Of Determination	0.999629	Standard Y Error Estimate	0.0191661
Linear Correlation Coeff	-0.999452		

Column Calibration Data Points

Point	Peak Max RT (mins)	MW	Log MW	Point in Use?	Percent Error
1	11.21667	283800	5.45	Yes	2.59
2	11.21667	283800	5.45	Yes	2.59
3	11.83333	135700	5.13	Yes	-8.47
4	12.63333	67600	4.83	Yes	-0.98
5	13.58333	29480	4.47	Yes	1.73
6	13.58333	29480	4.47	Yes	1.73
7	13.88333	22290	4.35	Yes	-0.05
8	14.93333	9820	3.99	Yes	7.23
9	15.63333	4910	3.69	Yes	-2.81
10	15.63333	4910	3.69	Yes	-2.81
11	16.18333	3050	3.48	Yes	-3.74
12	17.26667	1250	3.10	Yes	1.77
13	18.08333	580	2.76	Yes	0.33

Analyst: .... **Date:** ....

Checked By: .... **Date:** ....

Agilent GPC/SEC Software
Sample GPC Analysis Report

Agilent Technologies

Processing Parameters

Method	Last modified by Administrator at 13:44:30 on 15 June 2015
Using Flow Rate Correction	No
Mark-Houwink K ((10e-5) dL/g)	14.100
Mark-Houwink Alpha	0.700
Concentration Detector Used in Analysis	RI
Injection volume (µL)	100.00
Flow rate (mL/min)	1.00

MW Ranges Method

Calculate MW Ranges — No

Percentage Fractions Method

Calculate Percentage Fractions — No

Results

Analysed by — GPC at 16:40:04 on 02 July 2022

Comments

Molecular Weight Averages

Peak	Mp (g/mol)	Mn (g/mol)	Mw (g/mol)	Mz (g/mol)	Mz+1 (g/mol)	Mv (g/mol)	PD
Peak 1	81055	36282	90362	182767	302840	167474	2.491

Peak Information

	Start (mins)	End (mins)
Baseline region 1	4.98333	5.51667
Baseline region 2	29.13333	29.48333
Peak 1	10.20000	15.76667

Peak Trace Information

Peak	Trace	Peak Max RT (mins)	Peak Area (mV.s)	Peak Height (mV)
Peak 1	RI	12.40000	671.629	4.249
Peak 1	VS DP	11.95000	4046.041	27.256
Peak 1	VS IP	12.06667	151.127	1.029
Peak 1	LS 90°	11.68333	1011.191	8.222
Peak 1	LS 15°	11.68333	294.326	2.539

Analyst: .... **Date:** ....

Checked By: .... **Date:** ....

Agilent GPC/SEC Software
Sample GPC Analysis Report

Agilent Technologies

Chromatogram Plot

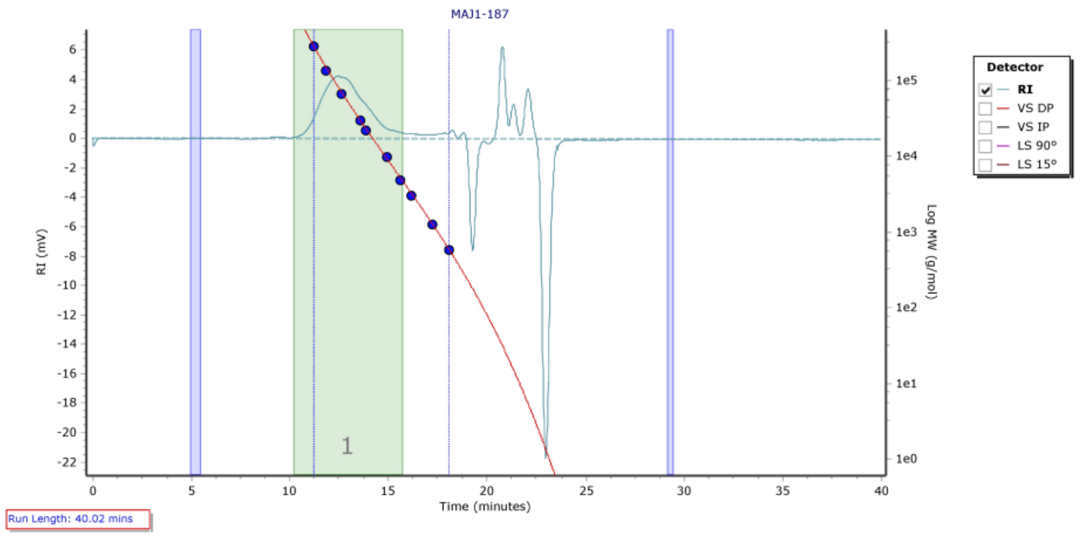

Analyst: .... **Date:** ....

Checked By: .... **Date:** ....

Agilent GPC/SEC Software
Sample GPC Analysis Report

Agilent Technologies

Distribution Plot

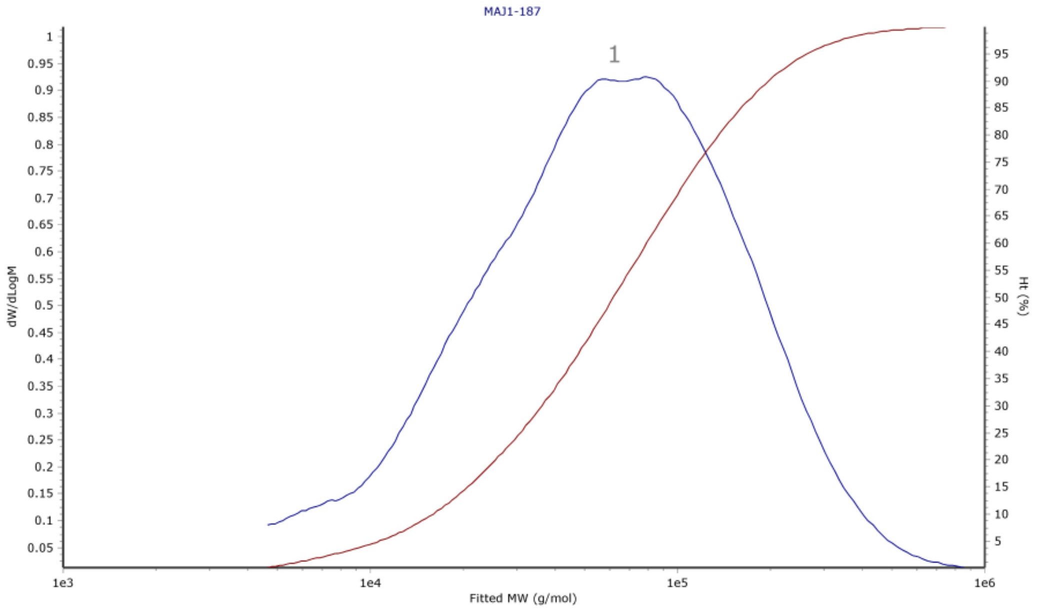

Analyst: .... **Date:** ....

Checked By: .... **Date:** ....

Agilent GPC/SEC Software A.02.01 [9] Page 4 of 4

Generated by GPC at 16:40 on 02 July 2022

Figure S51: GPC data of the pentane insoluble product formed from the polymerization of **2b** under dynamic vacuum conditions.

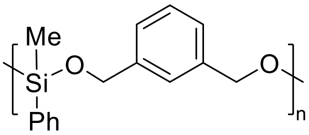

2d

Agilent GPC/SEC Software
Sample GPC Analysis Report

Agilent Technologies

MAJ1-171

Workspace Details

Workspace name	Polylactide
Location	C:\ProgramData\Agilent Technologies\GPC\Workspaces\Polylactide\
Comments	
Created by	Administrator at 13:44:31 on 15 June 2015

Sample Properties

Sample name	MAJ1-171
File name	ICF_16_05_2022-0016-1.sample
Collected by	GPC at 14:09:31 on 17 May 2022
Instrument name	Instrument 1

Column Calibration Details

Name	PSty May11th2022
Created by	GPC at 16:07:12 on 11 May 2022
Last modified by	GPC at 16:09:11 on 11 May 2022
Comments	GPC Column Calibration created 11 May 2022 by GPC<br>GPC Column Calibration amended 11 May 2022 by GPC<br>GPC Column Calibration amended 11 May 2022 by GPC<br>GPC Column Calibration amended 11 May 2022 by GPC

Calibration Type	Narrow Standard	Curve Fit Used	3
Calibration Curve	y = - 0.00209x^3 + 0.09348x^2 - 1.763x + 16.4		
High Limit MW RT (mins)	11.18333	Low Limit MW RT (mins)	17.98333
High Limit MW (g/mol)	283800	Low Limit MW (g/mol)	580
Flow Rate Marker Name		Flow Marker RT (mins)	0.00000
K (Input) ((10e-5) dL/g)	14.100		
Alpha (Input)	0.700		
Residual Sum Of Squares	0.00170957	Corrected Sum Of Squares	6.9722
Coeff. Of Determination	0.999755	Standard Y Error Estimate	0.0168798
Linear Correlation Coeff	-0.999649		

Column Calibration Data Points

Point	Peak Max RT (mins)	MW	Log MW	Point in Use?	Percent Error
1	11.18333	283800	5.45	Yes	2.07
2	11.88333	135700	5.13	Yes	-0.52
3	12.55000	67600	4.83	Yes	-6.63
4	13.56667	29460	4.47	Yes	2.25
5	13.90000	22390	4.35	Yes	3.82
6	14.85000	9820	3.99	Yes	2.84
7	15.58333	4910	3.69	Yes	-3.89
8	16.16667	3050	3.48	Yes	-0.93
9	17.16667	1250	3.10	Yes	-0.35
10	17.98333	580	2.76	Yes	0.89

Analyst: .... **Date:** ....

Checked By: .... **Date:** ....

Agilent GPC/SEC Software
Sample GPC Analysis Report

Agilent Technologies

Processing Parameters

Method	Last modified by Administrator at 13:44:30 on 15 June 2015
Using Flow Rate Correction	No
Mark-Houwink K ((10e-5) dL/g)	14.100
Mark-Houwink Alpha	0.700
Concentration Detector Used in Analysis	RI
Injection volume (µL)	100.00
Flow rate (mL/min)	1.00

MW Ranges Method

Calculate MW Ranges   No

Percentage Fractions Method

Calculate Percentage Fractions   No

Results

Analysed by   GPC at 15:10:10 on 17 May 2022

Comments

Molecular Weight Averages

Peak	Mp (g/mol)	Mn (g/mol)	Mw (g/mol)	Mz (g/mol)	Mz+1 (g/mol)	Mv (g/mol)	PD
Peak 1	15042	8354	34533	274240	906930	207514	4.134

Peak Information

	Start (mins)	End (mins)
Baseline region 1	4.25000	5.96667
Baseline region 2	32.30000	33.98333
Peak 1	9.60000	16.98333

Peak Trace Information

Peak	Trace	Peak Max RT (mins)	Peak Area (mV.s)	Peak Height (mV)
Peak 1	RI	14.31667	3435.551	17.454
Peak 1	VS DP	13.26667	4891.070	21.258
Peak 1	VS IP	13.86667	79.646	0.585
Peak 1	LS 90°	12.80000	4647.067	17.664
Peak 1	LS 15°	16.96667	1726.403	15.089

Analyst: .... **Date:** ....

Checked By: .... **Date:** ....

Agilent GPC/SEC Software
Sample GPC Analysis Report

Agilent Technologies

Chromatogram Plot

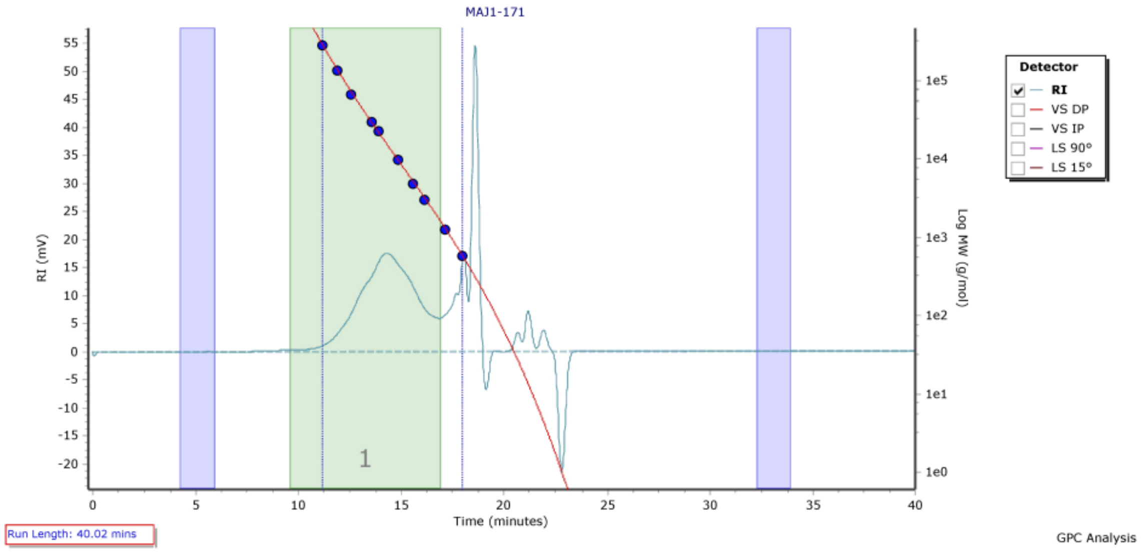

Analyst: .... **Date:** ....

Checked By: .... **Date:** ....

Agilent GPC/SEC Software
Sample GPC Analysis Report

Agilent Technologies

Distribution Plot

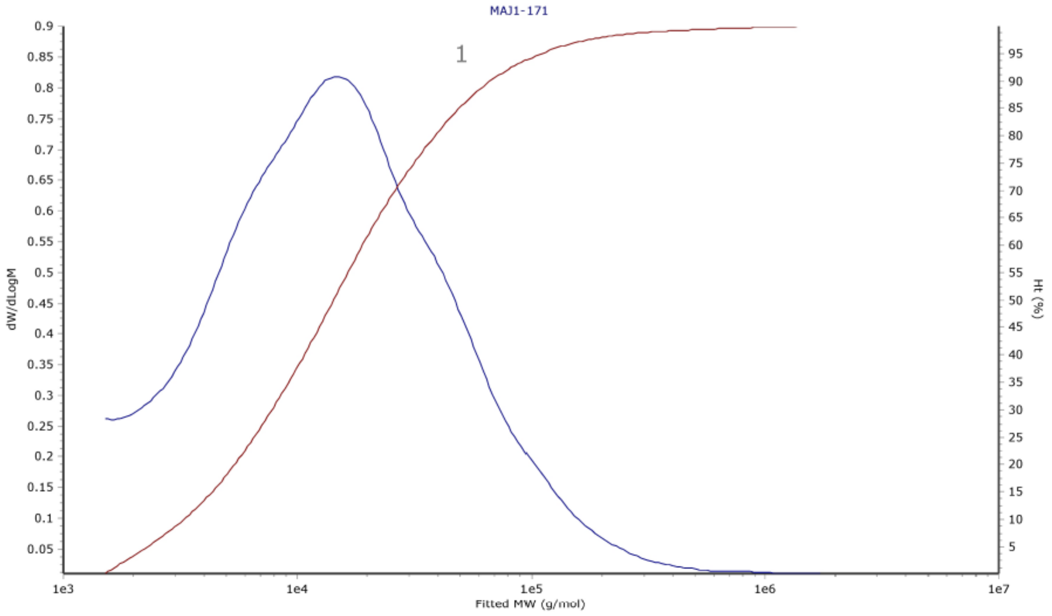

Analyst: .... **Date:** ....

Checked By: .... **Date:** ....

Agilent GPC/SEC Software A.02.01 [9] Page 4 of 4

Generated by GPC at 18:12 on 23 May 2022

Figure S52: GPC data of the product formed from the polymerization of **2d** under vacuum conditions (~~see Figure S43 for pre vacuum GPC results~~see Figure S43 for pre vacuum GPC results).

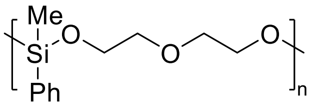

2f

Agilent GPC/SEC Software
Sample GPC Analysis Report

Agilent Technologies

MAJ1-173

Workspace Details

Workspace name	Polylactide
Location	C:\ProgramData\Agilent Technologies\GPC\Workspaces\Polylactide\
Comments	
Created by	Administrator at 13:44:31 on 15 June 2015

Sample Properties

Sample name	MAJ1-173
File name	ICF_18_05_2022-0013.sample
Collected by	GPC at 17:14:02 on 19 May 2022
Instrument name	Instrument 1

Column Calibration Details

Name	PSty May11th2022
Created by	GPC at 16:07:12 on 11 May 2022
Last modified by	GPC at 16:09:11 on 11 May 2022
Comments	GPC Column Calibration created 11 May 2022 by GPC<br>GPC Column Calibration amended 11 May 2022 by GPC<br>GPC Column Calibration amended 11 May 2022 by GPC<br>GPC Column Calibration amended 11 May 2022 by GPC

Calibration Type	Narrow Standard	Curve Fit Used	3
Calibration Curve	y = - 0.00209x^3 + 0.09348x^2 - 1.763x + 16.4		
High Limit MW RT (mins)	11.18333	Low Limit MW RT (mins)	17.98333
High Limit MW (g/mol)	283800	Low Limit MW (g/mol)	580
Flow Rate Marker Name		Flow Marker RT (mins)	0.00000
K (Input) ((10e-5) dL/g)	14.100		
Alpha (Input)	0.700		
Residual Sum Of Squares	0.00170957	Corrected Sum Of Squares	6.9722
Coeff. Of Determination	0.999755	Standard Y Error Estimate	0.0168798
Linear Correlation Coeff	-0.999649		

Column Calibration Data Points

Point	Peak Max RT (mins)	MW	Log MW	Point in Use?	Percent Error
1	11.18333	283800	5.45	Yes	2.07
2	11.88333	135700	5.13	Yes	-0.52
3	12.55000	67600	4.83	Yes	-6.63
4	13.56667	29460	4.47	Yes	2.25
5	13.90000	22390	4.35	Yes	3.82
6	14.85000	9820	3.99	Yes	2.84
7	15.58333	4910	3.69	Yes	-3.89
8	16.16667	3050	3.48	Yes	-0.93
9	17.16667	1250	3.10	Yes	-0.35
10	17.98333	580	2.76	Yes	0.89

Analyst: .... **Date:** ....

Checked By: .... **Date:** ....

Agilent GPC/SEC Software
Sample GPC Analysis Report

Agilent Technologies

Processing Parameters

Method	Last modified by Administrator at 13:44:30 on 15 June 2015
Using Flow Rate Correction	No
Mark-Houwink K ((10e-5) dL/g)	14.100
Mark-Houwink Alpha	0.700
Concentration Detector Used in Analysis	RI
Injection volume (µL)	100.00
Flow rate (mL/min)	1.00

MW Ranges Method

Calculate MW Ranges No

Percentage Fractions Method

Calculate Percentage Fractions No

Results

Analysed by GPC at 16:22:58 on 23 May 2022

Comments

Molecular Weight Averages

Peak	Mp (g/mol)	Mn (g/mol)	Mw (g/mol)	Mz (g/mol)	Mz+1 (g/mol)	Mv (g/mol)	PD
Peak 1	783	1060	1258	1546	1924	1496	1.187

Peak Information

	Start (mins)	End (mins)
Baseline region 1	8.01667	8.73333
Baseline region 2	27.95000	28.80000
Peak 1	15.65000	17.93333

Peak Trace Information

Peak	Trace	Peak Max RT (mins)	Peak Area (mV.s)	Peak Height (mV)
Peak 1	RI	17.66667	200.962	3.273
Peak 1	VS DP	17.16667	75.387	1.183
Peak 1	VS IP	16.53333	9.922	0.258
Peak 1	LS 90°	17.23333	3.656	0.063
Peak 1	LS 15°	17.35000	40.523	0.333

Analyst: .... **Date:** ....

Checked By: .... **Date:** ....

Agilent GPC/SEC Software
Sample GPC Analysis Report

Chromatogram Plot

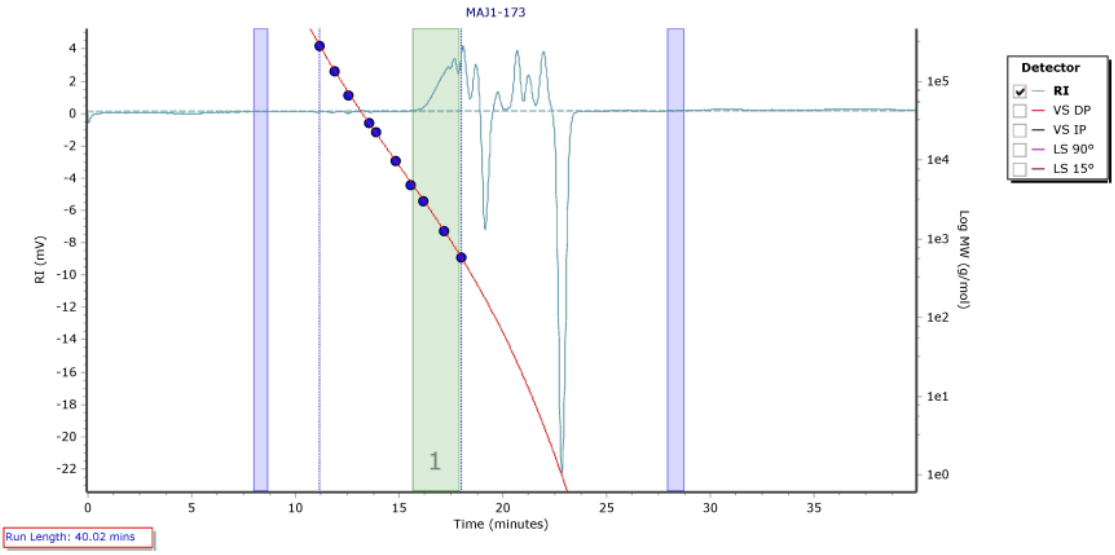

Analyst: .... **Date:** ....

Checked By: .... **Date:** ....

Agilent GPC/SEC Software
Sample GPC Analysis Report

Agilent Technologies

Distribution Plot

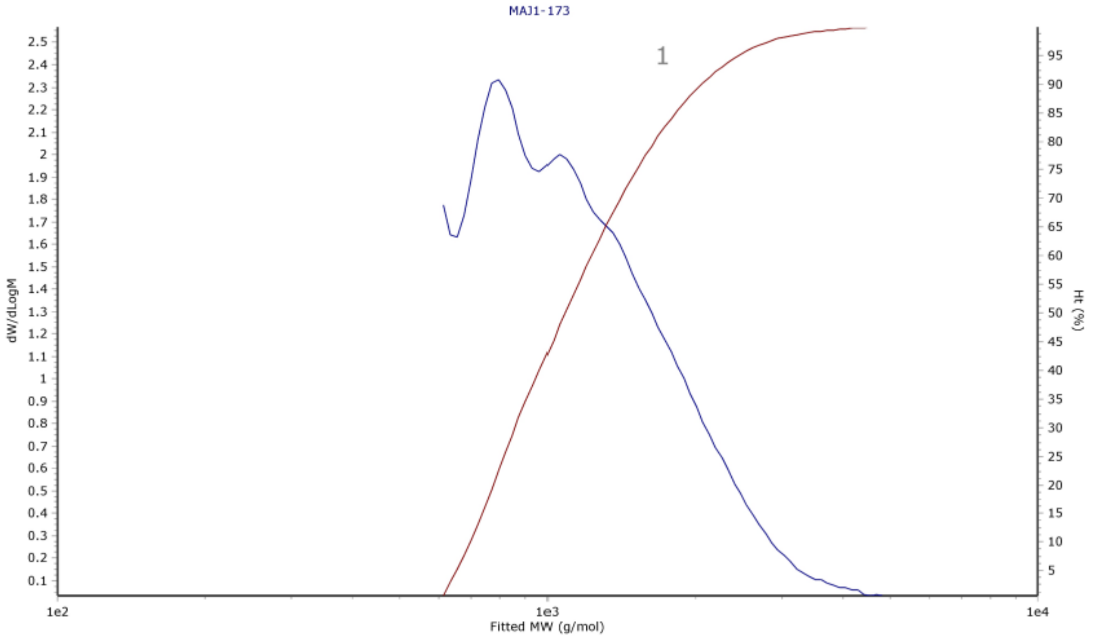

Analyst: .... **Date:** ....

Checked By: .... **Date:** ....

Agilent GPC/SEC Software A.02.01 [9] Page 4 of 4

Generated by GPC at 16:23 on 23 May 2022

Figure S53: GPC data of the crude product formed from the polymerization of **2f** under standard conditions (before further reaction under a dynamic vacuum).

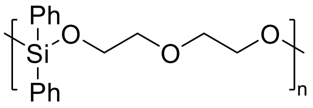

2g

Agilent GPC/SEC Software
Sample GPC Analysis Report

Agilent Technologies

MAJ1-174

Workspace Details

Workspace name	Polylactide
Location	C:\ProgramData\Agilent Technologies\GPC\Workspaces\Polylactide\
Comments	
Created by	Administrator at 13:44:31 on 15 June 2015

Sample Properties

Sample name	MAJ1-174
File name	ICF_18_05_2022-0014.sample
Collected by	GPC at 17:55:52 on 19 May 2022
Instrument name	Instrument 1

Column Calibration Details

Name	PSty May11th2022
Created by	GPC at 16:07:12 on 11 May 2022
Last modified by	GPC at 16:09:11 on 11 May 2022
Comments	GPC Column Calibration created 11 May 2022 by GPC<br>GPC Column Calibration amended 11 May 2022 by GPC<br>GPC Column Calibration amended 11 May 2022 by GPC<br>GPC Column Calibration amended 11 May 2022 by GPC

Calibration Type	Narrow Standard	Curve Fit Used	3
Calibration Curve	y = - 0.00209x^3 + 0.09348x^2 - 1.763x + 16.4		
High Limit MW RT (mins)	11.18333	Low Limit MW RT (mins)	17.98333
High Limit MW (g/mol)	283800	Low Limit MW (g/mol)	580
Flow Rate Marker Name		Flow Marker RT (mins)	0.00000
K (Input) ((10e-5) dL/g)	14.100		
Alpha (Input)	0.700		
Residual Sum Of Squares	0.00170957	Corrected Sum Of Squares	6.9722
Coeff. Of Determination	0.999755	Standard Y Error Estimate	0.0168798
Linear Correlation Coeff	-0.999649		

Column Calibration Data Points

Point	Peak Max RT (mins)	MW	Log MW	Point in Use?	Percent Error
1	11.18333	283800	5.45	Yes	2.07
2	11.88333	135700	5.13	Yes	-0.52
3	12.55000	67600	4.83	Yes	-6.63
4	13.56667	29460	4.47	Yes	2.25
5	13.90000	22390	4.35	Yes	3.82
6	14.85000	9820	3.99	Yes	2.84
7	15.58333	4910	3.69	Yes	-3.89
8	16.16667	3050	3.48	Yes	-0.93
9	17.16667	1250	3.10	Yes	-0.35
10	17.98333	580	2.76	Yes	0.89

Analyst: .... **Date:** ....

Checked By: .... **Date:** ....

Agilent GPC/SEC Software
Sample GPC Analysis Report

Agilent Technologies

Processing Parameters

Method	Last modified by Administrator at 13:44:30 on 15 June 2015
Using Flow Rate Correction	No
Mark-Houwink K ((10e-5) dL/g)	14.100
Mark-Houwink Alpha	0.700
Concentration Detector Used in Analysis	RI
Injection volume (µL)	100.00
Flow rate (mL/min)	1.00

MW Ranges Method

Calculate MW Ranges	No

Percentage Fractions Method

Calculate Percentage Fractions	No

Results

Analysed by	GPC at 16:24:13 on 23 May 2022
Comments	

Molecular Weight Averages

Peak	Mp (g/mol)	Mn (g/mol)	Mw (g/mol)	Mz (g/mol)	Mz+1 (g/mol)	Mv (g/mol)	PD
Peak 1	4054	2392	3893	5948	8043	5641	1.628

Peak Information

	Start (mins)	End (mins)
Baseline region 1	6.76667	7.36667
Baseline region 2	30.93333	31.56667
Peak 1	13.80000	17.63333

Peak Trace Information

Peak	Trace	Peak Max RT (mins)	Peak Area (mV.s)	Peak Height (mV)
Peak 1	RI	15.85000	516.283	3.734
Peak 1	VS DP	15.60000	321.704	2.308
Peak 1	VS IP	15.40000	27.049	0.359
Peak 1	LS 90°	15.46667	79.256	0.669
Peak 1	LS 15°	17.58333	53.114	-0.404

Analyst: .... **Date:** ....

Checked By: .... **Date:** ....

Agilent GPC/SEC Software
Sample GPC Analysis Report

Agilent Technologies

Chromatogram Plot

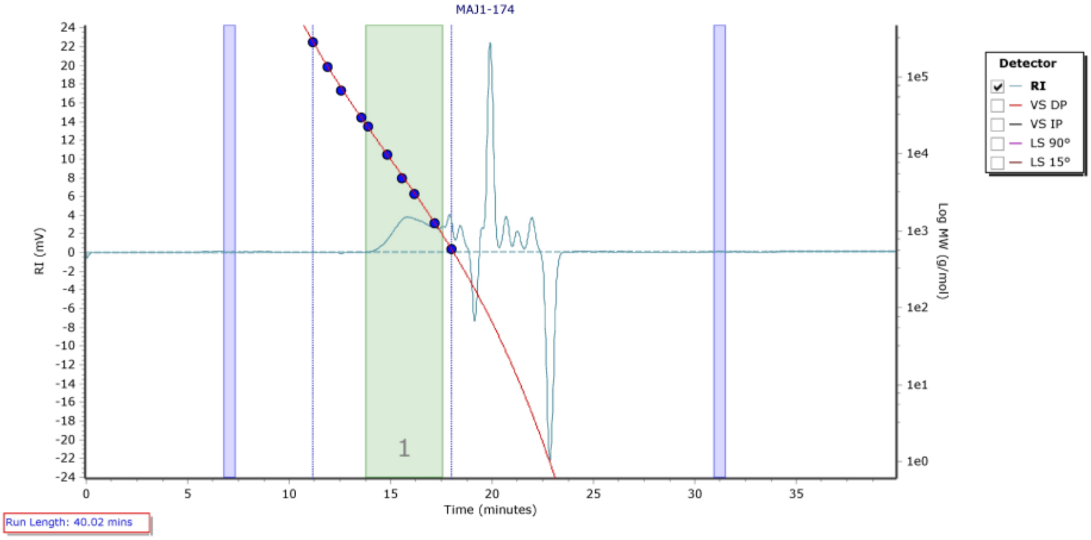

Analyst: .... **Date:** ....

Checked By: .... **Date:** ....

Agilent GPC/SEC Software
Sample GPC Analysis Report

Distribution Plot

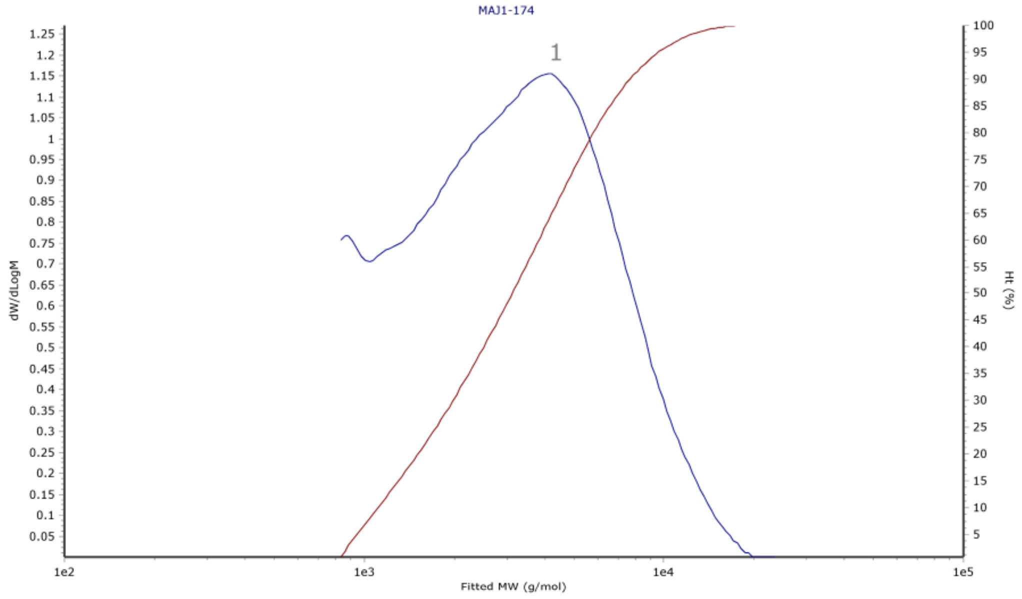

Analyst: .... **Date:** ....

Checked By: .... **Date:** ....

Agilent GPC/SEC Software A.02.01 [9]   Page 4 of 4

Generated by GPC at 16:24 on 23 May 2022

Figure S54: GPC data of the crude product formed from the polymerization of **2g** under standard conditions (before further reaction under a dynamic vacuum).

MAJ1-174 vac poly

Workspace Details

Workspace name	Polylactide
Location	C:\ProgramData\Agilent Technologies\GPC\Workspaces\Polylactide\
Comments	
Created by	Administrator at 13:44:31 on 15 June 2015

Sample Properties

Sample name	MAJ1-174 vac poly
File name	ICF_20_05_2022-0003.sample
Collected by	GPC at 13:07:32 on 20 May 2022
Instrument name	Instrument 1

Column Calibration Details

Name	PSty May11th2022
Created by	GPC at 16:07:12 on 11 May 2022
Last modified by	GPC at 16:09:11 on 11 May 2022
Comments	GPC Column Calibration created 11 May 2022 by GPC<br>GPC Column Calibration amended 11 May 2022 by GPC<br>GPC Column Calibration amended 11 May 2022 by GPC<br>GPC Column Calibration amended 11 May 2022 by GPC

Calibration Type	Narrow Standard	Curve Fit Used	3
Calibration Curve	y = - 0.00209x^3 + 0.09348x^2 - 1.763x + 16.4		
High Limit MW RT (mins)	11.18333	Low Limit MW RT (mins)	17.98333
High Limit MW (g/mol)	283800	Low Limit MW (g/mol)	580
Flow Rate Marker Name		Flow Marker RT (mins)	0.00000
K (Input) ((10e-5) dL/g)	14.100		
Alpha (Input)	0.700		
Residual Sum Of Squares	0.00170957	Corrected Sum Of Squares	6.9722
Coeff. Of Determination	0.999755	Standard Y Error Estimate	0.0168798
Linear Correlation Coeff	-0.999649		

Column Calibration Data Points

Point	Peak Max RT (mins)	MW	Log MW	Point in Use?	Percent Error
1	11.18333	283800	5.45	Yes	2.07
2	11.88333	135700	5.13	Yes	-0.52
3	12.55000	67600	4.83	Yes	-6.63
4	13.56667	29460	4.47	Yes	2.25
5	13.90000	22390	4.35	Yes	3.82
6	14.85000	9820	3.99	Yes	2.84
7	15.58333	4910	3.69	Yes	-3.89
8	16.16667	3050	3.48	Yes	-0.93
9	17.16667	1250	3.10	Yes	-0.35
10	17.98333	580	2.76	Yes	0.89

Analyst: .... **Date:** ....

Checked By: .... **Date:** ....

Agilent GPC/SEC Software
Sample GPC Analysis Report

Agilent Technologies

Processing Parameters

Method	Last modified by Administrator at 13:44:30 on 15 June 2015
Using Flow Rate Correction	No
Mark-Houwink K ((10e-5) dL/g)	14.100
Mark-Houwink Alpha	0.700
Concentration Detector Used in Analysis	RI
Injection volume (µL)	100.00
Flow rate (mL/min)	1.00

MW Ranges Method

Calculate MW Ranges No

Percentage Fractions Method

Calculate Percentage Fractions No

Results

Analysed by GPC at 14:49:44 on 20 May 2022

Comments

Molecular Weight Averages

Peak	Mp (g/mol)	Mn (g/mol)	Mw (g/mol)	Mz (g/mol)	Mz+1 (g/mol)	Mv (g/mol)	PD
Peak 1	19751	14986	32925	62855	99780	57972	2.197

Peak Information

	Start (mins)	End (mins)
Baseline region 1	9.11667	9.61667
Baseline region 2	35.55000	36.01667
Peak 1	11.06667	16.46667

Peak Trace Information

Peak	Trace	Peak Max RT (mins)	Peak Area (mV.s)	Peak Height (mV)
Peak 1	RI	14.00000	301.895	1.876
Peak 1	VS DP	13.00000	410.497	3.017
Peak 1	VS IP	11.18333	6.919	-0.346
Peak 1	LS 90°	12.93333	299.284	2.445
Peak 1	LS 15°	12.93333	112.062	0.811

Analyst: .... **Date:** ....

Checked By: .... **Date:** ....

Agilent GPC/SEC Software
Sample GPC Analysis Report

Agilent Technologies

Chromatogram Plot

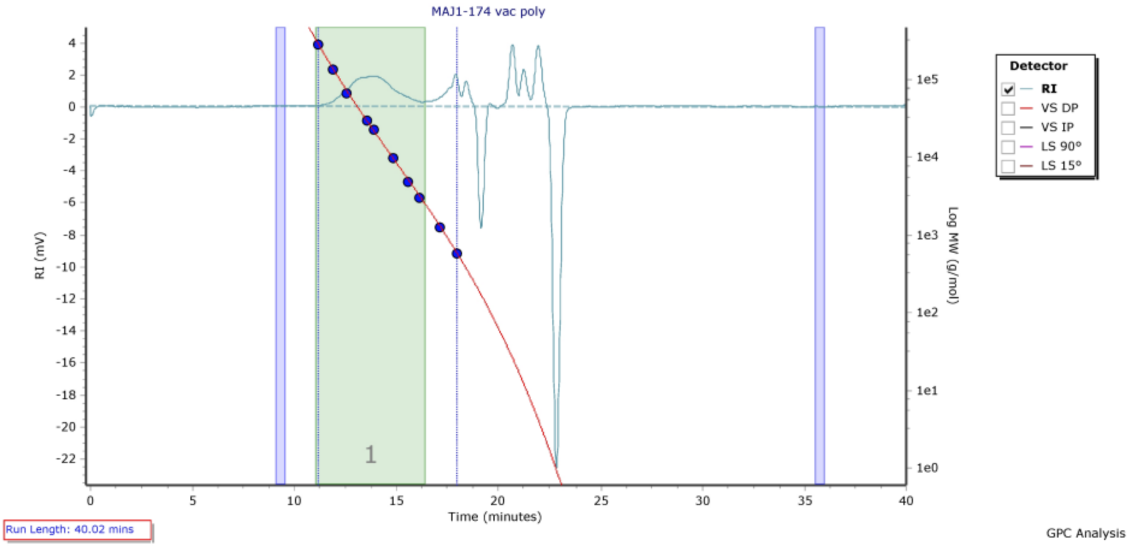

Analyst: .... **Date:** ....

Checked By: .... **Date:** ....

Agilent GPC/SEC Software
Sample GPC Analysis Report

Agilent Technologies

Distribution Plot

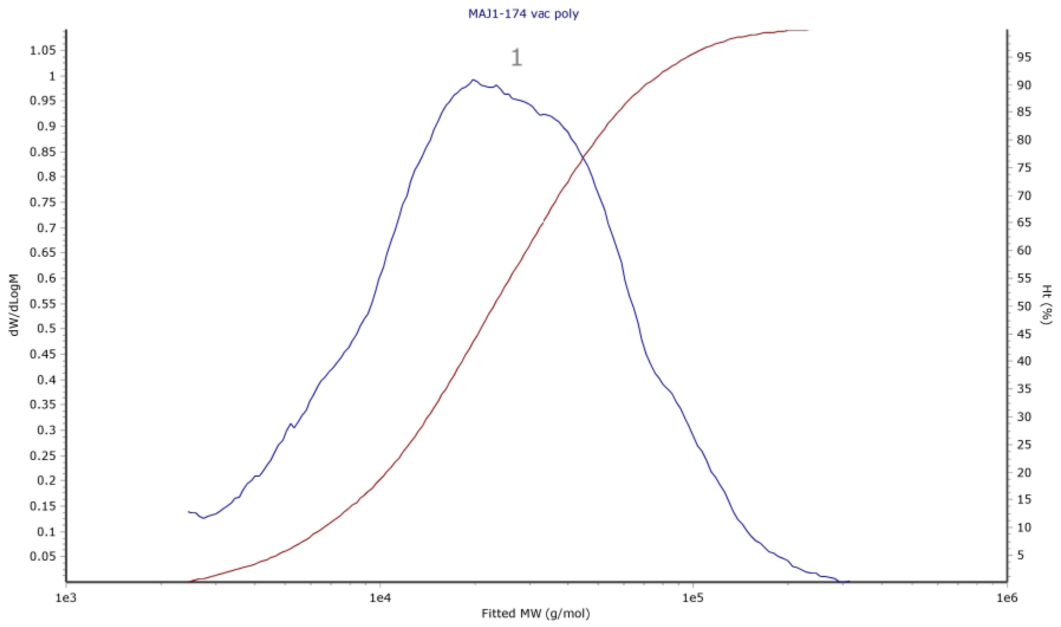

Analyst: .... **Date:** ....

Checked By: .... **Date:** ....

Agilent GPC/SEC Software A.02.01 [9]   Page 4 of 4

Generated by GPC at 14:53 on 20 May 2022

Figure S55: GPC data of the crude product formed from the polymerization of **2g** under dynamic vacuum conditions.

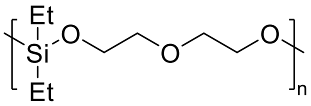

2h

Agilent GPC/SEC Software
Sample GPC Analysis Report

Agilent Technologies

MAJ1-188 pre vac

Workspace Details

Workspace name	Polylactide
Location	C:\ProgramData\Agilent Technologies\GPC\Workspaces\Polylactide\
Comments	
Created by	Administrator at 13:44:31 on 15 June 2015

Sample Properties

Sample name	MAJ1-188 pre vac
File name	ICF_29_06_2022-0005.sample
Collected by	GPC at 15:24:53 on 30 June 2022
Instrument name	Instrument 1

Column Calibration Details

Name	PSty26thMay2022
Created by	GPC at 14:09:29 on 26 May 2022
Last modified by	GPC at 14:10:44 on 26 May 2022
Comments	GPC Column Calibration created 26 May 2022 by GPC<br>GPC Column Calibration amended 26 May 2022 by GPC<br>GPC Column Calibration amended 26 May 2022 by GPC<br>GPC Column Calibration amended 26 May 2022 by GPC<br>GPC Column Calibration amended 26 May 2022 by GPC

Calibration Type	Narrow Standard	Curve Fit Used	3
Calibration Curve	y = - 0.001896x^3 + 0.08659x^2 - 1.684x + 16.11		
High Limit MW RT (mins)	11.21667	Low Limit MW RT (mins)	18.08333
High Limit MW (g/mol)	283800	Low Limit MW (g/mol)	580
Flow Rate Marker Name		Flow Marker RT (mins)	0.00000
K (Input) ((10e-5) dL/g)	14.100		
Alpha (Input)	0.700		
Residual Sum Of Squares	0.00330604	Corrected Sum Of Squares	8.92203
Coeff. Of Determination	0.999629	Standard Y Error Estimate	0.0191661
Linear Correlation Coeff	-0.999452		

Column Calibration Data Points

Point	Peak Max RT (mins)	MW	Log MW	Point in Use?	Percent Error
1	11.21667	283800	5.45	Yes	2.59
2	11.21667	283800	5.45	Yes	2.59
3	11.83333	135700	5.13	Yes	-8.47
4	12.63333	67600	4.83	Yes	-0.98
5	13.58333	29480	4.47	Yes	1.73
6	13.58333	29480	4.47	Yes	1.73
7	13.88333	22290	4.35	Yes	-0.05
8	14.93333	9820	3.99	Yes	7.23
9	15.63333	4910	3.69	Yes	-2.81
10	15.63333	4910	3.69	Yes	-2.81
11	16.18333	3050	3.48	Yes	-3.74
12	17.26667	1250	3.10	Yes	1.77
13	18.08333	580	2.76	Yes	0.33

Analyst: .... **Date:** ....

Checked By: .... **Date:** ....

Agilent GPC/SEC Software
Sample GPC Analysis Report

Agilent Technologies

Processing Parameters

Method	Last modified by Administrator at 13:44:30 on 15 June 2015
Using Flow Rate Correction	No
Mark-Houwink K ((10e-5) dL/g)	14.100
Mark-Houwink Alpha	0.700
Concentration Detector Used in Analysis	RI
Injection volume (µL)	100.00
Flow rate (mL/min)	1.00

MW Ranges Method

Calculate MW Ranges — No

Percentage Fractions Method

Calculate Percentage Fractions — No

Results

Analysed by — GPC at 16:30:58 on 02 July 2022

Comments

Molecular Weight Averages

Peak	Mp (g/mol)	Mn (g/mol)	Mw (g/mol)	Mz (g/mol)	Mz+1 (g/mol)	Mv (g/mol)	PD
Peak 1	1803	1465	1990	2669	3374	2566	1.358

Peak Information

	Start (mins)	End (mins)
Baseline region 1	7.48333	8.21667
Baseline region 2	32.13333	32.63333
Peak 1	14.66667	18.05000

Peak Trace Information

Peak	Trace	Peak Max RT (mins)	Peak Area (mV.s)	Peak Height (mV)
Peak 1	RI	16.83333	196.524	1.798
Peak 1	VS DP	16.75000	227.799	1.945
Peak 1	VS IP	16.35000	15.757	0.252
Peak 1	LS 90°	14.91667	15.624	-0.146
Peak 1	LS 15°	17.50000	48.791	-0.277

Analyst: .... **Date:** ....

Checked By: .... **Date:** ....

Chromatogram Plot

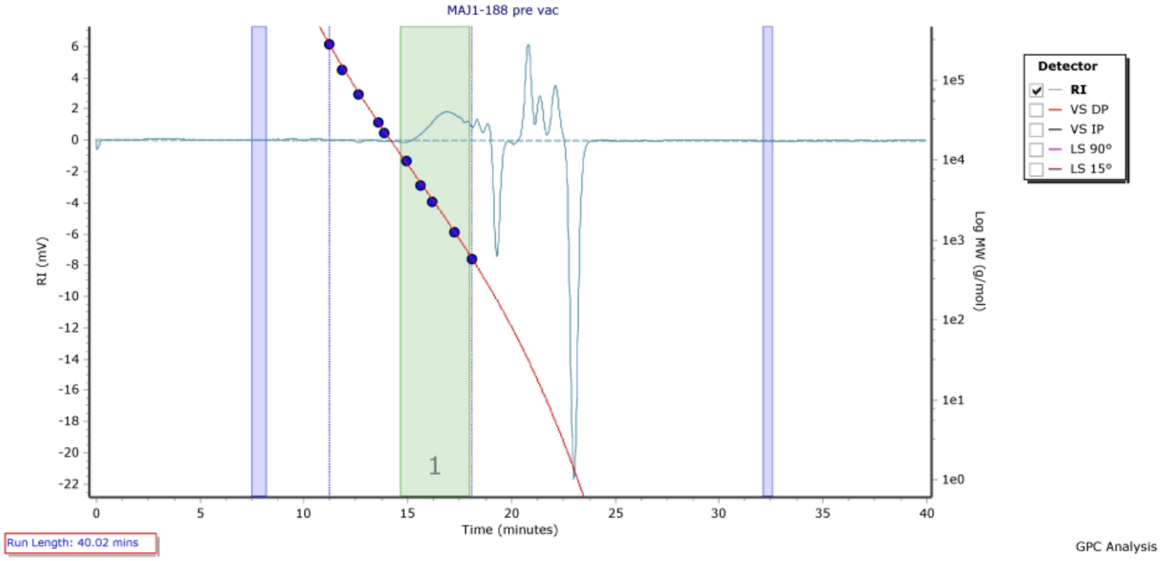

Analyst: .... **Date:** ....

Checked By: .... **Date:** ....

Agilent GPC/SEC Software
Sample GPC Analysis Report

Agilent Technologies

Distribution Plot

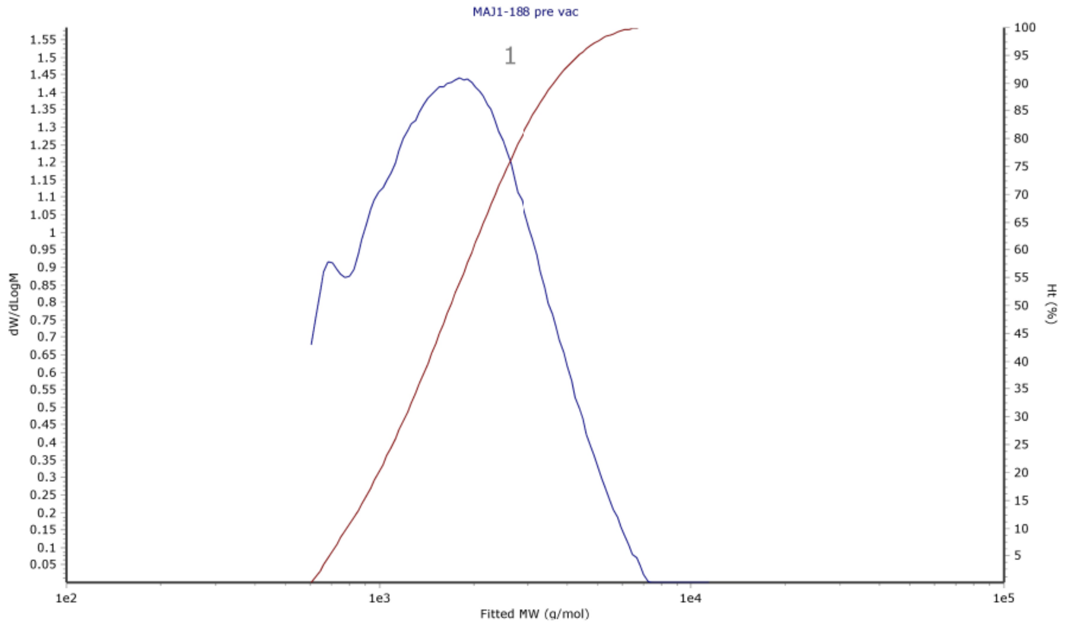

Analyst: .... **Date:** ....

Checked By: .... **Date:** ....

Agilent GPC/SEC Software A.02.01 [9] Page 4 of 4

Generated by GPC at 16:34 on 02 July 2022

Figure S56: GPC data of the crude product formed from the polymerization of **2h** under standard conditions (before further reaction under a dynamic vacuum).

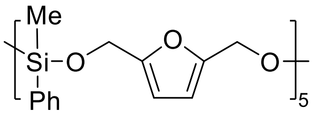

2n

Agilent GPC/SEC Software
Sample GPC Analysis Report

Agilent Technologies

MAJ1-189 pre vac

Workspace Details

Workspace name	Polylactide
Location	C:\ProgramData\Agilent Technologies\GPC\Workspaces\Polylactide\
Comments	
Created by	Administrator at 13:44:31 on 15 June 2015

Sample Properties

Sample name	MAJ1-189 pre vac
File name	ICF_29_06_2022-0006.sample
Collected by	GPC at 16:06:44 on 30 June 2022
Instrument name	Instrument 1

Column Calibration Details

Name	PSty26thMay2022
Created by	GPC at 14:09:29 on 26 May 2022
Last modified by	GPC at 14:10:44 on 26 May 2022
Comments	GPC Column Calibration created 26 May 2022 by GPC<br>GPC Column Calibration amended 26 May 2022 by GPC<br>GPC Column Calibration amended 26 May 2022 by GPC<br>GPC Column Calibration amended 26 May 2022 by GPC<br>GPC Column Calibration amended 26 May 2022 by GPC

Calibration Type	Narrow Standard	Curve Fit Used	3
Calibration Curve	y = - 0.001896x^3 + 0.08659x^2 - 1.684x + 16.11		
High Limit MW RT (mins)	11.21667	Low Limit MW RT (mins)	18.08333
High Limit MW (g/mol)	283800	Low Limit MW (g/mol)	580
Flow Rate Marker Name		Flow Marker RT (mins)	0.00000
K (Input) ((10e-5) dL/g)	14.100		
Alpha (Input)	0.700		
Residual Sum Of Squares	0.00330604	Corrected Sum Of Squares	8.92203
Coeff. Of Determination	0.999629	Standard Y Error Estimate	0.0191661
Linear Correlation Coeff	-0.999452		

Column Calibration Data Points

Point	Peak Max RT (mins)	MW	Log MW	Point in Use?	Percent Error
1	11.21667	283800	5.45	Yes	2.59
2	11.21667	283800	5.45	Yes	2.59
3	11.83333	135700	5.13	Yes	-8.47
4	12.63333	67600	4.83	Yes	-0.98
5	13.58333	29480	4.47	Yes	1.73
6	13.58333	29480	4.47	Yes	1.73
7	13.88333	22290	4.35	Yes	-0.05
8	14.93333	9820	3.99	Yes	7.23
9	15.63333	4910	3.69	Yes	-2.81
10	15.63333	4910	3.69	Yes	-2.81
11	16.18333	3050	3.48	Yes	-3.74
12	17.26667	1250	3.10	Yes	1.77
13	18.08333	580	2.76	Yes	0.33

Analyst: .... **Date:** ....

Checked By: .... **Date:** ....

Agilent GPC/SEC Software
Sample GPC Analysis Report

Agilent Technologies

Processing Parameters

Method	Last modified by Administrator at 13:44:30 on 15 June 2015
Using Flow Rate Correction	No
Mark-Houwink K ((10e-5) dL/g)	14.100
Mark-Houwink Alpha	0.700
Concentration Detector Used in Analysis	RI
Injection volume (µL)	100.00
Flow rate (mL/min)	1.00

MW Ranges Method

Calculate MW Ranges No

Percentage Fractions Method

Calculate Percentage Fractions No

Results

Analysed by GPC at 16:38:43 on 02 July 2022

Comments

Molecular Weight Averages

Peak	Mp (g/mol)	Mn (g/mol)	Mw (g/mol)	Mz (g/mol)	Mz+1 (g/mol)	Mv (g/mol)	PD
Peak 1	828	1257	1883	3042	4533	2840	1.498

Peak Information

	Start (mins)	End (mins)
Baseline region 1	5.30000	5.86667
Baseline region 2	33.46667	34.16667
Peak 1	14.81667	18.11667

Peak Trace Information

Peak	Trace	Peak Max RT (mins)	Peak Area (mV.s)	Peak Height (mV)
Peak 1	RI	17.71667	344.279	3.067
Peak 1	VS DP	17.23333	134.156	1.361
Peak 1	VS IP	16.08333	3.172	-0.326
Peak 1	LS 90°	15.88333	11.019	0.225
Peak 1	LS 15°	15.18333	27.288	-0.177

Analyst: .... **Date:** ....

Checked By: .... **Date:** ....

Chromatogram Plot

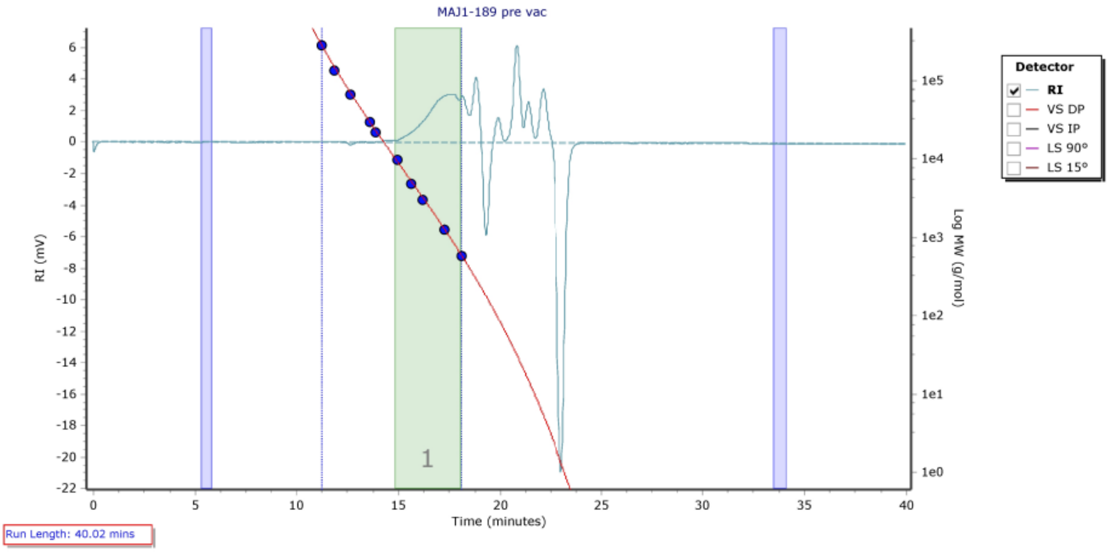

Analyst: .... **Date:** ....

Checked By: .... **Date:** ....

Agilent GPC/SEC Software
Sample GPC Analysis Report

Agilent Technologies

Distribution Plot

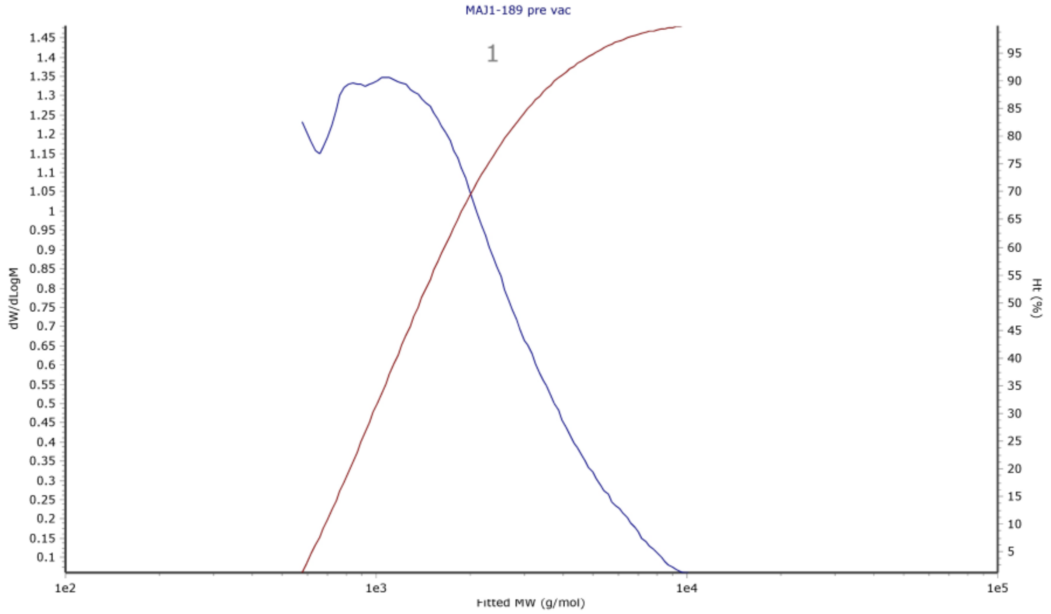

Analyst: .... **Date:** ....

Checked By: .... **Date:** ....

Agilent GPC/SEC Software A.02.01 [9]   Page 4 of 4

Generated by GPC at 16:38 on 02 July 2022

Figure S57: GPC data of the crude product formed from the polymerization of **2n** under standard conditions (before further reaction under a dynamic vacuum).

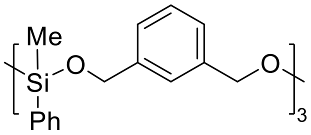

2d

Agilent GPC/SEC Software
Sample GPC Analysis Report

Agilent Technologies

MAJ1-172

Workspace Details

Workspace name	Polylactide
Location	C:\ProgramData\Agilent Technologies\GPC\Workspaces\Polylactide\
Comments	
Created by	Administrator at 13:44:31 on 15 June 2015

Sample Properties

Sample name	MAJ1-172
File name	ICF_18_05_2022-0006.sample
Collected by	GPC at 15:22:46 on 18 May 2022
Instrument name	Instrument 1

Column Calibration Details

Name	PSty May11th2022
Created by	GPC at 16:07:12 on 11 May 2022
Last modified by	GPC at 16:09:11 on 11 May 2022
Comments	GPC Column Calibration created 11 May 2022 by GPC<br>GPC Column Calibration amended 11 May 2022 by GPC<br>GPC Column Calibration amended 11 May 2022 by GPC<br>GPC Column Calibration amended 11 May 2022 by GPC

Calibration Type	Narrow Standard	Curve Fit Used	3
Calibration Curve	y = - 0.00209x^3 + 0.09348x^2 - 1.763x + 16.4		

High Limit MW RT (mins)	11.18333	Low Limit MW RT (mins)	17.98333
High Limit MW (g/mol)	283800	Low Limit MW (g/mol)	580
Flow Rate Marker Name		Flow Marker RT (mins)	0.00000
K (Input) ((10e-5) dL/g)	14.100		
Alpha (Input)	0.700		
Residual Sum Of Squares	0.00170957	Corrected Sum Of Squares	6.9722
Coeff. Of Determination	0.999755	Standard Y Error Estimate	0.0168798
Linear Correlation Coeff	-0.999649		

Column Calibration Data Points

Point	Peak Max RT (mins)	MW	Log MW	Point in Use?	Percent Error
1	11.18333	283800	5.45	Yes	2.07
2	11.88333	135700	5.13	Yes	-0.52
3	12.55000	67600	4.83	Yes	-6.63
4	13.56667	29460	4.47	Yes	2.25
5	13.90000	22390	4.35	Yes	3.82
6	14.85000	9820	3.99	Yes	2.84
7	15.58333	4910	3.69	Yes	-3.89
8	16.16667	3050	3.48	Yes	-0.93
9	17.16667	1250	3.10	Yes	-0.35
10	17.98333	580	2.76	Yes	0.89

Analyst: .... **Date:** ....

Checked By: .... **Date:** ....

Agilent GPC/SEC Software
Sample GPC Analysis Report

Agilent Technologies

Processing Parameters

Method	Last modified by Administrator at 13:44:30 on 15 June 2015
Using Flow Rate Correction	No
Mark-Houwink K ((10e-5) dL/g)	14.100
Mark-Houwink Alpha	0.700
Concentration Detector Used in Analysis	RI
Injection volume (µL)	100.00
Flow rate (mL/min)	1.00

MW Ranges Method

Calculate MW Ranges	No

Percentage Fractions Method

Calculate Percentage Fractions	No

Results

Analysed by	GPC at 19:05:25 on 18 May 2022
Comments	

Molecular Weight Averages

Peak	Mp (g/mol)	Mn (g/mol)	Mw (g/mol)	Mz (g/mol)	Mz+1 (g/mol)	Mv (g/mol)	PD
Peak 1	565	774	989	1413	2181	1327	1.278

Peak Information

	Start (mins)	End (mins)
Baseline region 1	5.86667	7.00000
Baseline region 2	32.23333	32.73333
Peak 1	15.15000	18.36667

Peak Trace Information

Peak	Trace	Peak Max RT (mins)	Peak Area (mV.s)	Peak Height (mV)
Peak 1	RI	18.01667	2796.192	53.670
Peak 1	VS DP	18.00000	1026.818	12.024
Peak 1	VS IP	17.95000	22.654	0.455
Peak 1	LS 90°	18.01667	115.790	1.321
Peak 1	LS 15°	17.50000	33.502	0.476

Analyst: .... **Date:** ....

Checked By: .... **Date:** ....

Agilent GPC/SEC Software
Sample GPC Analysis Report

Agilent Technologies

Chromatogram Plot

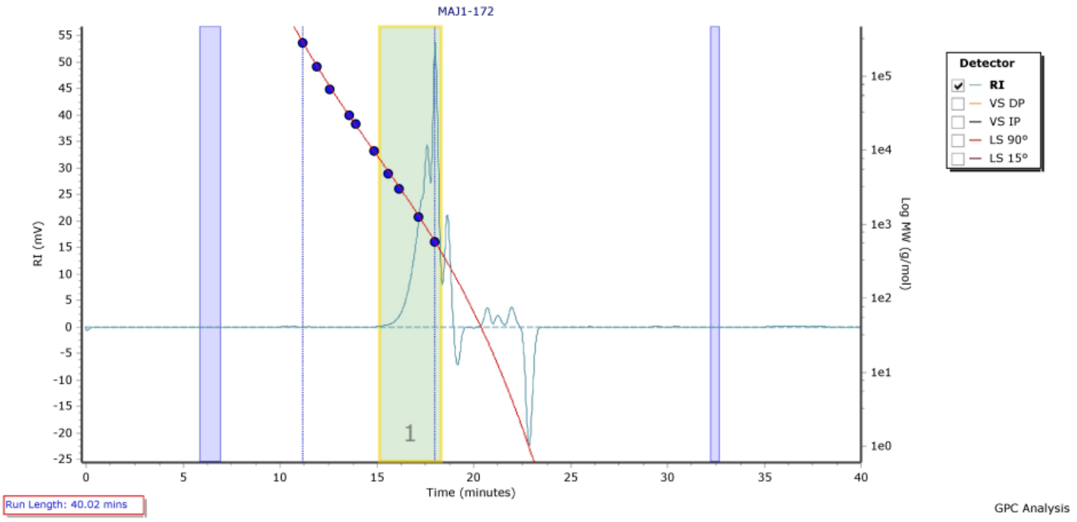

Analyst: .... **Date:** ....

Checked By: .... **Date:** ....

Agilent GPC/SEC Software
Sample GPC Analysis Report

Agilent Technologies

Distribution Plot

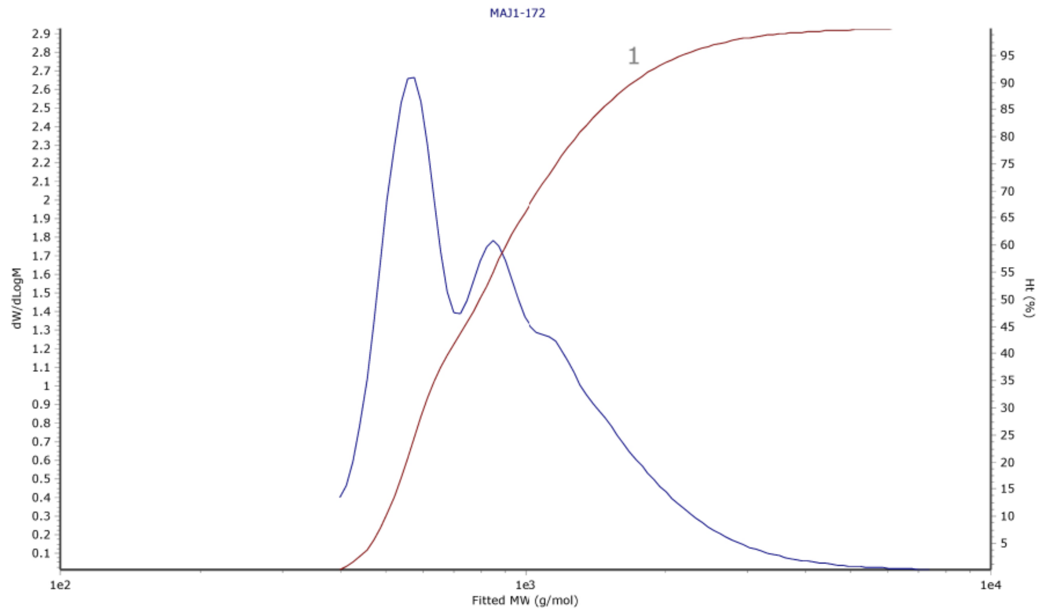

Analyst: .... **Date:** ....

Checked By: .... **Date:** ....

Agilent GPC/SEC Software A.02.01 [9] Page 4 of 4

Generated by GPC at 19:20 on 18 May 2022

Figure S58: GPC data of the crude product formed from the blank polymerization reaction of **2d** under a dynamic vacuum without the presence of **1**.

GPC monitored chain growth experiment

Agilent GPC/SEC Software
Sample GPC Analysis Report

Agilent Technologies

MAJ1-152_1(1H)

Workspace Details

Workspace name	Polylactide
Location	C:\ProgramData\Agilent Technologies\GPC\Workspaces\Polylactide\
Comments	
Created by	Administrator at 13:44:31 on 15 June 2015

Sample Properties

Sample name	MAJ1-152_1(1H)
File name	ICF_23_02_2022-0012.sample
Collected by	GPC at 13:50:05 on 24 February 2022
Instrument name	Instrument 1

Column Calibration Details

Name	PSty Jan 2022
Created by	GPC at 07:21:08 on 05 January 2022
Last modified by	GPC at 07:23:45 on 05 January 2022
Comments	GPC Column Calibration created 05 January 2022 by GPC<br>GPC Column Calibration amended 05 January 2022 by GPC<br>GPC Column Calibration amended 05 January 2022 by GPC<br>GPC Column Calibration amended 05 January 2022 by GPC

Calibration Type	Narrow Standard	Curve Fit Used	3
Calibration Curve	y = - 0.003181x^3 + 0.1419x^2 - 2.499x + 20.12		
High Limit MW RT (mins)	11.25000	Low Limit MW RT (mins)	17.58333
High Limit MW (g/mol)	283800	Low Limit MW (g/mol)	580
Flow Rate Marker Name		Flow Marker RT (mins)	0.00000
K (Input) ((10e-5) dL/g)	14.100		
Alpha (Input)	0.700		
Residual Sum Of Squares	0.00186199	Corrected Sum Of Squares	6.97134
Coeff. Of Determination	0.999733	Standard Y Error Estimate	0.0176162
Linear Correlation Coeff	-0.999415		

Column Calibration Data Points

Point	Peak Max RT (mins)	MW	Log MW	Point in Use?	Percent Error
1	11.25000	283800	5.45	Yes	1.92
2	11.85000	135700	5.13	Yes	-3.66
3	12.55000	67600	4.83	Yes	0.37
4	13.43333	29460	4.47	Yes	3.26
5	13.66667	22290	4.35	Yes	-2.84
6	14.65000	9820	3.99	Yes	4.37
7	15.35000	4910	3.69	Yes	-2.15
8	15.85000	3050	3.48	Yes	-4.49
9	16.88333	1250	3.10	Yes	4.07
10	17.58333	580	2.76	Yes	-1.35

Analyst: .... **Date:** ....

Checked By: .... **Date:** ....

Agilent GPC/SEC Software
Sample GPC Analysis Report

Agilent Technologies

Processing Parameters

Method	Last modified by Administrator at 13:44:30 on 15 June 2015
Using Flow Rate Correction	No
Mark-Houwink K ((10e-5) dL/g)	14.100
Mark-Houwink Alpha	0.700
Concentration Detector Used in Analysis	RI
Injection volume (µL)	100.00
Flow rate (mL/min)	1.00

MW Ranges Method

Calculate MW Ranges No

Percentage Fractions Method

Calculate Percentage Fractions No

Results

Analysed by GPC at 16:17:11 on 24 February 2022

Comments

Molecular Weight Averages

Peak	Mp (g/mol)	Mn (g/mol)	Mw (g/mol)	Mz (g/mol)	Mz+1 (g/mol)	Mv (g/mol)	PD
Peak 1	2611	2146	3388	5343	7721	5022	1.579

Peak Information

	Start (mins)	End (mins)
Baseline region 1	7.76667	8.30000
Baseline region 2	32.61667	33.11667
Peak 1	13.56667	17.35000

Peak Trace Information

Peak	Trace	Peak Max RT (mins)	Peak Area (mV.s)	Peak Height (mV)
Peak 1	RI	16.08333	446.654	3.590
Peak 1	VS DP	16.00000	222.586	1.703
Peak 1	VS IP	14.51667	11.184	-0.637
Peak 1	LS 90°	15.51667	40.370	0.338
Peak 1	LS 15°	15.63333	79.384	0.423

Analyst: .... **Date:** ....

Checked By: .... **Date:** ....

Chromatogram Plot

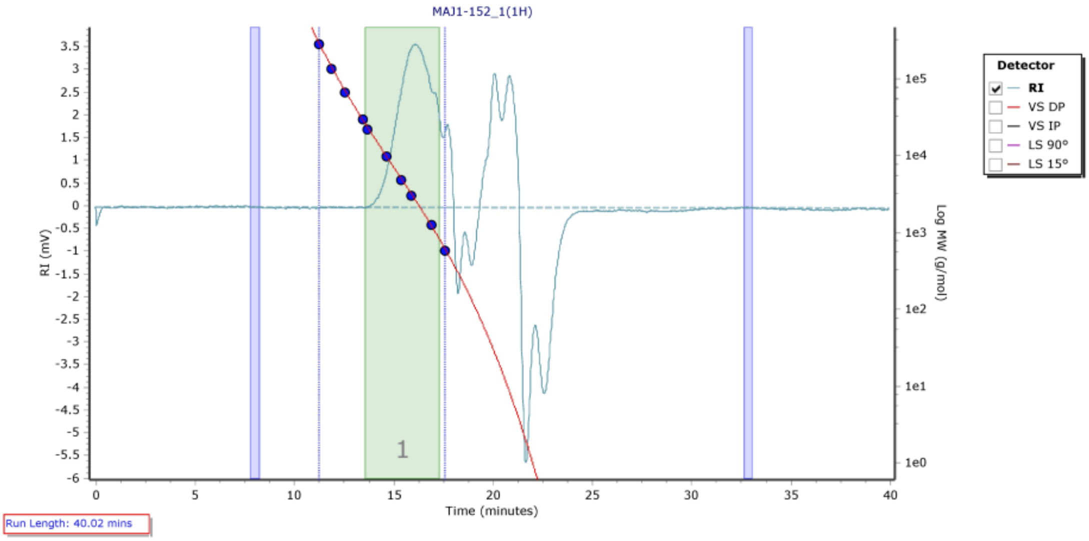

Analyst: .... **Date:** ....

Checked By: .... **Date:** ....

Agilent GPC/SEC Software
Sample GPC Analysis Report

Distribution Plot

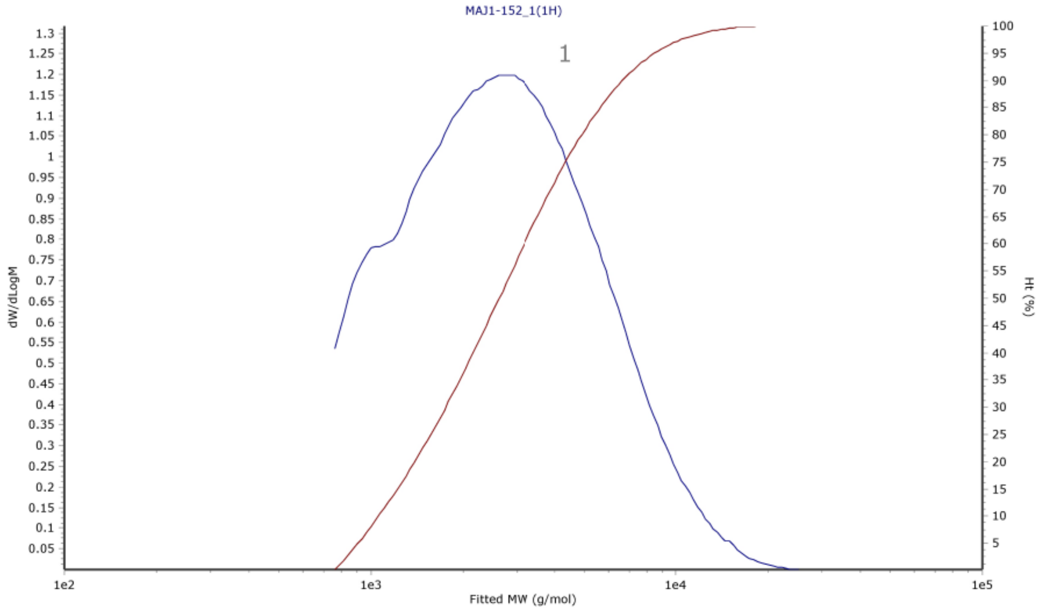

Analyst: .... **Date:** ....

Checked By: .... **Date:** ....

Agilent GPC/SEC Software A.02.01 [9]

Page 4 of 4

Generated by GPC at 16:17 on 24 February 2022

Figure S59: GPC data of the crude product of the dehydrocoupling reaction of **2a** at a 1 h timepoint.

MAJ1-152_3(4H)

Workspace Details

Workspace name	Polylactide
Location	C:\ProgramData\Agilent Technologies\GPC\Workspaces\Polylactide\
Comments	
Created by	Administrator at 13:44:31 on 15 June 2015

Sample Properties

Sample name	MAJ1-152_3(4H)
File name	ICF_23_02_2022-0014.sample
Collected by	GPC at 15:13:44 on 24 February 2022
Instrument name	Instrument 1

Column Calibration Details

Name	PSty Jan 2022
Created by	GPC at 07:21:08 on 05 January 2022
Last modified by	GPC at 07:23:45 on 05 January 2022
Comments	GPC Column Calibration created 05 January 2022 by GPC<br>GPC Column Calibration amended 05 January 2022 by GPC<br>GPC Column Calibration amended 05 January 2022 by GPC<br>GPC Column Calibration amended 05 January 2022 by GPC

Calibration Type	Narrow Standard	Curve Fit Used	3
Calibration Curve	y = - 0.003181x^3 + 0.1419x^2 - 2.499x + 20.12		
High Limit MW RT (mins)	11.25000	Low Limit MW RT (mins)	17.58333
High Limit MW (g/mol)	283800	Low Limit MW (g/mol)	580
Flow Rate Marker Name		Flow Marker RT (mins)	0.00000
K (Input) ((10e-5) dL/g)	14.100		
Alpha (Input)	0.700		
Residual Sum Of Squares	0.00186199	Corrected Sum Of Squares	6.97134
Coeff. Of Determination	0.999733	Standard Y Error Estimate	0.0176162
Linear Correlation Coeff	-0.999415		

Column Calibration Data Points

Point	Peak Max RT (mins)	MW	Log MW	Point in Use?	Percent Error
1	11.25000	283800	5.45	Yes	1.92
2	11.85000	135700	5.13	Yes	-3.66
3	12.55000	67600	4.83	Yes	0.37
4	13.43333	29460	4.47	Yes	3.26
5	13.66667	22290	4.35	Yes	-2.84
6	14.65000	9820	3.99	Yes	4.37
7	15.35000	4910	3.69	Yes	-2.15
8	15.85000	3050	3.48	Yes	-4.49
9	16.88333	1250	3.10	Yes	4.07
10	17.58333	580	2.76	Yes	-1.35

Analyst: .... **Date:** ....

Checked By: .... **Date:** ....

Agilent GPC/SEC Software
Sample GPC Analysis Report

Agilent Technologies

Processing Parameters

Method	Last modified by Administrator at 13:44:30 on 15 June 2015
Using Flow Rate Correction	No
Mark-Houwink K ((10e-5) dL/g)	14.100
Mark-Houwink Alpha	0.700
Concentration Detector Used in Analysis	RI
Injection volume (µL)	100.00
Flow rate (mL/min)	1.00

MW Ranges Method

Calculate MW Ranges — No

Percentage Fractions Method

Calculate Percentage Fractions — No

Results

Analysed by — GPC at 16:20:02 on 24 February 2022

Comments

Molecular Weight Averages

Peak	Mp (g/mol)	Mn (g/mol)	Mw (g/mol)	Mz (g/mol)	Mz+1 (g/mol)	Mv (g/mol)	PD
Peak 1	7852	4505	9254	17781	30692	16283	2.054

Peak Information

	Start (mins)	End (mins)
Baseline region 1	4.06667	5.01667
Baseline region 2	30.26667	31.60000
Peak 1	11.96667	16.93333

Peak Trace Information

Peak	Trace	Peak Max RT (mins)	Peak Area (mV.s)	Peak Height (mV)
Peak 1	RI	14.86667	1387.055	9.038
Peak 1	VS DP	14.51667	1418.435	8.607
Peak 1	VS IP	14.83333	31.056	0.565
Peak 1	LS 90°	14.20000	347.029	2.607
Peak 1	LS 15°	14.10000	156.130	1.153

Analyst: .... **Date:** ....

Checked By: .... **Date:** ....

Chromatogram Plot

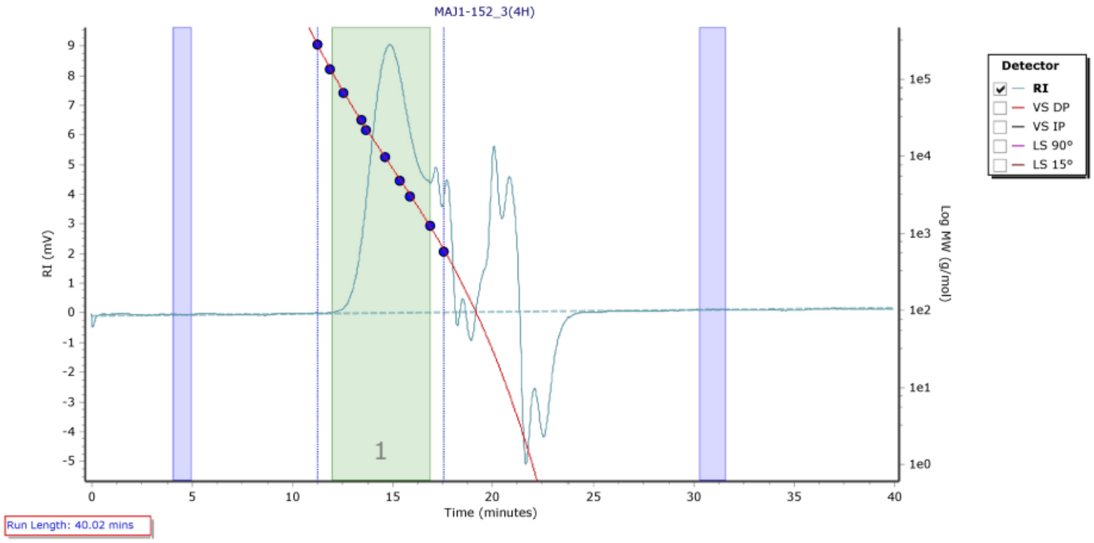

Analyst: .... **Date:** ....

Checked By: .... **Date:** ....

Agilent GPC/SEC Software
Sample GPC Analysis Report

Agilent Technologies

Distribution Plot

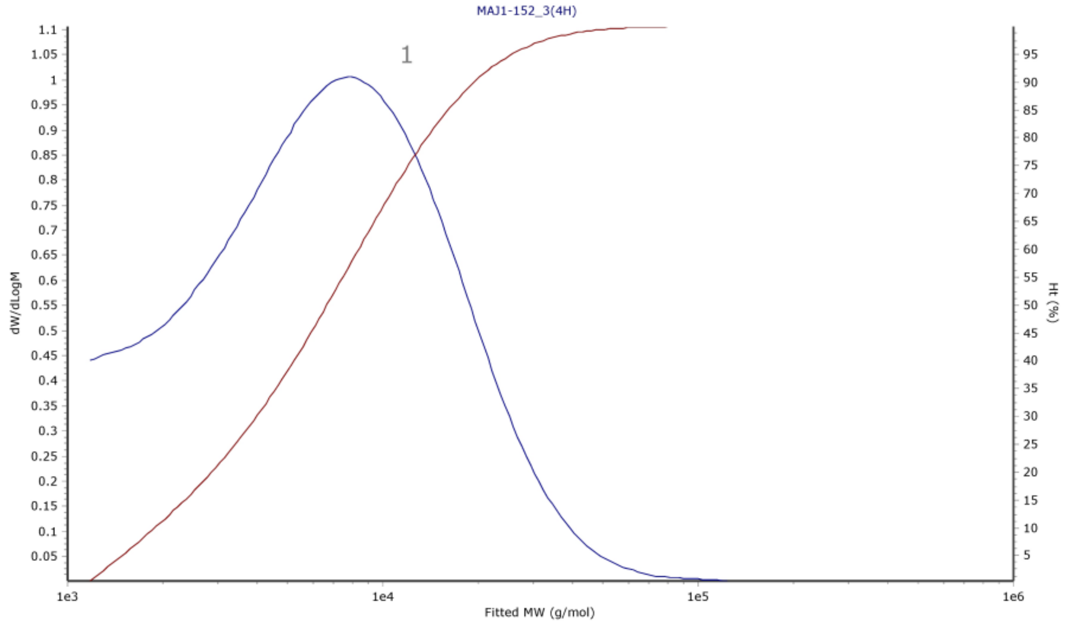

Analyst: .... **Date:** ....

Checked By: .... **Date:** ....

Agilent GPC/SEC Software A.02.01 [9] Page 4 of 4

Generated by GPC at 16:20 on 24 February 2022

Figure S60: GPC data of the crude product of the dehydrocoupling reaction of **2a** at a 4 h timepoint.

Agilent GPC/SEC Software
Sample GPC Analysis Report

Agilent Technologies

MAJ1-152_5(8H)

Workspace Details

Workspace name	Polylactide
Location	C:\ProgramData\Agilent Technologies\GPC\Workspaces\Polylactide\
Comments	
Created by	Administrator at 13:44:31 on 15 June 2015

Sample Properties

Sample name	MAJ1-152_5(8H)
File name	ICF_25_02_2022-0008.sample
Collected by	GPC at 14:53:10 on 25 February 2022
Instrument name	Instrument 1

Column Calibration Details

Name	PSty Jan 2022
Created by	GPC at 07:21:08 on 05 January 2022
Last modified by	GPC at 07:23:45 on 05 January 2022
Comments	GPC Column Calibration created 05 January 2022 by GPC<br>GPC Column Calibration amended 05 January 2022 by GPC<br>GPC Column Calibration amended 05 January 2022 by GPC<br>GPC Column Calibration amended 05 January 2022 by GPC

Calibration Type	Narrow Standard	Curve Fit Used	3
Calibration Curve	y = - 0.003181x^3 + 0.1419x^2 - 2.499x + 20.12		
High Limit MW RT (mins)	11.25000	Low Limit MW RT (mins)	17.58333
High Limit MW (g/mol)	283800	Low Limit MW (g/mol)	580
Flow Rate Marker Name		Flow Marker RT (mins)	0.00000
K (Input) ((10e-5) dL/g)	14.100		
Alpha (Input)	0.700		
Residual Sum Of Squares	0.00186199	Corrected Sum Of Squares	6.97134
Coeff. Of Determination	0.999733	Standard Y Error Estimate	0.0176162
Linear Correlation Coeff	-0.999415		

Column Calibration Data Points

Point	Peak Max RT (mins)	MW	Log MW	Point in Use?	Percent Error
1	11.25000	283800	5.45	Yes	1.92
2	11.85000	135700	5.13	Yes	-3.66
3	12.55000	67600	4.83	Yes	0.37
4	13.43333	29460	4.47	Yes	3.26
5	13.66667	22290	4.35	Yes	-2.84
6	14.65000	9820	3.99	Yes	4.37
7	15.35000	4910	3.69	Yes	-2.15
8	15.85000	3050	3.48	Yes	-4.49
9	16.88333	1250	3.10	Yes	4.07
10	17.58333	580	2.76	Yes	-1.35

Analyst: .... **Date:** ....

Checked By: .... **Date:** ....

Processing Parameters

Method	Last modified by Administrator at 13:44:30 on 15 June 2015
Using Flow Rate Correction	No
Mark-Houwink K ((10e-5) dL/g)	14.100
Mark-Houwink Alpha	0.700
Concentration Detector Used in Analysis	RI
Injection volume (µL)	100.00
Flow rate (mL/min)	1.00

MW Ranges Method

Calculate MW Ranges — No

Percentage Fractions Method

Calculate Percentage Fractions — No

Results

Analysed by — GPC at 17:04:19 on 25 February 2022

Comments

Molecular Weight Averages

Peak	Mp (g/mol)	Mn (g/mol)	Mw (g/mol)	Mz (g/mol)	Mz+1 (g/mol)	Mv (g/mol)	PD
Peak 1	36125	20256	134460	3233498	13795612	2196275	6.638

Peak Information

	Start (mins)	End (mins)
Baseline region 1	2.96667	3.31667
Baseline region 2	32.68333	33.46667
Peak 1	8.20000	15.91667

Peak Trace Information

Peak	Trace	Peak Max RT (mins)	Peak Area (mV.s)	Peak Height (mV)
Peak 1	RI	13.20000	1265.254	6.148
Peak 1	VS DP	12.38333	3202.287	15.538
Peak 1	VS IP	14.96667	34.053	-0.587
Peak 1	LS 90°	11.05000	2557.230	13.118
Peak 1	LS 15°	11.11667	767.532	3.910

Analyst: .... **Date:** ....

Checked By: .... **Date:** ....

Chromatogram Plot

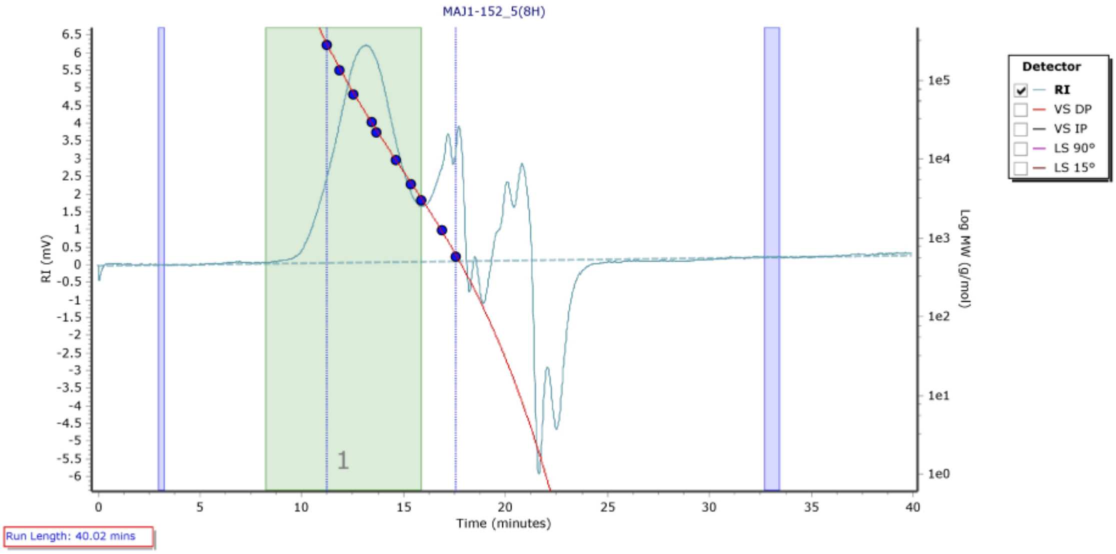

Analyst: .... **Date:** ....

Checked By: .... **Date:** ....

Agilent GPC/SEC Software
Sample GPC Analysis Report

Agilent Technologies

Distribution Plot

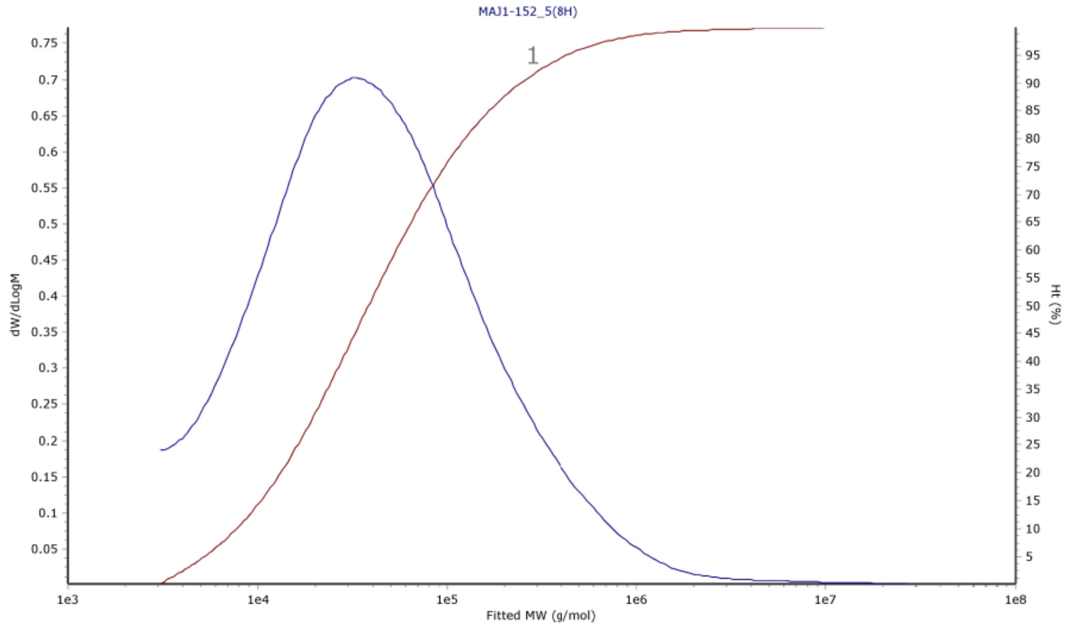

Analyst: .... **Date:** ....

Checked By: .... **Date:** ....

Agilent GPC/SEC Software A.02.01 [9] Page 4 of 4

Generated by GPC at 17:04 on 25 February 2022

Figure S61: GPC data of the crude product of the dehydrocoupling reaction of **2a** at a 8 h timepoint.

Agilent GPC/SEC Software
Sample GPC Analysis Report

Agilent Technologies

MAJ1-152_6(15.5H)

Workspace Details

Workspace name	Polylactide
Location	C:\ProgramData\Agilent Technologies\GPC\Workspaces\Polylactide\
Comments	
Created by	Administrator at 13:44:31 on 15 June 2015

Sample Properties

Sample name	MAJ1-152_6(15.5H)
File name	ICF_25_02_2022-0009.sample
Collected by	GPC at 15:35:02 on 25 February 2022
Instrument name	Instrument 1

Column Calibration Details

Name	PSty Jan 2022
Created by	GPC at 07:21:08 on 05 January 2022
Last modified by	GPC at 07:23:45 on 05 January 2022
Comments	GPC Column Calibration created 05 January 2022 by GPC<br>GPC Column Calibration amended 05 January 2022 by GPC<br>GPC Column Calibration amended 05 January 2022 by GPC<br>GPC Column Calibration amended 05 January 2022 by GPC

Calibration Type	Narrow Standard	Curve Fit Used	3
Calibration Curve	y = - 0.003181x^3 + 0.1419x^2 - 2.499x + 20.12		
High Limit MW RT (mins)	11.25000	Low Limit MW RT (mins)	17.58333
High Limit MW (g/mol)	283800	Low Limit MW (g/mol)	580
Flow Rate Marker Name		Flow Marker RT (mins)	0.00000
K (Input) ((10e-5) dL/g)	14.100		
Alpha (Input)	0.700		
Residual Sum Of Squares	0.00186199	Corrected Sum Of Squares	6.97134
Coeff. Of Determination	0.999733	Standard Y Error Estimate	0.0176162
Linear Correlation Coeff	-0.999415		

Column Calibration Data Points

Point	Peak Max RT (mins)	MW	Log MW	Point in Use?	Percent Error
1	11.25000	283800	5.45	Yes	1.92
2	11.85000	135700	5.13	Yes	-3.66
3	12.55000	67600	4.83	Yes	0.37
4	13.43333	29460	4.47	Yes	3.26
5	13.66667	22290	4.35	Yes	-2.84
6	14.65000	9820	3.99	Yes	4.37
7	15.35000	4910	3.69	Yes	-2.15
8	15.85000	3050	3.48	Yes	-4.49
9	16.88333	1250	3.10	Yes	4.07
10	17.58333	580	2.76	Yes	-1.35

Analyst: .... **Date:** ....

Checked By: .... **Date:** ....

Agilent GPC/SEC Software
Sample GPC Analysis Report

Agilent Technologies

Processing Parameters

Method	Last modified by Administrator at 13:44:30 on 15 June 2015
Using Flow Rate Correction	No
Mark-Houwink K ((10e-5) dL/g)	14.100
Mark-Houwink Alpha	0.700
Concentration Detector Used in Analysis	RI
Injection volume (µL)	100.00
Flow rate (mL/min)	1.00

MW Ranges Method

Calculate MW Ranges	No

Percentage Fractions Method

Calculate Percentage Fractions	No

Results

Analysed by	GPC at 17:05:16 on 25 February 2022
Comments	

Molecular Weight Averages

Peak	Mp (g/mol)	Mn (g/mol)	Mw (g/mol)	Mz (g/mol)	Mz+1 (g/mol)	Mv (g/mol)	PD
Peak 1	67349	27227	201615	1755192	7441574	1322451	7.405

Peak Information

	Start (mins)	End (mins)
Baseline region 1	5.45000	6.61667
Baseline region 2	27.60000	28.75000
Peak 1	8.33333	15.91667

Peak Trace Information

Peak	Trace	Peak Max RT (mins)	Peak Area (mV.s)	Peak Height (mV)
Peak 1	RI	12.55000	1875.461	8.790
Peak 1	VS DP	11.70000	8072.520	32.470
Peak 1	VS IP	11.48333	129.915	1.105
Peak 1	LS 90°	10.23333	6976.495	39.773
Peak 1	LS 15°	10.20000	2280.434	13.031

Analyst: .... **Date:** ....

Checked By: .... **Date:** ....

Agilent GPC/SEC Software
Sample GPC Analysis Report

Agilent Technologies

Chromatogram Plot

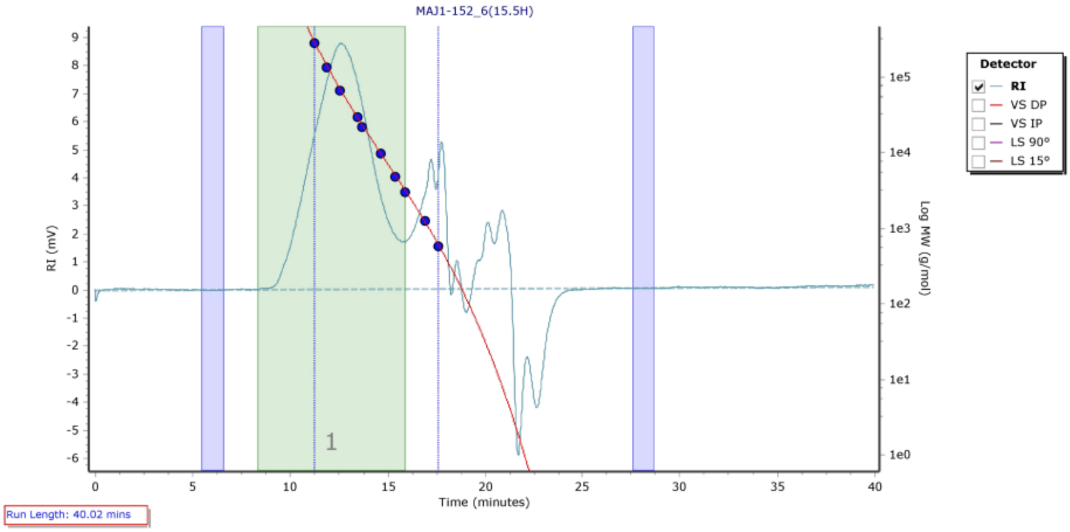

Analyst: .... **Date:** ....

Checked By: .... **Date:** ....

Agilent GPC/SEC Software
Sample GPC Analysis Report

Agilent Technologies

Distribution Plot

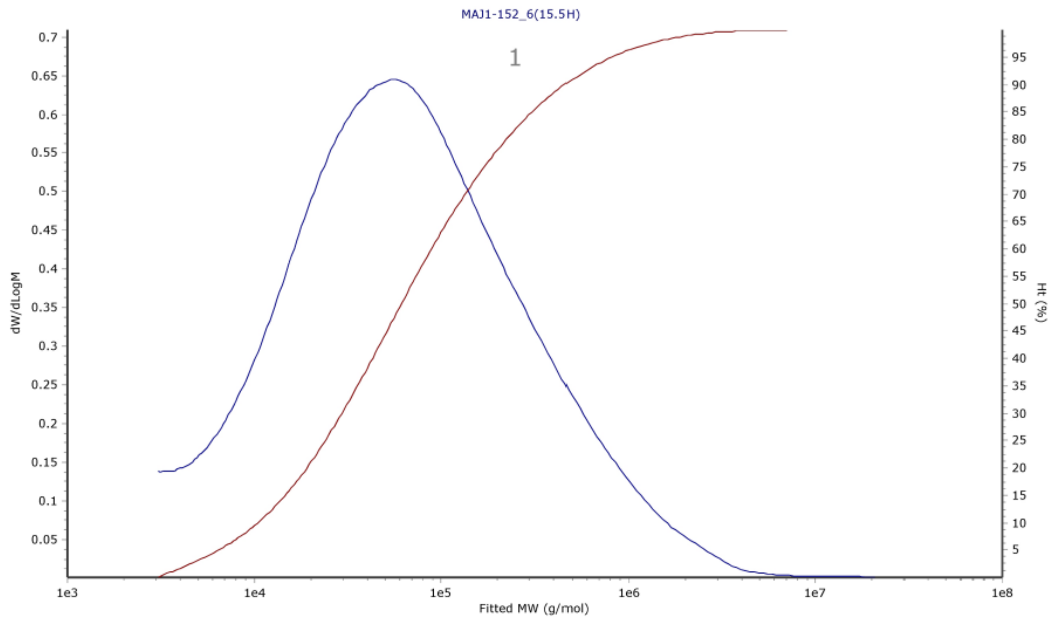

Analyst: .... **Date:** ....

Checked By: .... **Date:** ....

Agilent GPC/SEC Software A.02.01 [9] Page 4 of 4

Generated by GPC at 17:05 on 25 February 2022

Figure S62: GPC data of the crude product of the dehydrocoupling reaction of **2a** at a 16 h timepoint.

Matrix-assisted Laser Desorption/Ionization Time-of-Flight Mass Spectrometry (MALDI-TOF) data

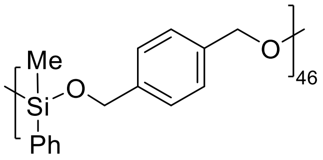

2a

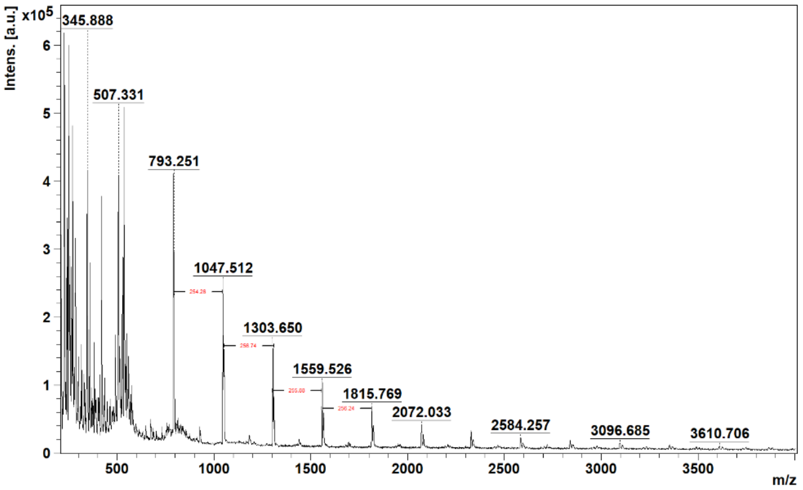

Figure S63: MALDI-TOF data of product **2a**. Obtained using dithranol as the matrix and sodium trifluoroacetate as the cation in a 10:2:1, **2a**:matrix:cation ratio.

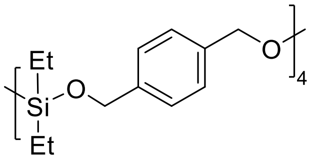

2b

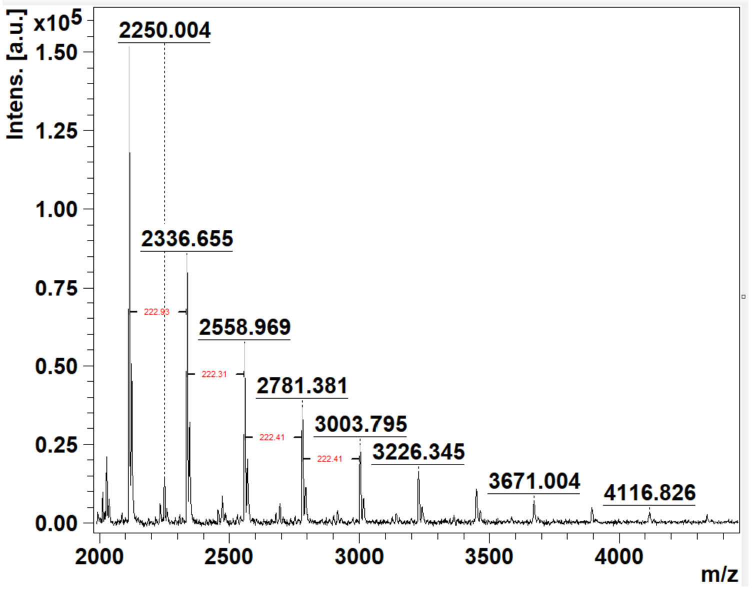

Figure S64: MALDI-TOF data of product **2b**. Obtained using dithranol as the matrix and sodium trifluoroacetate as the cation in a 10:2:1, **2b**:matrix:cation ratio.

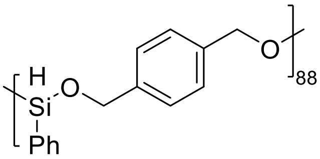

2c

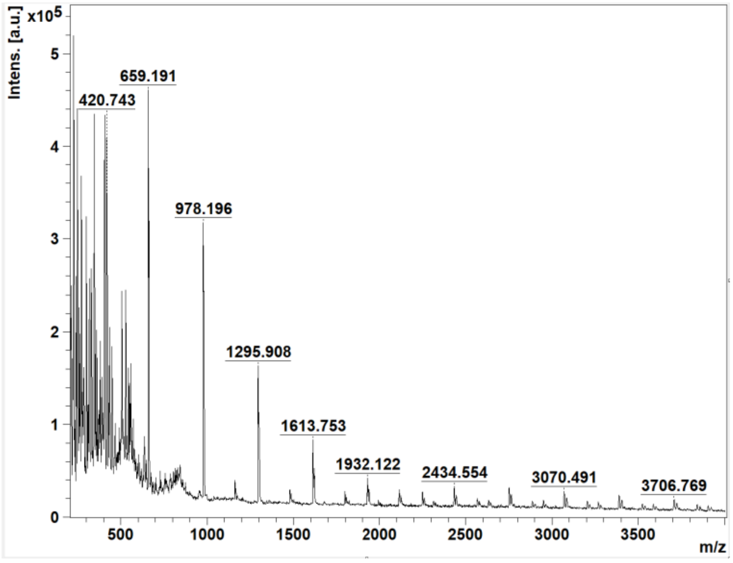

Figure S65: MALDI-TOF data of product **2c**. Obtained using dithranol as the matrix and sodium trifluoroacetate as the cation in a 10:2:1, **2c**:matrix:cation ratio.

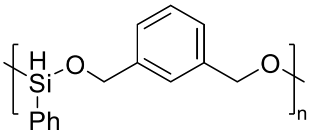

2e

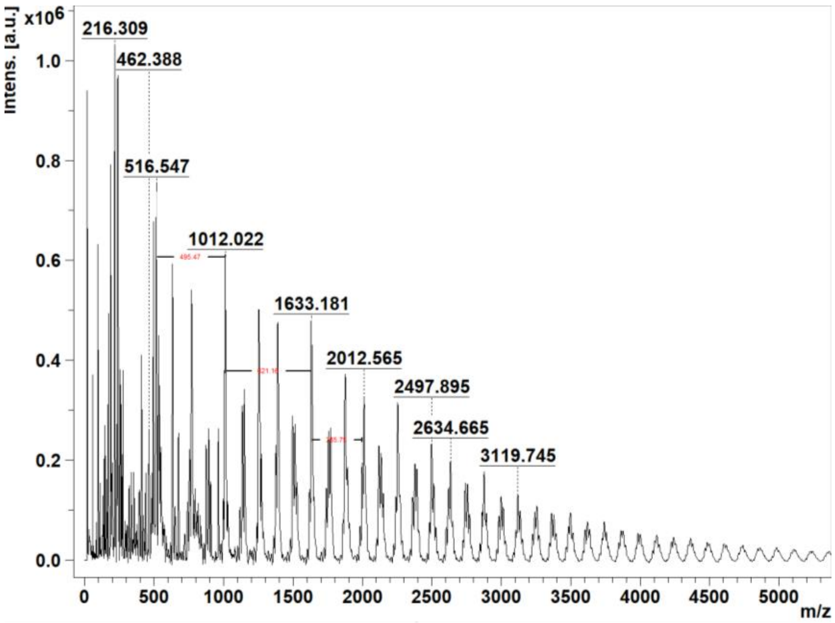

Figure S66: MALDI-TOF data of product **2e**. Obtained using dithranol as the matrix and sodium trifluoroacetate as the cation in a 10:2:1, **2e**:matrix:cation ratio.

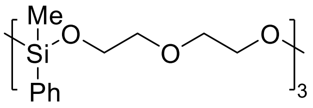

2f

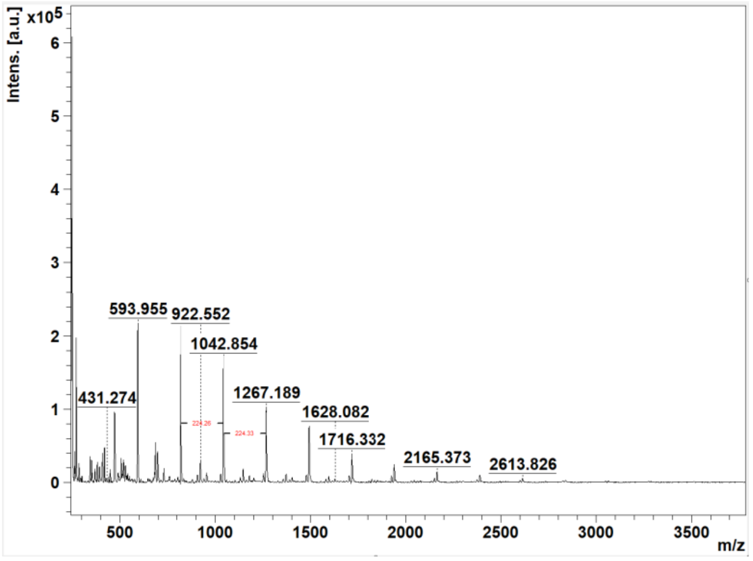

Figure S67: MALDI-TOF data of product **2f**. Obtained using dithranol as the matrix and sodium trifluoroacetate as the cation in a 10:2:1, **2f**:matrix:cation ratio.

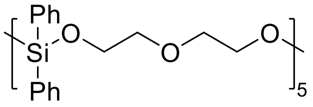

2g

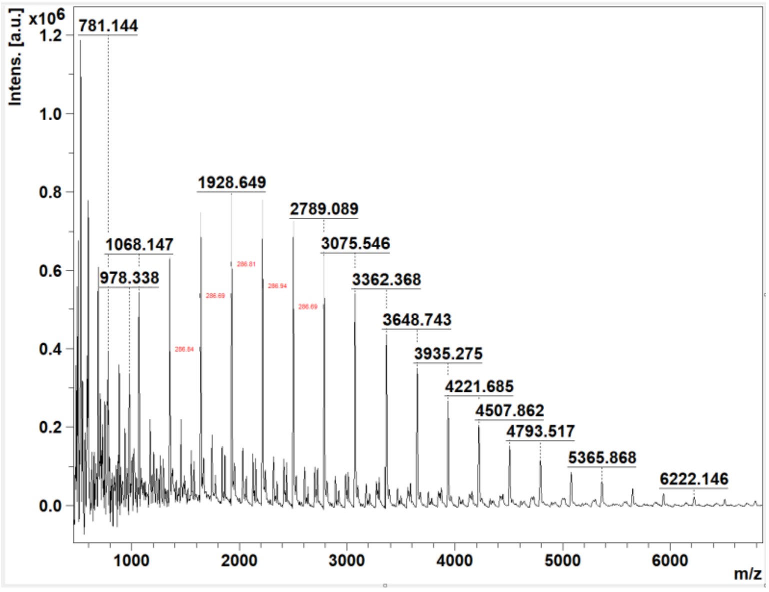

Figure S68: MALDI-TOF data of product **2g**. Obtained using DCTB as the matrix and sodium trifluoroacetate as the cation in a 10:2:1, **2g**:matrix:cation ratio.

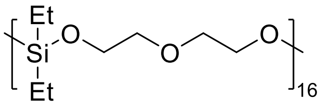

2h

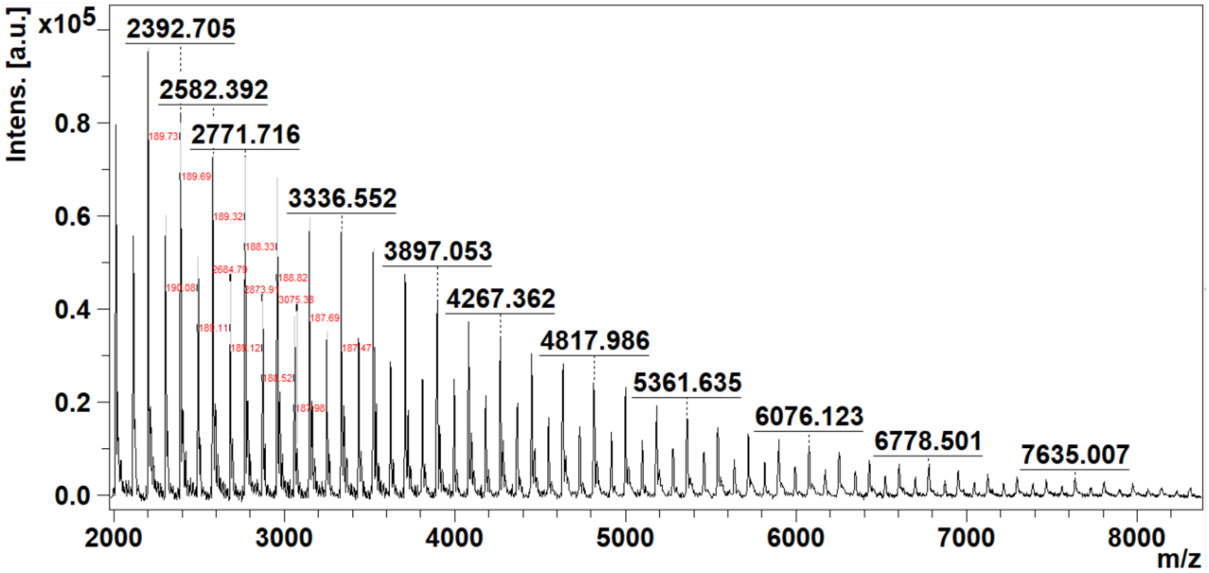

Figure S69: MALDI-TOF data of product **2h**. Obtained using dithranol as the matrix and sodium trifluoroacetate as the cation in a 10:2:1, **2h**:matrix:cation ratio.

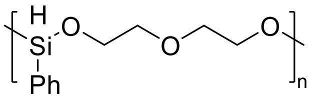

2i

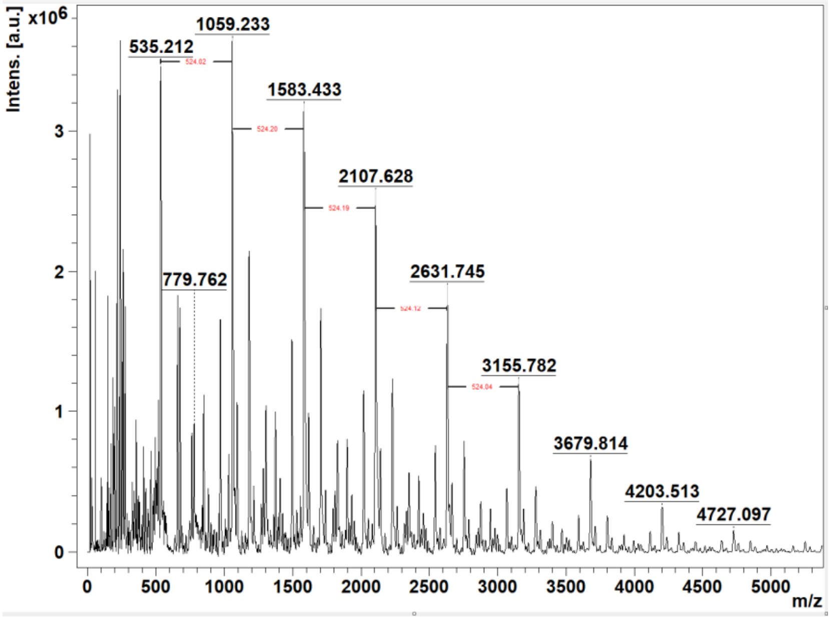

Figure S70: MALDI-TOF data of product **2i**. Obtained using dithranol as the matrix and sodium trifluoroacetate as the cation in a 10:2:1, **2i**:matrix:cation ratio.

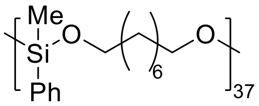

2j

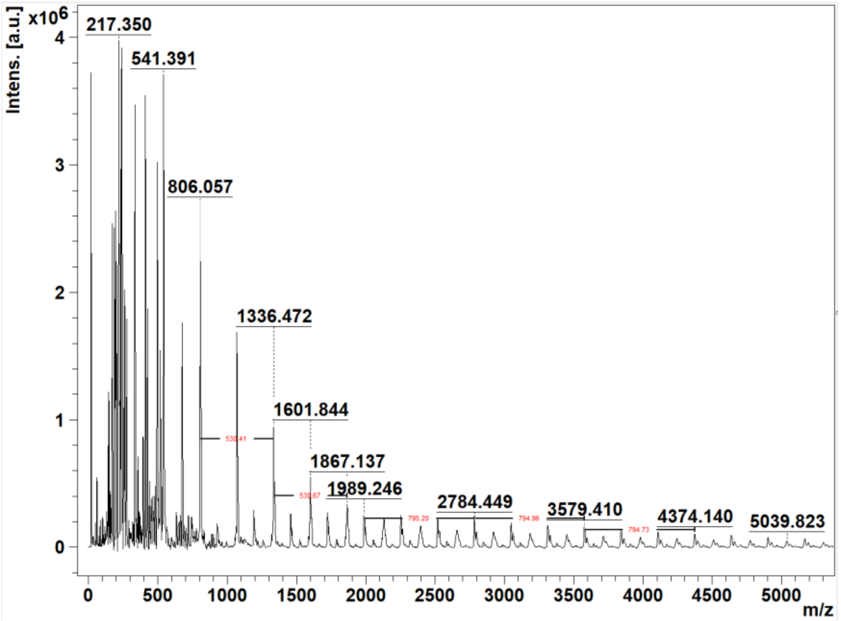

Figure S71: MALDI-TOF data of product **2j**. Obtained using dithranol as the matrix and sodium trifluoroacetate as the cation in a 10:2:1, **2j**:matrix:cation ratio.

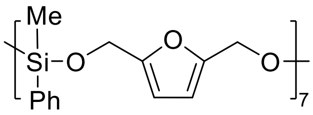

2n

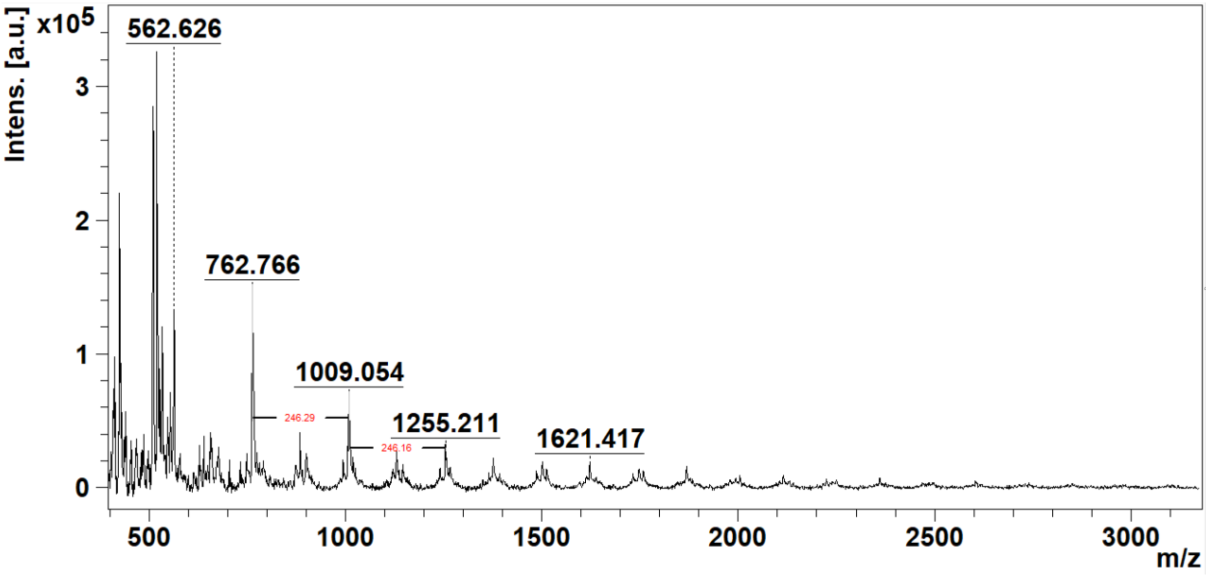

Figure S72: MALDI-TOF data of product **2n**. Obtained using dithranol as the matrix and sodium trifluoroacetate as the cation in a 10:2:1, **2n**:matrix:cation ratio.

Fourier-transform infrared (FTIR) Spectroscopy Data of 2a-2n

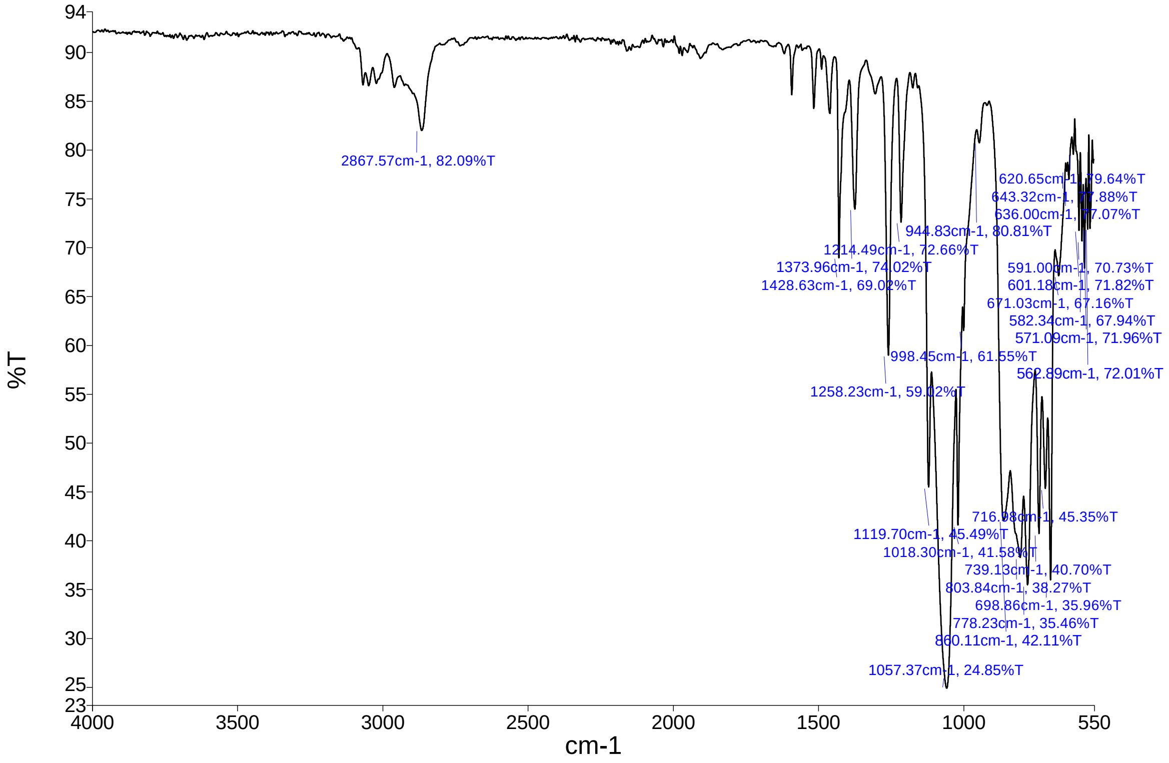

Figure S73: FTIR spectrum of **2a**.

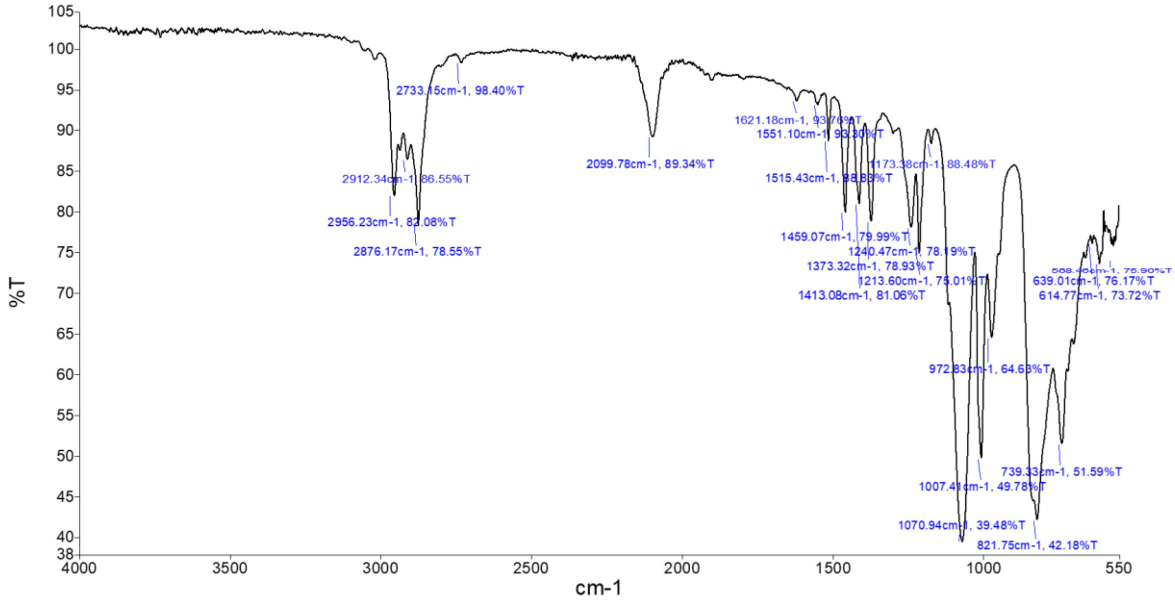

Figure S74: FTIR spectrum of **2b**.

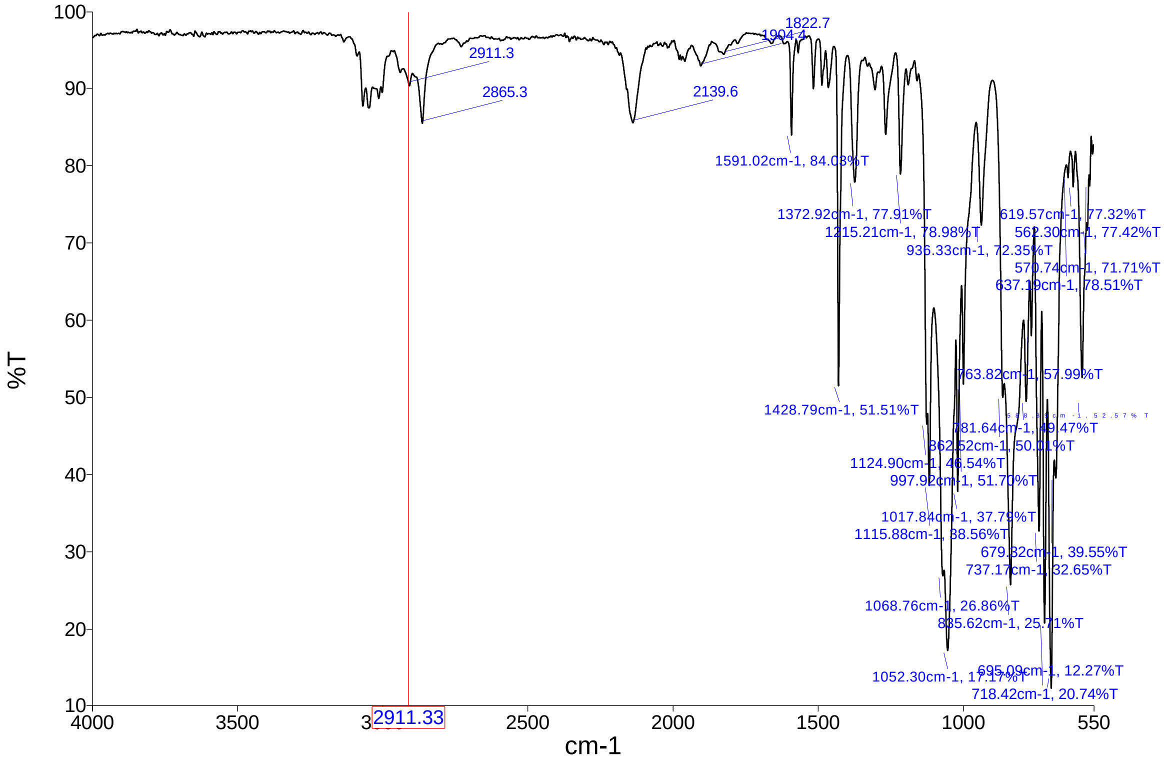

Figure S75: FTIR spectrum of **2c**.

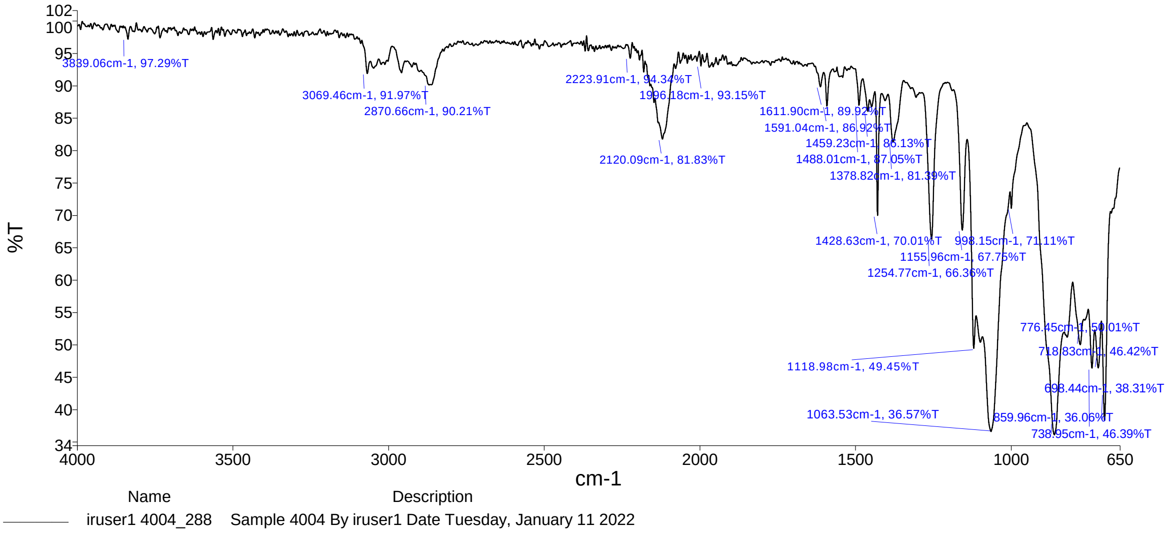

Figure S76: FTIR spectrum of **2d**.

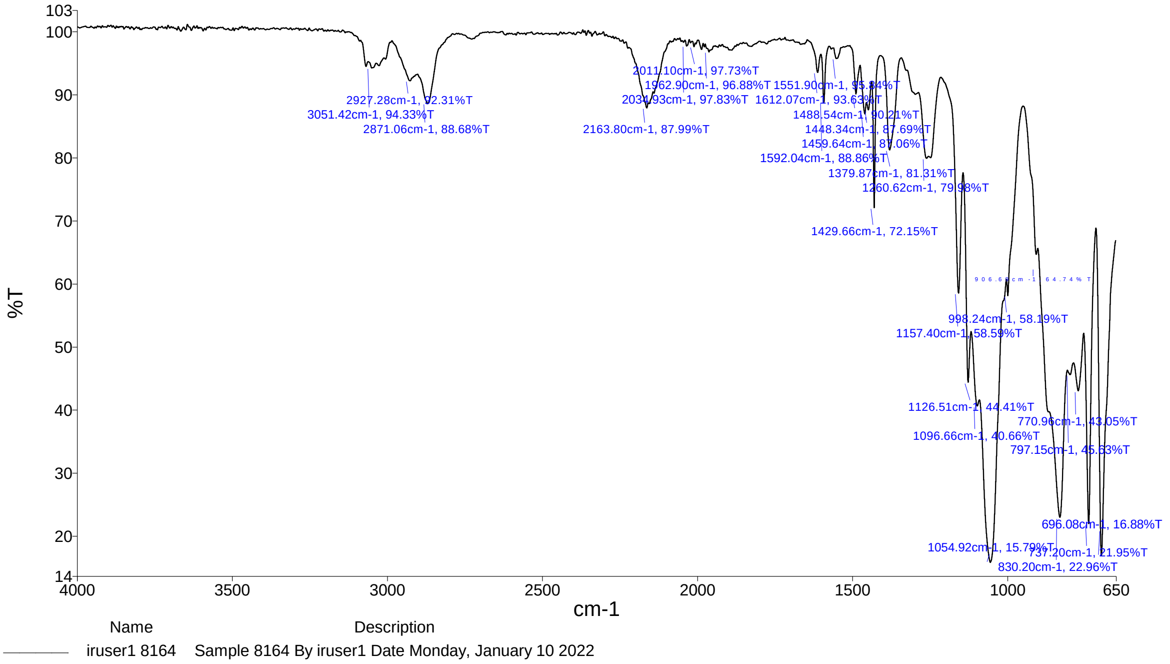

Figure S77: FTIR spectrum of **2e**.

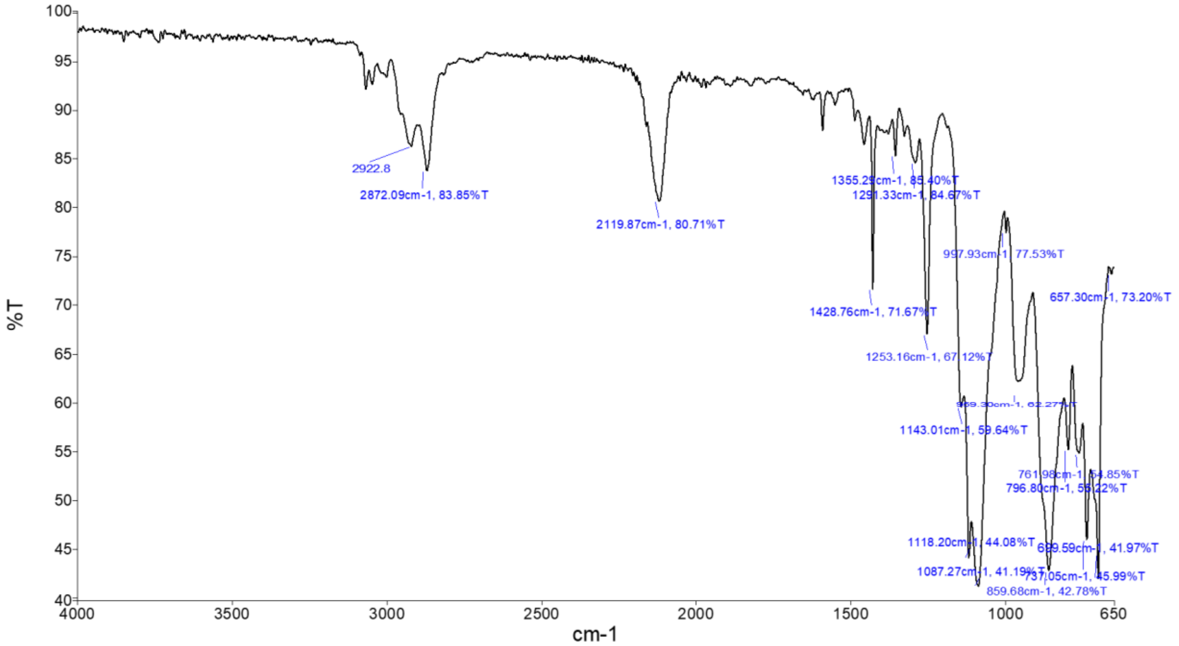

Figure S78: FTIR spectrum of **2f**.

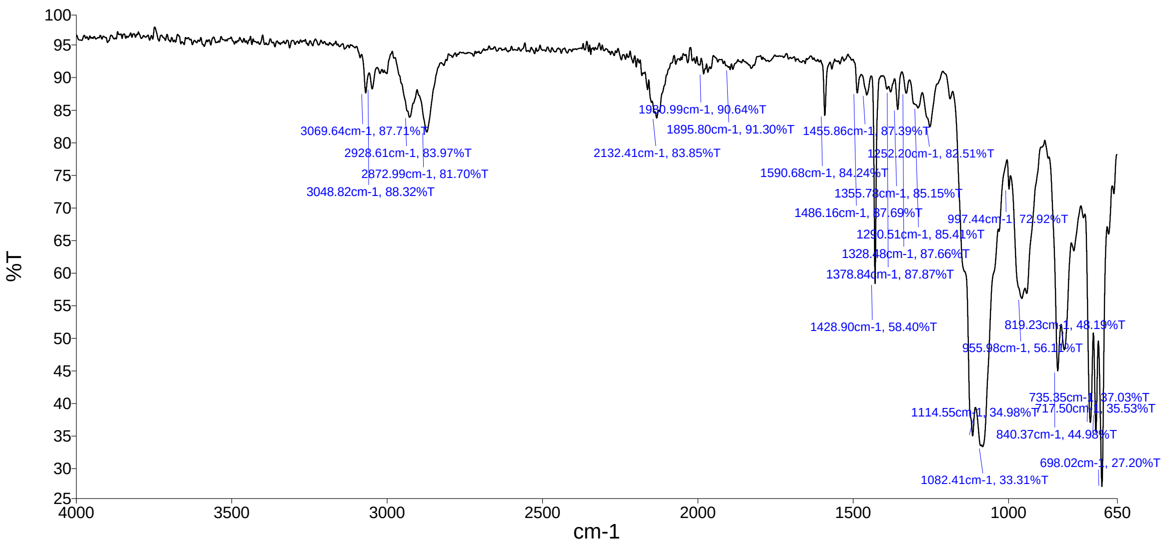

Figure S79: FTIR spectrum of **2g**.

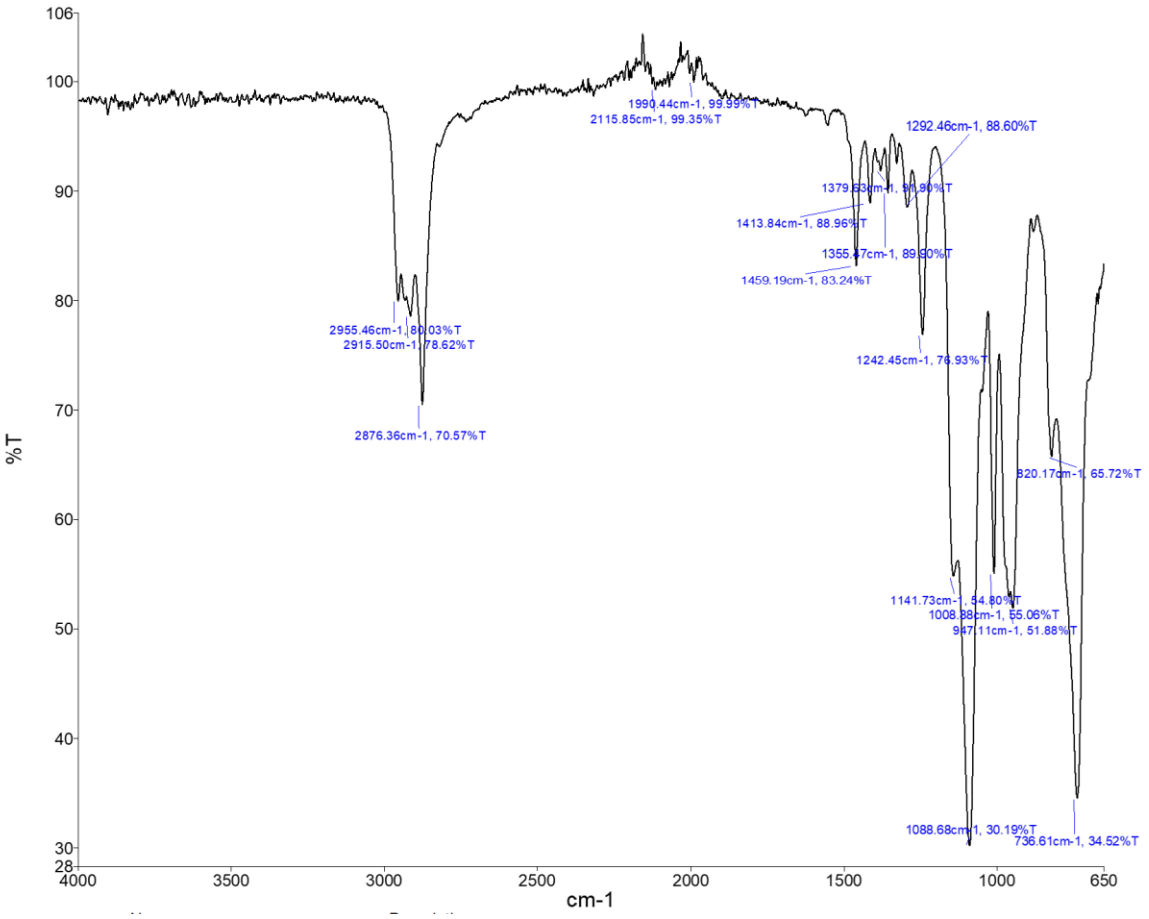

Figure S80: FTIR spectrum of **2h**.

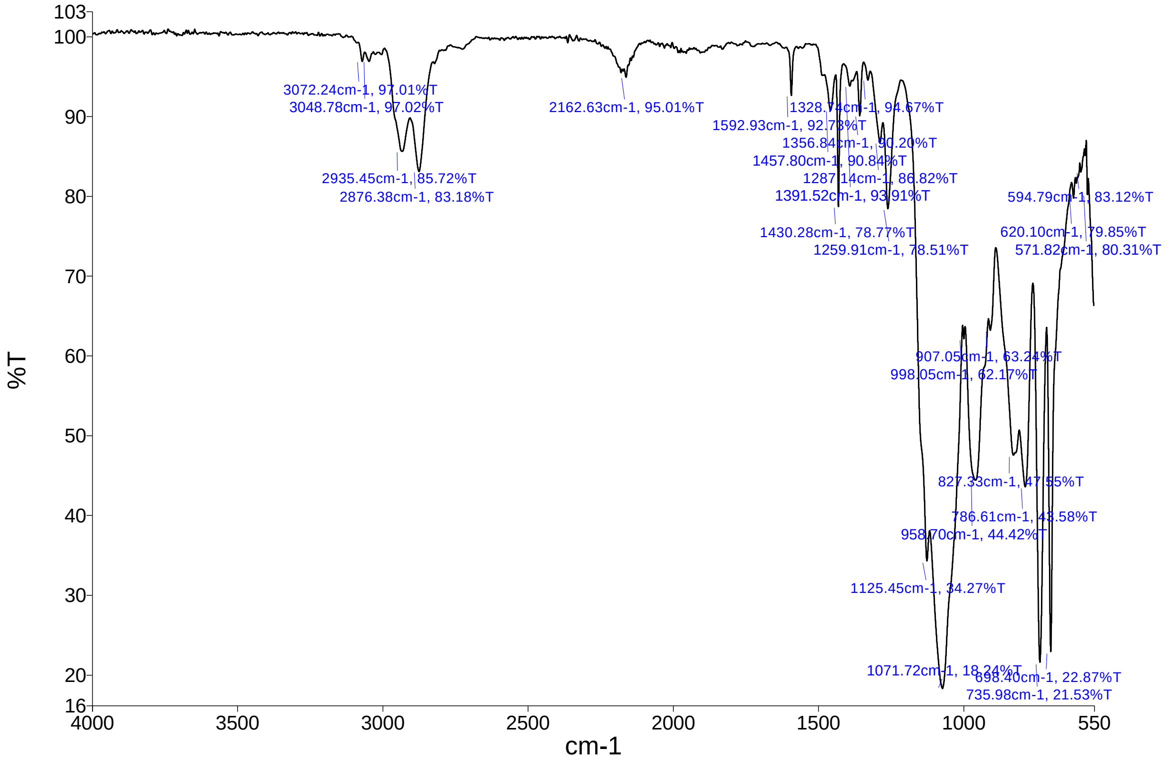

Figure S81: FTIR spectrum of **2i**.

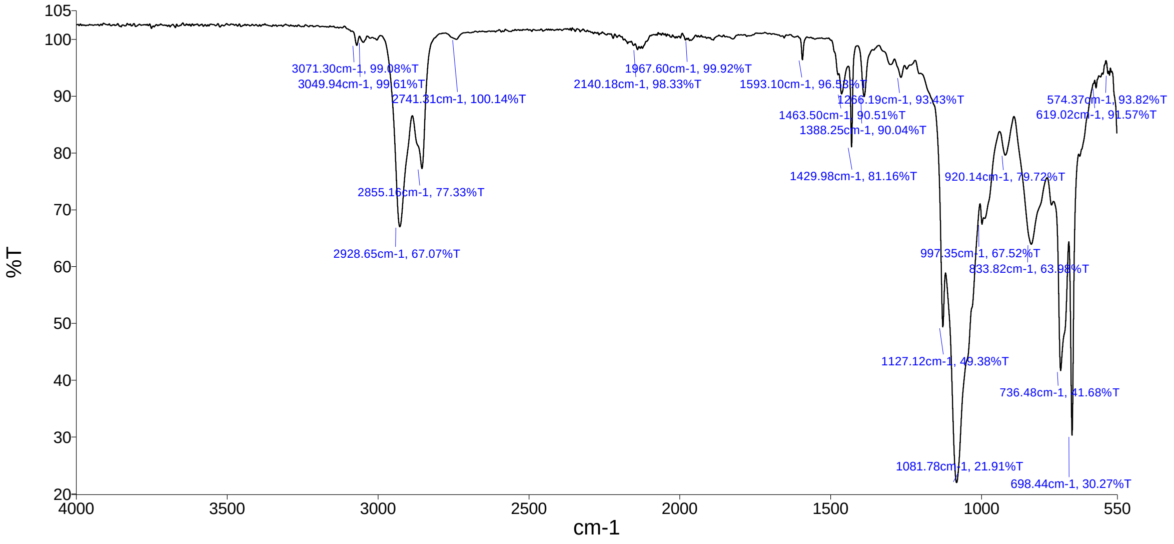

Figure S82: FTIR spectrum of **2j**.

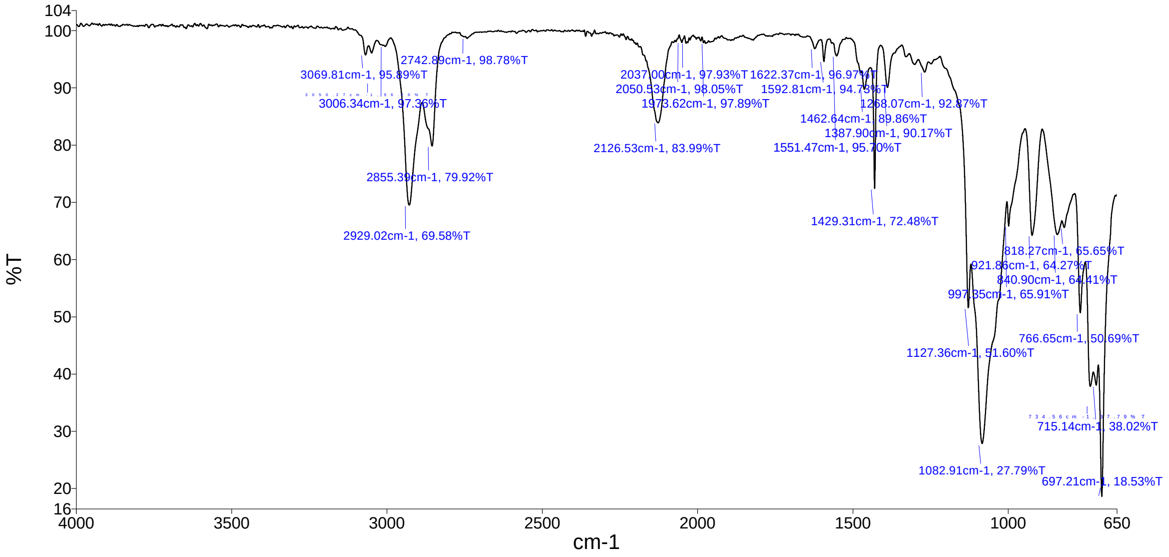

Figure S83: FTIR spectrum of **2k**.

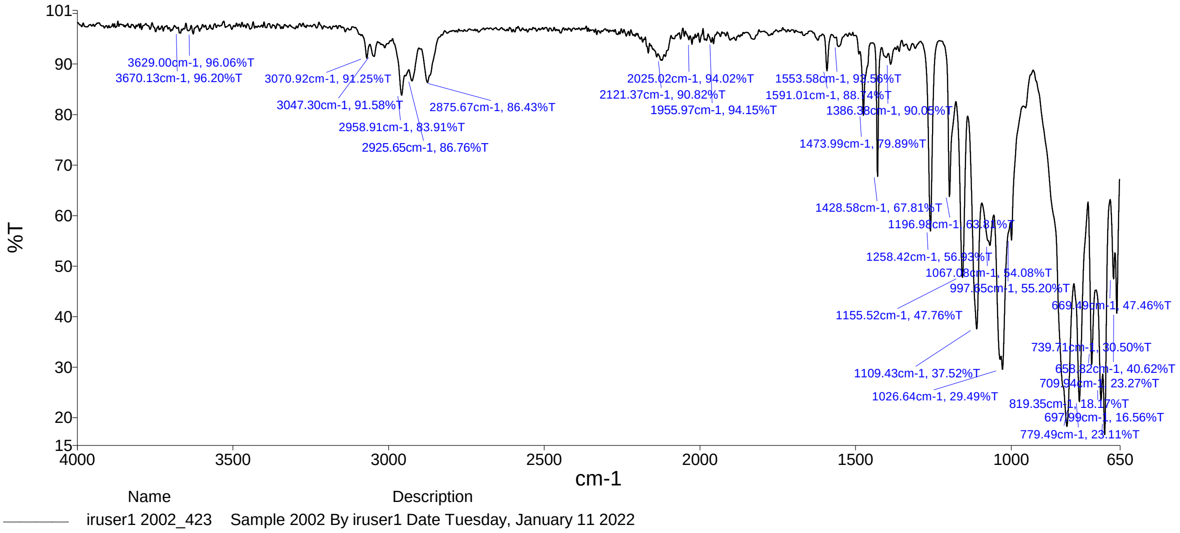

Figure S84: FTIR spectrum of **2l**.

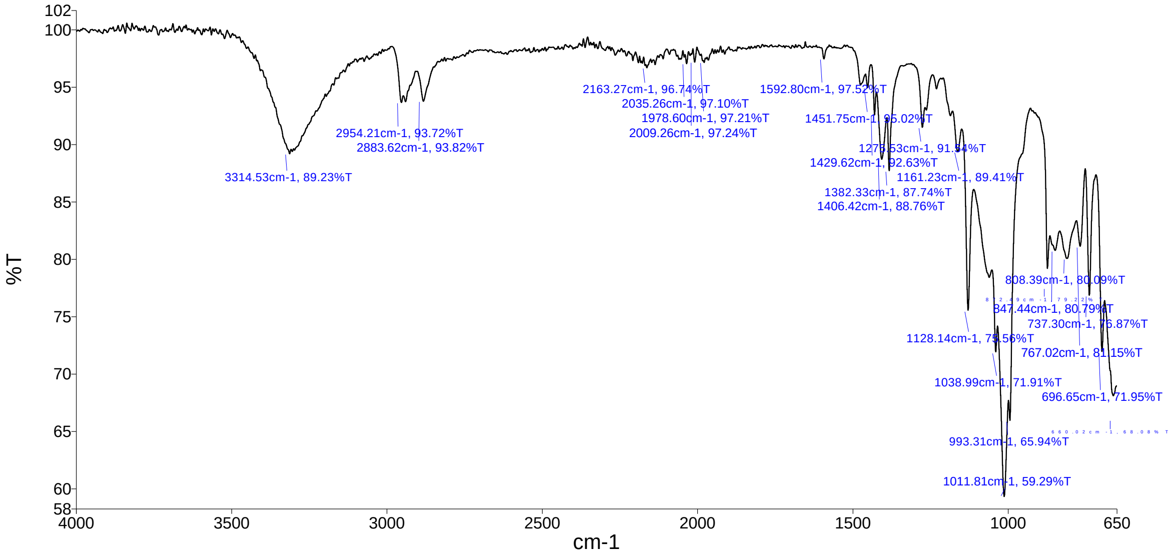

Figure S85: FTIR spectrum of **2m**.

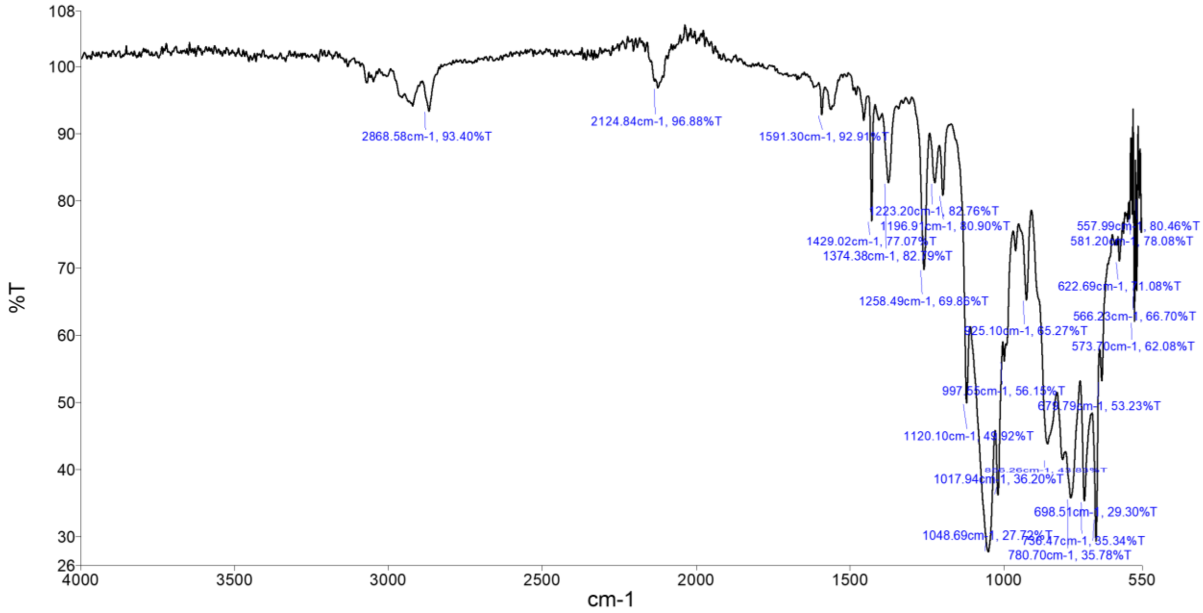

Figure S86: FTIR spectrum of **2n**.

Differential Scanning Calorimetry (DSC) data of 2a-2n

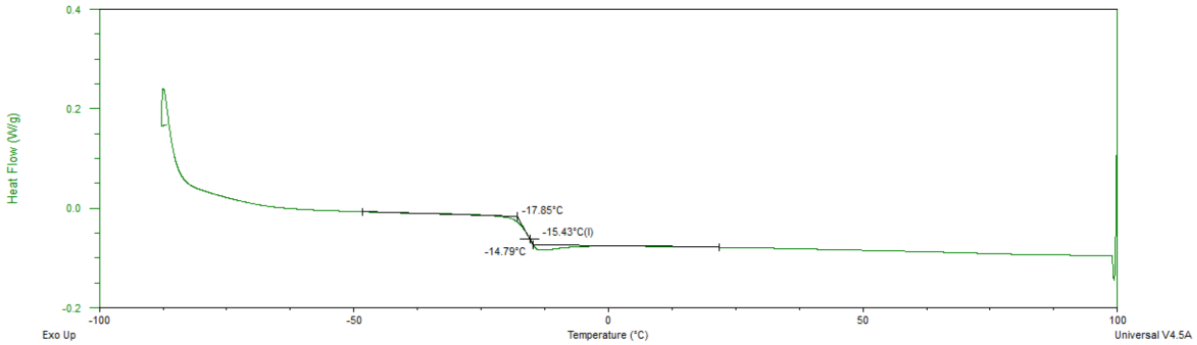

Figure S87: DCS trace showing the third heating cycle of **2a**.

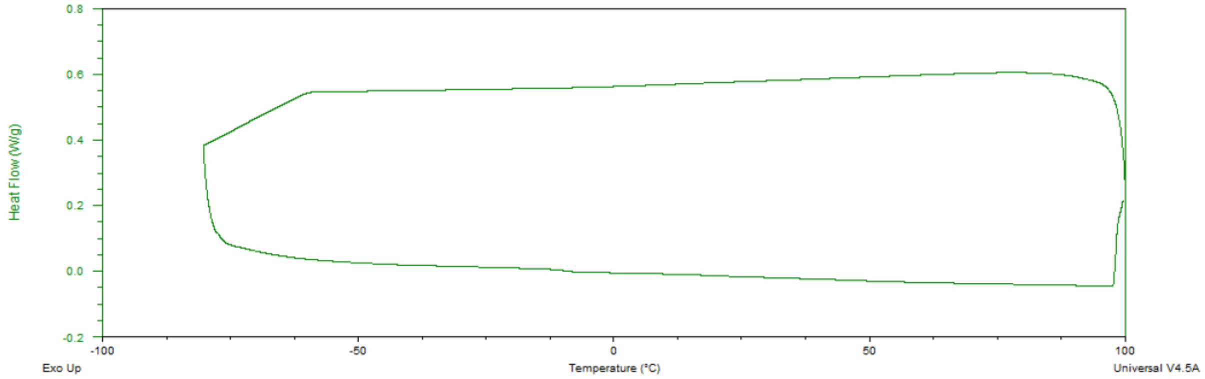

Figure S88: DCS trace showing the second and third heating cycles of **2b**.

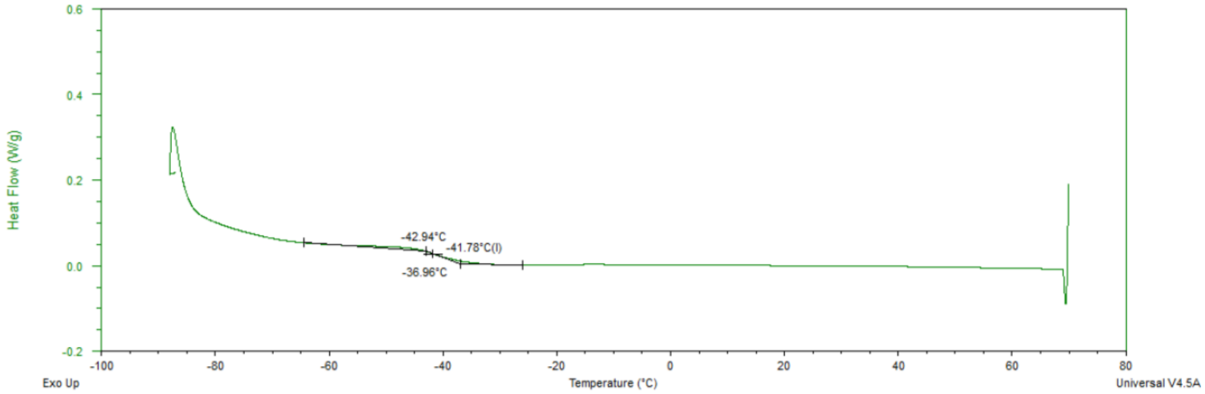

Figure S89: DCS trace showing the third heating cycle of **2c**.

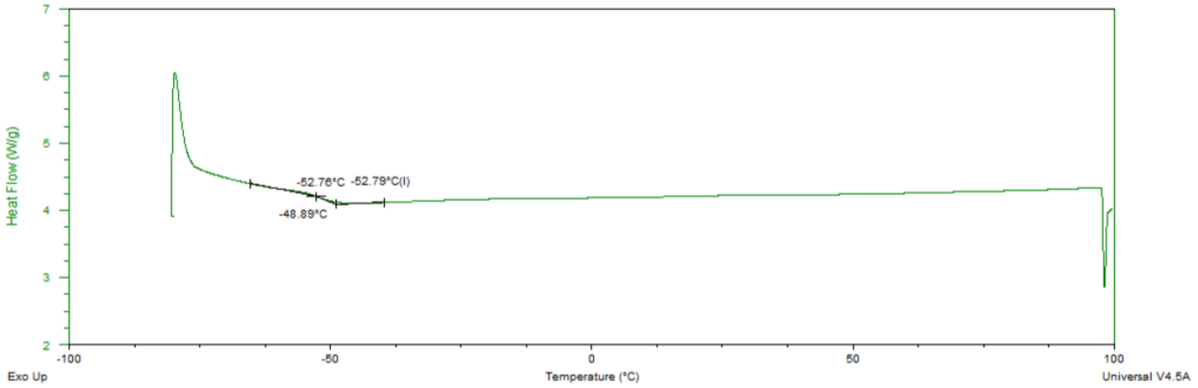

Figure S90: DCS trace showing the third heating cycle of **2d**.

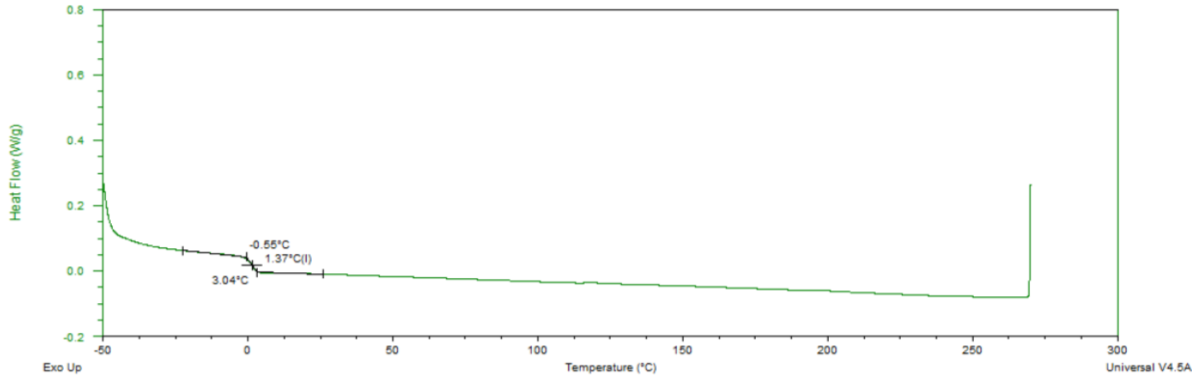

Figure S91: DCS trace showing the third heating cycle of **2e**.

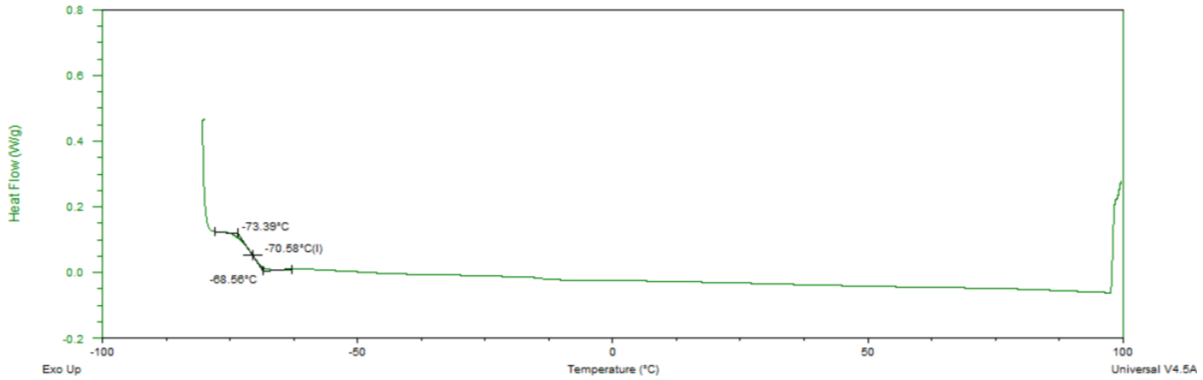

Figure S92: DCS trace showing the third heating cycle of **2f**.

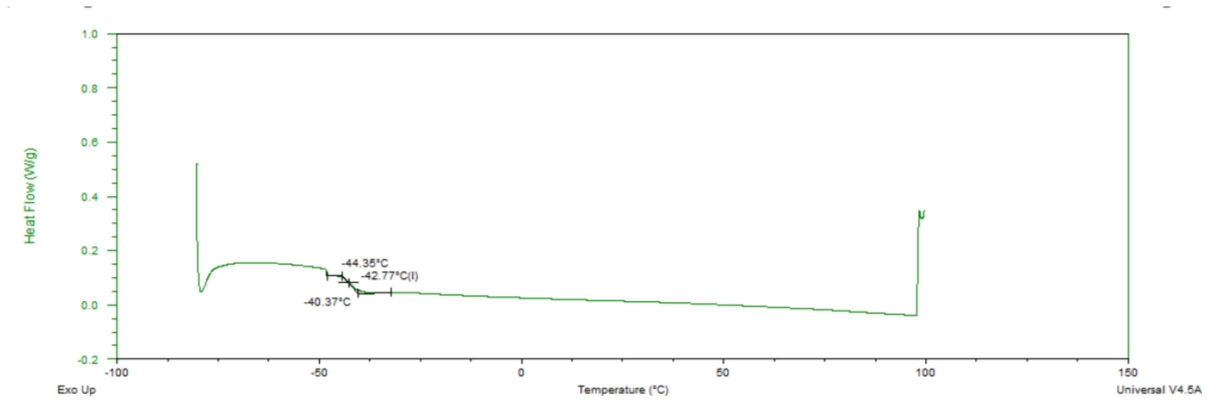

Figure S93: DCS trace showing the third heating cycle of **2g**.

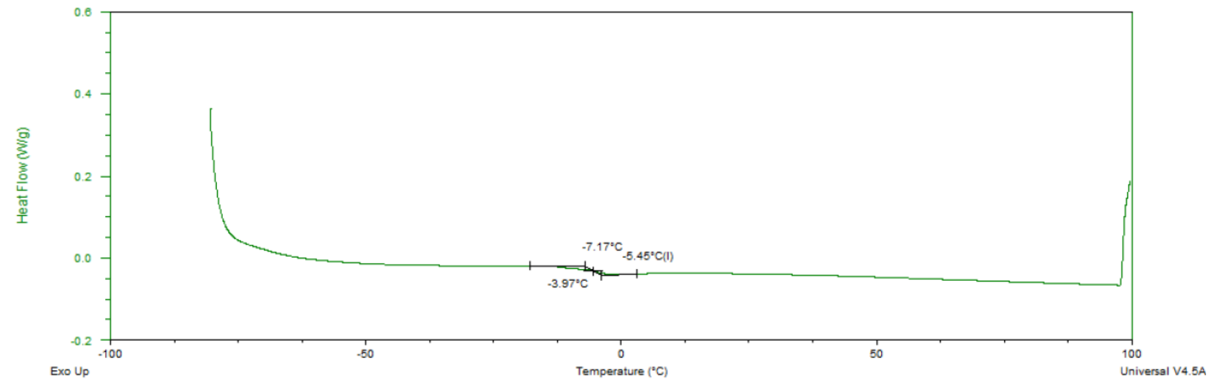

Figure S94: DCS trace showing the third heating cycle of **2h**.

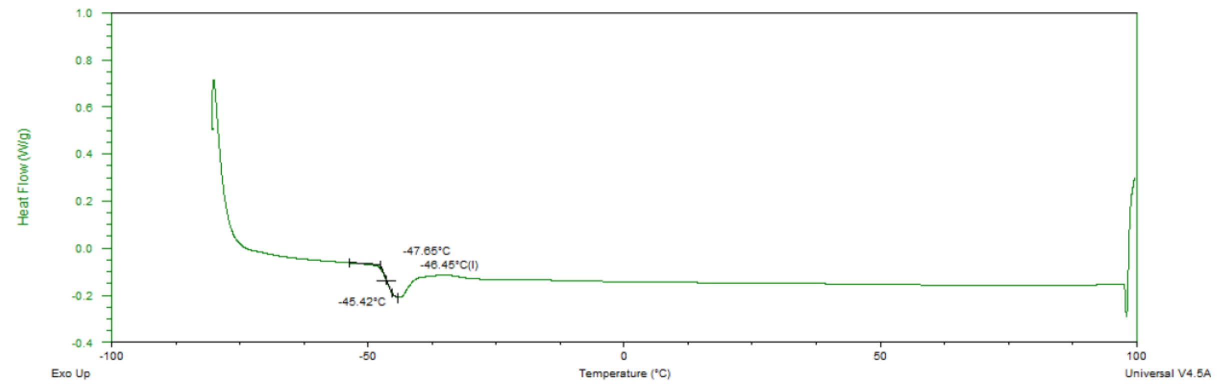

Figure S95: DCS trace showing the third heating cycle of **2i**.

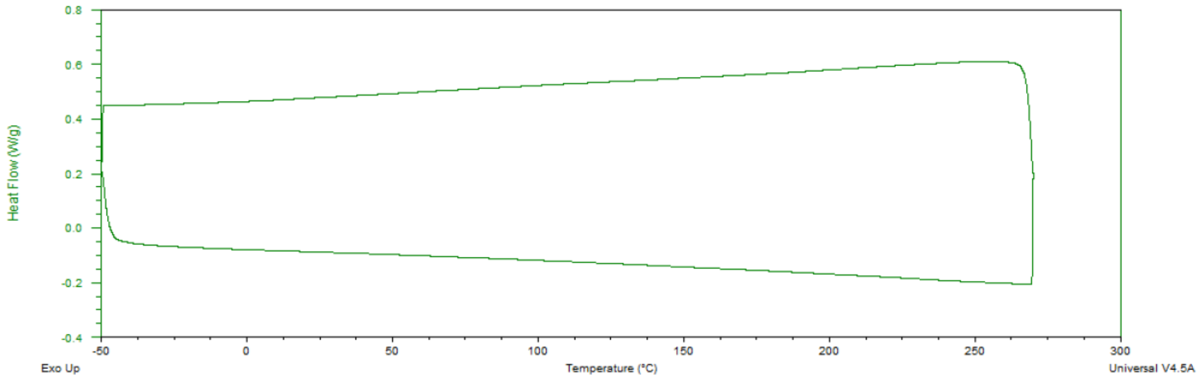

Figure S96: DCS trace showing the second and third heating cycles of **2j**.

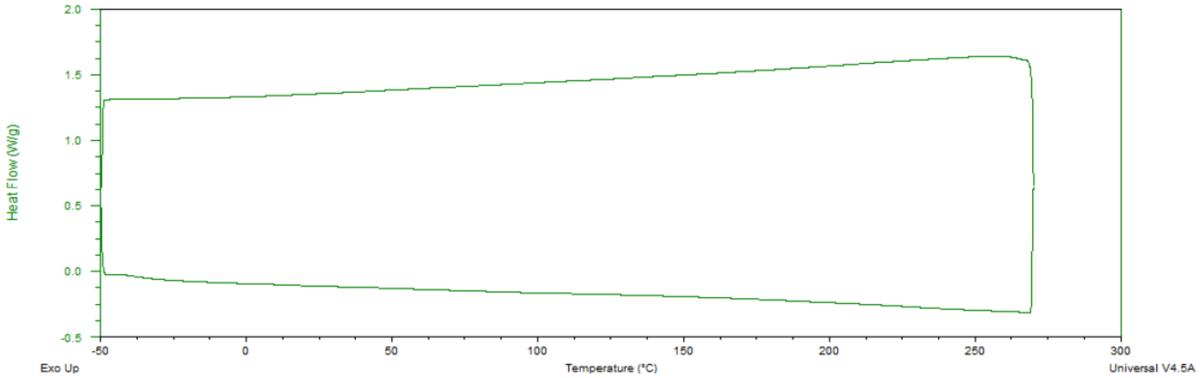

Figure S97: DCS trace showing the second and third heating cycles of **2k**.

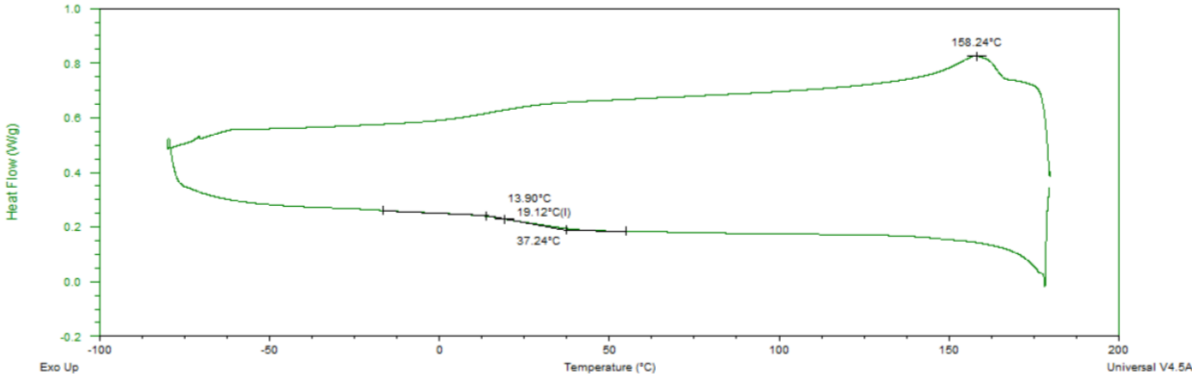

Figure S98: DCS trace showing the second and third heating cycles of **2l**.

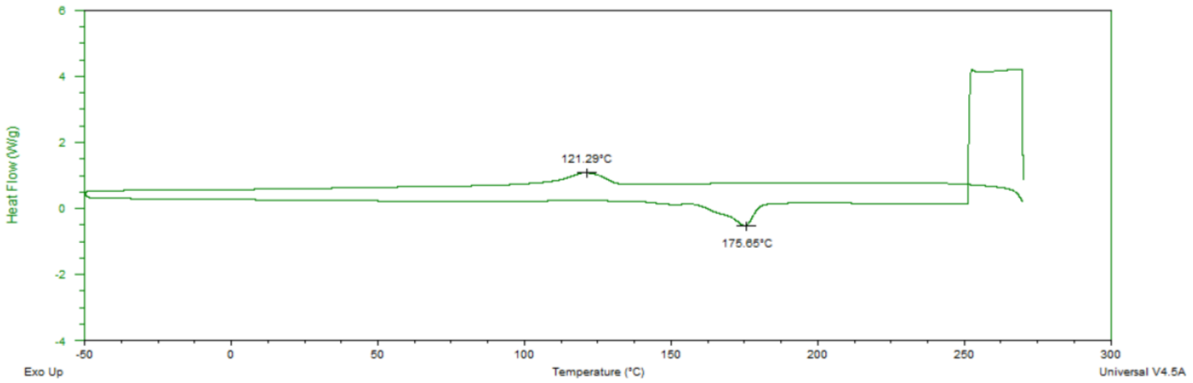

Figure S99: DCS trace showing the second and third heating cycles of **2m**.

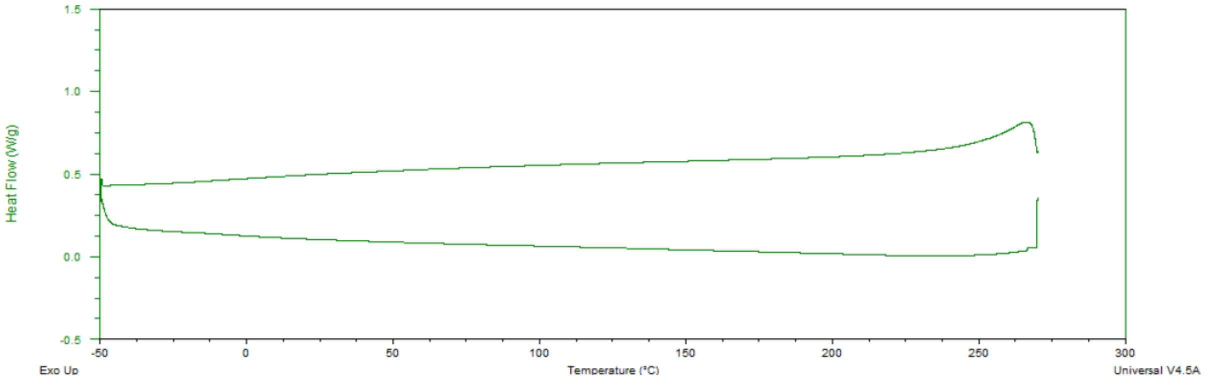

Figure S100: DCS trace showing the second and third heating cycles of **2n**.

Differential Scanning Calorimetry (DSC) data of vacuum polymerizations

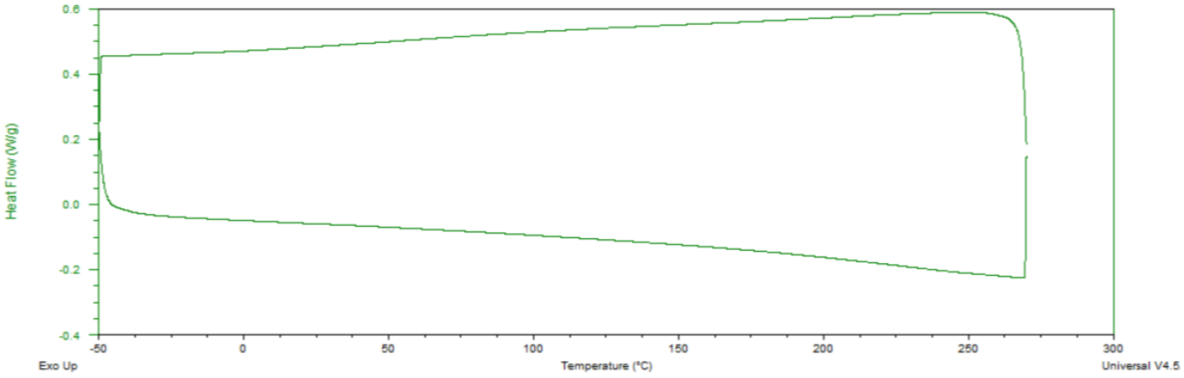

Figure S101: DCS trace showing the second and third heating cycles of **2b** after polymerization under dynamic vacuum conditions.

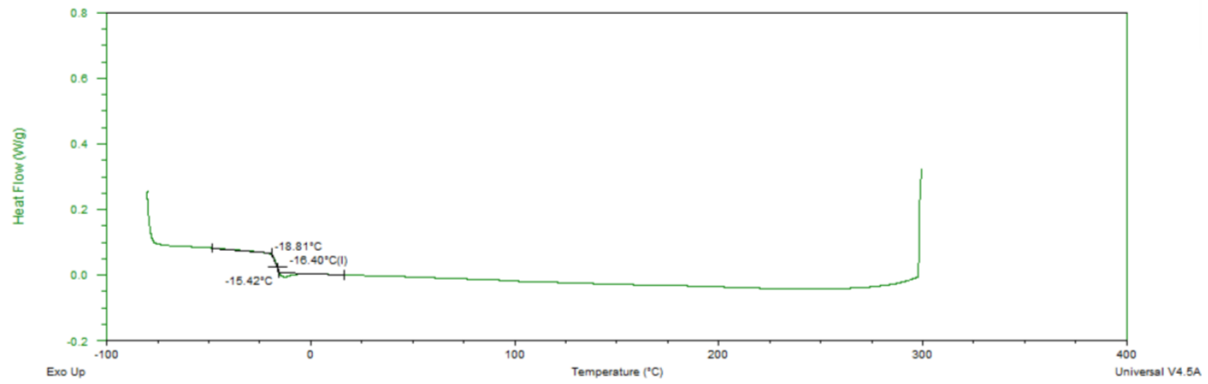

Figure S102: DCS trace showing the third heating cycle of **2d** after polymerization under dynamic vacuum conditions.

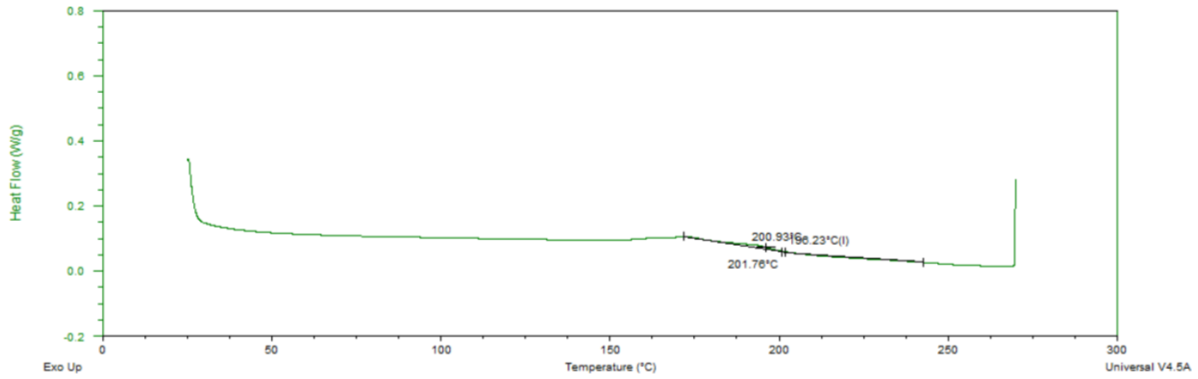

Figure S103: DCS trace showing the third heating cycle of **2f** after polymerization under dynamic vacuum conditions.

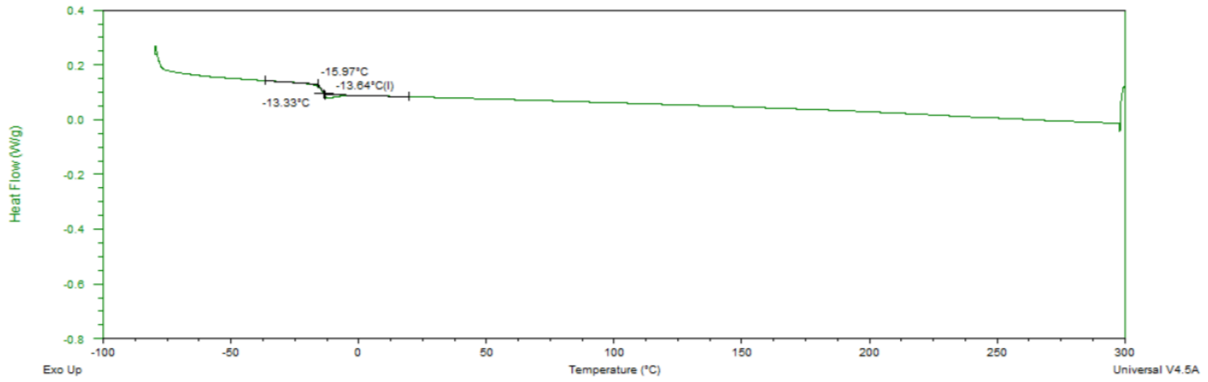

Figure S104: DCS trace showing the third heating cycle of **2g** after polymerization under dynamic vacuum conditions.

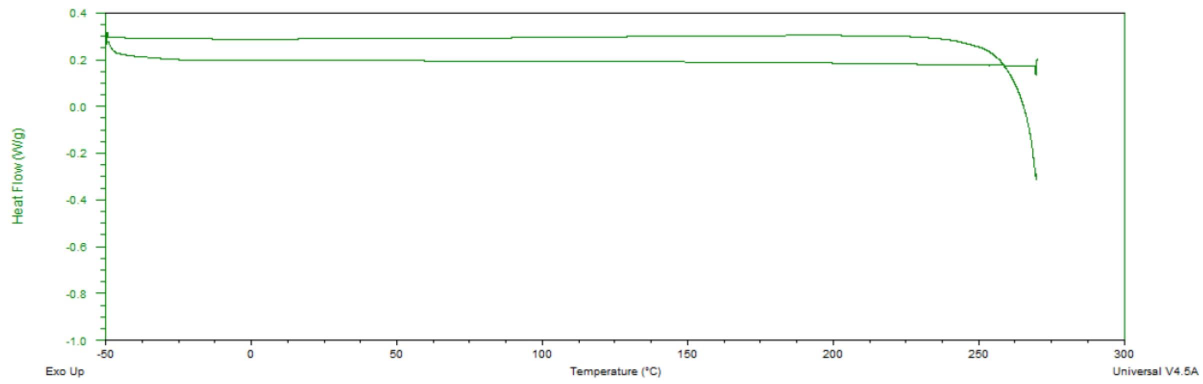

Figure S105: DCS trace showing the second and third heating cycles of **2h** after polymerization under dynamic vacuum conditions.

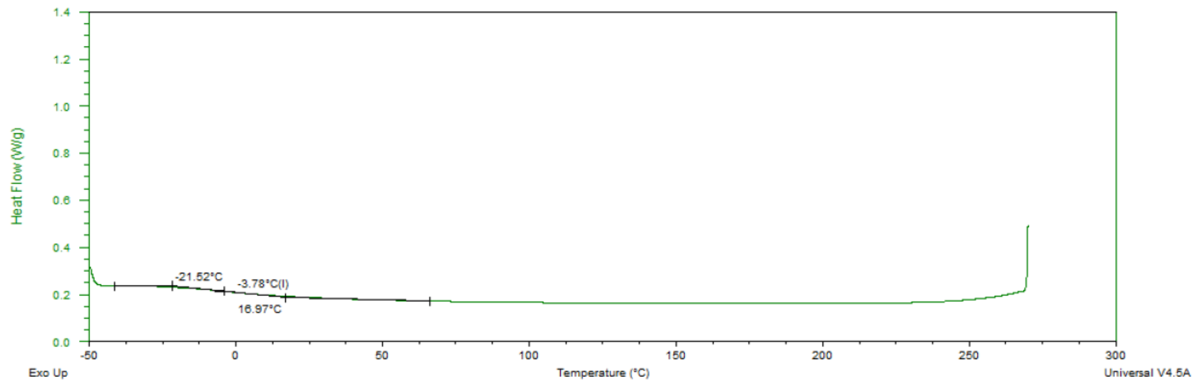

Figure S106: DCS trace showing the third heating cycle of **2n** after polymerization under dynamic vacuum conditions.

Thermogravimetric Analysis (TGA) Data of 2a-2n

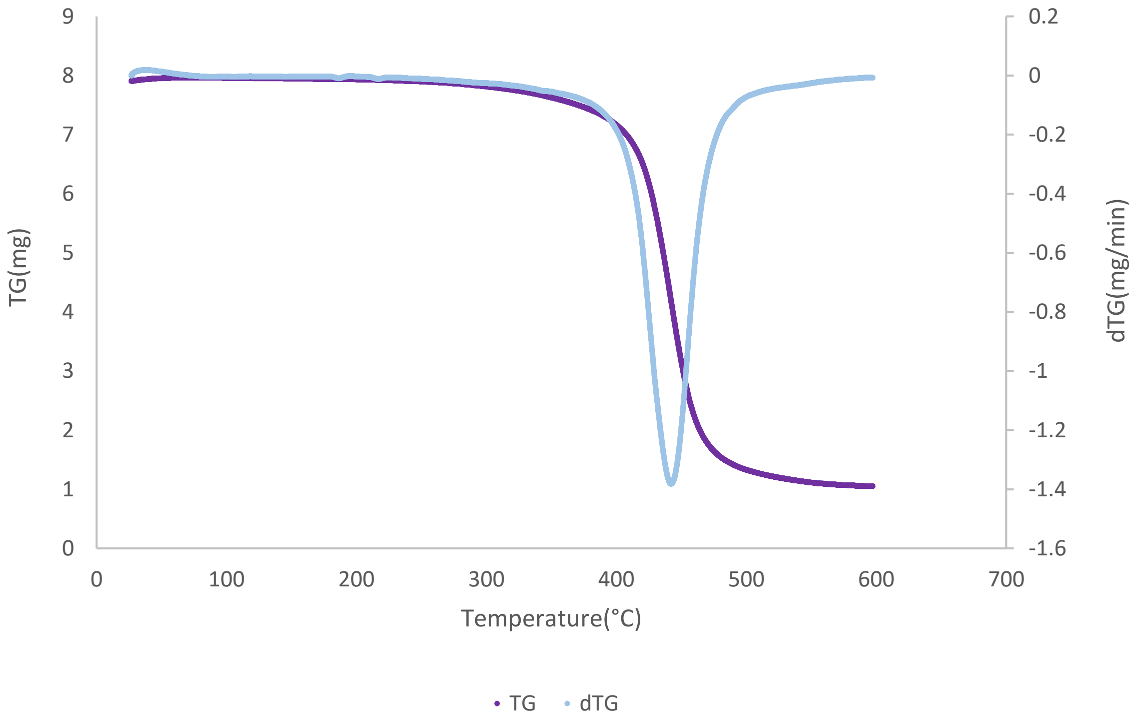

Figure S107: TGA analysis of **2a** showing the plot of mass *versus* temperature (TG) and the derivative of mass loss *versus* temperature (dTG).

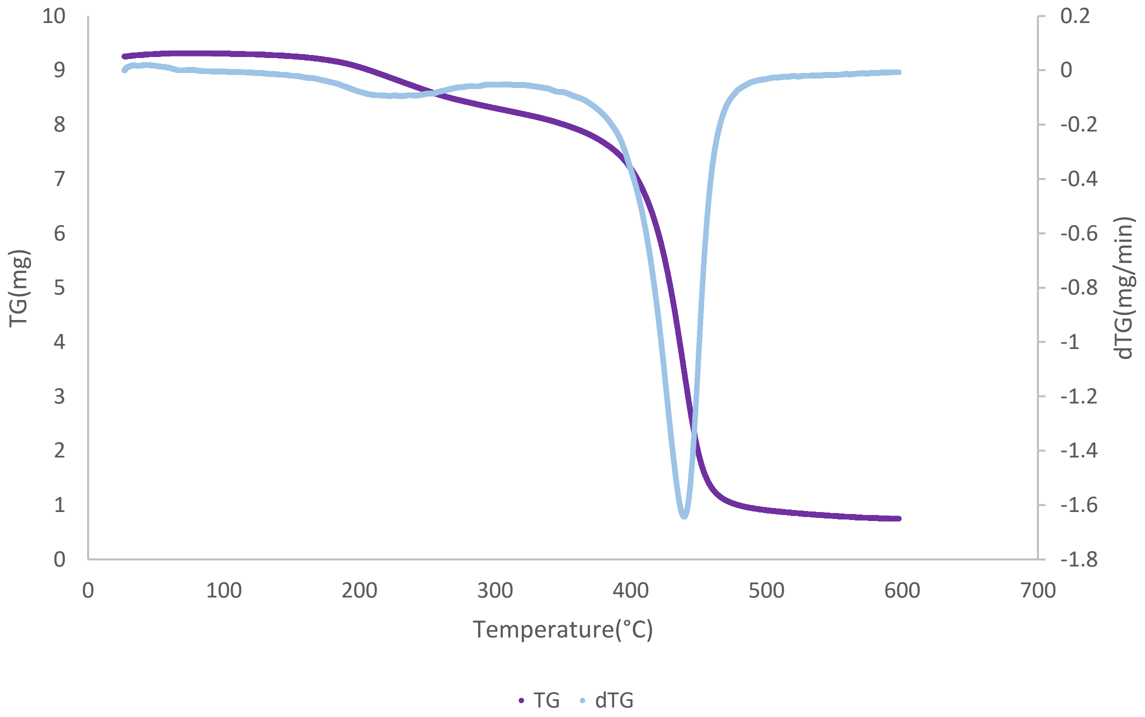

Figure S108: TGA analysis of **2b** showing the plot of mass *versus* temperature (TG) and the derivative of mass loss *versus* temperature (dTG).

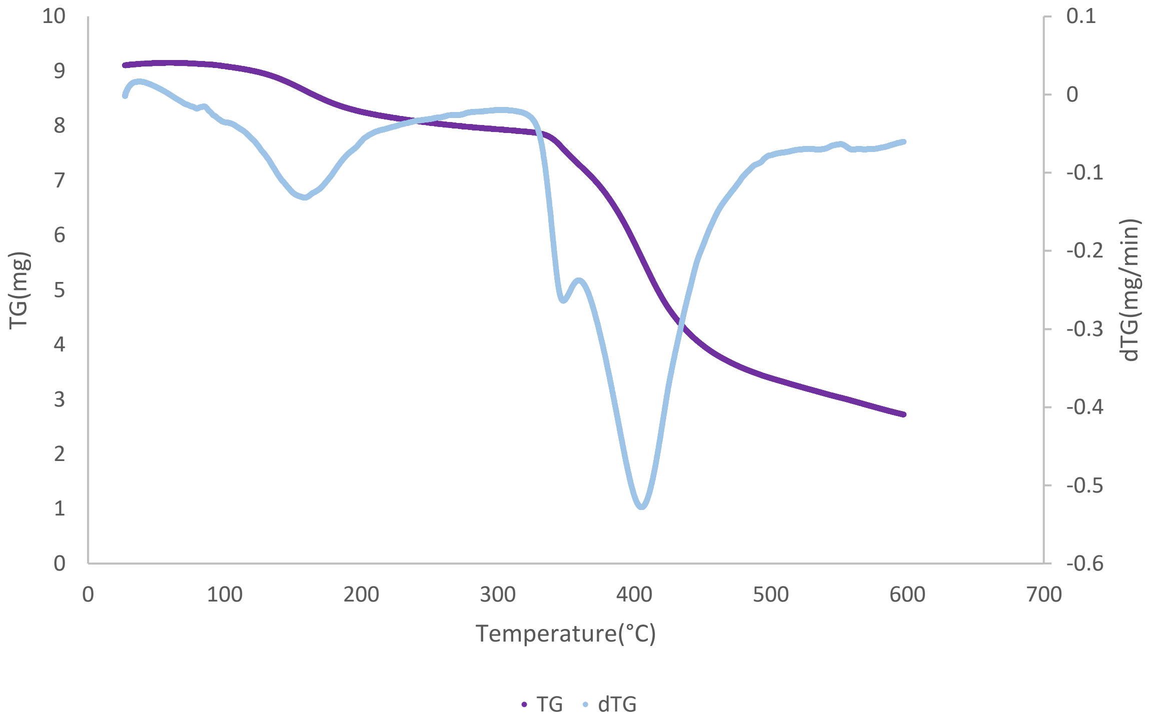

Figure S109: TGA analysis of **2c** showing the plot of mass *versus* temperature (TG) and the derivative of mass loss *versus* temperature (dTG).

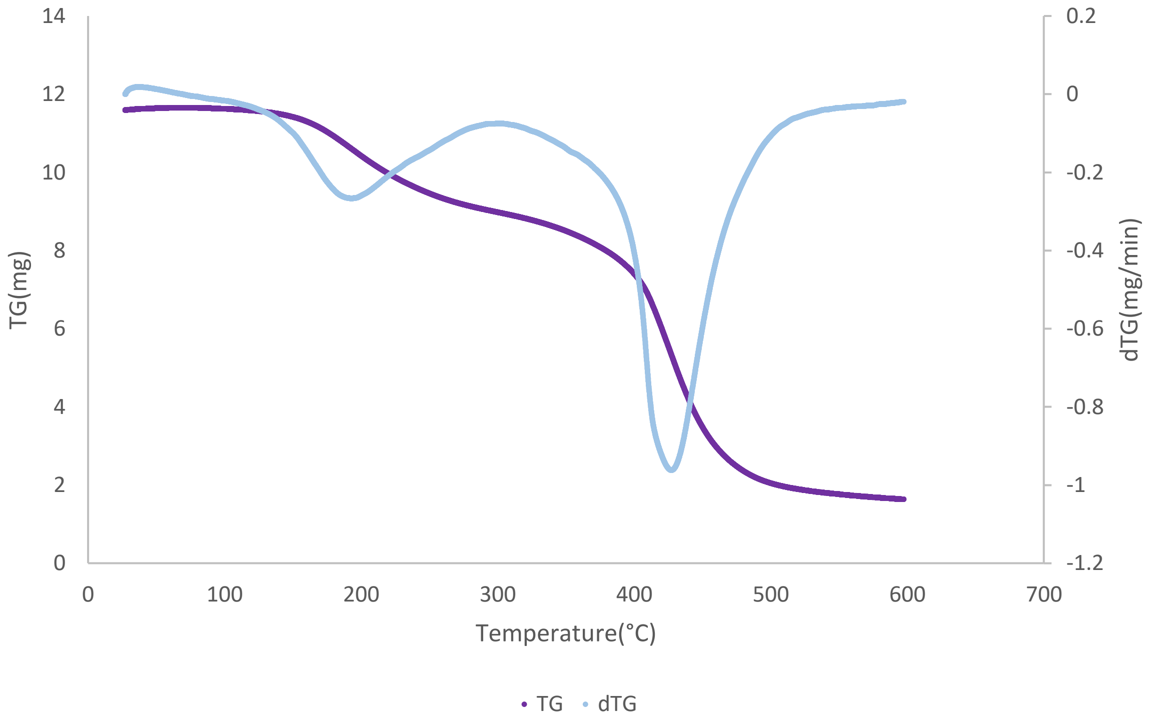

Figure S110: TGA analysis of **2d** showing the plot of mass *versus* temperature (TG) and the derivative of mass loss *versus* temperature (dTG).

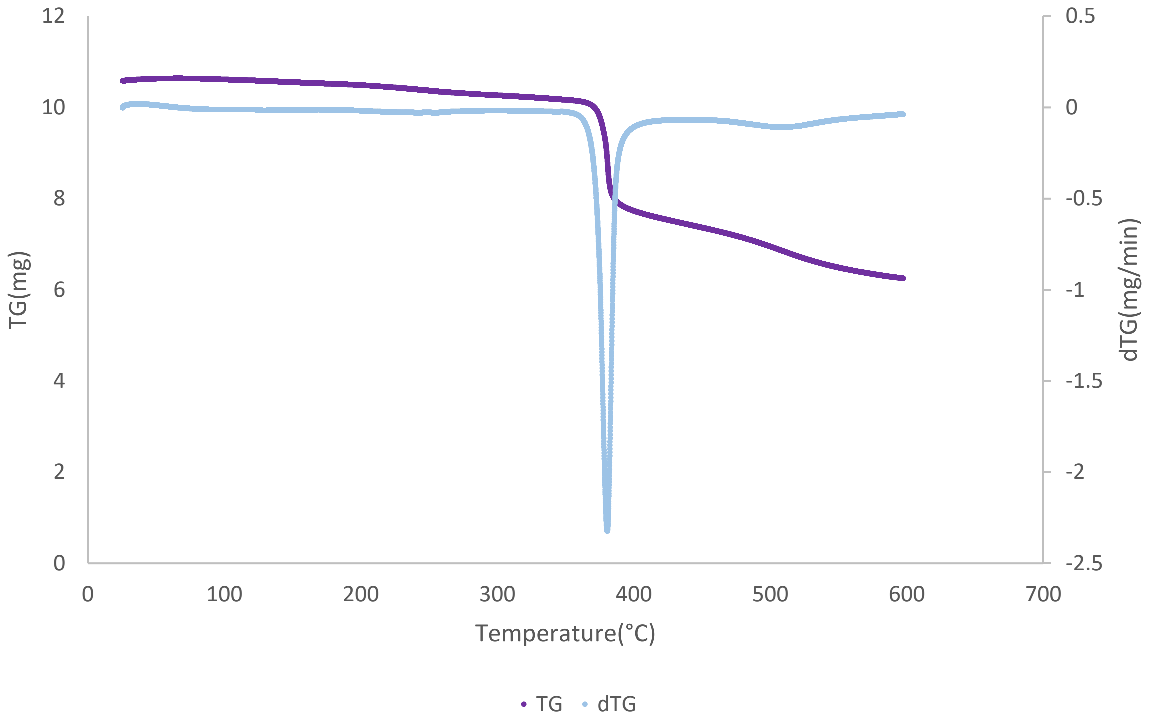

Figure S111: TGA analysis of **2e** showing the plot of mass *versus* temperature (TG) and the derivative of mass loss *versus* temperature (dTG).

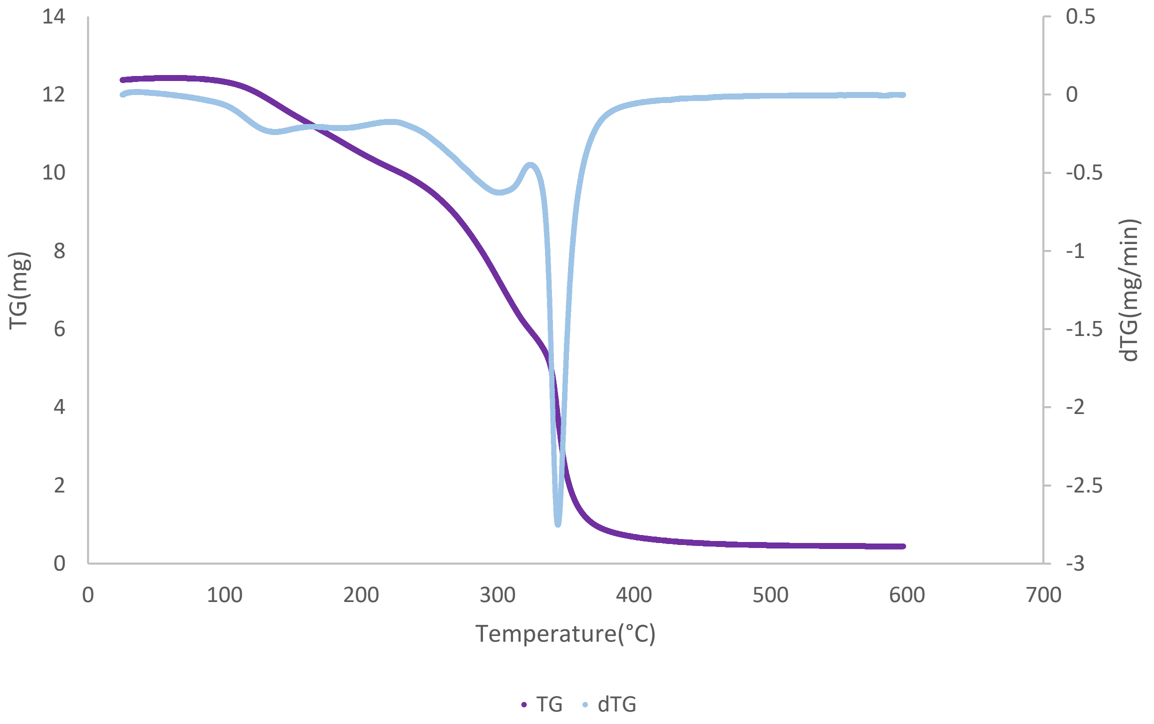

Figure S112: TGA analysis of **2f** showing the plot of mass *versus* temperature (TG) and the derivative of mass loss *versus* temperature (dTG).

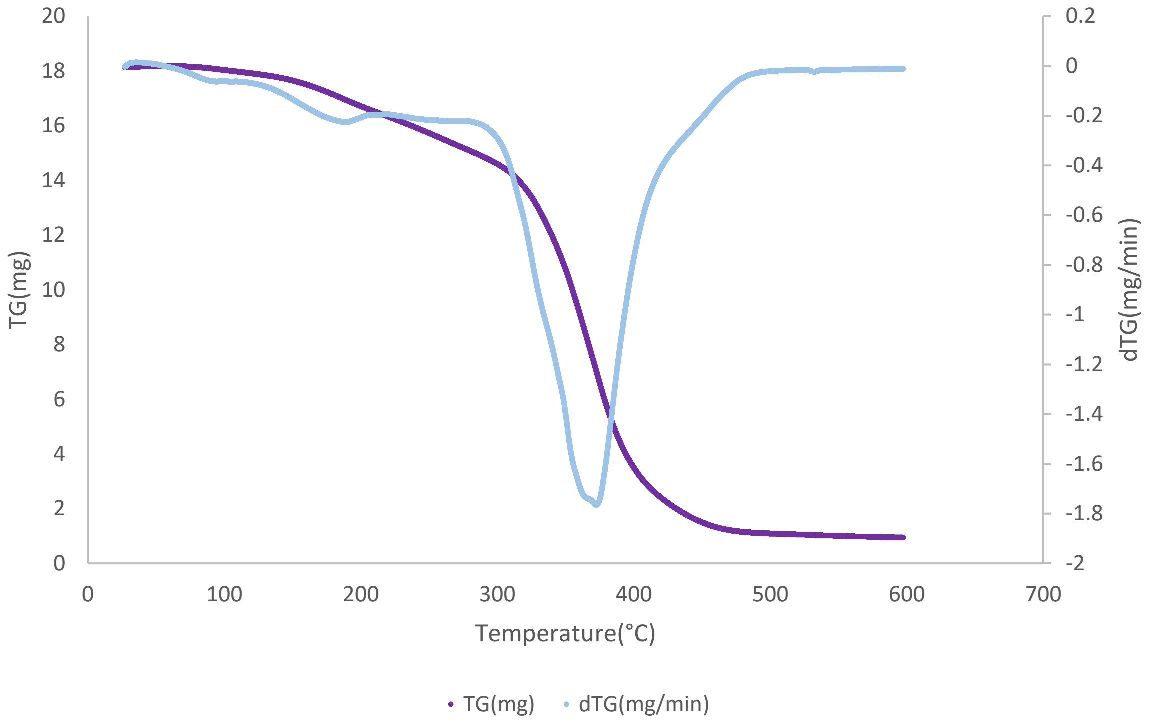

Figure S113: TGA analysis of **2g** showing the plot of mass *versus* temperature (TG) and the derivative of mass loss *versus* temperature (dTG).

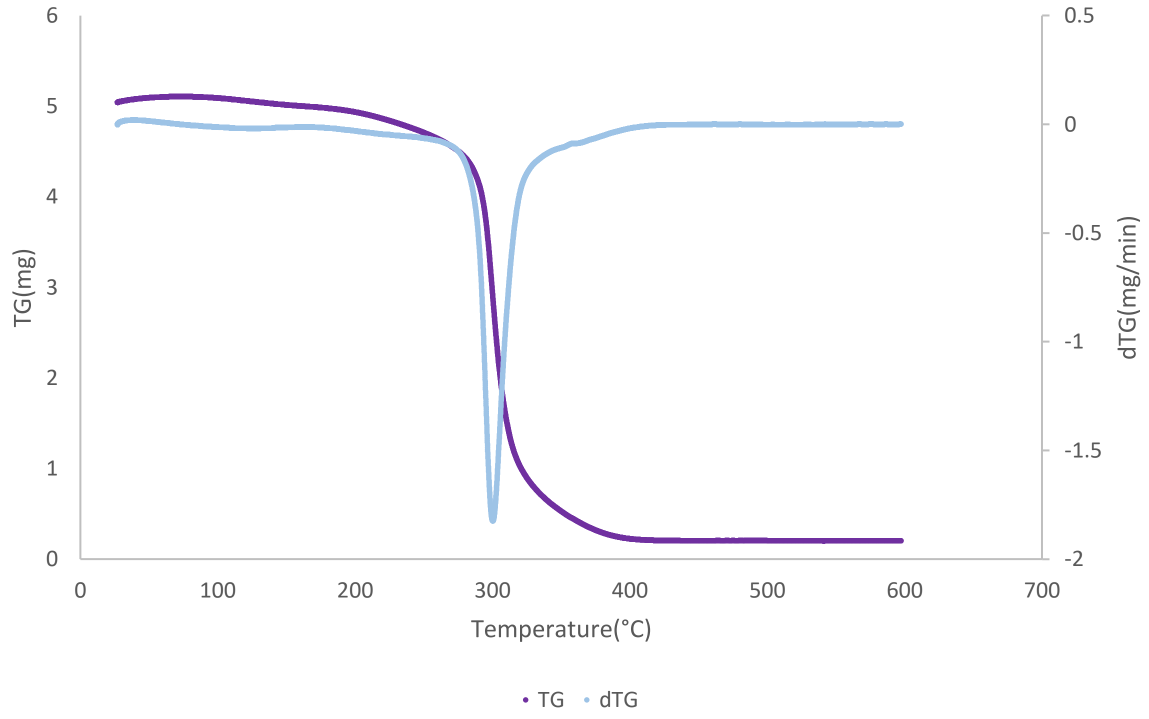

Figure S114: TGA analysis of **2h** showing the plot of mass *versus* temperature (TG) and the derivative of mass loss *versus* temperature (dTG).

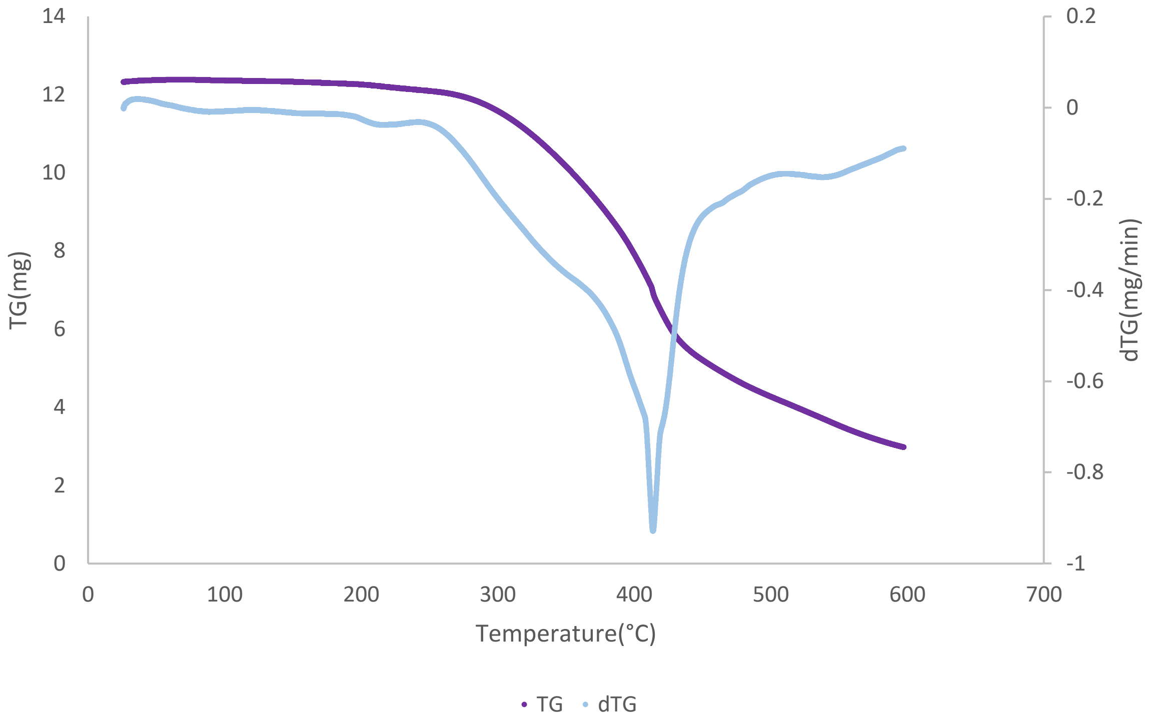

Figure S115: TGA analysis of **2i** showing the plot of mass *versus* temperature (TG) and the derivative of mass loss *versus* temperature (dTG).

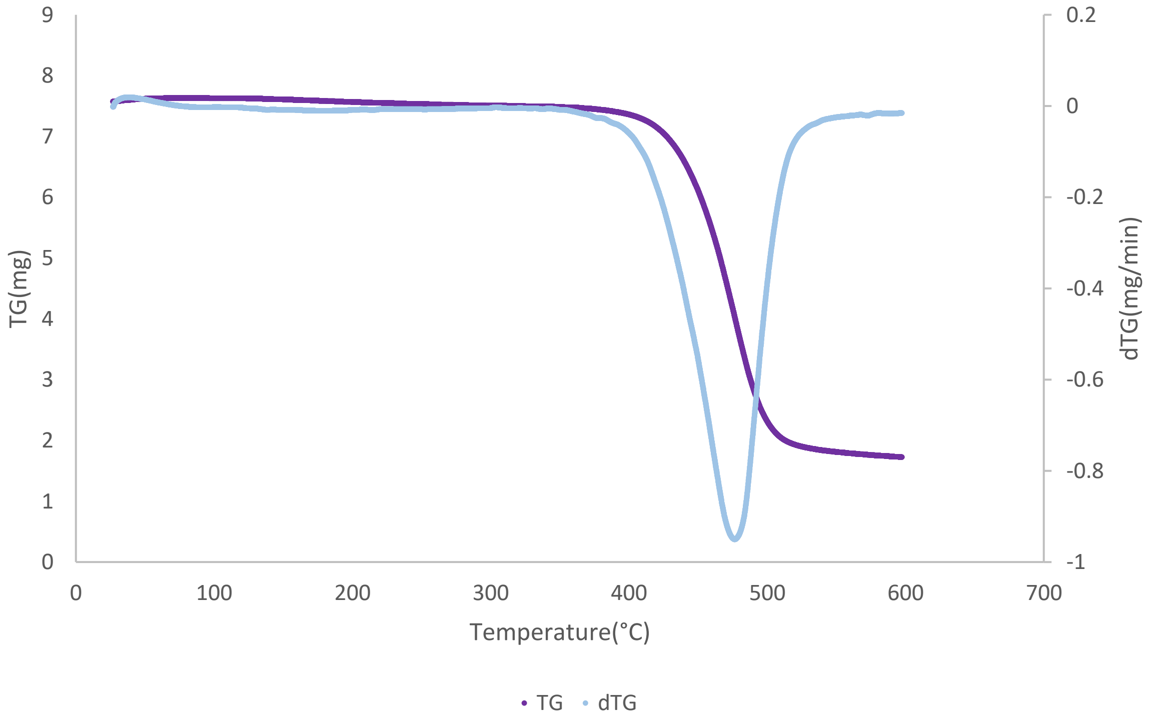

Figure S116: TGA analysis of **2j** showing the plot of mass *versus* temperature (TG) and the derivative of mass loss *versus* temperature (dTG).

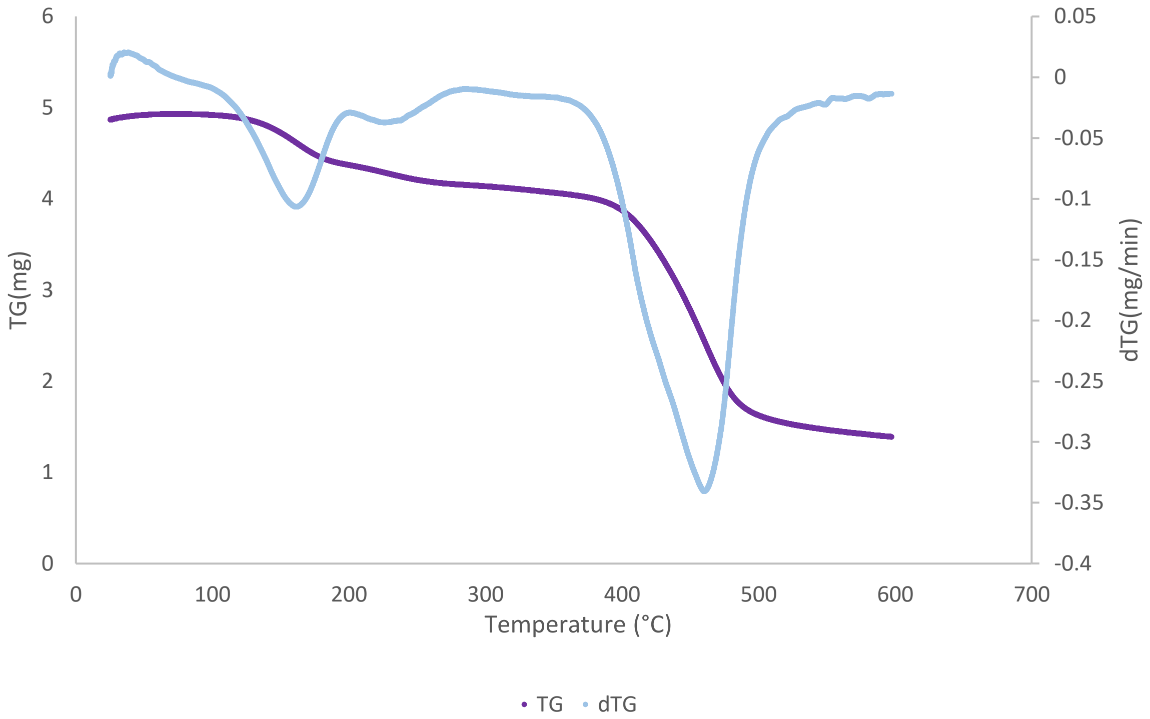

Figure S117: TGA analysis of **2k** showing the plot of mass *versus* temperature (TG) and the derivative of mass loss *versus* temperature (dTG).

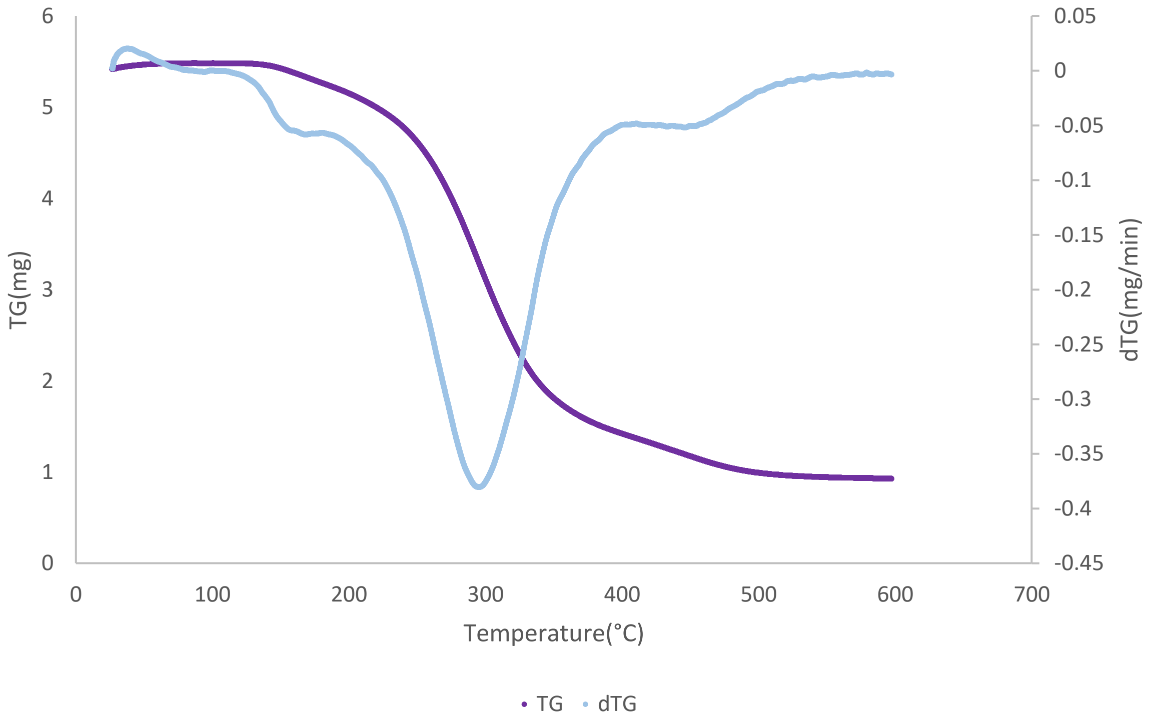

Figure S118: TGA analysis of **2l** showing the plot of mass *versus* temperature (TG) and the derivative of mass loss *versus* temperature (dTG).

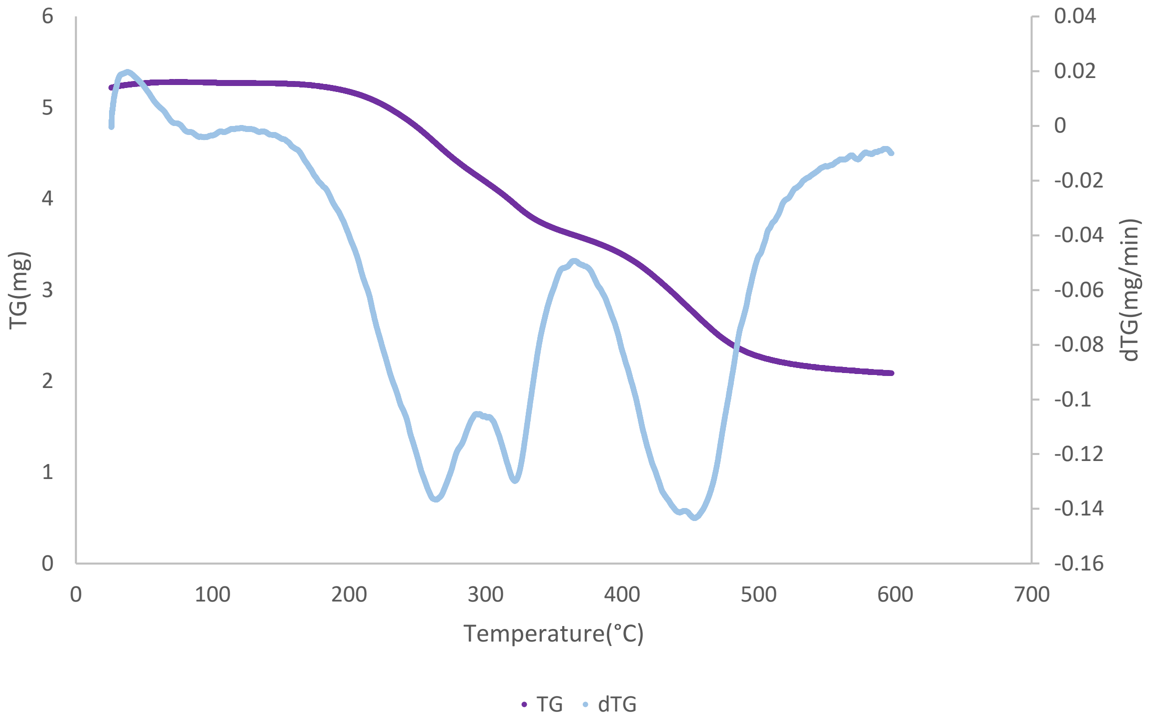

Figure S119: TGA analysis of **2m** showing the plot of mass *versus* temperature (TG) and the derivative of mass loss *versus* temperature (dTG).

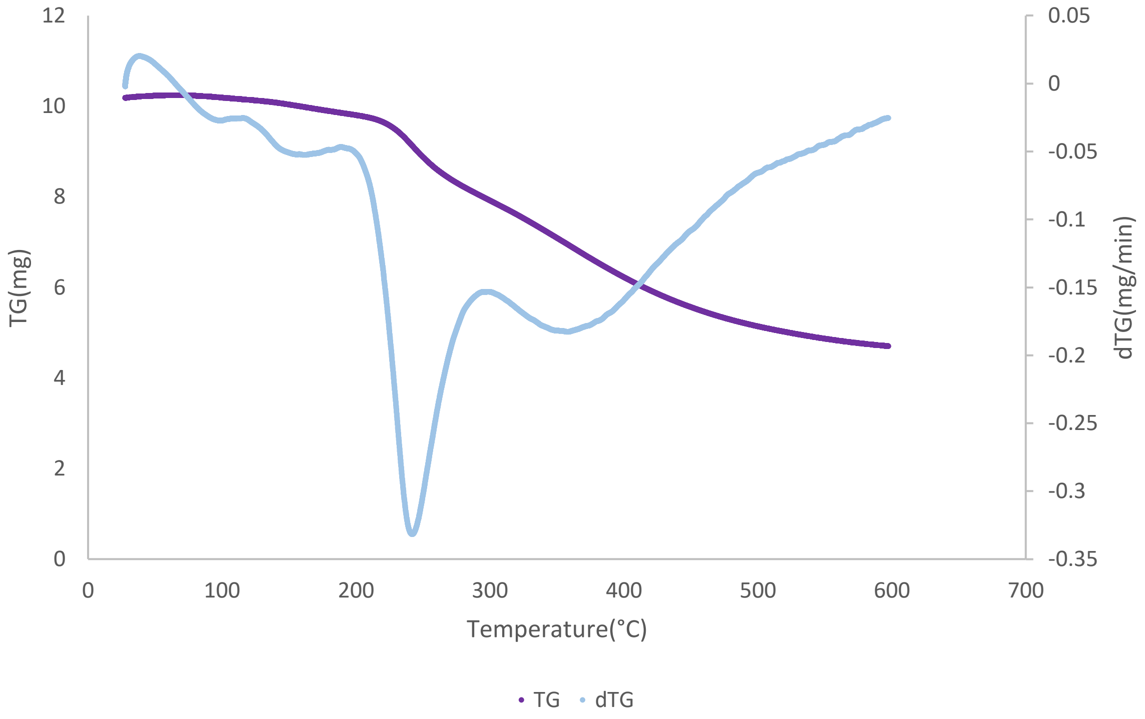

Figure S120: TGA analysis of **2n** showing the plot of mass *versus* temperature (TG) and the derivative of mass loss *versus* temperature (dTG).

Thermogravimetric Analysis (TGA) data of vacuum polymerization reactions

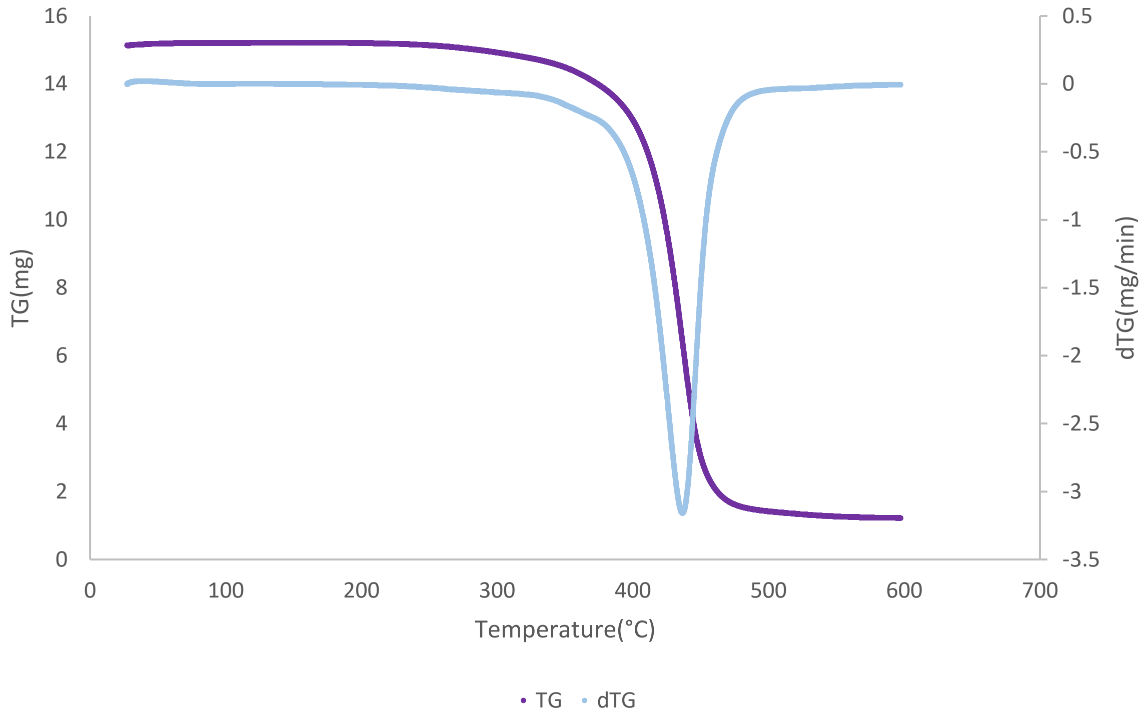

Figure S121: TGA analysis of **2b** after polymerization under dynamic vacuum conditions showing the plot of mass *versus* temperature (TG) and the derivative of mass loss *versus* temperature (dTG).

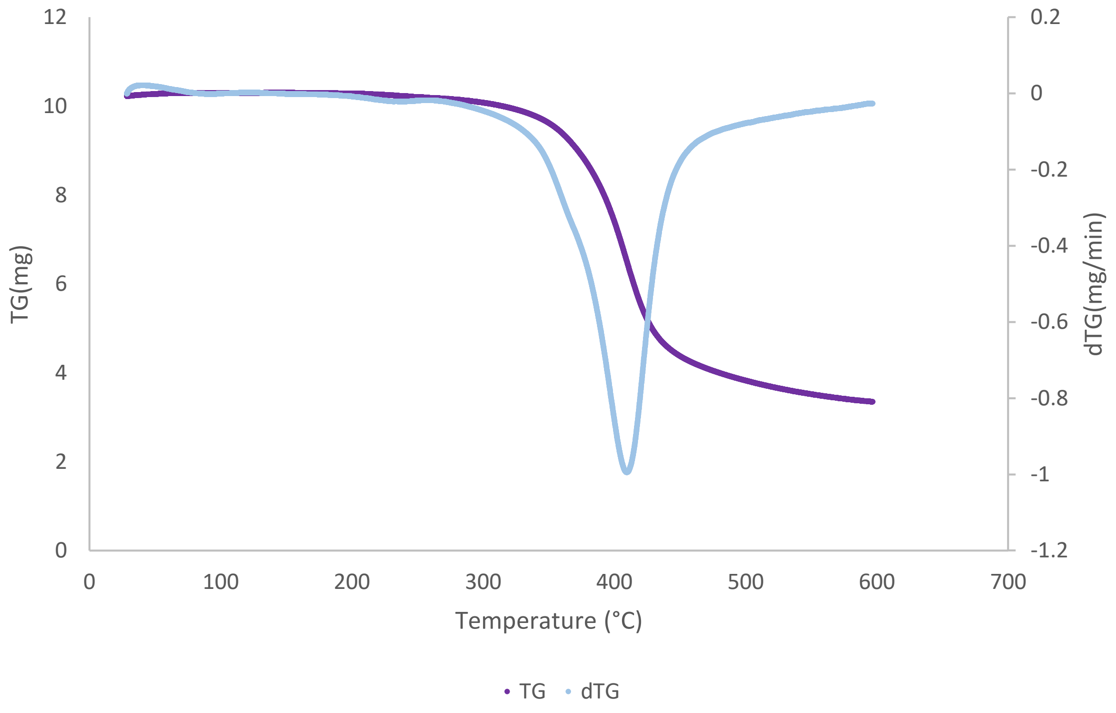

Figure S122: TGA analysis of **2d** after polymerization under dynamic vacuum conditions showing the plot of mass *versus* temperature (TG) and the derivative of mass loss *versus* temperature (dTG).

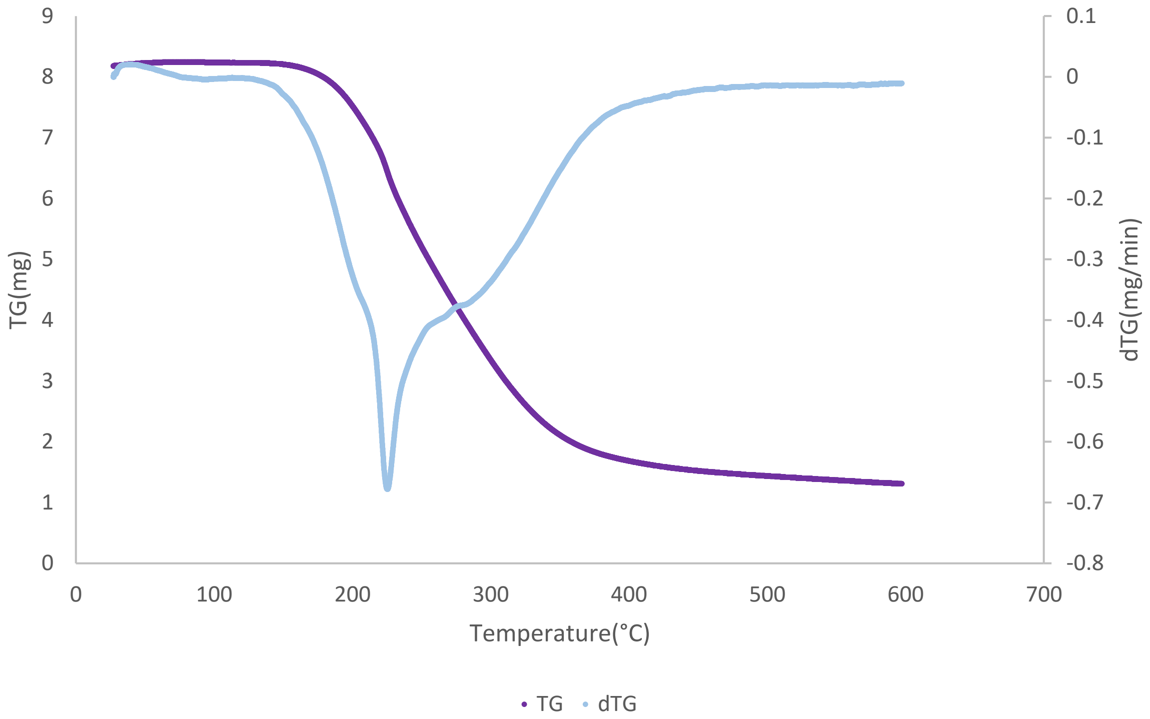

Figure S123: TGA analysis of **2f** after polymerization under dynamic vacuum conditions showing the plot of mass *versus* temperature (TG) and the derivative of mass loss *versus* temperature (dTG).

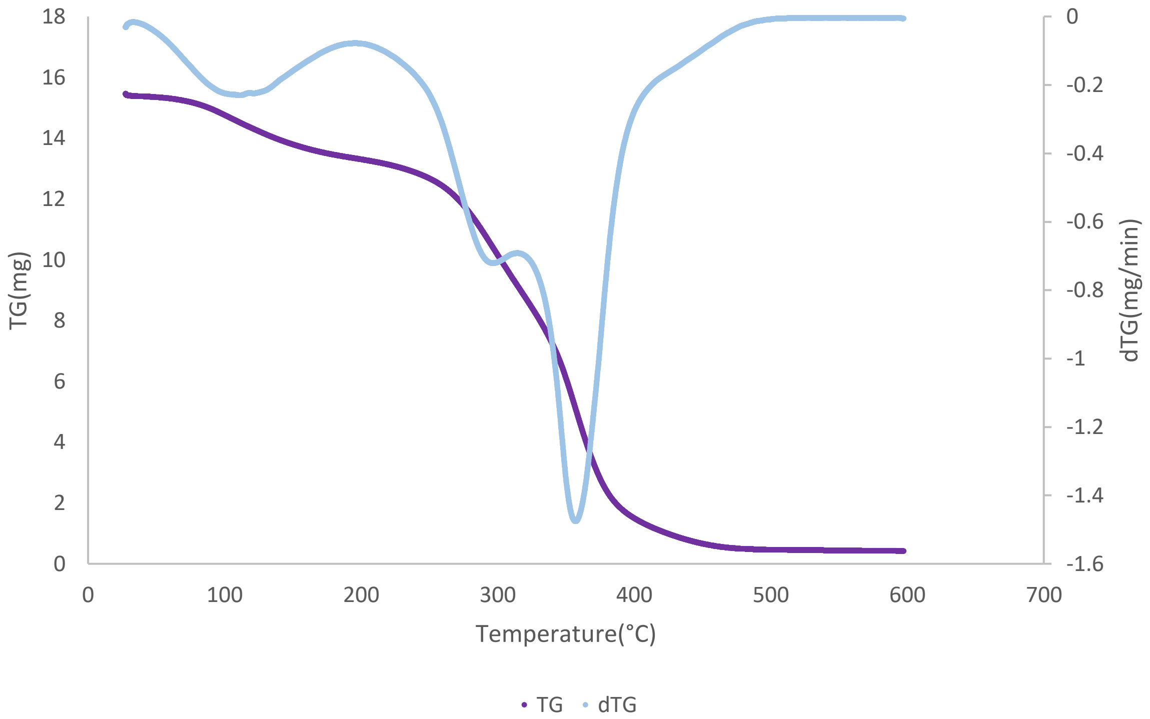

Figure S124: TGA analysis of **2g** after polymerization under dynamic vacuum conditions showing the plot of mass *versus* temperature (TG) and the derivative of mass loss *versus* temperature (dTG).

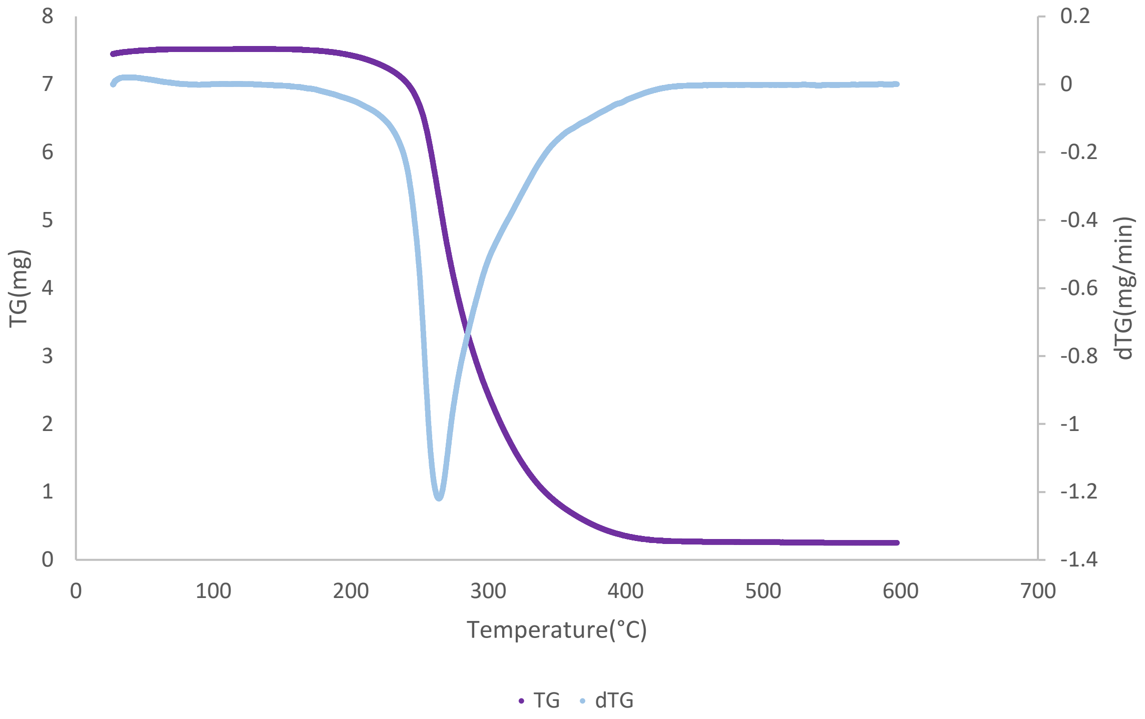

Figure S125: TGA analysis of **2h** after polymerization under dynamic vacuum conditions showing the plot of mass *versus* temperature (TG) and the derivative of mass loss *versus* temperature (dTG).

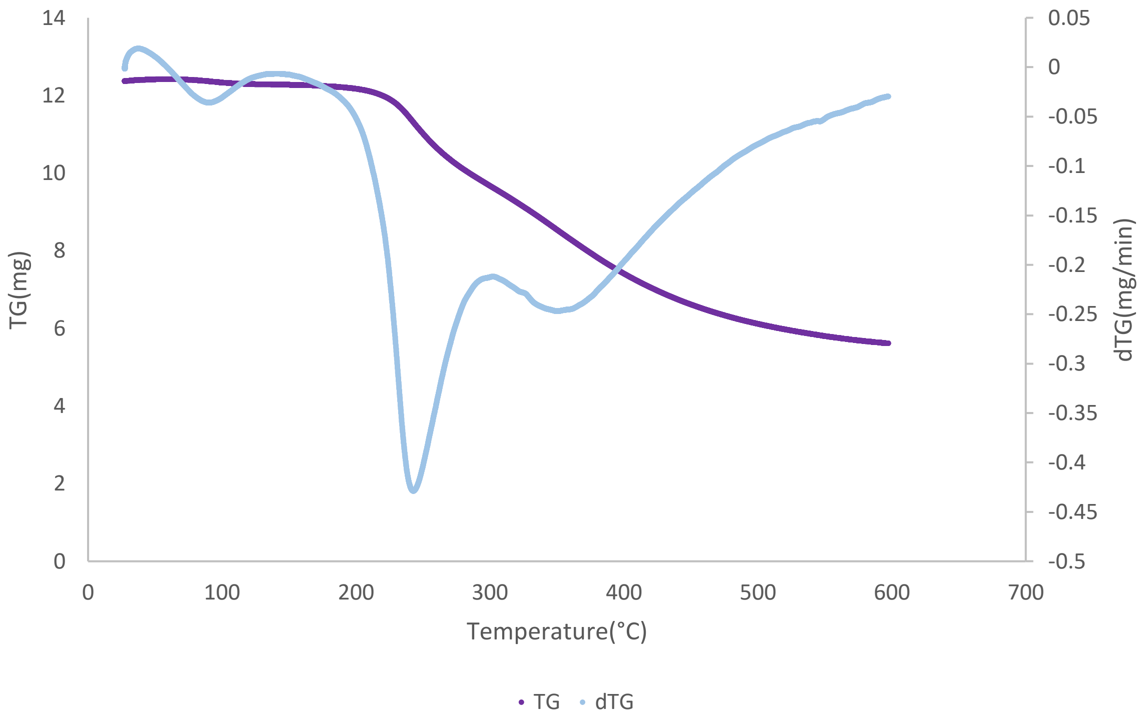

Figure S126: TGA analysis of **2n** after polymerization under dynamic vacuum conditions showing the plot of mass *versus* temperature (TG) and the derivative of mass loss *versus* temperature (dTG).

References

1 N. T. Coles, M. F. Mahon and R. L. Webster, *Organometallics*, 2017, **36**, 2262–2268.

2 T. J. J. Sciarone, A. Meetsma and B. Hessen, *Inorganica Chim. Acta*, 2006, **359**, 1815–1825.

3 T. R. Dugan, E. Bill, K. C. Macleod, W. W. Brennessel and P. L. Holland, *Inorg. Chem.*, 2014, **53**, 2370–2380.

4 B. Arkles and P. J. Launer, in *Silicon Compounds: Silanes and Silicones*, Gelest Inc., Morrisville, 2013, pp. 175–178.
